# Quantum-classical deep learning hybrid architecture with graphene-printed low-cost capacitive sensor for essential tremor detection

**DOI:** 10.1038/s41598-025-06359-1

**Published:** 2025-06-20

**Authors:** Javier Villalba-Díez, Ana González-Marcos

**Affiliations:** 1 Fakultät Wirtschaft, Hochschule Heilbronn, Max-Planck-Str. 39, 74081 Heilbronn, Baden-Württemberg Germany; 2 Department of Mechanical Engineering, Universidad de La Rioja, Edificio Departamental, c/ San José de Calasanz, 31, 26004 Logroño, La Rioja Spain

**Keywords:** Quantum-classical deep learning hybrid architecture, Graphene-based sensors, Essential tremor detection, Neurological disorder diagnostics, Computational science, Medical imaging

## Abstract

This study presents a novel hardware and software architecture combining capacitive sensors, quantum-inspired algorithms, and deep learning applied to the detection of Essential Tremor. At the core of this architecture are graphene-printed capacitive sensors, which provide a cost-effective and efficient solution for tremor data acquisition. These sensors, known for their flexibility and precision, are specifically calibrated to monitor tremor movements across various fingers. A distinctive feature of this study is the incorporation of quantum-inspired computational filters—namely, *Quantvolution* and *QuantClass*—into the deep learning framework. This integration offers improved processing capabilities, facilitating a more nuanced analysis of tremor patterns. Initial findings indicate greater stability in loss variability; however, further research is necessary to confirm these effects across broader datasets and clinical environments. The approach highlights a promising application of quantum-inspired methods within healthcare diagnostics.

## Introduction

Essential tremor (ET), a prevalent neurological disorder characterized by involuntary rhythmic movements primarily in the hands and arms, presents a significant challenge in neurology^1^. Accurate detection of ET is crucial for effective diagnosis and treatment, underscoring the need for advanced yet accessible detection methods^2^. This study introduces a pioneering framework that leverages the synergy between quantum-inspired algorithms and deep learning (DL), utilizing innovative capacitive sensors developed through graphene printing techniques for ET detection^3,4^. Our approach, highlighting the integration of quantum layers within a DL architecture and the application of graphene-based capacitive sensors, represents a significant innovation aimed at enhancing diagnostic performance, measured by its accuracy, defined as the ratio of correct predictions to total predictions, and accessibility.

The core innovation of our study lies in the introduction of quantum layers, referred to as *Quantvolution*, and quantum classification algorithms, termed *QuantClass*, within our DL framework. These quantum algorithms work with a small amount of qubits and therefore are perfectly simulable in a classical machine, which greatly increases their applicability. That is why, with a slight abuse of the term, we call these solutions *quantum-inspired*. The advantages, which we discuss later, do not require quantum computers. While the general applicability of these quantum-inspired techniques across various domains remains under exploration, our hypothesis is grounded in the intuition that they exhibit heightened efficiency in scenarios characterized by sparse data sets with inherently complex data points. This observation is particularly relevant in numerous medical applications, where data scarcity coupled with the high dimensionality of available data poses a significant challenge. ET detection has been selected as a case study for this research, driven by the availability of specialized data and the potential for impactful contributions to the field.

Traditional diagnostic methods for ET rely on a combination of clinical assessments and instrumental evaluations, which, despite their effectiveness, are often hindered by high costs and limited accessibility^1,5,6^. Addressing these challenges, our research introduces a cost-effective, highly sensitive capacitive sensor enabled by the electrical properties of graphene^7–12^. This technological advancement, when integrated with our quantum-inspired DL framework, proposes a novel and accessible approach for ET detection, potentially democratizing access to precise diagnostics and personalized treatment strategies.

In summary, the innovation of our research is twofold: the development of low-cost graphene-printed capacitive sensors and the integration of quantum computational layers within a DL framework for ET detection. By focusing on ET as a case study, we not only address a significant healthcare challenge but also illustrate the broader applicability of our approach to medical diagnostics, where the nature of data and the scarcity of large datasets are prevalent.

### Advantages of our hardware and software solutions

The development of low-cost graphene-printed capacitive sensors marks a significant advancement in sensor technology^13^. These sensors leverage graphene’s exceptional electrical conductivity and sensitivity, enabling the detection of subtle electrical changes associated with tremor activity. The accessibility and affordability of the fabrication process (based on additive inkjet printing on flexible substrates) allow for broader adoption in healthcare settings, overcoming previous cost barriers associated with advanced sensor technologies^14^.

The following Table 1 provides a comparative overview of our graphene-printed capacitive sensor alongside various state-of-the-art wearable sensors for tremor detection, highlighting key attributes such as cost, sensitivity, robustness, and signal quality to contextualize the advantages and trade-offs of each technology.

**Table 1:** Comparison of graphene-printed sensor with other wearable sensors for tremor detection.

Sensor type	Cost (EUR)	Sensitivity	Robustness	Signal quality	Notes
Graphene-printed	15–20 (sensor)/ 120 (system)	High	Moderate (flexible)	High	Additive printing, low-cost, flexible, lightweight
Piezoelectric^15,16^	100–300	High	High (rigid)	Mod-High	Durable; higher cost
Resistive^17^	10–150	Moderate	Moderate	Moderate	Low-cost options; lower sensitivity
Optical^18^	50–400	Mod-High	Moderate	High	High signal quality; motion artifact sensitivity
Electromyography (EMG)^19^	50–500	High	Moderate	High	High accuracy; cost varies by system
Hybrid-flexible^20,21^	5–50	Mod-High	Moderate	Mod-High	Bimodal, ultra-low cost; wearable glove

The comparative analysis provided in Table 1 highlights the strengths and trade-offs of our graphene-printed capacitive sensor relative to other common wearable sensors used in tremor detection. The graphene-printed sensor demonstrates several unique advantages in terms of cost-efficiency, sensitivity, and flexibility, while also exhibiting certain limitations that are important for potential applications.

The primary benefit of the graphene-printed capacitive sensor lies in its low production cost, approximately 15–20 EUR, which is significantly lower than many alternative technologies, such as piezoelectric and electromyography (EMG) sensors. This cost efficiency arises not only from the relatively low price of graphene-based materials but also from the streamlined fabrication process, which leverages accessible, additive printing techniques, specifically inkjet printing on flexible substrates. Unlike traditional sensor manufacturing methods that require expensive cleanroom facilities and complex lithographic steps, our approach enables rapid, scalable, and low-waste production. This combination of material affordability and manufacturing simplicity facilitates large-scale deployment at minimal expense, making the graphene-printed sensor a highly promising candidate for widespread clinical adoption and wearable applications, where stringent cost constraints often limit technology accessibility. Importantly, the low-cost and straightforward fabrication process also makes this technology particularly suitable for deployment in resource-limited settings, where access to advanced manufacturing infrastructure and high-cost medical devices is often restricted. This expands the potential impact of the technology, supporting equitable healthcare delivery in underserved regions.

In addition to cost-effectiveness, the paper-printed graphene sensor has been engineered to capture tremor signals with fine temporal resolution, although we have not yet quantified its minimum detectable amplitude. The intrinsic electrical conductivity and mechanical compliance of graphene ink on a paper substrate are expected to facilitate detailed signal acquisition across a range of tremor intensities, contributing to high overall signal fidelity. However, rigorous benchmarking against alternative sensor technologies (e.g., resistive or optical sensors) is future work. Moreover, the flexibility of the paper substrate allows the sensor to conform to different anatomical placements, enhancing patient comfort and ease of use.

However, certain limitations accompany the graphene sensor’s flexible substrate. While this flexibility is beneficial for comfort and placement, it compromises the robustness of the sensor compared to more rigid options like piezoelectric sensors, which are better suited for environments requiring high durability. Additionally, the performance of the graphene sensor is more susceptible to environmental conditions, such as humidity and temperature variations, which can affect the stability of signal quality over prolonged use^22^.

Overall, the graphene-printed capacitive sensor offers a balanced trade-off between cost, sensitivity, and adaptability, making it an optimal choice for applications prioritizing affordability and high signal quality in essential tremor detection. These attributes position the graphene sensor as a promising tool in wearable health technology, especially for accessible and scalable deployment in clinical and personal health monitoring contexts. Future work will explore ways to enhance the sensor’s durability and environmental resilience, ensuring that it remains functional and reliable in varied conditions.

When combined with a DL architecture, these sensors enable the system to accurately identify complex tremor patterns and adapt to individual patient profiles, thereby enhancing diagnostic precision^23^. This improvement is bolstered by live feedback and visualization techniques discussed later in this paper.

The incorporation of quantum-inspired algorithms specifically enhances the system’s capability in areas such as the efficient processing of high-dimensional sensor data and the recognition of tremor patterns. By exploiting the principles of quantum computation, like unitary evolution, these algorithms improve learning efficiency, particularly in scenarios where classical algorithms face limitations due to the complexity and volume of the data. This targeted application of quantum-inspired algorithms increases the stability of the system, enabling a more accurate analysis of complex tremor patterns.

DL networks, known for their ability to identify patterns in data, complement the nuanced nature of tremor signals^24^. The synergy between quantum algorithms and DL reduces variability in the learning process^4^, an essential feature for handling limited patient datasets typical in healthcare. While the results indicate a potential benefit in stability with the quantum-inspired hybrid approach, these findings should be interpreted with caution. The quantum layers may enhance feature capture in specific cases, but extensive testing across varied datasets is essential to confirm these findings. Moreover, computational overhead currently limits the hybrid model’s scalability and efficiency, highlighting a need for further refinement before clinical applicability.

Our approach aims to democratize ET diagnostics by offering an affordable, accessible, and accurate solution. By combining graphene-printed capacitive sensors with quantum-inspired algorithms in a DL framework, we seek to enhance ET assessment and improve the quality of life for individuals with this neurological condition.

### Organization of the paper

This paper is organized as follows: “Case study” introduces the case study used to assess our hypothesis, detailing the system architecture and its integration with a quantum-inspired DL architecture. Following this, “Scope establishment” establishes the study’s scope. The investigation’s specific hardware and software components are detailed in “Hardware implementation” and “Software”, respectively. “Data collection” addresses the data collection aspects of our setup. The results of the study are summarized in “Results”, and the paper concludes with “Summary and discussion”, which provides a comprehensive discussion on various aspects of the study, outlines the main conclusions, identifies limitations, and explores potential applications of our research.

**Fig. 1:**
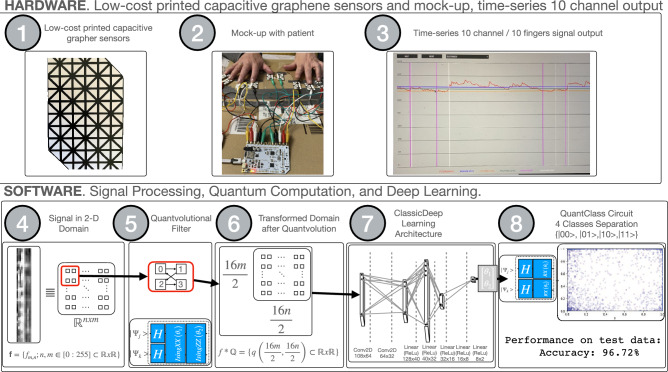
Graphical abstract of the paper.

Below in Fig. 1 is the graphical abstract of our paper, which provides a visual summary of the main concepts and methodologies employed in our research. The graphical abstract is intended to facilitate a quick understanding of the paper’s themes and structure.

In alignment with the recommendations of Eisenhardt ^25^, we follow a clear case study roadmap.

## Case study

### Scope establishment

The primary objective of this section is to establish the scope and boundaries of our research within the broader context of ET detection. ETs, characterized by rhythmic shaking, predominantly affects the hands but can also involve other body parts. Despite being common, their detection and differentiation from normal tremor patterns remain challenging due to variability in tremor characteristics and the influence of external factors like stress or caffeine^26^. The paper discuss the advances in understanding ET over the past decade. It highlights the expansion of the clinical phenotype of ET from a simple, monosymptomatic entity to a more complex condition with various tremors and other motor and non-motor features. They also emphasize that ET is likely not a single disease but a family of diseases, suggesting the use of the term “the ETs” for a more accurate representation.

The scope of our study includes:

- Development of the “Quantum-inspired DL model”: We aim to create a model that integrates quantum computing concepts with traditional DL techniques. This model is designed to process and analyze data from the graphene sensor efficiently, focusing on the detection of subtle tremor patterns that are often challenging to capture with conventional methods.
- Graphene sensor implementation and data acquisition: The design and utilization of the graphene-printed sensor are crucial. This sensor’s sensitivity to minute physiological tremors is expected to yield high-quality data, essential for the effective training and testing of our DL model. This part will elaborate on the sensor’s design parameters, its interaction with human skin, and the nuances of capturing accurate tremor data.
- Data Analysis and Pattern Recognition: A significant portion of our research is devoted to analyzing the data collected by the graphene sensor. This involves preprocessing the raw data, extracting relevant features, and employing our quantum-inspired model to identify patterns indicative of ET. The uniqueness of our approach lies in its ability to distinguish between normal physiological tremor and pathological tremor patterns, a task that has been a longstanding challenge in this field.
- Comparative Analysis and Validation: To validate our model’s efficacy, we will compare its performance with existing diagnostic tools and methods. This comparative analysis aims to demonstrate improvements in robustness measured as the standard deviation of the learning loss, in tremor detection. Additionally, we will assess the model’s applicability in real-world clinical settings, considering factors like ease of use, cost-effectiveness, and patient comfort.
- Ethical and Privacy Considerations: Given the sensitive nature of medical data, this study adheres strictly to ethical guidelines and privacy laws. All patients provided informed consent, data were properly anonymized to ensure confidentiality, and robust data security measures were implemented. The experimental protocols were formally approved by the responsible institutional licensing committee, which has requested to remain anonymous.

### Hardware implementation

The first important part of our research project is the use of a graphene-printed capacitive sensor designed for low-cost yet effective tremor detection. Graphene, celebrated for its superior electrical conductivity and exceptional mechanical properties, provides an optimal medium for the sensitive detection of physiological tremor signals. The unique electrical and mechanical properties of graphene, including its high conductivity and flexibility, underpin the design of our tremor detection sensor. The atomic thinness and robustness of graphene enhance its sensitivity to minute tremor movements, which is crucial for the objectives of our study.

Our sensor is engineered to detect tremor frequencies and amplitudes across a broad spectrum, facilitating exhaustive data capture. This section delineates the sensor’s design specifications, its fabrication process, calibration methodologies, and the integration of the sensor within a DL framework. Modifications and custom adaptations implemented to enable this integration are also discussed in detail.

#### Design and fabrication of the graphene sensor

**Fig. 2:**
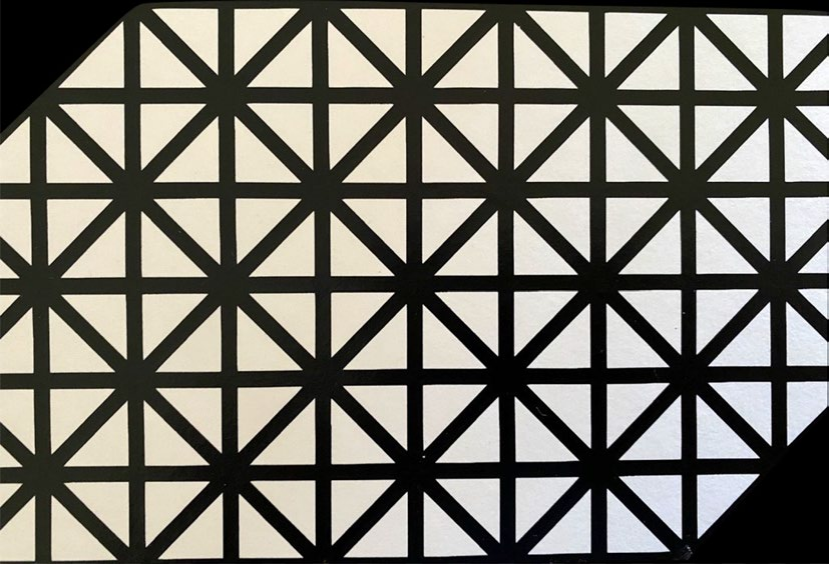
The top view of our sensor shows a 100 x 60 mm grid with 10 mm squares and a millimetre-scale bar on a white paper background. A cross-section would show 0.2 mm paper substrate and the 0.05 mm graphene-enriched ink layer, with a white “paper substrate” and a black “graphene-ink trace.

The design phase involved a rigorous selection process for the graphene material, precise determination of sensor dimensions, and layout optimization to maximize sensitivity and signal integrity. The fabrication process comprises three main stages: graphene deposition, patterning, and encapsulation. To ensure durability, biocompatibility, and consistent operational efficacy, the sensor undergoes a controlled encapsulation process that protects it from environmental degradation and mechanical stress. The sensor grid is printed using black ink infused with 50 g/kg ferrite chips using a conventional Deskjet printer. Intersection points are strategically left uncoated to facilitate connectivity at specific locations, as depicted in Figure 2.

#### Sensor calibration and testing

Post-fabrication, the sensor is subjected to an exhaustive calibration regimen to ensure its output accurately reflects the tremor movements it is designed to detect. The use of flexible paper substrates for printing the graphene sensors allows for their adaptation to diverse surfaces, enhancing the ergonomics of sensor placement on all ten fingers during data collection. This strategic sensor positioning is vital for accurately capturing tremor movements across all digits, thus providing a comprehensive tremor profile for each subject.

Calibration is paramount for validating the sensor’s performance, involving a series of tests under controlled conditions to simulate known tremor frequencies and amplitudes. This step aims to establish a reference response for the sensors against a spectrum of tremor standards. Additionally, calibration includes fine-tuning the sensor’s response to minimize interference and noise, with particular focus on eliminating environmental and electrical disturbances that could compromise data integrity.

#### Cost effectiveness of our proposal

Within the framework of this study’s exploration into an innovative hybrid architecture that integrates capacitive sensors, quantum-inspired algorithms, and deep learning for ET detection, considerable attention was given to the financial aspects of implementation. Our design process prioritized cost-effectiveness without compromising efficiency or precision. Notably, the total system cost is approximately 120 EUR, with the fabrication of each graphene sensor accounting for only 15–20 EUR of this amount. This distinction highlights that the core sensing technology is highly affordable and scalable, especially when compared to the substantial costs of alternative diagnostic or therapeutic modalities such as magnetic resonance-guided focused ultrasound thalamotomy^27^ or radiosurgery^28^, which can reach tens of thousands.

This significant difference in expenditure underscores the economic viability and scalability of our approach, particularly for large-scale screening or long-term monitoring in both well-resourced and resource-limited healthcare settings. The modular and accessible nature of our system further facilitates maintenance and sensor replacement, supporting sustainable deployment in diverse clinical environments. By leveraging the synergistic potential of advanced, flexible sensor technologies and quantum-inspired computational methodologies, our solution not only addresses cost barriers but also enhances the accessibility and equity of ET detection and management.

#### Data acquisition and processing hardware

**Fig. 3:**
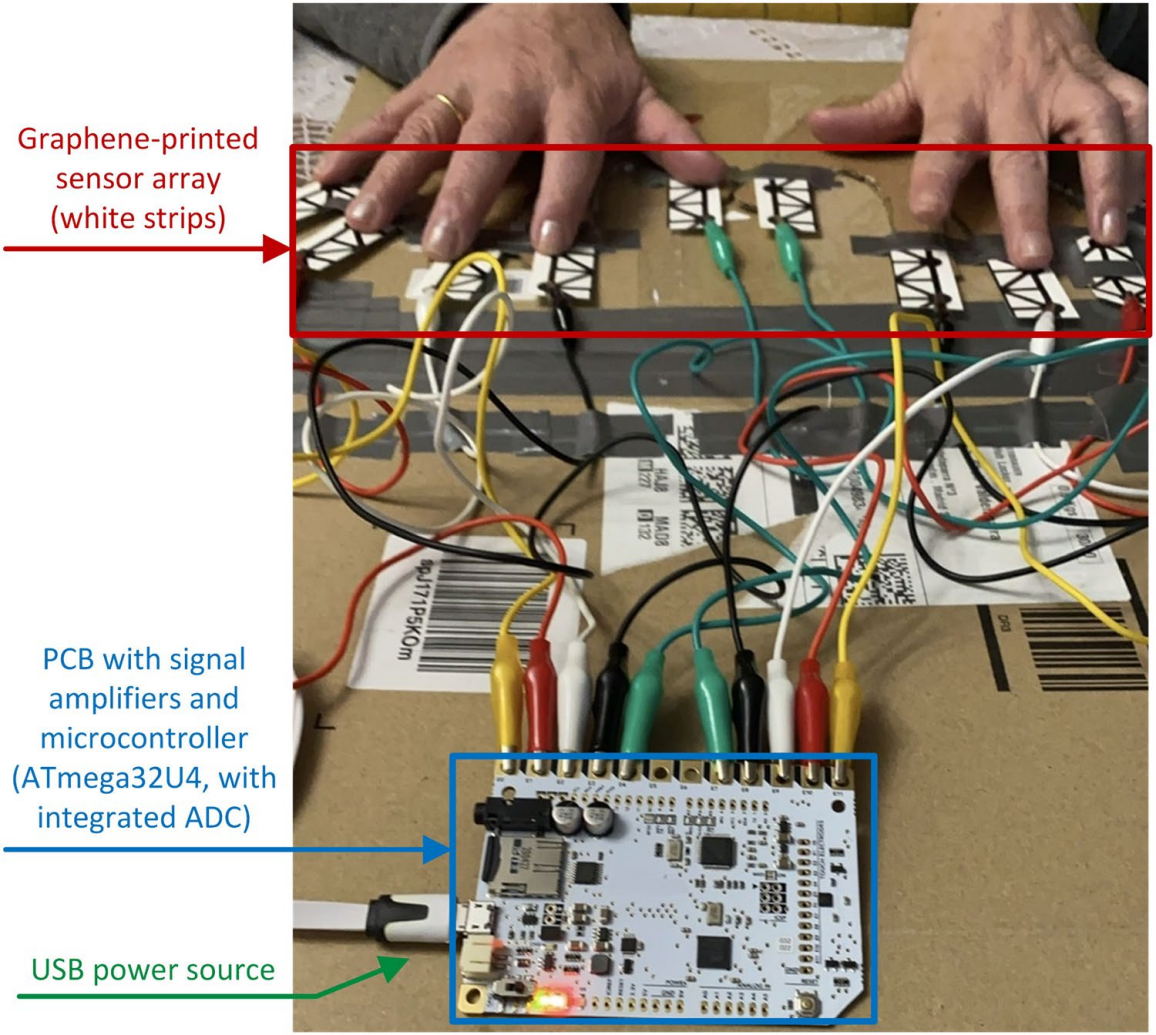
Data acquisition and processing hardware configuration. The system includes: graphene-printed sensor array (white strips), PCB with integrated signal amplifiers, ADC (via ATmega32U4 microcontroller), and USB power source.

The efficacy of our study relies on the precise data acquisition from the graphene-printed sensors and its subsequent analysis via a DL system. Our hardware configuration, illustrated in Figure 3, has been specifically designed to ensure high-fidelity capture of tremor signals and seamless data transmission for subsequent processing. At the core of this setup is the Arduino platform, featuring an ATmega32U4 microcontroller operating at 16 MHz and powered by a 5V supply. This platform was selected for its optimal balance of affordability, open-source flexibility, and the availability of multiple analog input channels—attributes essential for supporting our multi-channel graphene sensor array. By choosing widely available and well-documented components, we aimed to maximize reproducibility and scalability, while ensuring accessibility for both research and potential clinical translation.

The microcontroller plays a pivotal role in the initial stages of data handling, enabling real-time acquisition, parsing, and preprocessing of sensor signals prior to their transmission to the DL analysis system. Our hardware setup also integrates dedicated signal amplifiers and an analog-to-digital converter (ADC), which were specifically chosen and configured to achieve a high signal-to-noise ratio and sufficient bandwidth for accurately capturing tremor signals within the clinically relevant frequency range of 4–12 Hz. This configuration was informed by a trade-off analysis between cost, complexity, and performance, as well as the need for precise detection of subtle tremor features.

A key aspect of our design is the sensor data sampling rate, which is set at 128 Hz. This rate was chosen to provide sufficient temporal resolution for accurate signal capture, to facilitate effective digital filtering, and to support detailed tremor pattern analysis, in line with clinical and engineering standards for tremor monitoring.

We selected a sampling rate of 128 Hz to ensure accurate capture of essential tremor signals while maintaining a low-cost, resource-efficient acquisition system. Essential tremor typically occurs in the 4–12 Hz range^29^, so sampling above twice the highest tremor frequency, Nyquist criterion^30^, is necessary to avoid aliasing. At 128 Hz, frequencies up to 64 Hz are preserved with ample margin for higher-order harmonics or transient spikes, ensuring high fidelity of the clinically relevant band. Furthermore, an Arduino-based setup with a 128 Hz sampling rate imposes modest demands on ADC throughput, memory, and power consumption, enabling real-time preprocessing and data transmission without overtaxing the hardware. Higher sampling rates (e.g., 256 Hz) would increase CPU load and data storage requirements, contradicting our goal of a simple, low-cost platform. Therefore, 128 Hz offers the optimal trade-off between signal fidelity and hardware constraints.

**Fig. 4:**
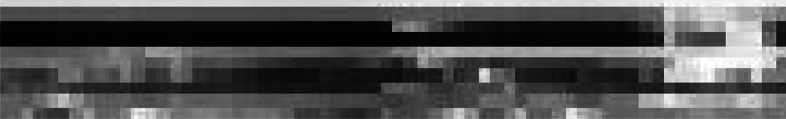
Sensor output visualization.

This hardware framework enables the generation of a structured dataset for training, testing, and validating the DL model. As illustrated in Fig. 4, each dataset entry represents a 5-second recording of tremor data, comprising a total of 640 data points ($128 \, \text {Hz} \times 5 \, \text {s}$). These data points are organized within a gray scale image format measuring 640 pixels in width by 10 pixels in height, where each row corresponds to data captured from an individual finger. The 193 images generated dataset was split into 80% training, 10% validation, and 10% test sets as follows:

- Training set: 153 images
- Validation set: 17 images
- Test set: 23 images

In this configuration, each pixel along the width dimension represents a single tremor sample over time. The resulting image offers a comprehensive and temporally coherent visualization of tremor patterns across all fingers, making it particularly well-suited for deep learning analysis. This structured data representation captures intricate temporal changes and inter-finger correlations within a compact format, facilitating the model’s ability to learn distinct tremor characteristics and identify meaningful patterns effectively.

The discussion around the selection and configuration of hardware components within our system is thorough, focusing specifically on those elements that are crucial to the innovative aspects of our architecture. We delve deeply into the rationale behind the choice of each hardware component, emphasizing those configurations that directly contribute to the system’s unique capabilities, particularly in the realm of tremor data acquisition and processing. The decision to omit details pertaining to standard hardware stems from their ubiquitous nature and well-established understanding within the field. Given that these components do not differentiate our approach from existing methodologies, we prioritize elaboration on more distinctive aspects of our system’s hardware setup. Considerable attention is dedicated to the mechanisms of data transfer within our architecture. These mechanisms are critical to the system’s performance, ensuring the integrity of tremor data and minimizing latency. Such efficiency is paramount for real-time analysis of tremor movements and seamless integration with the DL framework. By focusing on these specialized data transfer protocols, we highlight our commitment to overcoming common challenges in medical diagnostic systems, such as data corruption and delays, which can significantly impede the accuracy and responsiveness of tremor detection and analysis.

Therefore, special emphasis is placed on the data transfer mechanisms employed to maintain data integrity and minimize latency, which is critical for real-time tremor analysis and efficient processing by the DL framework.

#### Integration with the quantum-inspired deep learning system

The final part of this section describes the integration of the sensor hardware with the quantum inspired architecture DL. This process involves establishing interfaces and communication protocols to seamlessly connect the sensor array with the computational system running the DL model. Key challenges addressed during this integration include data format standardization, real-time synchronization, and preserving data integrity during transmission.

Additionally, we examine hardware considerations necessary for implementing the quantum-inspired components of the DL system. This includes the potential use of quantum simulators or quantum processing units, which are pivotal in realizing the quantum aspect of the model. The successful integration of these advanced computational units with our sensor hardware is critical to harness the full potential of the quantum inspired DL approach, especially in the nuanced realm of analysis and classification of tremor patterns.

### Software

In this section, we delve into the details of the software components crucial to our research, with a special focus on the quantum filters—*Quantvolution* and *QuantClass*. These components form the backbone of our quantum deep learning hybrid (QDLH) architecture, utilizing unitary transformations to significantly improve data processing and classification.

Convolutional neural networkss (CNNs) represent a cornerstone of DL, well-known for their effectiveness in handling visual and spatial data. At their core, CNNs automatically detect and integrate *local features* from input data, primarily images, through layers of convolutions. This process efficiently captures hierarchical patterns, enabling CNNs to stand out in tasks such as classification, object detection, and more. Each *convolution layer* in a CNN applies numerous filters to the input, producing feature maps that summarize key aspects of the data. These layers are typically followed by *pooling layers* that reduce the dimensionality and increase the field of view of deeper layers. The architecture’s ability to learn increasingly complex patterns through its depth and design choices is a primary driver of its widespread adoption and success across numerous applications.

Beyond the capabilities of traditional CNNs, the introduction of quantum-inspired technologies represents a notable advance in DL. Classical CNNs, while effective in processing large volumes of data, are limited by the boundaries of classical computation. The introduction of the *quantvolution* filter incorporates quantum mechanics to enhance CNN capabilities.

All quantum neural network components (Quantvolution and QuantClass) were executed in simulation using PennyLane’s default.qubit backend in Python (1024 shots per circuit); no physical quantum hardware was used.

Quantvolution filter The *quantvolution* filter^31,32^ involves a transformation process of the input images by applying a quantum-inspired preprocessing approach. This quantum pre-processing filter incorporates a series of quantum unitary gates, designed to map discrete signal inputs onto complex elements within a Hilbert space, enabling a richer and more structured feature extraction for subsequent DL analysis.

The *Quantvolution* filter applies unitary transformations *U*, such as Ising-like gates, to the input feature space. These transformations preserve the *inner product* of input vectors, ensuring:

1 $$\begin{aligned} \langle U \psi _i | U \psi _j \rangle = \langle \psi _i | \psi _j \rangle , \end{aligned}$$

where $\psi _i$ and $\psi _j$ represent the input states in the feature space. This preservation is a fundamental property of unitary operations, guaranteeing that the geometric relationships between data points (e.g., distances and angles in the vector space) remain unchanged during the transformation.

In contrast, standard CNNs filters are linear transformations represented by convolution operations, often followed by nonlinear activations. A standard convolution operation is defined as:

2 $$\begin{aligned} h_{ij} = \sum _{k,l} w_{kl} \cdot x_{i+k,j+l}, \end{aligned}$$

where $h_{ij}$ is the output feature map, $x_{i+k,j+l}$ represents the local input patch, and $w_{kl}$ are the weights of the filter. While this operation effectively extracts spatially localized features, it does not inherently preserve geometric relationships like the inner product. Instead, the convolution operation can introduce distortions such as changes in the relative magnitudes and directions of feature vectors.

These distortions arise due to the following reasons:

- Filter non-orthogonality: convolutional filters in CNNs are not constrained to be orthogonal. As a result, the dot product $\langle x, y \rangle$ between two feature vectors *x* and *y* can change after the convolution operation, altering the relative geometry of data points.
- Nonlinear activation distortion: nonlinear activation functions (e.g., ReLU, sigmoid) applied after convolution can introduce further distortions by truncating or compressing certain dimensions of the feature space.
- Gradient flow and optimization artifacts: during training, the filters are optimized for specific tasks, which may lead to overfitting to local data distributions and loss of general geometric properties.

This geometric distortion can hinder feature discrimination in tasks requiring precise relationships between input data points, especially when dealing with high-dimensional spaces or datasets with subtle inter-class variations.

In contrast, the *Quantvolution* filter, through its unitary transformations, ensures:

- Inner product preservation: as shown, the transformations preserve the inner product, maintaining the original geometric relationships between the input vectors.
- Exploitation of interference effects: the unitary transformations leverage quantum interference-like effects, where the output depends on coherent superpositions of the input features. This enhances the ability to separate data points in the feature space based on subtle variations.
- Feature robustness: by preserving the geometry of the input space, the quantvolution filter is less prone to overfitting and maintains better generalization properties.

The ability of quantvolution filters to maintain geometric integrity and exploit interference effects positions them as superior to standard CNNs filters in tasks requiring high precision and robustness in feature extraction.

We delve into the mechanics of unitary quantum transformation of discrete signals, specifically focusing on a two-dimensional domain pertinent to image processing in ET diagnostics. This transformation, while applicable to higher-dimensional domains, would require exponentially more computational resources, thus we constrain our analysis to a 2D space for efficiency.

In recent studies, quantum circuits have been employed for visual information pre-processing, with each pixel of the convolution window being assigned a qubit. These qubits undergo various rotations, and the average measurement on the Z-basis axis is projected. This method has shown promising results in image recognition tasks with standard datasets like MNIST ^31,33^. Our approach, however, introduces a novel concept where we treat the pixels of the convolution window as a network. In this network, the edges are weighted, allowing for the adjustment of their relative importance. Here, instead of mapping each pixel to a qubit, we assign a qubit to each edge of the pixel network.

Consider an image represented by a matrix of pixel intensities, ${\bf{f}}$, where each element $f_{m,n}$ corresponds to the intensity at position $(m,n)$ within a discrete two-dimensional grid, such that $m,n \in [0,255]$, encompassing the range of standard 8-bit grayscale values. This matrix ${\bf{f}}$ can be interpreted as a discrete signal defined over a two-dimensional spatial domain.

To analyze this image using graph-based convolutional methods, we introduce a convolutional graph, ${\bf{G}}$, consisting of node pairs $\{(0,1), (1,2), (2,3), (3,0)\}$. Each pair represents an edge connecting nodes in a structured manner, forming a $2 \times 2$ graph layout. This graph serves as the foundation for applying convolutional operations, facilitating the analysis of spatial relationships and patterns within ${\bf{f}}$.

For quantum-enhanced analysis, we map each edge of ${\bf{G}}$ to a distinct qubit, denoted by $q_i$, where $i \in \{0,1,2,3\}$.

**Fig. 5:**
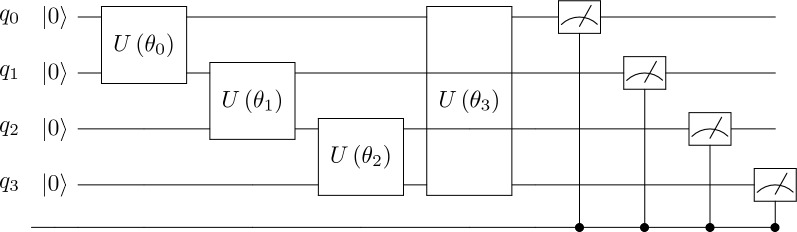
Quantum convolution filter circuit (quantvolution).

The proposed unitary transformation ${\bf{Q}}$, as described by Fig. 5, is a composite of two types of quantum unitary gates. Initially, we apply Hadamard gates to all qubits, a standard equalizing step in many quantum circuit designs^34^. Following this, we apply a parametrized Ising coupling gate that rotates signals in the $X$ and $Z$ spatial directions, ensuring the spatial coherence of the transformed data. This method results in a probability distribution for each possible classical output of the circuit, with the parametrized circuit inputting the pixel values of the domain and outputting a newly convoluted image along the two-dimensional isotropic space ${\textbf {f}}$. The gray-scaled pixel values are normalized between 0 and 1, and this value represents the cosine of the $\theta$ initialization of the quantum gates.

To represent the matrix multiplication involved in the given quantum circuit for four qubits, we consider the operations applied: Hadamard gates, IsingXX gates, and IsingZZ gates. Let’s denote the qubits as $q_0$, $q_1$, $q_2$, and $q_3$.

- Hadamard gate (H): applied to each of the qubits $j$ and $k$ for every edge in $G$. The Hadamard gate is represented by the matrix $$H = \frac{1}{\sqrt{2}}\begin{pmatrix}1 & 1\\ 1 & -1\end{pmatrix}$$. It creates a uniform superposition of the $|0\rangle$ and $|1\rangle$ states when applied to the state $|0\rangle$.
- IsingXX gate: with an angle $\theta = \pi \theta [j]$, it can be represented as $XX(\theta ) = \exp \left( -i \frac{\theta }{2} (X \otimes X)\right)$, where $$X = \begin{pmatrix}0 & 1\\ 1 & 0\end{pmatrix}$$.
- IsingZZ gate: similarly, with an angle $\theta = \pi \theta [j]$, the IsingZZ gate is $ZZ(\theta ) = \exp \left( -i \frac{\theta }{2} (Z \otimes Z)\right)$, with $$Z = \begin{pmatrix}1 & 0\\ 0 & -1\end{pmatrix}$$.

Given these operations, and denoting the indices of target qubits as the subscript of each gate, the sequence applied to qubits $j$ and $k$ for each edge $E_{jk}$ in the circuit is represented as:

$$\begin{aligned} U_{\text {quantvol}}(\theta [j])&= H_j H_k \cdot XX(\pi \theta [j])_{jk} \cdot ZZ(\pi \theta [j])_{jk} \\&= \left( \frac{1}{\sqrt{2}}\begin{pmatrix}1 & 1\\ 1 & -1\end{pmatrix}\right) _j \left( \frac{1}{\sqrt{2}}\begin{pmatrix}1 & 1\\ 1 & -1\end{pmatrix}\right) _k \\&\quad \cdot \exp \left( -i \frac{\pi \theta [j]}{2} X_j \otimes X_k\right) \cdot \exp \left( -i \frac{\pi \theta [j]}{2} Z_j \otimes Z_k\right) . \end{aligned}$$

This formula represents the Hadamard gates’ application to qubits $j$ and $k$, followed by the IsingXX and IsingZZ gates. The overall unitary operation $U_{\text {Quantvol}}$ for the full circuit would be the product of such matrices for all edges $E$, applied to the qubits’ initial state. This is the unitary that is applied four times in Fig. 5, where the notation has been slightly simplified for clarity.

*Quantum inspired deep learning architecture*

**Fig. 6:**
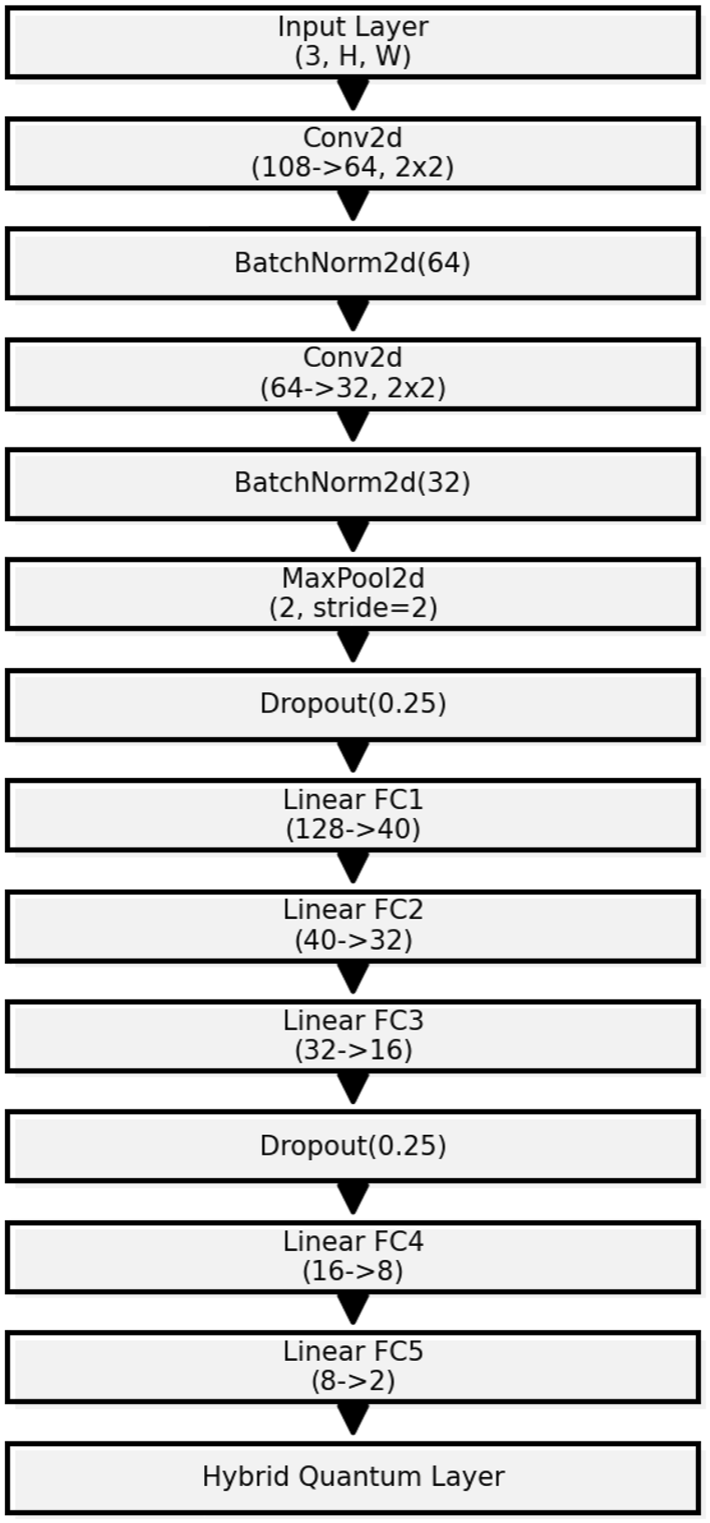
Enhanced visualization of the quantum-classic DL network architecture.

The DL architecture employed in this study is constructed using a CNN framework, which is particularly designed for processing data with Euclidean topology such as images. The architecture, consists of sequential layers aiming to extract and learn hierarchical features from the input data. Initially, the network employs two convolutional layers (conv1 and conv2), each followed by batch normalization (conv1_bn1 and conv2_bn2) and max pooling to reduce spatial dimensions while retaining important features. Dropout layers are inserted to prevent overfitting by randomly omitting subsets of features during training. The network transitions from convolutional layers to fully connected linear layers (fc1 to fc4), culminating in a hybrid quantum-classical layer (hybrid) that performs the *QuantClass* filter. This design facilitates a deep understanding of both classical and quantum data representations, enabling the network to perform complex tasks with high efficiency. The learning rate is dynamically adjusted using a polynomial decay strategy to optimize training. Figure 6 illustrates the detailed structure of this network, providing a visual representation of its comprehensive and intricate design.

In this study, cross-entropy loss is used as a measure of prediction error across training iterations. The primary objective in deep learning training is to minimize this loss, which corresponds to reducing the model’s prediction error. Cross-entropy loss is defined as:

$$\begin{aligned} L = -\frac{1}{N} \sum _{i=1}^{N} \sum _{c=1}^{C} y_{i,c} \log (p_{i,c}), \end{aligned}$$

where $N$ is the total number of samples, $C$ represents the classes, $y_{i,c}$ denotes the binary indicator (0 or 1) if class label $c$ is the correct classification for sample $i$, and $p_{i,c}$ is the model’s predicted probability for class $c$ for sample $i$.

For the implementation of this architecture we used the *PennyLane* quantum machine learning library integrated into *PyTorch*.

This design can be summarized as follows:

- The first part (not included in the actual definition of the class) consists in preprocessing the data with the *Quantvolution* filter described above.
- The network then comprises multiple convolutional layers with batch normalization and dropout for regularization, followed by fully connected layers and a hybrid quantum-classical layer for processing.
- The training procedure involves adjusting the learning rate dynamically and employing a cross-entropy loss function. The model’s parameters are optimized using the Adam optimizer.

This proposed approach leverages the unique capabilities of quantum circuits to process information in a way that classical computing alone cannot, thus potentially enhancing the performance of machine learning models on certain tasks. The detailed explanation of the code is as follows:

- Forward Method. The “forward” method computes the output of the hybrid quantum-classical function for a given input. It takes as inputs an input tensor, a quantum circuit, and a shift parameter for gradient estimation. First, the method executes the quantum circuit using the input parameters to obtain a probability distribution. To enhance the model’s ability to explore the solution space, a small random shift is introduced into the input parameters. The gradient of the quantum circuit with respect to its input is then estimated using the parameter shift rule, which involves running the circuit with inputs shifted by $\pi /2$ and $-\pi /2$. The resulting probabilities are converted back into tensors, ensuring compatibility with the neural network framework. These tensors are stored along with the input tensor for use in the backward pass.
- Backward Method. The ‘backward‘ method computes the gradients of the loss function with respect to the input parameters of the quantum circuit. It applies the parameter shift rule to compute these gradients, using the probabilities computed during the forward pass with shifted inputs. The gradient is computed as the difference between the probabilities obtained with positive and negative shifts, divided by two. This gradient is then multiplied by the gradient of the loss function to the hybrid function’s output (provided by the autograd mechanism) to obtain the final gradients that are propagated back through the network.
- Hybrid Class. The ‘Hybrid‘ class defines a neural network module that incorporates the hybrid quantum-classical function as a layer within a larger neural network architecture. The constructor initializes the quantum circuit with the specified backend and number of shots (measurements), along with a shift parameter that influences the gradient estimation. The ‘forward‘ method of this class simply calls the ‘apply‘ method of the ‘HybridFunction‘, passing along the input tensor with the initialized quantum circuit and shift parameter.

Optimizing the parameters $\theta [j]$ in a quantum circuit using gradient descent requires computing the gradient of a cost function with respect to each parameter. In quantum computing, this is often achieved through the parameter-shift rule^35^, a widely used technique for estimating gradients in parameterized quantum gates. Here, we outline the application of gradient descent to the quantum circuit operations described previously, along with the mathematical expressions for computing gradients with respect to $\theta [j]$.

Gradient descent is an iterative optimization algorithm used to minimize a cost function $C(\theta )$ by adjusting the parameters in the direction of the steepest descent, determined by the negative gradient:

$$\begin{aligned} \theta [j]_{\text {new}} = \theta [j] - \eta \frac{\partial C}{\partial \theta [j]} \end{aligned}$$

where $\eta$ is the learning rate.

In a quantum circuit with parameterized gates (e.g., IsingXX and IsingZZ gates dependent on $\theta [j]$), the goal is to determine the set of parameters that minimizes the expectation value of a measurement, which serves as the cost function.

The parameter-shift rule^35^ is a gradient estimation technique that leverages circuit evaluations at shifted parameter values, ensuring an efficient estimation of gradients without requiring explicit differentiation within the quantum circuit. For a quantum gate with a parameter $\theta [j]$, the gradient with respect to $\theta [j]$ can be computed using the difference of the expectation values of the circuit with the parameter shifted by $\pm s$, where $s$ is typically $\pi /2$. For a gate $G(\theta [j])$ parameterized by $\theta [j]$, the gradient of the expectation value $C(\theta )$ of a measurement operator $M$ with respect to $\theta [j]$ is:

$$\begin{aligned} \frac{\partial C}{\partial \theta [j]} = \frac{C(\theta [j] + s) - C(\theta [j] - s)}{2\sin (s)} \end{aligned}$$

where $C(\theta [j] \pm s)$ denotes the cost function evaluated with the parameter $\theta [j]$ shifted by $\pm s$.

For the IsingXX and IsingZZ gates in the circuit, applying the parameter-shift rule yields:

$$\begin{aligned} \frac{\partial C}{\partial \theta [j]} = \frac{C_{XX,ZZ}(\theta [j] + \frac{\pi }{2}) - C_{XX,ZZ}(\theta [j] - \frac{\pi }{2})}{2} \end{aligned}$$

assuming $$s = \pi /2$$.

Given the composite quantum operation $U_{\text {Quantvol}}$ involving parameterized IsingXX and IsingZZ gates, the derivative of the expectation value of an observable $O$ with respect to the parameter $\theta [j]$ can be computed using the parameter-shift rule as follows:

$$\begin{aligned} \frac{\partial \langle O \rangle }{\partial \theta [j]} = \frac{\langle O \rangle _{\theta [j] + \pi /2} - \langle O \rangle _{\theta [j] - \pi /2}}{2} \end{aligned}$$

In this expression:

- $\langle O \rangle _{\theta [j] + \pi /2}$ denotes the expectation value of $O$ after applying $U_{\text {Quantvol}}$, where the parameter $\theta [j]$ in each IsingXX and IsingZZ gate is shifted by $+\pi /2$.
- $\langle O \rangle _{\theta [j] - \pi /2}$ denotes the expectation value of $O$ after applying $U_{\text {Quantvol}}$, where the parameter $\theta [j]$ in each IsingXX and IsingZZ gate is shifted by $-\pi /2$.

This calculation enables the gradient-based optimization of parameters within quantum circuits by providing a method to estimate the gradient of the expectation value of observables with respect to the parameters governing the behavior of quantum gates.

*QuantClass* We introduce the *QuantClass* filter, the final addition to our quantum DL architecture. Positioned at the final stage of the classical DL pipeline, *QuantClass* serves as a generalization of the conventional decision layer found in DL models. This filter, through its integration of quantum computational principles, redefines the decision-making process, particularly vital in complex binary classification tasks prevalent in medical diagnostics.

Integrating the novel *QuantClass* filter with deep learning (DL) architectures requires understanding the role of activation functions in the final layer of traditional DL models. These functions are crucial for tailoring the model’s output to specific tasks, such as classification or regression^36^. In binary classification, the sigmoid function is used to convert inputs into a probability score, indicating the likelihood of belonging to one of two classes. For tasks requiring multi-class classification, the *softmax* function assigns a probability distribution across all classes, ensuring the sum of probabilities equals one. This makes it suitable for categorizing instances into multiple classes. In contrast, regression tasks, which aim to predict continuous quantities, might not use an activation function in the last layer or might use a linear one, allowing the model to output a wide range of values. The choice of activation function affects the model’s training efficiency and performance. While Rectified Linear Unit (ReLU) and its variants help alleviate the vanishing gradient problem and are popular in various layers, their use in the final layer depends on the specific requirements of the task at hand^37^.

**Fig. 7:**
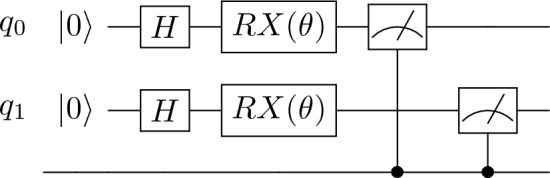
Quantum classification filter circuit (*QuantClass*).

The primary innovation of *QuantClass* lies in its ability to transform the traditional linear decision-making process of DL into a multi-dimensional quantum computational space. This transformation is executed through a unitary transformation, detailed in Fig. 7. At the outset, a Hadamard gate is applied, which functions to distribute probability amplitudes uniformly across computational basis states, thereby ensuring unbiased processing in subsequent stages.

After the initial application of a Hadamard gate to evenly distribute probability, the system applies a parametrized rotation around the *X*-axis. This rotation is directly influenced by the output of the preceding classical DL layers, allowing the system to adapt dynamically to the specific features of the input data. The parameterized nature of this rotation enables fine-tuning during the processing stage, enhancing the model’s flexibility. Finally, the quantum state is measured, introducing an irreversible step that collapses the state into a definite basis. This measurement outcome directly determines the classification of the input, assigning it to one of four possible categories. Unlike classical bits, which can only represent binary states (0 or 1), two qubits can encode and process information beyond simple binary classification, allowing for a richer and more efficient categorization using quantum computation principles.

Given a quantum circuit with Hadamard gates followed by RX gates parameterized by $\theta$, we aim to express the overall unitary operation $U_{Qclass}(\theta )$ as a function of $\theta$. This operation is applied to a two-qubit system initially in the state $|00\rangle$. When applied to both qubits in a two-qubit system, we use the tensor product of two Hadamard gates $H \otimes H$, resulting in:

$$\begin{aligned} H \otimes H = \frac{1}{2} \begin{pmatrix} 1 & 1 & 1 & 1 \\ 1 & -1 & 1 & -1 \\ 1 & 1 & -1 & -1 \\ 1 & -1 & -1 & 1 \end{pmatrix}. \end{aligned}$$

The rotation around the X-axis by an angle $\theta$ for a single qubit is represented by:

$$\begin{aligned} RX(\theta ) = \begin{pmatrix} \cos \left( \frac{\theta }{2}\right) & -i\sin \left( \frac{\theta }{2}\right) \\ -i\sin \left( \frac{\theta }{2}\right) & \cos \left( \frac{\theta }{2}\right) \end{pmatrix} \end{aligned}$$

Applying this gate to both qubits involves the tensor product $RX(\theta ) \otimes RX(\theta )$, conceptually expanding to:

$$\begin{aligned} \begin{pmatrix} \cos ^2\left( \frac{\theta }{2}\right) & -i\cos \left( \frac{\theta }{2}\right) \sin \left( \frac{\theta }{2}\right) & -i\cos \left( \frac{\theta }{2}\right) \sin \left( \frac{\theta }{2}\right) & -\sin ^2\left( \frac{\theta }{2}\right) \\ -i\cos \left( \frac{\theta }{2}\right) \sin \left( \frac{\theta }{2}\right) & \cos ^2\left( \frac{\theta }{2}\right) & -\sin ^2\left( \frac{\theta }{2}\right) & -i\cos \left( \frac{\theta }{2}\right) \sin \left( \frac{\theta }{2}\right) \\ -i\cos \left( \frac{\theta }{2}\right) \sin \left( \frac{\theta }{2}\right) & -\sin ^2\left( \frac{\theta }{2}\right) & \cos ^2\left( \frac{\theta }{2}\right) & -i\cos \left( \frac{\theta }{2}\right) \sin \left( \frac{\theta }{2}\right) \\ -\sin ^2\left( \frac{\theta }{2}\right) & -i\cos \left( \frac{\theta }{2}\right) \sin \left( \frac{\theta }{2}\right) & -i\cos \left( \frac{\theta }{2}\right) \sin \left( \frac{\theta }{2}\right) & \cos ^2\left( \frac{\theta }{2}\right) \end{pmatrix} \end{aligned}$$

The overall operation $U_{Qclass}(\theta )$ is the product of these matrices, representing the sequential application of the Hadamard and $RX(\theta )$ gates to a two-qubit system:

$$\begin{aligned} U_{Qclass}(\theta ) = (H \otimes H) \cdot (RX(\theta ) \otimes RX(\theta )) \end{aligned}$$

This matrix $U_{Qclass}(\theta )$ encapsulates the transformation of the quantum state through these operations, preparing the qubits in superposition states due to the Hadamard gates and then rotating them around the X-axis by an angle $\theta$ due to the $RX(\theta )$ gates.

This hybrid quantum-classical model offers a new paradigm for enhancing computational capabilities and potentially unlocking new applications. Furthermore, the adaptability of the *QuantClass* filter to accommodate a broader spectrum of classes is not only a theoretical advantage but a practical solution to the limitations faced by classical DL architectures. Through the addition of more qubits, the *QuantClass* filter significantly enhances the model’s capability to handle complex, multi-class classification problems. This scalability is inherent to quantum computing principles, where the addition of each qubit doubles the system’s computational space, thereby creating an exponentially large space and allowing for the encoding and processing of a much richer set of possibilities.

**Fig. 8:**
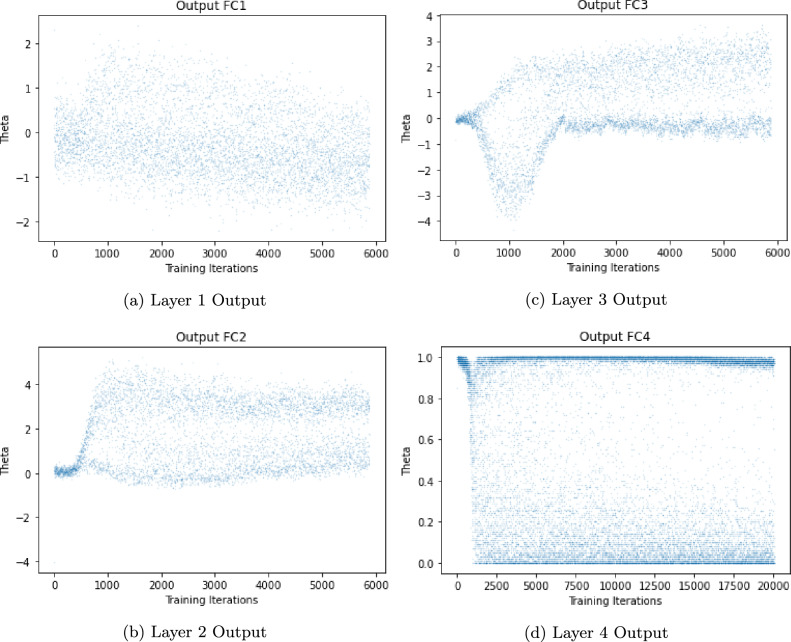
Sequential visualization of the hybrid neural network layer outputs, illustrating the progressive feature extraction and transformation process.

The input to this quantum filter, the parameter $\theta$, is derived from the classical DL architecture, bridging the gap between classical and quantum computing paradigms. Figure 8 delineates the evolution of this parameter across a learning process encompassing 6000 iterations and 32 images, illustrating the dynamic adaptation of the *QuantClass* filter in response to the learning process. The output measurement from the *QuantClass* filter, representing the culmination of this quantum-classical interplay, provides the classification of the ET patients’ behavior.

In a standard deep-learning model, the final layer typically consists of a fully connected linear transformation followed by an activation such as softmax, which maps the pre-activation logits $z \in \mathbb {R}^C$ into a probability vector

$$\begin{aligned} p_c = \frac{\exp (z_c)}{\sum _{k=1}^{C} \exp (z_k)},\quad c=1,\dots ,C, \end{aligned}$$

and the class prediction is $\arg \max _{c} p_c$. This approach effectively embeds the decision boundary in $\mathbb {R}^C$, but it remains constrained by the linear separation in that space and by the specific form of the softmax activation.

By contrast, *QuantClass* replaces the linear + softmax stage with a two-qubit unitary circuit $U_{Qclass}(\theta )$. After the preceding dense layer produces a two-dimensional input $(x_1,x_2)$, we encode $\theta = f(x_1,x_2)$ (e.g., a simple affine map) and apply

$$\begin{aligned} U_{Qclass}(\theta ) \;=\; (H \otimes H)\,\bigl (RX(\theta )\otimes RX(\theta )\bigr ), \end{aligned}$$

where $H$ is the Hadamard gate and $RX(\theta )$ is a rotation about the $X$-axis by $\theta$. Measurement of the two qubits yields four outcome probabilities $\{p_{00},p_{01},p_{10},p_{11}\}$, one for each computational basis state $\{|00\rangle ,|01\rangle ,|10\rangle ,|11\rangle \}$. Unlike softmax, *QuantClass* can exploit interference between amplitudes in a 4-dimensional Hilbert space, potentially allowing more complex decision boundaries. In effect:

$$\begin{aligned} p_{ij} \;=\; \bigl |\langle ij\,|\,U_{Qclass}(\theta )\,|\,00\rangle \bigr |^2,\quad i,j\in \{0,1\}, \end{aligned}$$

and the predicted class is $\arg \max _{i,j} p_{ij}$. Although both softmax and *QuantClass* output a probability distribution over $C$ classes, *QuantClass* leverages a unitary transformation that preserves inner products and can implement non-linear separations via quantum interference. In practice, this results in (a) potentially richer feature maps when data is sparse and (b) different loss-landscape properties during training, as observed in our ablation study. As a result, *QuantClass* generalizes the “linear + softmax” paradigm by embedding the decision step into a higher-dimensional, unitary-preserving transformation, rather than a purely linear mapping in $\mathbb {R}^C$.

### Data collection

Our data collection strategy was designed to capture comprehensive tremor data from participants diagnosed with ET. We employed a mixed-methods approach, combining quantitative measurements from the state-of-the-art sensors proposed here with qualitative data provided by medical professionals, both of them extracted from the participants.

### Classification of tremor data

In our study, we analyzed tremor data collected from 40 Caucasian females aged between 50 and 65 years. The participants were evenly distributed across four distinct classes, with 10 individuals in each category. This demographic was specifically chosen to provide insights into the prevalence and characteristics of ET patterns within this population. All subjects gave written informed consent before participation. All experimental procedures were conducted according to the policies and ethical principles of the Declaration of Helsinki. All experimental protocols were approved by the institutional ethics committee of a Klinik in Heilbronn, Germany. All data were properly anonymized to ensure the privacy of the subjects. Our classification scheme was instrumental in discerning different levels of tremor severity and control status, enabling a comprehensive analysis and understanding of ET patterns. The following details each of these classes:

1. Healthy individuals: comprising data from our control group, these individuals exhibited no pathological tremor patterns. Representing the baseline of our study, they provide essential comparative data to distinguish ET symptoms from normal physiological tremor levels.
2. Mild ET: This category included participants with minimal tremor impact. While their tremors were noticeable, they did not significantly interfere with daily activities. Understanding this class is crucial for recognizing the early stages of ET and developing preventive interventions against its progression.
3. Moderate ET: Participants in this class demonstrated more pronounced tremor symptoms, impacting their performance in various activities and tasks. This group’s study aids in understanding the daily life effects of ET and the effectiveness of early intervention strategies.
4. Severe ET: Including participants with severe tremors that continuously impacted their quality of life and ability to perform daily tasks. These cases often required comprehensive management strategies, emphasizing the importance of advanced treatment approaches in severe ET.

This classification framework is fundamental to our study, enabling us to develop a deeper understanding of ET, its progression, and the effectiveness of various treatment modalities. By differentiating these categories, our research contributes significantly to neurology and patient care, providing new insights into the management and understanding of ET.

### Data analysis and processing

In this study, we embarked on a comprehensive process of data collection, followed by preprocessing and classification using a quantum DL architecture, to enhance our understanding and detection of ET patterns. This multifaceted approach began with the systematic gathering of data, ensuring a robust dataset that was both diverse and representative of the varied manifestations of ETs.

ETs is one of the most common movement disorders. Its key feature is a tremor in both hands and arms during action without other neurological signs. It also may affect a person’s head, voice, or lower limbs. ETs is usually diagnosed based on family history and examination; thus, laboratory and imaging studies are usually not required. No biologic markers exist for essential tremor. Electromyography or accelerometry can be used to assess tremor frequency, rhythmicity, and amplitude but is not part of the routine evaluation. For confidentiality reasons, the specific German hospital or doctor in which this classification was performed cannot be named. However, it is important to note that the labelling was conducted by experienced medical professionals specialized in movement disorders. The data was pre-labelled: patients had been already been labelled at four different levels following the standard way to categorize tremors in European laboratories^38^. The labelling process adhered strictly to the standard diagnostic criteria used across European neurology departments, ensuring that each patient’s data reflects accurate clinical observations.

Once collected, the data underwent a series of preprocessing steps, designed to enhance signal quality and extract features of paramount importance. These preprocessing stages were carefully tailored to address specific challenges inherent in the data. Firstly, noise reduction techniques were employed to eliminate extraneous signals and background interference, thereby improving the clarity and accuracy of the data. This was followed by normalization, a critical step in standardizing the data to a common scale, which is essential for comparative analysis and ensuring consistency across the dataset. Finally, segmentation was carried out, which involved dividing the continuous data stream into meaningful and manageable segments, thereby facilitating more efficient and focused analysis.

The first step of our preprocessing involved the transformation of signals from ten sensors, one for each finger, into grayscale images. Each sensor’s signal was converted into a pixel intensity value, resulting in a 10-pixel wide image representation. This transformation was achieved by first calibrating each sensor to ensure uniformity in signal intensity ranges. The calibrated signals were then mapped onto a grayscale spectrum, where the intensity of the tremor at each sensor corresponded to a specific shade of gray. This method allowed us to encapsulate the multidimensional nature of the tremor data into a two-dimensional image format, facilitating more efficient processing by the DL architecture.

With the data thus refined, we leveraged a state-of-the-art quantum DL architecture. This advanced computational model was specifically chosen for its potential to handle the complexities of the data and its ability to learn and adapt from large datasets. The model was employed to classify the preprocessed data into predetermined categories. This classification process was pivotal, serving as a litmus test for the model’s capability to discern between varying intensities of tremors and, crucially, to differentiate between patients with ET and healthy control subjects. The success of this classification stage was indicative of the model’s sophistication and its potential as a diagnostic tool.

The methodology employed in the data collection and classification phases is a cornerstone of our research. It provides the foundational data critical for a thorough evaluation of our proposed quantum DL model. By executing this rigorous and methodically structured process, our objective is to showcase the model’s efficacy in accurately detecting and categorizing ETs patterns. This, we believe, could mark a significant advancement in the field, offering a novel and potentially more effective means of diagnosing and understanding ETs, thus contributing to improved patient outcomes and advancing our knowledge in this critical area of medical science.

### Results

In this section, we illustrate the *QuantClass* filter’s processing capabilities within a hybrid DL architecture, emphasizing its theoretical and practical implications. We present a comparative analysis of the loss distribution between a classic DL architecture and our proposed hybrid model, which incorporates principles of quantum computing.

Figure 8 offers a visual representation of the hybrid neural network layer outputs, revealing the transformation process across the network.

This figure comprises four panels, each depicting the output from one of the network’s fully connected layers, thereby elucidating the role of each layer in feature extraction and learning. The panels, through a dataset of 6000 iterations and 32 images within the trainset (80% of the dataset), demonstrate the network’s evolving internal representations, thus providing insights into the learning mechanisms.

**Fig. 9:**
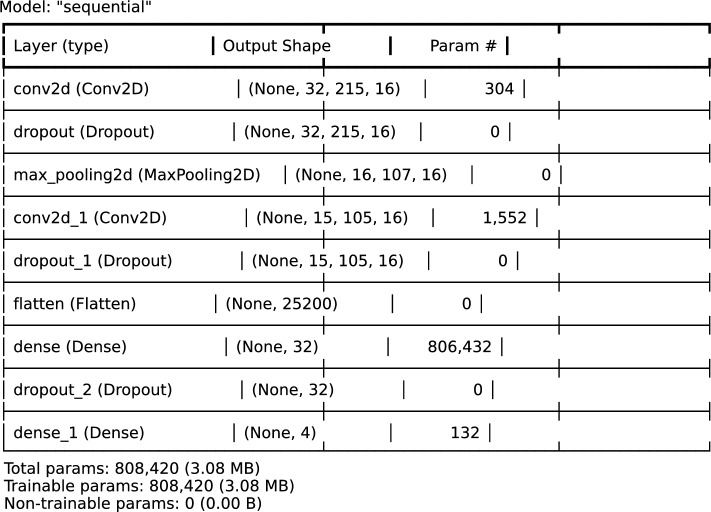
Best classic deep learning model architecture.

The model’s classical baseline architecture and hyperparameters were optimized using the Hyperband ^39^ method, which performed an exhaustive search across configurations. The Hyperband tuner optimizes model configurations by using validation accuracy as the objective, testing each model for up to 15 epochs, narrowing trials by a factor of 3, and averaging results across 3 executions per trial for reliable performance metrics. The classic deep learning model performs image classification on a dataset of four classes, with data preprocessing steps that include resizing images to $32 \times 215$ pixels, normalization, and augmentation through label noise. The best-performing architecture, as illustrated in Fig. 9, consists of two convolutional layers, a max-pooling layer, and multiple dense layers designed for classification accuracy and computational efficiency. The model begins with a 16-filter convolutional layer of size $3 \times 3$, producing an output shape of $32 \times 215 \times 16$, followed by a dropout layer to reduce overfitting. A max-pooling layer with a pool size of $2 \times 2$ is applied next, reducing the spatial dimensions to $16 \times 107 \times 16$. A second convolutional layer with 16 filters and a kernel size of $3 \times 3$ is added, followed by another dropout layer. The number of feature extraction layers is identical as in the hybrid architecture. After feature extraction, the model flattens the output to a 1D layer with 25,200 units, preparing the data for dense layers in the inference phase. The dense section of the architecture contains a fully connected layer with 32 units, followed by a 50% dropout layer, and a final dense layer with 4 units (one for each class), using softmax activation for classification. The model was evaluated for robustness through 10-fold cross-validation, with the dataset split into 80% training, 10% validation, and 10% test data. Performance metrics were logged for thorough evaluation.

The parameter search process tunes the neural networks’s hyperparameters to find the best configuration for the task at hand. Keras Tuner’s Hyperband algorithm was used due to its efficient exploration of the hyperparameter space, balancing thoroughness with computational efficiency. Hyperband quickly narrows down promising configurations by allocating more resources (e.g., training epochs) to high-performing models in initial trials, allowing early elimination of less effective configurations. The primary hyperparameters tuned included the number of filters in each convolutional layer (ranging from 32 to 128), kernel size (mostly $3 \times 3$ but also $5 \times 5$ in some trials), dropout rates (10–-30% for convolutional layers and up to 50% for dense layers), and the number of units in dense layers. Learning rates are consistent across models. To validate the chosen hyperparameters’ robustness, a 10-fold cross-validation was performed, dividing the data into ten parts and iteratively training on nine parts while testing on the remaining one. This provided reliable metrics on model performance, with 10% of the data set aside for final testing, helping to confirm that the selected configuration would generalize well to unseen data.

#### Ablation study

This section provides a detailed and professional analysis of the ablation study, which was conducted to assess the impact of incorporating quantum-inspired components into our deep learning framework. The study compared multiple configurations that combine a *QuantClass* layer with a *Quantvolution* layer at different resolutions, and contrasted these with a classical baseline (No *QuantClass* + No Filter). All models were evaluated using a consistent cross-validation protocol, and performance metrics were recorded for training, validation, and testing phases alongside the total computation time. All configurations were trained for a fixed 100 epochs; the only quantitative time measure we recorded is the total wall-clock time. We did not track per-epoch convergence thresholds, gradient norms, FLOPs, memory usage, or inference latency. In this few-data regime, fixing epochs ensured a fair comparison, and the total training times already reflect each model’s relative computational cost. Since all models ran without exhausting our hardware resources, more granular metrics were not critical for this study.

The performance of each configuration is summarized in a detailed Analysis of Configurations:

- **Test** #0 (No ***QuantClass*** + No Filter – Classical Baseline):
  - Train Loss: $0.1310 \pm 0.2971$, Train Accuracy: $96.23\% \pm 11.32\%$
  - Validation Loss: $0.0942 \pm 0.2797$, Validation Accuracy: $98.00\% \pm 11.65\%$
  - Test Loss: $0.0892 \pm 0.2763$, Test Accuracy: $98.22\% \pm 10.62\%$
  - Total Computation Time: 65.76 s
  The classical baseline attains the highest test accuracy (98.22%) but exhibits substantial variability in both validation and test losses (std = 0.2797 and 0.2763, respectively). This indicates sensitivity to data splits and a higher overfitting risk. Its total training time of 65.76 seconds provides a benchmark for comparing quantum-enhanced variants.
- **Test** #1 (***QuantClass*** + ***Quantvolution*** 2$\times$2):
  - Train Loss: $0.4027 \pm 0.0979$, Train Accuracy: $93.19\% \pm 11.27\%$
  - Validation Loss: $0.4192 \pm 0.0965$, Validation Accuracy: $96.73\% \pm 12.33\%$
  - Test Loss: $0.4075 \pm 0.0922$, Test Accuracy: $96.72\% \pm 11.44\%$
  - Total Computation Time: 21.02 s
  Integrating a 2$\times$2 Quantvolution filter with QuantClass yields slightly lower test accuracy (96.72%) but substantially reduces loss variability (validation-loss std = 0.0965, test-loss std = 0.0922). This stable convergence occurs in just 21.02 seconds, demonstrating that the 2$\times$2 Quantvolution layer effectively supports feature extraction without excessive computational overhead.
- **Test** #2 (***QuantClass*** + ***Quantvolution*** 4$\times$4):
  - Train Loss: $0.7175 \pm 0.0927$, Train Accuracy: $54.65\% \pm 11.17\%$
  - Validation Loss: $0.7454 \pm 0.0620$, Validation Accuracy: $52.05\% \pm 9.46\%$
  - Test Loss: $0.7127 \pm 0.1271$, Test Accuracy: $55.62\% \pm 15.89\%$
  - Total Computation Time: 88.26 s
  Increasing Quantvolution resolution to 4$\times$4 degrades performance drastically: test accuracy falls to 55.62% with high loss (test-loss std = 0.1271). Although training completes in 88.26 seconds, the excessive filter complexity introduces noise and impairs generalization.
- **Test** #3 (***QuantClass*** + No ***Quantvolution***):
  - Train Loss: $0.3960 \pm 0.1323$, Train Accuracy: $93.66\% \pm 14.00\%$
  - Validation Loss: $0.3731 \pm 0.1472$, Validation Accuracy: $94.94\% \pm 15.27\%$
  - Test Loss: $0.3656 \pm 0.1337$, Test Accuracy: $95.48\% \pm 13.67\%$
  - Total Computation Time: 7.59 s
  Omitting Quantvolution but retaining QuantClass yields 95.48% test accuracy with moderate variability (test-loss std = 0.1337). It also requires only 7.59 seconds, indicating that QuantClass alone captures key features efficiently, although a 2$\times$2 Quantvolution filter (Test #1) offers better stability per unit time.
- **Test** #4 (No ***QuantClass*** + ***Quantvolution*** 2$\times$2):
  - Train Loss: $1.2719 \pm 0.1111$, Train Accuracy: $36.95\% \pm 7.54\%$
  - Validation Loss: $1.3985 \pm 0.0278$, Validation Accuracy: $30.77\% \pm 5.18\%$
  - Test Loss: $1.4083 \pm 0.0210$, Test Accuracy: $22.03\% \pm 3.74\%$
  - Total Computation Time: 4.45 s
  Without QuantClass, even a 2$\times$2 Quantvolution filter cannot extract meaningful features: test accuracy collapses to 22.03% and losses remain high. The very low computation time (4.45 seconds) does not compensate for this drastic accuracy loss.
- **Test** #5 (No ***QuantClass*** + ***Quantvolution*** 4$\times$4):
  - Train Loss: $0.0692 \pm 0.2347$, Train Accuracy: $97.72\% \pm 9.87\%$
  - Validation Loss: $3.4513 \pm 0.7994$, Validation Accuracy: $20.82\% \pm 5.42\%$
  - Test Loss: $3.5621 \pm 0.8763$, Test Accuracy: $20.72\% \pm 4.46\%$
  - Total Computation Time: 40.73 s
  Removing QuantClass while using a 4$\times$4 Quantvolution filter leads to severe overfitting: although training accuracy is 97.72%, validation and test accuracies drop below 21%, with extremely high losses. The 40.73-second runtime underscores that neither speed nor high training accuracy compensates for the lack of generalization.

**Fig. 10:**
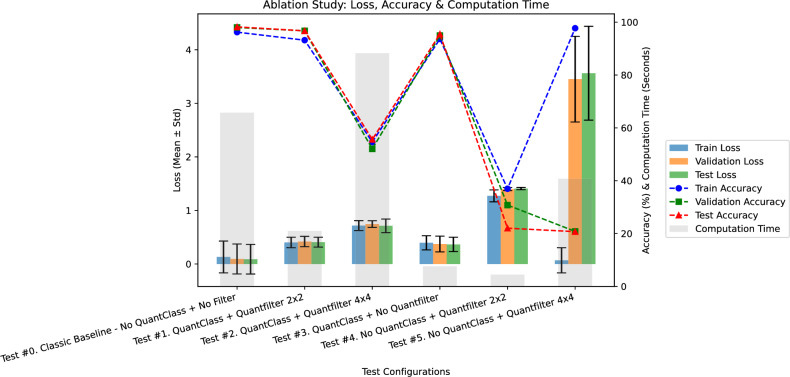
Ablation study.

Figure 10 provides a visualization of the ablation study. This figure reinforces the quantitative observations by highlighting the stable performance of the Test #1 configuration relative to the alternatives. Table 2 provides a consolidated overview of the performance metrics and computation times for each test configuration discussed in this study.

**Table 2:** Summary of test configurations and performance metrics.

Test	Train loss	Train acc.	Val loss	Val acc.	Test loss	Test acc.	Time (s)
Test #0:	$0.1310\pm 0.2971$	$96.23\%\pm 11.32\%$	$0.0942\pm 0.2797$	$98.00\%\pm 11.65\%$	$0.0892\pm 0.2763$	$98.22\%\pm 10.62\%$	65.76
Test #1:	$0.4027\pm 0.0979$	$93.19\%\pm 11.27\%$	$0.4192\pm 0.0965$	$96.73\%\pm 12.33\%$	$0.4075\pm 0.0922$	$96.72\%\pm 11.44\%$	21.02
Test #2:	$0.7175\pm 0.0927$	$54.65\%\pm 11.17\%$	$0.7454\pm 0.0620$	$52.05\%\pm 9.46\%$	$0.7127\pm 0.1271$	$55.62\%\pm 15.89\%$	88.26
Test #3:	$0.3960\pm 0.1323$	$93.66\%\pm 14.00\%$	$0.3731\pm 0.1472$	$94.94\%\pm 15.27\%$	$0.3656\pm 0.1337$	$95.48\%\pm 13.67\%$	7.59
Test #4:	$1.2719\pm 0.1111$	$36.95\%\pm 7.54\%$	$1.3985\pm 0.0278$	$30.77\%\pm 5.18\%$	$1.4083\pm 0.0210$	$22.03\%\pm 3.74\%$	4.45
Test #5:	$0.0692\pm 0.2347$	$97.72\%\pm 9.87\%$	$3.4513\pm 0.7994$	$20.82\%\pm 5.42\%$	$3.5621\pm 0.8763$	$20.72\%\pm 4.46\%$	40.73

In particular, comparing **Test #0** and **Test #1** illustrates the trade-off between raw accuracy and stability:

- *Test #0:*
  - Test Accuracy: 98.22 %
  - Validation Loss: $0.0942 \pm 0.2797$ (Std = 0.2797)
  - Test Loss: $0.0892 \pm 0.2763$ (Std = 0.2763)
  Although Test #0 achieves the highest test accuracy, its large standard deviation in validation and test losses indicates considerable variability across folds. In a few-data healthcare setting, this variability can translate into unreliable probability estimates—depending on the random split, the model’s confidence scores may fluctuate widely, making it difficult for clinicians to trust any single risk threshold.
- *Test #1:*
  - Test Accuracy: 96.72 %
  - Validation Loss: $0.4192 \pm 0.0965$ (Std = 0.0965)
  - Test Loss: $0.4075 \pm 0.0922$ (Std = 0.0922)
  By contrast, Test #1 incurs only a modest drop in test accuracy ($\approx$ 1.5 percentage points) but reduces the standard deviation of validation loss by roughly a factor of three. This tighter loss distribution signals far more consistent performance across different cross-validation folds. In practice, that consistency means clinicians can adjust the decision threshold (e.g., to emphasize sensitivity or specificity) without large swings in false-positive or false-negative rates.

The results of the ANOVA shown in Table 3 reveal that *there are statistically significant differences* between the models. The *p-value = 0* confirms that at least one model performs significantly differently from the others. However, ANOVA does not indicate which models differ from each other; therefore, we use Tukey’s Honest Significant Difference^40^ (Tukey HSD) test shown in Table 4 for specific pairwise comparisons.

**Table 3:** ANOVA table.

Source of variation	Sum of squares	df	F-statistic	p-value
**C(test)**	**693272.01**	5	**1149.15**	**0**
Residual	71670.83	594	NaN	NaN

Significant values are in bold.

**Table 4:** Summary of tukey HSD results.

Model 1	Model 2	Mean difference	p-value	Confidence interval	Significant?
Test #1	Test #2	$-41.10$	**0.000**	($-45.54, -36.66$)	Yes
Test #1	Test #3	$-1.25$	0.9671	($-5.69, 3.19$)	No
Test #1	Test #4	$-74.69$	**0.000**	($-79.13, -70.25$)	Yes
Test #1	Test #5	$-75.99$	**0.000**	($-80.44, -71.56$)	Yes
Test #1	Test #0	1.49	0.9299	($-2.95, 5.93$)	No
Test #2	Test #3	39.86	**0.000**	(35.42, 44.30)	Yes
Test #2	Test #4	$-33.59$	**0.000**	($-38.03, -29.14$)	Yes
Test #2	Test #5	$-34.90$	**0.000**	($-39.34, -30.45$)	Yes
Test #2	Test #0	42.60	**0.000**	(38.16, 47.04)	Yes
Test #3	Test #4	$-73.44$	**0.000**	($-77.88, -69.00$)	Yes
Test #3	Test #5	$-74.75$	**0.000**	($-79.19, -70.31$)	Yes
Test #3	Test #0	2.74	0.4903	($-1.70, 7.18$)	No
Test #4	Test #5	$-1.31$	0.9591	($-5.75, 3.13$)	No
Test #4	Test #0	76.18	**0.000**	(71.74, 80.62)	Yes
Test #5	Test #0	77.49	**0.000**	(73.05, 81.93)	Yes

Significant values are in bold.

Models that are not significantly different from each other (p > 0.05) include:

- Test #1 vs. Test #3
- Test #1 vs. Test #0
- Test #3 vs. Test #0
- Test #4 vs. Test #5

Models with significant differences include:

- Test #2 performs worse than Test #1, Test #3, and Test #0.
- Test #4 and Test #5 perform worse than Test #0.
- Test #0 is the best in accuracy, being significantly better than the rest.

The model Test #1 provides the best balance between stability, accuracy, and computational efficiency. If the goal is purely the highest accuracy regardless of stability, then Test #0 is the best option. Test #5 suffers from severe overfitting and have the worst overall performance. From a quantum deep learning perspective, the results indicate the following:

1. Critical role of quantum classification: the quantum classification layer is indispensable for achieving robust performance. Its absence results in a substantial decline in accuracy and generalization, as evidenced by Tests #4 and #5.
2. Optimal quantvolution resolution: a moderate *Quantvolution* resolution (2x2) optimally complements the quantum classification layer. It achieves high accuracy with low loss variability while offering a favorable trade-off between computational efficiency and performance stability.
3. Classical baseline considerations: although the classical model attains slightly higher absolute test accuracy, its increased loss variability suggests that the quantum-enhanced configurations may offer more reliable performance under scarce data conditions.

In summary, these results highlight how stability (loss variability) and raw accuracy trade off in few-data healthcare contexts:

- Accuracy vs. stability trade-off: Table 2 shows that **Test #0** achieves the highest test accuracy (98.22%) but also exhibits a large standard deviation in validation loss (Std = 0.2797). In contrast, **Test #1** attains slightly lower accuracy (96.72%) but reduces validation-loss variability to Std = 0.0965. Whenever Quantvolution is present, the standard deviation of both validation and test losses is markedly smaller than in the corresponding classical configurations.
- Implications for few-data clinical settings: In healthcare applications with small patient cohorts, reliable probability estimates and consistent convergence across cross-validation folds are often more valuable than marginal gains in raw accuracy. A large loss standard deviation implies that, depending on the random data split, the model’s output probabilities (and thus its sensitivity/specificity at a chosen threshold) can fluctuate significantly. Table 2 confirms that Quantvolution enhances robustness: configurations incorporating a 2$\times$2 Quantvolution (**Tests #1** and #3) have lower loss variation, making them less prone to overfitting and more likely to generalize predictably on unseen patients.
- Overall recommendation: while pure accuracy metrics favor **Test #0**, stability-oriented metrics highlight that Quantvolution-enabled architectures (**Tests #1** and #3) are better suited to contexts where limited labels make overfitting especially risky.

The ablation study provides evidence that an optimally configured quantum deep learning model, specifically, one incorporating a quantum classification layer paired with a moderately resolved 2x2 *Quantvolution*, achieves a balanced performance with high accuracy and robust generalization. While the classical baseline model performs well in terms of absolute accuracy, the quantum-enhanced configurations offer improved stability and reduced sensitivity to data variations. These results highlight the potential of quantum-inspired architectures in advanced deep learning applications and pave the way for further exploration into the fine-tuning of quantum components to maximize their benefits.

### Summary and discussion

This study introduces an approach in ET detection, merging graphene-printed capacitive sensors with quantum-inspired DL. This integration has shown considerable promise in differentiating ET tremors from normal tremors, potentially impacting the field of low-cost diagnostics.

A key innovation in our approach is the quantum filter employed as a generalization of activation functions in classical DL models. This novel application allows for the enhancement of the neural network’s ability to process complex tremor data efficiently. Significantly, this aspect opens the possibility for specialized chip design optimizations, potentially reducing the cost and complexity of hardware implementations. Such advancements could lead to more accessible and cost-effective diagnostic tools in clinical settings.

The *QuantClass* filter, by integrating quantum computing with DL, enhances classical multi-class classification methods. This hybrid approach facilitates a refined classification schema through strategic quantum state manipulations, beginning with a Hadamard gate and proceeding with an X-axis rotation based on the output from preceding layers. Such precision in quantum state calibration permits the differentiation of four distinct classes, represented by orthogonal quantum states $|00\rangle$, $|01\rangle$, $|10\rangle$, and $|11\rangle$, thereby expanding the classification potential beyond conventional methods.

The empirical results from model testing highlight the hybrid model’s effective data categorization. Tensors with values near 1 or 0 denote high confidence in the model’s classifications, while the variation in these values portrays a probabilistic assessment of class memberships, enabling operation within a multi-dimensional computational space. The shift from binary to probabilistic outputs mirrors the *QuantClass* filter’s adaptive learning, which is responsive to the nuances of the input data. This adaptability is crucial for the model’s application to real-world datasets, which often present complex and non-linear relationships.

The hybrid model’s ability to maintain a tighter distribution of loss values throughout training epochs suggests that the learning process is not only more stable but potentially more generalizable to unseen data.

Discrepancies between the predicted tensors and the target values indicate areas where the model may benefit from further refinement. These discrepancies offer valuable insights into the model’s learning dynamics and can guide future improvements in architecture and training protocols.

The performance metrics underscore the hybrid model’s capabilities, with a mean cross-entropy loss that reflects its robustness against overfitting, as evidenced by the low standard deviation. This robustness, combined with an accuracy of 96.72%, showcases the effectiveness of the *QuantClass* filter in enhancing DL models with quantum principles, making it a promising approach for advanced classification tasks in complex datasets.

However, the potential of the *QuantClass* filter be understood as a generalization of the decision layer in classical deep learning leveraging the potential of using $N$ qubits to classify $2^N$ classes. By leveraging the quantum mechanical properties of superposition, where qubits can represent multiple states simultaneously, and entanglement, which allows for correlations between qubits, the *QuantClass* filter has the capability to handle multi-class classification with potentially enhanced depth and granularity over classical methods. This quantum-inspired approach enables the processing of information in a multi-dimensional computational space, which could offer more nuanced insights into data features. Such capabilities are promising, especially in fields like medical diagnostics, where distinctions between different conditions can be subtle and complex.

While the ablation study demonstrates that quantum-inspired architectures hold promise, the new results also reveal several important limitations. In our experiments, the optimal configuration—incorporating a quantum classification layer with a 2x2 quantum filter—achieved a test accuracy of 96.72%, which is competitive yet slightly lower than the classical baseline’s 98.22% accuracy. Furthermore, although the optimal quantum-enhanced model exhibited low variability in loss (with a standard deviation comparable to other quantum configurations), its mean test loss of 0.4075 remains substantially higher than the classical model’s mean loss of 0.0892. These findings suggest that, while quantum-inspired layers contribute to enhanced performance consistency, their overall accuracy improvement is modest and appears to be highly sensitive to the specific configuration and filter resolution employed.

In addition, the computational overhead associated with the quantum components is a notable concern. The integration of the quantum classification and filtering layers leads to increased training times and higher resource usage relative to the classical approach. For instance, the optimal quantum configuration required 65.76 seconds per run compared to 40.73 seconds for the classical baseline. Conversely, configurations with a higher-resolution (4x4) quantum filter or those omitting the quantum classification layer entirely resulted in drastically reduced performance—even though they sometimes achieved shorter computation times. This trade-off underscores that while quantum-inspired architectures may offer improved loss stability, the benefits are offset by increased computational demands, which could limit their applicability in scenarios where efficiency and rapid deployment are critical.

Furthermore, despite promising results, the study has limitations that constrain the applicability of the current study’s findings:

1. Limited training and testing protocol
  Single dataset source: The model was trained and tested on data from a single dataset source. This could limit its ability to generalize to varied clinical settings, patient demographics, or alternative sensor setups, potentially affecting its robustness in real-world applications.
2. Data and model constraints
  Small sample size for demographic variability: the dataset may not fully capture the demographic and clinical diversity needed to generalize across patient populations. For instance, variations in tremor patterns across different age groups, ethnicities, and severity levels could affect the model’s generalizability and should be accounted for in future studies.
  Lack of real-world validation for dataset quality: while the dataset captures essential tremor patterns under controlled conditions, real-world variability—such as differences in sensor placement, environmental noise, and natural patient movement—could pose additional challenges that this study’s dataset may not fully encompass.
3. Model complexity and interpretability
  Limited model interpretability: quantum-inspired models are inherently less interpretable than classical models. In medical diagnostics, interpretability is crucial for clinician trust and validation. Future research could explore methods to elucidate the quantum component’s contributions to model decisions.
  Potential overfitting to specific data characteristics: due to the model’s complexity and the dataset constraints, there is a risk of overfitting to specific data features, which may reduce generalizability. Addressing overfitting by expanding the dataset or by optimizing the model architecture could enhance the model’s robustness.

These limitations underscore that the study offers an initial exploration rather than definitive evidence of the model’s clinical utility.

While the current study demonstrates the feasibility of low-cost, graphene-printed capacitive sensors integrated with an Arduino platform for ET detection, several practical considerations must be addressed to enable real-world deployment in wearable or mobile contexts. First, wireless data transmission is a essential for untethered operation in daily life. Although our prototype currently relies on a wired connection to ensure data integrity during initial validation, the Arduino is compatible with widely available Bluetooth and WiFi modules. Future iterations will integrate Bluetooth Low Energy (BLE) or similar modules to enable secure, real-time transmission of sensor data to mobile devices or cloud-based platforms, supporting continuous remote monitoring and analysis. The selection of wireless technology must be balanced against power consumption, data rate requirements, and the intended use scenario. Second, power consumption is a key factor for wearable applications. The base system (Arduino with the direct sensor readout) consumes approximately 0.23 W. adding a Bluetooth module such as the HC-05/HC-06 increases total energy consumption to approximately 0.35–0.43 W, while the use of WiFi modules (e.g., ESP8266/ESP32) raises consumption substantially to $\sim$0.9–1.25 W. This difference has direct implications for battery sizing and operational autonomy: Bluetooth-based solutions are notably more suitable for long-term, battery-powered wearables, potentially offering 12–14 hours of operation on a typical 1000 mAh battery, whereas WiFi-based approaches may be more appropriate for short-term monitoring or scenarios with external power sources. Third, regarding real-time inference feasibility, our current system achieves a sampling rate of 128 Hz, which is adequate for capturing the frequency spectrum of pathological tremors. While the Arduino platform can handle basic preprocessing and feature extraction, more computationally demanding DL inference is best offloaded to a paired smartphone or edge device. This hybrid approach balances the need for low-latency feedback with the computational requirements of advanced algorithms, and preserves user privacy by enabling on-device processing. In summary, while our current setup demonstrates core technical feasibility, future work will focus on optimizing wireless communication (favoring low-power Bluetooth for extended battery life), minimizing power consumption through hardware and firmware strategies, and refining real-time data processing to realize a fully wearable, affordable, and user-friendly solution for tremor monitoring in diverse healthcare settings.

Future research should also focus on expanding the participant base to ensure a more diverse and representative dataset. In particular, recruiting individuals spanning a wider range of ages, genders, and ethnic backgrounds will be essential for rigorously assessing the generalizability and fairness of our methodology. Inclusion of participants from multiple clinical sites and varied demographic groups will allow us to capture a broader spectrum of physiological and behavioral variability, which is essential for developing models that perform robustly in real-world, heterogeneous populations. Such diversity will also enable comprehensive subgroup analyses and support the identification and mitigation of potential algorithmic biases. Collectively, these efforts will strengthen the clinical relevance, equity, and reliability of our approach, paving the way for its responsible and effective deployment in diverse healthcare environments.

Additionally, future work should focus on facilitating the seamless integration of quantum-inspired DL models for practical use in healthcare settings, particularly by leveraging the potential for specialized chip design to reduce costs and enhance usability for healthcare professionals.

A key consideration underlying our design choices was to prioritize a low-cost, easily deployable hardware platform that demonstrates the quantum-inspired algorithm under realistic, few-data conditions. Consequently, the paper-printed graphene sensor and Arduino-based data acquisition were selected as a proof-of-concept rather than a fully optimized sensing solution. In future work, we intend to conduct controlled sensor characterizations–comparing sensitivity, noise floor, and dynamic range against established alternatives—so that the impact of hardware improvements can be systematically evaluated alongside algorithmic refinements. This staged approach ensures that our quantum-inspired deep-learning framework remains validated in resource-constrained settings before investing in higher-precision (and higher-cost) sensor technologies.

## Conclusions and further steps

In conclusion, this study offers an exploratory step toward enhancing ET detection and analysis through the integration of graphene-printed capacitive sensors with a quantum-inspired DL model. The approach presented here provides a novel framework for ET diagnostics, demonstrating a hybrid model that incorporates both classical and quantum-inspired components. While the model showed promise in stability over traditional DL models, its overall effectiveness and efficiency in practical applications require further validation.

The findings suggest that, with continued development, this hybrid approach could contribute to cost-effective ET diagnostics, especially if the technology was adapted to specialized chip design that could optimize DL processes and lower computational costs. However, it is important to acknowledge that the current study was limited by factors such as computational overhead and the need for additional optimization, which could affect scalability and real-world applicability.

The results also encourage continued investigation into quantum-inspired principles for medical diagnostics, where the potential for nuanced pattern recognition could benefit the detection of other neurological disorders. Future studies should explore the scalability of this technology, its adaptability to various medical conditions, and its viability in personalized medicine, where tailored diagnostic and treatment approaches are crucial. Additionally, research into hardware optimization, such as specialized chip design, could reduce computational demands, enabling broader accessibility and practical implementation.

These advancements, though incremental, indicate new possibilities for enhancing patient care across medical disciplines. By refining the hybrid model and addressing existing limitations, future research may uncover pathways for more efficient and accessible diagnostic tools, potentially broadening the impact of quantum-inspired deep learning in healthcare applications.

## Supplementary Information

Supplementary Information 1.

Supplementary Information 2.

Supplementary Information 3.

Supplementary Information 4.

## Data Availability

The datasets generated and/or analyzed during the current study have been uploaded with the manuscript submission. The authors want to recognize that this research has been partially supported by the Ministerio de Ciencia e Innovación of Spain (Grant Ref. PID2022-137748OB-C31 funded by MCIN/AEI/10.13039/501100011033) and “ERDF A way of making Europe”.
