# Supplementary material for: Quantum-classical deep learning hybrid architecture with graphene-printed low-cost capacitive sensor for essential tremor detection
Source: Sci Rep. 2025 Jun 20;15:20204. doi: 10.1038/s41598-025-06359-1 (PMC12181389; doi:10.1038/s41598-025-06359-1)

Ablation Study - Quantum-Classical Deep Learning Hybrid Architecture with Graphene-Printed Low-Cost Capacitive Sensor for Essential Tremor Detection

March 7, 2025

1 Ablation of concept. "Quantum-Classical Deep Learning Hybrid Architecture with Graphene-Printed Low-Cost Capacitive Sensor for Essential Tremor Detection"

1.1 Summary of results

[19]:
```
import numpy as np
import matplotlib.pyplot as plt

# Define the ablation study results
tests = [
    "Test #0. Classic Baseline - No QuantClass + No Filter",
    "Test #1. QuantClass + Quantfilter 2x2",
    "Test #2. QuantClass + Quantfilter 4x4",
    "Test #3. QuantClass + No Quantfilter",
    "Test #4. No QuantClass + Quantfilter 2x2",
    "Test #5. No QuantClass + Quantfilter 4x4",
]

# Loss (Mean, Std)
train_loss = [(0.1310, 0.2971),
    (0.4027, 0.0979), (0.7175, 0.0927), (0.3960, 0.1323),
    (1.2719, 0.1111), (0.0692, 0.2347)
]
val_loss = [(0.0942, 0.2797),
    (0.4192, 0.0965), (0.7454, 0.0620), (0.3731, 0.1472),
    (1.3985, 0.0278), (3.4513, 0.7994)
]
test_loss = [(0.0892, 0.2763),
    (0.4075, 0.0922), (0.7127, 0.1271), (0.3656, 0.1337),
    (1.4083, 0.0210), (3.5621, 0.8763)
]
```

```python
# Accuracy (Mean, Std)
train_acc = [(96.23, 11.32),
    (93.19, 11.27), (54.65, 11.17), (93.66, 14.00),
    (36.95, 7.54), (97.72, 9.87)
]
val_acc = [(98.00, 11.65),
    (96.73, 12.33), (52.05, 9.46), (94.94, 15.27),
    (30.77, 5.18), (20.82, 5.42)
]
test_acc = [(98.22, 10.62),
    (96.72, 11.44), (55.62, 15.89), (95.48, 13.67),
    (22.03, 3.74), (20.72, 4.46)
]

# Computation Time
computation_time = [65.76, 21.02, 88.26, 7.59, 4.45, 40.73]

# Print results to verify
for i, test in enumerate(tests):
    print(f"\n## {test}")
    print(f"Train Loss: Mean = {train_loss[i][0]:.4f}, Std = {train_loss[i][1]:.
↪4f}")
    print(f"Train Accuracy: Mean = {train_acc[i][0]:.2f}%, Std =␣
↪{train_acc[i][1]:.2f}%")
    print(f"Validation Loss: Mean = {val_loss[i][0]:.4f}, Std = {val_loss[i][1]:
↪.4f}")
    print(f"Validation Accuracy: Mean = {val_acc[i][0]:.2f}%, Std =␣
↪{val_acc[i][1]:.2f}%")
    print(f"Test Loss: Mean = {test_loss[i][0]:.4f}, Std = {test_loss[i][1]:.
↪4f}")
    print(f"Test Accuracy: Mean = {test_acc[i][0]:.2f}%, Std = {test_acc[i][1]:.
↪2f}%")
    print(f"Total Computation Time: {computation_time[i]:.2f} seconds")


# Plot all metrics in one figure
fig, ax1 = plt.subplots(figsize=(10, 6))

x = np.arange(len(tests))  # Position for bars
width = 0.2  # Bar width

# Loss bars
ax1.bar(x - width, [y[0] for y in train_loss], width, yerr=[y[1] for y in␣
↪train_loss], label="Train Loss", capsize=5, alpha=0.7)
ax1.bar(x, [y[0] for y in val_loss], width, yerr=[y[1] for y in val_loss],␣
↪label="Validation Loss", capsize=5, alpha=0.7)
```

```python
ax1.bar(x + width, [y[0] for y in test_loss], width, yerr=[y[1] for y in␣
    ↪test_loss], label="Test Loss", capsize=5, alpha=0.7)

ax1.set_ylabel("Loss (Mean ± Std)")
ax1.set_xlabel("Test Configurations")
ax1.set_xticks(x)
ax1.set_xticklabels(tests, rotation=15, ha="right")

# Create secondary y-axis for accuracy and computation time
ax2 = ax1.twinx()

# Accuracy lines
ax2.plot(x, [y[0] for y in train_acc], marker="o", linestyle="--", label="Train␣
    ↪Accuracy", color="blue")
ax2.plot(x, [y[0] for y in val_acc], marker="s", linestyle="--",␣
    ↪label="Validation Accuracy", color="green")
ax2.plot(x, [y[0] for y in test_acc], marker="^", linestyle="--", label="Test␣
    ↪Accuracy", color="red")

# Computation time as a bar overlay
ax2.bar(x, computation_time, width=0.5, alpha=0.2, label="Computation Time",␣
    ↪color="gray")

ax2.set_ylabel("Accuracy (%) & Computation Time (Seconds)")

# Move legend outside the plot to the right
fig.legend(loc="center left", bbox_to_anchor=(1, 0.5))

plt.title("Ablation Study: Loss, Accuracy & Computation Time")
plt.tight_layout()
plt.savefig("Ablation Study.pdf", format="pdf", bbox_inches="tight")

plt.show()
```

```
## Test #0. Classic Baseline - No QuantClass + No Filter
Train Loss: Mean = 0.1310, Std = 0.2971
Train Accuracy: Mean = 96.23%, Std = 11.32%
Validation Loss: Mean = 0.0942, Std = 0.2797
Validation Accuracy: Mean = 98.00%, Std = 11.65%
Test Loss: Mean = 0.0892, Std = 0.2763
Test Accuracy: Mean = 98.22%, Std = 10.62%
Total Computation Time: 65.76 seconds

## Test #1. QuantClass + Quantfilter 2x2
Train Loss: Mean = 0.4027, Std = 0.0979
Train Accuracy: Mean = 93.19%, Std = 11.27%
```

```
Validation Loss: Mean = 0.4192, Std = 0.0965
Validation Accuracy: Mean = 96.73%, Std = 12.33%
Test Loss: Mean = 0.4075, Std = 0.0922
Test Accuracy: Mean = 96.72%, Std = 11.44%
Total Computation Time: 21.02 seconds

## Test #2. QuantClass + Quantfilter 4x4
Train Loss: Mean = 0.7175, Std = 0.0927
Train Accuracy: Mean = 54.65%, Std = 11.17%
Validation Loss: Mean = 0.7454, Std = 0.0620
Validation Accuracy: Mean = 52.05%, Std = 9.46%
Test Loss: Mean = 0.7127, Std = 0.1271
Test Accuracy: Mean = 55.62%, Std = 15.89%
Total Computation Time: 88.26 seconds

## Test #3. QuantClass + No Quantfilter
Train Loss: Mean = 0.3960, Std = 0.1323
Train Accuracy: Mean = 93.66%, Std = 14.00%
Validation Loss: Mean = 0.3731, Std = 0.1472
Validation Accuracy: Mean = 94.94%, Std = 15.27%
Test Loss: Mean = 0.3656, Std = 0.1337
Test Accuracy: Mean = 95.48%, Std = 13.67%
Total Computation Time: 7.59 seconds

## Test #4. No QuantClass + Quantfilter 2x2
Train Loss: Mean = 1.2719, Std = 0.1111
Train Accuracy: Mean = 36.95%, Std = 7.54%
Validation Loss: Mean = 1.3985, Std = 0.0278
Validation Accuracy: Mean = 30.77%, Std = 5.18%
Test Loss: Mean = 1.4083, Std = 0.0210
Test Accuracy: Mean = 22.03%, Std = 3.74%
Total Computation Time: 4.45 seconds

## Test #5. No QuantClass + Quantfilter 4x4
Train Loss: Mean = 0.0692, Std = 0.2347
Train Accuracy: Mean = 97.72%, Std = 9.87%
Validation Loss: Mean = 3.4513, Std = 0.7994
Validation Accuracy: Mean = 20.82%, Std = 5.42%
Test Loss: Mean = 3.5621, Std = 0.8763
Test Accuracy: Mean = 20.72%, Std = 4.46%
Total Computation Time: 40.73 seconds
```

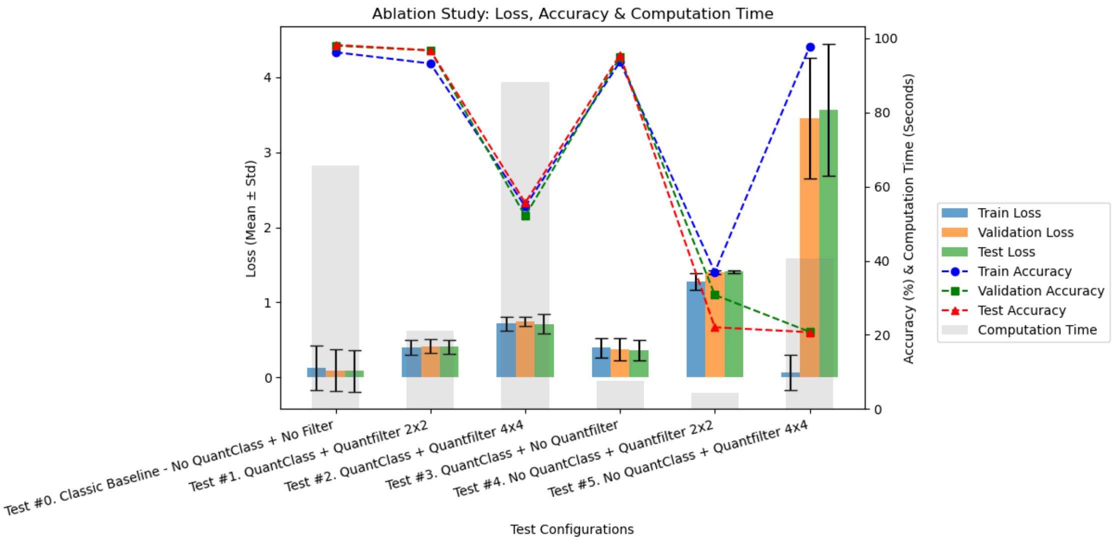

1.2 ANOVA and Tukey HSD Results

[7]:
```
import re
import pandas as pd
import scipy.stats as stats
import statsmodels.api as sm
from statsmodels.formula.api import ols
from statsmodels.stats.multicomp import pairwise_tukeyhsd
import matplotlib.pyplot as plt
import seaborn as sns


# Example log data (Replace with actual log text)
log_data = """
## test 1. Quantvolution + Quantfilter 2x2
--- Overall Metrics ---
Train Loss: Mean = 0.4027, Std = 0.0979
Train Accuracy: Mean = 93.19%, Std = 11.27%
Validation Loss: Mean = 0.4192, Std = 0.0965
Validation Accuracy: Mean = 96.73%, Std = 12.33%
Test Loss: Mean = 0.4075, Std = 0.0922
Test Accuracy: Mean = 96.72%, Std = 11.44%
Total Computation Time: 65.76 seconds

Epoch [1/100]  Train Loss: 0.8170, Train Acc: 48.21%  Val Loss: 0.8414, Val Acc:
↪ 45.45%  Test Loss: 0.8257, Test Acc: 48.28%
```

```
Epoch [2/100]  Train Loss: 0.8147, Train Acc: 48.21%  Val Loss: 0.8376, Val Acc:
 ↪ 45.45%  Test Loss: 0.8114, Test Acc: 48.28%
Epoch [3/100]  Train Loss: 0.8093, Train Acc: 48.21%  Val Loss: 0.8423, Val Acc:
 ↪ 45.45%  Test Loss: 0.8067, Test Acc: 48.28%
Epoch [4/100]  Train Loss: 0.8007, Train Acc: 48.21%  Val Loss: 0.8239, Val Acc:
 ↪ 45.45%  Test Loss: 0.8011, Test Acc: 48.28%
Epoch [5/100]  Train Loss: 0.7692, Train Acc: 48.21%  Val Loss: 0.7946, Val Acc:
 ↪ 45.45%  Test Loss: 0.7531, Test Acc: 48.28%
Epoch [6/100]  Train Loss: 0.6491, Train Acc: 56.41%  Val Loss: 0.5628, Val Acc:
 ↪ 63.64%  Test Loss: 0.5355, Test Acc: 75.86%
Epoch [7/100]  Train Loss: 0.4833, Train Acc: 85.64%  Val Loss: 0.4478, Val Acc:
 ↪ 95.45%  Test Loss: 0.4141, Test Acc: 96.55%
Epoch [8/100]  Train Loss: 0.4571, Train Acc: 90.26%  Val Loss: 0.4323, Val Acc:
 ↪ 95.45%  Test Loss: 0.4111, Test Acc: 96.55%
Epoch [9/100]  Train Loss: 0.4281, Train Acc: 93.85%  Val Loss: 0.4051, Val Acc:
 ↪ 100.00%  Test Loss: 0.3932, Test Acc: 100.00%
Epoch [10/100]  Train Loss: 0.4194, Train Acc: 93.33%  Val Loss: 0.4147, Val␣
 ↪Acc: 95.45%  Test Loss: 0.4022, Test Acc: 96.55%
Epoch [11/100]  Train Loss: 0.4257, Train Acc: 94.87%  Val Loss: 0.3890, Val␣
 ↪Acc: 100.00%  Test Loss: 0.3776, Test Acc: 100.00%
Epoch [12/100]  Train Loss: 0.4201, Train Acc: 93.33%  Val Loss: 0.3922, Val␣
 ↪Acc: 100.00%  Test Loss: 0.3860, Test Acc: 100.00%
Epoch [13/100]  Train Loss: 0.4166, Train Acc: 93.33%  Val Loss: 0.3878, Val␣
 ↪Acc: 100.00%  Test Loss: 0.3816, Test Acc: 100.00%
Epoch [14/100]  Train Loss: 0.4110, Train Acc: 93.33%  Val Loss: 0.3800, Val␣
 ↪Acc: 100.00%  Test Loss: 0.3779, Test Acc: 96.55%
Epoch [15/100]  Train Loss: 0.3954, Train Acc: 93.85%  Val Loss: 0.3972, Val␣
 ↪Acc: 100.00%  Test Loss: 0.3970, Test Acc: 96.55%
Epoch [16/100]  Train Loss: 0.3891, Train Acc: 95.38%  Val Loss: 0.3963, Val␣
 ↪Acc: 100.00%  Test Loss: 0.3996, Test Acc: 100.00%
Epoch [17/100]  Train Loss: 0.3981, Train Acc: 94.87%  Val Loss: 0.4150, Val␣
 ↪Acc: 100.00%  Test Loss: 0.4095, Test Acc: 93.10%
Epoch [18/100]  Train Loss: 0.3830, Train Acc: 94.87%  Val Loss: 0.4041, Val␣
 ↪Acc: 100.00%  Test Loss: 0.4000, Test Acc: 93.10%
Epoch [19/100]  Train Loss: 0.3900, Train Acc: 94.87%  Val Loss: 0.4168, Val␣
 ↪Acc: 100.00%  Test Loss: 0.3943, Test Acc: 100.00%
Epoch [20/100]  Train Loss: 0.3943, Train Acc: 92.31%  Val Loss: 0.4097, Val␣
 ↪Acc: 100.00%  Test Loss: 0.4052, Test Acc: 96.55%
Epoch [21/100]  Train Loss: 0.4016, Train Acc: 94.87%  Val Loss: 0.3773, Val␣
 ↪Acc: 100.00%  Test Loss: 0.3717, Test Acc: 100.00%
Epoch [22/100]  Train Loss: 0.4045, Train Acc: 93.85%  Val Loss: 0.4003, Val␣
 ↪Acc: 100.00%  Test Loss: 0.4028, Test Acc: 96.55%
Epoch [23/100]  Train Loss: 0.3780, Train Acc: 97.44%  Val Loss: 0.3977, Val␣
 ↪Acc: 100.00%  Test Loss: 0.3932, Test Acc: 100.00%
```

```
Epoch [24/100]  Train Loss: 0.3951, Train Acc: 93.85%  Val Loss: 0.4301, Val␣
 ↪Acc: 95.45%  Test Loss: 0.4271, Test Acc: 96.55%
Epoch [25/100]  Train Loss: 0.3943, Train Acc: 91.28%  Val Loss: 0.3985, Val␣
 ↪Acc: 100.00%  Test Loss: 0.3851, Test Acc: 100.00%
Epoch [26/100]  Train Loss: 0.3948, Train Acc: 93.85%  Val Loss: 0.4112, Val␣
 ↪Acc: 100.00%  Test Loss: 0.3976, Test Acc: 100.00%
Epoch [27/100]  Train Loss: 0.3815, Train Acc: 93.85%  Val Loss: 0.3731, Val␣
 ↪Acc: 100.00%  Test Loss: 0.3816, Test Acc: 100.00%
Epoch [28/100]  Train Loss: 0.3987, Train Acc: 92.31%  Val Loss: 0.3999, Val␣
 ↪Acc: 100.00%  Test Loss: 0.4003, Test Acc: 100.00%
Epoch [29/100]  Train Loss: 0.3840, Train Acc: 95.90%  Val Loss: 0.3731, Val␣
 ↪Acc: 100.00%  Test Loss: 0.3745, Test Acc: 100.00%
Epoch [30/100]  Train Loss: 0.3735, Train Acc: 96.92%  Val Loss: 0.3834, Val␣
 ↪Acc: 100.00%  Test Loss: 0.3779, Test Acc: 100.00%
Epoch [31/100]  Train Loss: 0.3786, Train Acc: 96.92%  Val Loss: 0.4059, Val␣
 ↪Acc: 100.00%  Test Loss: 0.3917, Test Acc: 100.00%
Epoch [32/100]  Train Loss: 0.3775, Train Acc: 96.41%  Val Loss: 0.3837, Val␣
 ↪Acc: 100.00%  Test Loss: 0.3650, Test Acc: 100.00%
Epoch [33/100]  Train Loss: 0.3905, Train Acc: 94.87%  Val Loss: 0.3782, Val␣
 ↪Acc: 100.00%  Test Loss: 0.3657, Test Acc: 100.00%
Epoch [34/100]  Train Loss: 0.3860, Train Acc: 93.33%  Val Loss: 0.4269, Val␣
 ↪Acc: 100.00%  Test Loss: 0.3952, Test Acc: 100.00%
Epoch [35/100]  Train Loss: 0.3901, Train Acc: 94.36%  Val Loss: 0.4138, Val␣
 ↪Acc: 100.00%  Test Loss: 0.3922, Test Acc: 100.00%
Epoch [36/100]  Train Loss: 0.3761, Train Acc: 94.87%  Val Loss: 0.3688, Val␣
 ↪Acc: 100.00%  Test Loss: 0.3819, Test Acc: 100.00%
Epoch [37/100]  Train Loss: 0.3858, Train Acc: 95.90%  Val Loss: 0.3810, Val␣
 ↪Acc: 100.00%  Test Loss: 0.3685, Test Acc: 100.00%
Epoch [38/100]  Train Loss: 0.3954, Train Acc: 94.36%  Val Loss: 0.4098, Val␣
 ↪Acc: 100.00%  Test Loss: 0.4008, Test Acc: 100.00%
Epoch [39/100]  Train Loss: 0.3817, Train Acc: 95.38%  Val Loss: 0.3985, Val␣
 ↪Acc: 100.00%  Test Loss: 0.3935, Test Acc: 100.00%
Epoch [40/100]  Train Loss: 0.3700, Train Acc: 97.44%  Val Loss: 0.4197, Val␣
 ↪Acc: 100.00%  Test Loss: 0.4080, Test Acc: 100.00%
Epoch [41/100]  Train Loss: 0.3896, Train Acc: 94.36%  Val Loss: 0.4229, Val␣
 ↪Acc: 100.00%  Test Loss: 0.4022, Test Acc: 100.00%
Epoch [42/100]  Train Loss: 0.3879, Train Acc: 96.41%  Val Loss: 0.3837, Val␣
 ↪Acc: 100.00%  Test Loss: 0.3780, Test Acc: 100.00%
Epoch [43/100]  Train Loss: 0.3740, Train Acc: 95.90%  Val Loss: 0.3899, Val␣
 ↪Acc: 100.00%  Test Loss: 0.3870, Test Acc: 100.00%
Epoch [44/100]  Train Loss: 0.3785, Train Acc: 97.44%  Val Loss: 0.3649, Val␣
 ↪Acc: 100.00%  Test Loss: 0.3652, Test Acc: 100.00%
Epoch [45/100]  Train Loss: 0.3881, Train Acc: 93.85%  Val Loss: 0.3682, Val␣
 ↪Acc: 100.00%  Test Loss: 0.3743, Test Acc: 100.00%
```

```
Epoch [46/100]  Train Loss: 0.3782, Train Acc: 94.36%  Val Loss: 0.3640, Val␣
 ↪Acc: 100.00%  Test Loss: 0.3639, Test Acc: 100.00%
Epoch [47/100]  Train Loss: 0.3809, Train Acc: 95.38%  Val Loss: 0.3977, Val␣
 ↪Acc: 100.00%  Test Loss: 0.3831, Test Acc: 100.00%
Epoch [48/100]  Train Loss: 0.3789, Train Acc: 94.36%  Val Loss: 0.3934, Val␣
 ↪Acc: 100.00%  Test Loss: 0.3843, Test Acc: 100.00%
Epoch [49/100]  Train Loss: 0.3764, Train Acc: 96.41%  Val Loss: 0.3844, Val␣
 ↪Acc: 100.00%  Test Loss: 0.3768, Test Acc: 100.00%
Epoch [50/100]  Train Loss: 0.3840, Train Acc: 94.87%  Val Loss: 0.3932, Val␣
 ↪Acc: 100.00%  Test Loss: 0.3768, Test Acc: 100.00%
Epoch [51/100]  Train Loss: 0.3702, Train Acc: 95.38%  Val Loss: 0.3895, Val␣
 ↪Acc: 100.00%  Test Loss: 0.3830, Test Acc: 100.00%
Epoch [52/100]  Train Loss: 0.3730, Train Acc: 97.95%  Val Loss: 0.3970, Val␣
 ↪Acc: 100.00%  Test Loss: 0.3830, Test Acc: 100.00%
Epoch [53/100]  Train Loss: 0.3791, Train Acc: 96.92%  Val Loss: 0.3928, Val␣
 ↪Acc: 100.00%  Test Loss: 0.3787, Test Acc: 100.00%
Epoch [54/100]  Train Loss: 0.3575, Train Acc: 98.97%  Val Loss: 0.3938, Val␣
 ↪Acc: 100.00%  Test Loss: 0.3784, Test Acc: 100.00%
Epoch [55/100]  Train Loss: 0.3748, Train Acc: 95.38%  Val Loss: 0.3982, Val␣
 ↪Acc: 100.00%  Test Loss: 0.3806, Test Acc: 100.00%
Epoch [56/100]  Train Loss: 0.3755, Train Acc: 95.90%  Val Loss: 0.3734, Val␣
 ↪Acc: 100.00%  Test Loss: 0.3563, Test Acc: 100.00%
Epoch [57/100]  Train Loss: 0.3645, Train Acc: 96.92%  Val Loss: 0.3744, Val␣
 ↪Acc: 100.00%  Test Loss: 0.3675, Test Acc: 100.00%
Epoch [58/100]  Train Loss: 0.3737, Train Acc: 96.41%  Val Loss: 0.3757, Val␣
 ↪Acc: 100.00%  Test Loss: 0.3590, Test Acc: 100.00%
Epoch [59/100]  Train Loss: 0.3789, Train Acc: 94.87%  Val Loss: 0.3979, Val␣
 ↪Acc: 100.00%  Test Loss: 0.3804, Test Acc: 100.00%
Epoch [60/100]  Train Loss: 0.3741, Train Acc: 95.90%  Val Loss: 0.3927, Val␣
 ↪Acc: 100.00%  Test Loss: 0.3817, Test Acc: 100.00%
Epoch [61/100]  Train Loss: 0.3700, Train Acc: 95.90%  Val Loss: 0.4042, Val␣
 ↪Acc: 100.00%  Test Loss: 0.4039, Test Acc: 96.55%
Epoch [62/100]  Train Loss: 0.3770, Train Acc: 94.87%  Val Loss: 0.4088, Val␣
 ↪Acc: 100.00%  Test Loss: 0.3904, Test Acc: 100.00%
Epoch [63/100]  Train Loss: 0.3764, Train Acc: 95.90%  Val Loss: 0.3781, Val␣
 ↪Acc: 100.00%  Test Loss: 0.3798, Test Acc: 100.00%
Epoch [64/100]  Train Loss: 0.3749, Train Acc: 96.41%  Val Loss: 0.3934, Val␣
 ↪Acc: 100.00%  Test Loss: 0.3934, Test Acc: 100.00%
Epoch [65/100]  Train Loss: 0.3695, Train Acc: 95.38%  Val Loss: 0.3837, Val␣
 ↪Acc: 100.00%  Test Loss: 0.3824, Test Acc: 100.00%
Epoch [66/100]  Train Loss: 0.3689, Train Acc: 96.41%  Val Loss: 0.3794, Val␣
 ↪Acc: 100.00%  Test Loss: 0.3729, Test Acc: 100.00%
Epoch [67/100]  Train Loss: 0.3709, Train Acc: 96.92%  Val Loss: 0.4020, Val␣
 ↪Acc: 100.00%  Test Loss: 0.3826, Test Acc: 100.00%
```

```
Epoch [68/100]  Train Loss: 0.3721, Train Acc: 96.41%  Val Loss: 0.4206, Val␣
 ↪Acc: 100.00%  Test Loss: 0.4081, Test Acc: 100.00%
Epoch [69/100]  Train Loss: 0.3625, Train Acc: 96.41%  Val Loss: 0.3901, Val␣
 ↪Acc: 100.00%  Test Loss: 0.3818, Test Acc: 100.00%
Epoch [70/100]  Train Loss: 0.3684, Train Acc: 96.92%  Val Loss: 0.3991, Val␣
 ↪Acc: 100.00%  Test Loss: 0.3814, Test Acc: 100.00%
Epoch [71/100]  Train Loss: 0.3722, Train Acc: 96.92%  Val Loss: 0.3833, Val␣
 ↪Acc: 100.00%  Test Loss: 0.3804, Test Acc: 100.00%
Epoch [72/100]  Train Loss: 0.3672, Train Acc: 97.44%  Val Loss: 0.3697, Val␣
 ↪Acc: 100.00%  Test Loss: 0.3703, Test Acc: 100.00%
Epoch [73/100]  Train Loss: 0.3680, Train Acc: 96.41%  Val Loss: 0.3853, Val␣
 ↪Acc: 100.00%  Test Loss: 0.3719, Test Acc: 100.00%
Epoch [74/100]  Train Loss: 0.3689, Train Acc: 96.41%  Val Loss: 0.3791, Val␣
 ↪Acc: 100.00%  Test Loss: 0.3705, Test Acc: 100.00%
Epoch [75/100]  Train Loss: 0.3634, Train Acc: 98.46%  Val Loss: 0.3873, Val␣
 ↪Acc: 100.00%  Test Loss: 0.3783, Test Acc: 100.00%
Epoch [76/100]  Train Loss: 0.3652, Train Acc: 96.41%  Val Loss: 0.4135, Val␣
 ↪Acc: 100.00%  Test Loss: 0.4014, Test Acc: 100.00%
Epoch [77/100]  Train Loss: 0.3659, Train Acc: 97.95%  Val Loss: 0.3990, Val␣
 ↪Acc: 100.00%  Test Loss: 0.3901, Test Acc: 100.00%
Epoch [78/100]  Train Loss: 0.3698, Train Acc: 96.41%  Val Loss: 0.3893, Val␣
 ↪Acc: 100.00%  Test Loss: 0.3740, Test Acc: 100.00%
Epoch [79/100]  Train Loss: 0.3764, Train Acc: 93.33%  Val Loss: 0.3923, Val␣
 ↪Acc: 100.00%  Test Loss: 0.3784, Test Acc: 100.00%
Epoch [80/100]  Train Loss: 0.3667, Train Acc: 96.92%  Val Loss: 0.4263, Val␣
 ↪Acc: 100.00%  Test Loss: 0.4019, Test Acc: 100.00%
Epoch [81/100]  Train Loss: 0.3472, Train Acc: 99.49%  Val Loss: 0.3803, Val␣
 ↪Acc: 100.00%  Test Loss: 0.3738, Test Acc: 100.00%
Epoch [82/100]  Train Loss: 0.3636, Train Acc: 98.97%  Val Loss: 0.3939, Val␣
 ↪Acc: 100.00%  Test Loss: 0.3744, Test Acc: 100.00%
Epoch [83/100]  Train Loss: 0.3605, Train Acc: 98.97%  Val Loss: 0.4050, Val␣
 ↪Acc: 100.00%  Test Loss: 0.3785, Test Acc: 100.00%
Epoch [84/100]  Train Loss: 0.3644, Train Acc: 96.92%  Val Loss: 0.3952, Val␣
 ↪Acc: 100.00%  Test Loss: 0.3745, Test Acc: 100.00%
Epoch [85/100]  Train Loss: 0.3581, Train Acc: 99.49%  Val Loss: 0.3977, Val␣
 ↪Acc: 100.00%  Test Loss: 0.3776, Test Acc: 100.00%
Epoch [86/100]  Train Loss: 0.3566, Train Acc: 98.46%  Val Loss: 0.4034, Val␣
 ↪Acc: 100.00%  Test Loss: 0.3798, Test Acc: 100.00%
Epoch [87/100]  Train Loss: 0.3621, Train Acc: 97.44%  Val Loss: 0.4021, Val␣
 ↪Acc: 100.00%  Test Loss: 0.3757, Test Acc: 100.00%
Epoch [88/100]  Train Loss: 0.3556, Train Acc: 97.95%  Val Loss: 0.4011, Val␣
 ↪Acc: 100.00%  Test Loss: 0.3827, Test Acc: 100.00%
Epoch [89/100]  Train Loss: 0.3535, Train Acc: 99.49%  Val Loss: 0.3969, Val␣
 ↪Acc: 100.00%  Test Loss: 0.3845, Test Acc: 100.00%
```

```
Epoch [90/100]  Train Loss: 0.3517, Train Acc: 98.97%  Val Loss: 0.4027, Val␣
 ↪Acc: 100.00%  Test Loss: 0.3832, Test Acc: 100.00%
Epoch [91/100]  Train Loss: 0.3556, Train Acc: 98.46%  Val Loss: 0.3815, Val␣
 ↪Acc: 100.00%  Test Loss: 0.3787, Test Acc: 100.00%
Epoch [92/100]  Train Loss: 0.3558, Train Acc: 99.49%  Val Loss: 0.3936, Val␣
 ↪Acc: 100.00%  Test Loss: 0.3892, Test Acc: 100.00%
Epoch [93/100]  Train Loss: 0.3533, Train Acc: 98.97%  Val Loss: 0.4163, Val␣
 ↪Acc: 100.00%  Test Loss: 0.4011, Test Acc: 100.00%
Epoch [94/100]  Train Loss: 0.3465, Train Acc: 99.49%  Val Loss: 0.4023, Val␣
 ↪Acc: 100.00%  Test Loss: 0.3899, Test Acc: 100.00%
Epoch [95/100]  Train Loss: 0.3624, Train Acc: 98.97%  Val Loss: 0.3841, Val␣
 ↪Acc: 100.00%  Test Loss: 0.3772, Test Acc: 100.00%
Epoch [96/100]  Train Loss: 0.3546, Train Acc: 99.49%  Val Loss: 0.3918, Val␣
 ↪Acc: 100.00%  Test Loss: 0.3781, Test Acc: 100.00%
Epoch [97/100]  Train Loss: 0.3588, Train Acc: 98.46%  Val Loss: 0.4100, Val␣
 ↪Acc: 100.00%  Test Loss: 0.3885, Test Acc: 100.00%
Epoch [98/100]  Train Loss: 0.3562, Train Acc: 98.46%  Val Loss: 0.4013, Val␣
 ↪Acc: 100.00%  Test Loss: 0.3895, Test Acc: 100.00%
Epoch [99/100]  Train Loss: 0.3548, Train Acc: 98.97%  Val Loss: 0.4068, Val␣
 ↪Acc: 100.00%  Test Loss: 0.3875, Test Acc: 100.00%
Epoch [100/100]  Train Loss: 0.3620, Train Acc: 96.92%  Val Loss: 0.4047, Val␣
 ↪Acc: 100.00%  Test Loss: 0.3950, Test Acc: 100.00%


## test 2. Quantvolution + Quantfilter 4x4
--- Overall Metrics ---
Train Loss: Mean = 0.7175, Std = 0.0927
Train Accuracy: Mean = 54.65%, Std = 11.17%
Validation Loss: Mean = 0.7454, Std = 0.0620
Validation Accuracy: Mean = 52.05%, Std = 9.46%
Test Loss: Mean = 0.7127, Std = 0.1271
Test Accuracy: Mean = 55.62%, Std = 15.89%
Total Computation Time: 21.02 seconds

Epoch [1/100]  Train Loss: 0.6655, Train Acc: 64.62%  Val Loss: 0.7206, Val Acc:
 ↪ 59.09%  Test Loss: 0.4827, Test Acc: 82.76%
Epoch [2/100]  Train Loss: 0.6652, Train Acc: 64.62%  Val Loss: 0.7200, Val Acc:
 ↪ 59.09%  Test Loss: 0.4840, Test Acc: 82.76%
Epoch [3/100]  Train Loss: 0.6636, Train Acc: 64.62%  Val Loss: 0.7147, Val Acc:
 ↪ 59.09%  Test Loss: 0.4872, Test Acc: 82.76%
Epoch [4/100]  Train Loss: 0.6585, Train Acc: 64.62%  Val Loss: 0.7073, Val Acc:
 ↪ 59.09%  Test Loss: 0.4849, Test Acc: 82.76%
Epoch [5/100]  Train Loss: 0.6598, Train Acc: 64.62%  Val Loss: 0.7110, Val Acc:
 ↪ 59.09%  Test Loss: 0.4861, Test Acc: 82.76%
Epoch [6/100]  Train Loss: 0.6460, Train Acc: 64.62%  Val Loss: 0.7203, Val Acc:
 ↪ 59.09%  Test Loss: 0.4889, Test Acc: 82.76%
```

```
Epoch [7/100]  Train Loss: 0.6410, Train Acc: 64.62%  Val Loss: 0.7094, Val Acc:
 ↪ 59.09%  Test Loss: 0.4866, Test Acc: 82.76%
Epoch [8/100]  Train Loss: 0.6258, Train Acc: 64.62%  Val Loss: 0.7126, Val Acc:
 ↪ 59.09%  Test Loss: 0.4936, Test Acc: 82.76%
Epoch [9/100]  Train Loss: 0.6189, Train Acc: 64.62%  Val Loss: 0.7019, Val Acc:
 ↪ 59.09%  Test Loss: 0.4903, Test Acc: 82.76%
Epoch [10/100]  Train Loss: 0.6044, Train Acc: 64.62%  Val Loss: 0.7019, Val␣
 ↪Acc: 59.09%  Test Loss: 0.4918, Test Acc: 82.76%
Epoch [11/100]  Train Loss: 0.5758, Train Acc: 65.13%  Val Loss: 0.7016, Val␣
 ↪Acc: 59.09%  Test Loss: 0.4867, Test Acc: 82.76%
Epoch [12/100]  Train Loss: 0.5510, Train Acc: 69.23%  Val Loss: 0.6950, Val␣
 ↪Acc: 59.09%  Test Loss: 0.4966, Test Acc: 82.76%
Epoch [13/100]  Train Loss: 0.5286, Train Acc: 73.33%  Val Loss: 0.6904, Val␣
 ↪Acc: 59.09%  Test Loss: 0.4936, Test Acc: 82.76%
Epoch [14/100]  Train Loss: 0.5277, Train Acc: 72.82%  Val Loss: 0.6900, Val␣
 ↪Acc: 59.09%  Test Loss: 0.4873, Test Acc: 82.76%
Epoch [15/100]  Train Loss: 0.4736, Train Acc: 82.05%  Val Loss: 0.6754, Val␣
 ↪Acc: 59.09%  Test Loss: 0.4981, Test Acc: 82.76%
Epoch [16/100]  Train Loss: 0.4647, Train Acc: 84.62%  Val Loss: 0.7003, Val␣
 ↪Acc: 59.09%  Test Loss: 0.4945, Test Acc: 82.76%
Epoch [17/100]  Train Loss: 0.4369, Train Acc: 88.21%  Val Loss: 0.7016, Val␣
 ↪Acc: 59.09%  Test Loss: 0.4852, Test Acc: 82.76%
Epoch [18/100]  Train Loss: 0.4451, Train Acc: 88.21%  Val Loss: 0.7104, Val␣
 ↪Acc: 54.55%  Test Loss: 0.4872, Test Acc: 82.76%
Epoch [19/100]  Train Loss: 0.4410, Train Acc: 89.74%  Val Loss: 0.7036, Val␣
 ↪Acc: 59.09%  Test Loss: 0.4899, Test Acc: 82.76%
Epoch [20/100]  Train Loss: 0.4446, Train Acc: 91.28%  Val Loss: 0.7197, Val␣
 ↪Acc: 63.64%  Test Loss: 0.4800, Test Acc: 86.21%
Epoch [21/100]  Train Loss: 0.5204, Train Acc: 79.49%  Val Loss: 0.7013, Val␣
 ↪Acc: 63.64%  Test Loss: 0.5133, Test Acc: 79.31%
Epoch [22/100]  Train Loss: 0.6384, Train Acc: 66.15%  Val Loss: 0.7212, Val␣
 ↪Acc: 45.45%  Test Loss: 0.5663, Test Acc: 72.41%
Epoch [23/100]  Train Loss: 0.7956, Train Acc: 46.15%  Val Loss: 0.7115, Val␣
 ↪Acc: 54.55%  Test Loss: 0.6896, Test Acc: 65.52%
Epoch [24/100]  Train Loss: 0.8167, Train Acc: 44.62%  Val Loss: 0.7130, Val␣
 ↪Acc: 59.09%  Test Loss: 0.7556, Test Acc: 51.72%
Epoch [25/100]  Train Loss: 0.7390, Train Acc: 51.79%  Val Loss: 0.7076, Val␣
 ↪Acc: 54.55%  Test Loss: 0.8028, Test Acc: 41.38%
Epoch [26/100]  Train Loss: 0.6912, Train Acc: 55.90%  Val Loss: 0.6976, Val␣
 ↪Acc: 50.00%  Test Loss: 0.8377, Test Acc: 37.93%
Epoch [27/100]  Train Loss: 0.6854, Train Acc: 57.44%  Val Loss: 0.6948, Val␣
 ↪Acc: 54.55%  Test Loss: 0.8201, Test Acc: 37.93%
Epoch [28/100]  Train Loss: 0.6817, Train Acc: 60.51%  Val Loss: 0.6644, Val␣
 ↪Acc: 59.09%  Test Loss: 0.8628, Test Acc: 34.48%
```

```
Epoch [29/100]  Train Loss: 0.6939, Train Acc: 56.92%  Val Loss: 0.6703, Val␣
 ↪Acc: 63.64%  Test Loss: 0.8315, Test Acc: 41.38%
Epoch [30/100]  Train Loss: 0.8162, Train Acc: 40.00%  Val Loss: 0.6586, Val␣
 ↪Acc: 68.18%  Test Loss: 0.8201, Test Acc: 41.38%
Epoch [31/100]  Train Loss: 0.8101, Train Acc: 40.51%  Val Loss: 0.7237, Val␣
 ↪Acc: 54.55%  Test Loss: 0.8432, Test Acc: 44.83%
Epoch [32/100]  Train Loss: 0.7291, Train Acc: 51.79%  Val Loss: 0.7368, Val␣
 ↪Acc: 54.55%  Test Loss: 0.8406, Test Acc: 44.83%
Epoch [33/100]  Train Loss: 0.7653, Train Acc: 49.74%  Val Loss: 0.7239, Val␣
 ↪Acc: 59.09%  Test Loss: 0.7638, Test Acc: 44.83%
Epoch [34/100]  Train Loss: 0.7659, Train Acc: 46.67%  Val Loss: 0.8154, Val␣
 ↪Acc: 36.36%  Test Loss: 0.7529, Test Acc: 51.72%
Epoch [35/100]  Train Loss: 0.8160, Train Acc: 43.08%  Val Loss: 0.7754, Val␣
 ↪Acc: 40.91%  Test Loss: 0.7855, Test Acc: 51.72%
Epoch [36/100]  Train Loss: 0.7476, Train Acc: 49.74%  Val Loss: 0.7155, Val␣
 ↪Acc: 45.45%  Test Loss: 0.8047, Test Acc: 41.38%
Epoch [37/100]  Train Loss: 0.7890, Train Acc: 46.67%  Val Loss: 0.6681, Val␣
 ↪Acc: 50.00%  Test Loss: 0.8026, Test Acc: 37.93%
Epoch [38/100]  Train Loss: 0.7382, Train Acc: 52.31%  Val Loss: 0.7786, Val␣
 ↪Acc: 54.55%  Test Loss: 0.8619, Test Acc: 44.83%
Epoch [39/100]  Train Loss: 0.7265, Train Acc: 53.33%  Val Loss: 0.8268, Val␣
 ↪Acc: 45.45%  Test Loss: 0.8506, Test Acc: 37.93%
Epoch [40/100]  Train Loss: 0.7486, Train Acc: 51.79%  Val Loss: 0.8017, Val␣
 ↪Acc: 50.00%  Test Loss: 0.8396, Test Acc: 48.28%
Epoch [41/100]  Train Loss: 0.7531, Train Acc: 49.23%  Val Loss: 0.8534, Val␣
 ↪Acc: 36.36%  Test Loss: 0.8639, Test Acc: 31.03%
Epoch [42/100]  Train Loss: 0.7540, Train Acc: 47.18%  Val Loss: 0.7591, Val␣
 ↪Acc: 50.00%  Test Loss: 0.8370, Test Acc: 44.83%
Epoch [43/100]  Train Loss: 0.7778, Train Acc: 49.23%  Val Loss: 0.6813, Val␣
 ↪Acc: 63.64%  Test Loss: 0.8521, Test Acc: 37.93%
Epoch [44/100]  Train Loss: 0.7396, Train Acc: 52.82%  Val Loss: 0.7258, Val␣
 ↪Acc: 54.55%  Test Loss: 0.7701, Test Acc: 48.28%
Epoch [45/100]  Train Loss: 0.7402, Train Acc: 56.41%  Val Loss: 0.6494, Val␣
 ↪Acc: 68.18%  Test Loss: 0.7898, Test Acc: 51.72%
Epoch [46/100]  Train Loss: 0.7732, Train Acc: 48.21%  Val Loss: 0.7975, Val␣
 ↪Acc: 54.55%  Test Loss: 0.7593, Test Acc: 48.28%
Epoch [47/100]  Train Loss: 0.7584, Train Acc: 49.23%  Val Loss: 0.7176, Val␣
 ↪Acc: 59.09%  Test Loss: 0.7555, Test Acc: 51.72%
Epoch [48/100]  Train Loss: 0.7869, Train Acc: 47.18%  Val Loss: 0.7184, Val␣
 ↪Acc: 59.09%  Test Loss: 0.8133, Test Acc: 41.38%
Epoch [49/100]  Train Loss: 0.7864, Train Acc: 45.64%  Val Loss: 0.7380, Val␣
 ↪Acc: 54.55%  Test Loss: 0.7655, Test Acc: 51.72%
Epoch [50/100]  Train Loss: 0.8057, Train Acc: 42.56%  Val Loss: 0.7777, Val␣
 ↪Acc: 50.00%  Test Loss: 0.7459, Test Acc: 48.28%
```

```
Epoch [51/100]  Train Loss: 0.7371, Train Acc: 54.36%  Val Loss: 0.7644, Val␣
 ↪Acc: 50.00%  Test Loss: 0.7360, Test Acc: 55.17%
Epoch [52/100]  Train Loss: 0.7738, Train Acc: 47.18%  Val Loss: 0.7697, Val␣
 ↪Acc: 50.00%  Test Loss: 0.7222, Test Acc: 58.62%
Epoch [53/100]  Train Loss: 0.7296, Train Acc: 52.31%  Val Loss: 0.8592, Val␣
 ↪Acc: 31.82%  Test Loss: 0.7488, Test Acc: 48.28%
Epoch [54/100]  Train Loss: 0.7241, Train Acc: 56.92%  Val Loss: 0.8065, Val␣
 ↪Acc: 50.00%  Test Loss: 0.7351, Test Acc: 48.28%
Epoch [55/100]  Train Loss: 0.7681, Train Acc: 49.74%  Val Loss: 0.7544, Val␣
 ↪Acc: 50.00%  Test Loss: 0.7030, Test Acc: 62.07%
Epoch [56/100]  Train Loss: 0.7986, Train Acc: 45.64%  Val Loss: 0.8722, Val␣
 ↪Acc: 36.36%  Test Loss: 0.8398, Test Acc: 41.38%
Epoch [57/100]  Train Loss: 0.7748, Train Acc: 48.21%  Val Loss: 0.7164, Val␣
 ↪Acc: 45.45%  Test Loss: 0.7334, Test Acc: 55.17%
Epoch [58/100]  Train Loss: 0.7891, Train Acc: 46.15%  Val Loss: 0.8431, Val␣
 ↪Acc: 45.45%  Test Loss: 0.7328, Test Acc: 55.17%
Epoch [59/100]  Train Loss: 0.7846, Train Acc: 47.18%  Val Loss: 0.8344, Val␣
 ↪Acc: 40.91%  Test Loss: 0.7527, Test Acc: 48.28%
Epoch [60/100]  Train Loss: 0.7805, Train Acc: 46.67%  Val Loss: 0.7085, Val␣
 ↪Acc: 54.55%  Test Loss: 0.7132, Test Acc: 55.17%
Epoch [61/100]  Train Loss: 0.7728, Train Acc: 47.18%  Val Loss: 0.7009, Val␣
 ↪Acc: 59.09%  Test Loss: 0.7102, Test Acc: 55.17%
Epoch [62/100]  Train Loss: 0.7621, Train Acc: 49.23%  Val Loss: 0.8623, Val␣
 ↪Acc: 36.36%  Test Loss: 0.8091, Test Acc: 44.83%
Epoch [63/100]  Train Loss: 0.7631, Train Acc: 50.77%  Val Loss: 0.8338, Val␣
 ↪Acc: 36.36%  Test Loss: 0.7226, Test Acc: 55.17%
Epoch [64/100]  Train Loss: 0.7723, Train Acc: 49.23%  Val Loss: 0.7346, Val␣
 ↪Acc: 54.55%  Test Loss: 0.7934, Test Acc: 37.93%
Epoch [65/100]  Train Loss: 0.7531, Train Acc: 50.26%  Val Loss: 0.7117, Val␣
 ↪Acc: 59.09%  Test Loss: 0.7044, Test Acc: 55.17%
Epoch [66/100]  Train Loss: 0.7410, Train Acc: 50.77%  Val Loss: 0.6627, Val␣
 ↪Acc: 63.64%  Test Loss: 0.7151, Test Acc: 55.17%
Epoch [67/100]  Train Loss: 0.7486, Train Acc: 47.18%  Val Loss: 0.7961, Val␣
 ↪Acc: 45.45%  Test Loss: 0.7604, Test Acc: 51.72%
Epoch [68/100]  Train Loss: 0.7712, Train Acc: 50.26%  Val Loss: 0.8302, Val␣
 ↪Acc: 50.00%  Test Loss: 0.8116, Test Acc: 41.38%
Epoch [69/100]  Train Loss: 0.7684, Train Acc: 45.13%  Val Loss: 0.9572, Val␣
 ↪Acc: 22.73%  Test Loss: 0.6967, Test Acc: 58.62%
Epoch [70/100]  Train Loss: 0.7589, Train Acc: 48.72%  Val Loss: 0.7212, Val␣
 ↪Acc: 54.55%  Test Loss: 0.7728, Test Acc: 44.83%
Epoch [71/100]  Train Loss: 0.7205, Train Acc: 56.41%  Val Loss: 0.6761, Val␣
 ↪Acc: 59.09%  Test Loss: 0.7612, Test Acc: 48.28%
Epoch [72/100]  Train Loss: 0.7625, Train Acc: 50.26%  Val Loss: 0.7506, Val␣
 ↪Acc: 50.00%  Test Loss: 0.7743, Test Acc: 51.72%
```

```
Epoch [73/100]  Train Loss: 0.7158, Train Acc: 54.87%  Val Loss: 0.8542, Val␣
 ↪Acc: 40.91%  Test Loss: 0.8180, Test Acc: 44.83%
Epoch [74/100]  Train Loss: 0.7813, Train Acc: 47.69%  Val Loss: 0.8266, Val␣
 ↪Acc: 36.36%  Test Loss: 0.8293, Test Acc: 41.38%
Epoch [75/100]  Train Loss: 0.7825, Train Acc: 47.18%  Val Loss: 0.7729, Val␣
 ↪Acc: 50.00%  Test Loss: 0.7669, Test Acc: 51.72%
Epoch [76/100]  Train Loss: 0.7928, Train Acc: 46.15%  Val Loss: 0.8429, Val␣
 ↪Acc: 36.36%  Test Loss: 0.8329, Test Acc: 41.38%
Epoch [77/100]  Train Loss: 0.7595, Train Acc: 51.79%  Val Loss: 0.7616, Val␣
 ↪Acc: 40.91%  Test Loss: 0.8421, Test Acc: 37.93%
Epoch [78/100]  Train Loss: 0.7337, Train Acc: 50.77%  Val Loss: 0.7284, Val␣
 ↪Acc: 45.45%  Test Loss: 0.7839, Test Acc: 44.83%
Epoch [79/100]  Train Loss: 0.7536, Train Acc: 51.79%  Val Loss: 0.6684, Val␣
 ↪Acc: 63.64%  Test Loss: 0.7133, Test Acc: 55.17%
Epoch [80/100]  Train Loss: 0.7630, Train Acc: 50.26%  Val Loss: 0.7246, Val␣
 ↪Acc: 54.55%  Test Loss: 0.8940, Test Acc: 27.59%
Epoch [81/100]  Train Loss: 0.8168, Train Acc: 42.56%  Val Loss: 0.8251, Val␣
 ↪Acc: 40.91%  Test Loss: 0.6452, Test Acc: 55.17%
Epoch [82/100]  Train Loss: 0.7879, Train Acc: 43.08%  Val Loss: 0.6478, Val␣
 ↪Acc: 59.09%  Test Loss: 0.7422, Test Acc: 41.38%
Epoch [83/100]  Train Loss: 0.7275, Train Acc: 53.33%  Val Loss: 0.8243, Val␣
 ↪Acc: 40.91%  Test Loss: 0.7032, Test Acc: 58.62%
Epoch [84/100]  Train Loss: 0.7909, Train Acc: 46.15%  Val Loss: 0.8476, Val␣
 ↪Acc: 22.73%  Test Loss: 0.7322, Test Acc: 58.62%
Epoch [85/100]  Train Loss: 0.7689, Train Acc: 47.69%  Val Loss: 0.7336, Val␣
 ↪Acc: 59.09%  Test Loss: 0.7322, Test Acc: 55.17%
Epoch [86/100]  Train Loss: 0.7874, Train Acc: 46.67%  Val Loss: 0.7656, Val␣
 ↪Acc: 50.00%  Test Loss: 0.7326, Test Acc: 51.72%
Epoch [87/100]  Train Loss: 0.7239, Train Acc: 54.87%  Val Loss: 0.7421, Val␣
 ↪Acc: 59.09%  Test Loss: 0.6047, Test Acc: 68.97%
Epoch [88/100]  Train Loss: 0.7420, Train Acc: 50.77%  Val Loss: 0.7185, Val␣
 ↪Acc: 59.09%  Test Loss: 0.7541, Test Acc: 51.72%
Epoch [89/100]  Train Loss: 0.7374, Train Acc: 52.31%  Val Loss: 0.7820, Val␣
 ↪Acc: 45.45%  Test Loss: 0.7496, Test Acc: 41.38%
Epoch [90/100]  Train Loss: 0.7867, Train Acc: 46.15%  Val Loss: 0.7404, Val␣
 ↪Acc: 45.45%  Test Loss: 0.7373, Test Acc: 44.83%
Epoch [91/100]  Train Loss: 0.7643, Train Acc: 49.23%  Val Loss: 0.6752, Val␣
 ↪Acc: 54.55%  Test Loss: 0.7998, Test Acc: 48.28%
Epoch [92/100]  Train Loss: 0.7463, Train Acc: 51.79%  Val Loss: 0.8199, Val␣
 ↪Acc: 36.36%  Test Loss: 0.6627, Test Acc: 68.97%
Epoch [93/100]  Train Loss: 0.7317, Train Acc: 51.79%  Val Loss: 0.6888, Val␣
 ↪Acc: 54.55%  Test Loss: 0.7587, Test Acc: 55.17%
Epoch [94/100]  Train Loss: 0.7600, Train Acc: 49.74%  Val Loss: 0.8383, Val␣
 ↪Acc: 36.36%  Test Loss: 0.8402, Test Acc: 41.38%
```

```
Epoch [95/100]  Train Loss: 0.7581, Train Acc: 51.79%  Val Loss: 0.8219, Val␣
 ↪Acc: 50.00%  Test Loss: 0.8003, Test Acc: 51.72%
Epoch [96/100]  Train Loss: 0.7778, Train Acc: 47.18%  Val Loss: 0.7322, Val␣
 ↪Acc: 59.09%  Test Loss: 0.8443, Test Acc: 41.38%
Epoch [97/100]  Train Loss: 0.7208, Train Acc: 55.90%  Val Loss: 0.8166, Val␣
 ↪Acc: 40.91%  Test Loss: 0.7003, Test Acc: 55.17%
Epoch [98/100]  Train Loss: 0.7262, Train Acc: 53.85%  Val Loss: 0.6522, Val␣
 ↪Acc: 68.18%  Test Loss: 0.7598, Test Acc: 51.72%
Epoch [99/100]  Train Loss: 0.7561, Train Acc: 50.77%  Val Loss: 0.8001, Val␣
 ↪Acc: 54.55%  Test Loss: 0.8461, Test Acc: 37.93%
Epoch [100/100]  Train Loss: 0.7785, Train Acc: 45.64%  Val Loss: 0.6929, Val␣
 ↪Acc: 59.09%  Test Loss: 0.8226, Test Acc: 44.83%

## test 3. Quantvolution + No Quantfilter (original images)
--- Overall Metrics ---
Train Loss: Mean = 0.3960, Std = 0.1323
Train Accuracy: Mean = 93.66%, Std = 14.00%
Validation Loss: Mean = 0.3731, Std = 0.1472
Validation Accuracy: Mean = 94.94%, Std = 15.27%
Test Loss: Mean = 0.3656, Std = 0.1337
Test Accuracy: Mean = 95.48%, Std = 13.67%
Total Computation Time: 88.26 seconds

Epoch [1/100]  Train Loss: 0.8074, Train Acc: 50.59%  Val Loss: 0.8427, Val Acc:
 ↪ 47.06%  Test Loss: 0.7915, Test Acc: 52.17%
Epoch [2/100]  Train Loss: 0.8074, Train Acc: 50.59%  Val Loss: 0.8427, Val Acc:
 ↪ 47.06%  Test Loss: 0.7915, Test Acc: 52.17%
Epoch [3/100]  Train Loss: 0.8074, Train Acc: 50.59%  Val Loss: 0.8427, Val Acc:
 ↪ 47.06%  Test Loss: 0.7915, Test Acc: 52.17%
Epoch [4/100]  Train Loss: 0.8074, Train Acc: 50.59%  Val Loss: 0.8427, Val Acc:
 ↪ 47.06%  Test Loss: 0.7915, Test Acc: 52.17%
Epoch [5/100]  Train Loss: 0.8074, Train Acc: 50.59%  Val Loss: 0.8427, Val Acc:
 ↪ 47.06%  Test Loss: 0.7915, Test Acc: 52.17%
Epoch [6/100]  Train Loss: 0.8074, Train Acc: 50.59%  Val Loss: 0.8427, Val Acc:
 ↪ 47.06%  Test Loss: 0.7915, Test Acc: 52.17%
Epoch [7/100]  Train Loss: 0.8072, Train Acc: 50.59%  Val Loss: 0.8427, Val Acc:
 ↪ 47.06%  Test Loss: 0.7909, Test Acc: 52.17%
Epoch [8/100]  Train Loss: 0.8055, Train Acc: 50.59%  Val Loss: 0.8353, Val Acc:
 ↪ 47.06%  Test Loss: 0.7848, Test Acc: 52.17%
Epoch [9/100]  Train Loss: 0.7723, Train Acc: 52.35%  Val Loss: 0.7477, Val Acc:
 ↪ 52.94%  Test Loss: 0.6937, Test Acc: 65.22%
Epoch [10/100]  Train Loss: 0.6326, Train Acc: 65.29%  Val Loss: 0.6056, Val␣
 ↪Acc: 64.71%  Test Loss: 0.6027, Test Acc: 65.22%
Epoch [11/100]  Train Loss: 0.4462, Train Acc: 88.24%  Val Loss: 0.3501, Val␣
 ↪Acc: 100.00%  Test Loss: 0.3272, Test Acc: 100.00%
```

```
Epoch [12/100]  Train Loss: 0.3948, Train Acc: 97.06%  Val Loss: 0.3392, Val␣
 ↪Acc: 100.00%  Test Loss: 0.3354, Test Acc: 100.00%
Epoch [13/100]  Train Loss: 0.4095, Train Acc: 94.12%  Val Loss: 0.3360, Val␣
 ↪Acc: 100.00%  Test Loss: 0.3353, Test Acc: 100.00%
Epoch [14/100]  Train Loss: 0.3795, Train Acc: 95.88%  Val Loss: 0.3403, Val␣
 ↪Acc: 100.00%  Test Loss: 0.3260, Test Acc: 100.00%
Epoch [15/100]  Train Loss: 0.4078, Train Acc: 92.94%  Val Loss: 0.3469, Val␣
 ↪Acc: 100.00%  Test Loss: 0.3452, Test Acc: 100.00%
Epoch [16/100]  Train Loss: 0.3924, Train Acc: 93.53%  Val Loss: 0.3453, Val␣
 ↪Acc: 100.00%  Test Loss: 0.3365, Test Acc: 100.00%
Epoch [17/100]  Train Loss: 0.3765, Train Acc: 97.06%  Val Loss: 0.3305, Val␣
 ↪Acc: 100.00%  Test Loss: 0.3302, Test Acc: 100.00%
Epoch [18/100]  Train Loss: 0.4070, Train Acc: 92.94%  Val Loss: 0.3481, Val␣
 ↪Acc: 100.00%  Test Loss: 0.3422, Test Acc: 100.00%
Epoch [19/100]  Train Loss: 0.3741, Train Acc: 94.71%  Val Loss: 0.3350, Val␣
 ↪Acc: 100.00%  Test Loss: 0.3305, Test Acc: 100.00%
Epoch [20/100]  Train Loss: 0.3808, Train Acc: 94.71%  Val Loss: 0.3290, Val␣
 ↪Acc: 100.00%  Test Loss: 0.3234, Test Acc: 100.00%
Epoch [21/100]  Train Loss: 0.3685, Train Acc: 98.24%  Val Loss: 0.3286, Val␣
 ↪Acc: 100.00%  Test Loss: 0.3194, Test Acc: 100.00%
Epoch [22/100]  Train Loss: 0.3684, Train Acc: 95.29%  Val Loss: 0.3272, Val␣
 ↪Acc: 100.00%  Test Loss: 0.3345, Test Acc: 100.00%
Epoch [23/100]  Train Loss: 0.3571, Train Acc: 98.24%  Val Loss: 0.3291, Val␣
 ↪Acc: 100.00%  Test Loss: 0.3283, Test Acc: 100.00%
Epoch [24/100]  Train Loss: 0.3597, Train Acc: 96.47%  Val Loss: 0.3315, Val␣
 ↪Acc: 100.00%  Test Loss: 0.3308, Test Acc: 100.00%
Epoch [25/100]  Train Loss: 0.3617, Train Acc: 95.88%  Val Loss: 0.3303, Val␣
 ↪Acc: 100.00%  Test Loss: 0.3188, Test Acc: 100.00%
Epoch [26/100]  Train Loss: 0.3565, Train Acc: 98.24%  Val Loss: 0.3296, Val␣
 ↪Acc: 100.00%  Test Loss: 0.3292, Test Acc: 100.00%
Epoch [27/100]  Train Loss: 0.3551, Train Acc: 98.82%  Val Loss: 0.3281, Val␣
 ↪Acc: 100.00%  Test Loss: 0.3255, Test Acc: 100.00%
Epoch [28/100]  Train Loss: 0.3709, Train Acc: 96.47%  Val Loss: 0.3255, Val␣
 ↪Acc: 100.00%  Test Loss: 0.3222, Test Acc: 100.00%
Epoch [29/100]  Train Loss: 0.3450, Train Acc: 100.00%  Val Loss: 0.3290, Val␣
 ↪Acc: 100.00%  Test Loss: 0.3222, Test Acc: 100.00%
Epoch [30/100]  Train Loss: 0.3505, Train Acc: 98.24%  Val Loss: 0.3249, Val␣
 ↪Acc: 100.00%  Test Loss: 0.3196, Test Acc: 100.00%
Epoch [31/100]  Train Loss: 0.3405, Train Acc: 100.00%  Val Loss: 0.3343, Val␣
 ↪Acc: 100.00%  Test Loss: 0.3206, Test Acc: 100.00%
Epoch [32/100]  Train Loss: 0.3623, Train Acc: 97.65%  Val Loss: 0.3255, Val␣
 ↪Acc: 100.00%  Test Loss: 0.3206, Test Acc: 100.00%
Epoch [33/100]  Train Loss: 0.3588, Train Acc: 98.82%  Val Loss: 0.3266, Val␣
 ↪Acc: 100.00%  Test Loss: 0.3266, Test Acc: 100.00%
```

```
Epoch [34/100]  Train Loss: 0.3435, Train Acc: 99.41%  Val Loss: 0.3301, Val␣
 ↪Acc: 100.00%  Test Loss: 0.3183, Test Acc: 100.00%
Epoch [35/100]  Train Loss: 0.3488, Train Acc: 98.82%  Val Loss: 0.3216, Val␣
 ↪Acc: 100.00%  Test Loss: 0.3166, Test Acc: 100.00%
Epoch [36/100]  Train Loss: 0.3556, Train Acc: 97.06%  Val Loss: 0.3275, Val␣
 ↪Acc: 100.00%  Test Loss: 0.3228, Test Acc: 100.00%
Epoch [37/100]  Train Loss: 0.3612, Train Acc: 96.47%  Val Loss: 0.3322, Val␣
 ↪Acc: 100.00%  Test Loss: 0.3290, Test Acc: 100.00%
Epoch [38/100]  Train Loss: 0.3634, Train Acc: 98.24%  Val Loss: 0.3234, Val␣
 ↪Acc: 100.00%  Test Loss: 0.3215, Test Acc: 100.00%
Epoch [39/100]  Train Loss: 0.3482, Train Acc: 98.24%  Val Loss: 0.3258, Val␣
 ↪Acc: 100.00%  Test Loss: 0.3208, Test Acc: 100.00%
Epoch [40/100]  Train Loss: 0.3551, Train Acc: 99.41%  Val Loss: 0.3216, Val␣
 ↪Acc: 100.00%  Test Loss: 0.3238, Test Acc: 100.00%
Epoch [41/100]  Train Loss: 0.3580, Train Acc: 97.06%  Val Loss: 0.3210, Val␣
 ↪Acc: 100.00%  Test Loss: 0.3193, Test Acc: 100.00%
Epoch [42/100]  Train Loss: 0.3575, Train Acc: 98.24%  Val Loss: 0.3242, Val␣
 ↪Acc: 100.00%  Test Loss: 0.3222, Test Acc: 100.00%
Epoch [43/100]  Train Loss: 0.3506, Train Acc: 97.06%  Val Loss: 0.3221, Val␣
 ↪Acc: 100.00%  Test Loss: 0.3198, Test Acc: 100.00%
Epoch [44/100]  Train Loss: 0.3587, Train Acc: 97.06%  Val Loss: 0.3274, Val␣
 ↪Acc: 100.00%  Test Loss: 0.3196, Test Acc: 100.00%
Epoch [45/100]  Train Loss: 0.3522, Train Acc: 97.06%  Val Loss: 0.3236, Val␣
 ↪Acc: 100.00%  Test Loss: 0.3207, Test Acc: 100.00%
Epoch [46/100]  Train Loss: 0.3455, Train Acc: 99.41%  Val Loss: 0.3223, Val␣
 ↪Acc: 100.00%  Test Loss: 0.3147, Test Acc: 100.00%
Epoch [47/100]  Train Loss: 0.3404, Train Acc: 99.41%  Val Loss: 0.3294, Val␣
 ↪Acc: 100.00%  Test Loss: 0.3281, Test Acc: 100.00%
Epoch [48/100]  Train Loss: 0.3471, Train Acc: 98.82%  Val Loss: 0.3234, Val␣
 ↪Acc: 100.00%  Test Loss: 0.3220, Test Acc: 100.00%
Epoch [49/100]  Train Loss: 0.3422, Train Acc: 98.82%  Val Loss: 0.3246, Val␣
 ↪Acc: 100.00%  Test Loss: 0.3222, Test Acc: 100.00%
Epoch [50/100]  Train Loss: 0.3332, Train Acc: 100.00%  Val Loss: 0.3268, Val␣
 ↪Acc: 100.00%  Test Loss: 0.3246, Test Acc: 100.00%
Epoch [51/100]  Train Loss: 0.3527, Train Acc: 98.24%  Val Loss: 0.3217, Val␣
 ↪Acc: 100.00%  Test Loss: 0.3181, Test Acc: 100.00%
Epoch [52/100]  Train Loss: 0.3422, Train Acc: 99.41%  Val Loss: 0.3218, Val␣
 ↪Acc: 100.00%  Test Loss: 0.3168, Test Acc: 100.00%
Epoch [53/100]  Train Loss: 0.3395, Train Acc: 99.41%  Val Loss: 0.3278, Val␣
 ↪Acc: 100.00%  Test Loss: 0.3183, Test Acc: 100.00%
Epoch [54/100]  Train Loss: 0.3459, Train Acc: 98.82%  Val Loss: 0.3213, Val␣
 ↪Acc: 100.00%  Test Loss: 0.3156, Test Acc: 100.00%
Epoch [55/100]  Train Loss: 0.3493, Train Acc: 100.00%  Val Loss: 0.3251, Val␣
 ↪Acc: 100.00%  Test Loss: 0.3216, Test Acc: 100.00%
```

```
Epoch [56/100]  Train Loss: 0.3439, Train Acc: 98.82%  Val Loss: 0.3239, Val␣
 ↪Acc: 100.00%  Test Loss: 0.3188, Test Acc: 100.00%
Epoch [57/100]  Train Loss: 0.3446, Train Acc: 99.41%  Val Loss: 0.3264, Val␣
 ↪Acc: 100.00%  Test Loss: 0.3234, Test Acc: 100.00%
Epoch [58/100]  Train Loss: 0.3423, Train Acc: 98.82%  Val Loss: 0.3232, Val␣
 ↪Acc: 100.00%  Test Loss: 0.3232, Test Acc: 100.00%
Epoch [59/100]  Train Loss: 0.3394, Train Acc: 98.82%  Val Loss: 0.3240, Val␣
 ↪Acc: 100.00%  Test Loss: 0.3212, Test Acc: 100.00%
Epoch [60/100]  Train Loss: 0.3367, Train Acc: 99.41%  Val Loss: 0.3211, Val␣
 ↪Acc: 100.00%  Test Loss: 0.3225, Test Acc: 100.00%
Epoch [61/100]  Train Loss: 0.3505, Train Acc: 97.65%  Val Loss: 0.3249, Val␣
 ↪Acc: 100.00%  Test Loss: 0.3217, Test Acc: 100.00%
Epoch [62/100]  Train Loss: 0.3460, Train Acc: 98.82%  Val Loss: 0.3281, Val␣
 ↪Acc: 100.00%  Test Loss: 0.3263, Test Acc: 100.00%
Epoch [63/100]  Train Loss: 0.3491, Train Acc: 98.82%  Val Loss: 0.3208, Val␣
 ↪Acc: 100.00%  Test Loss: 0.3198, Test Acc: 100.00%
Epoch [64/100]  Train Loss: 0.3401, Train Acc: 99.41%  Val Loss: 0.3198, Val␣
 ↪Acc: 100.00%  Test Loss: 0.3176, Test Acc: 100.00%
Epoch [65/100]  Train Loss: 0.3360, Train Acc: 100.00%  Val Loss: 0.3211, Val␣
 ↪Acc: 100.00%  Test Loss: 0.3169, Test Acc: 100.00%
Epoch [66/100]  Train Loss: 0.3391, Train Acc: 100.00%  Val Loss: 0.3264, Val␣
 ↪Acc: 100.00%  Test Loss: 0.3180, Test Acc: 100.00%
Epoch [67/100]  Train Loss: 0.3427, Train Acc: 98.82%  Val Loss: 0.3188, Val␣
 ↪Acc: 100.00%  Test Loss: 0.3166, Test Acc: 100.00%
Epoch [68/100]  Train Loss: 0.3532, Train Acc: 98.24%  Val Loss: 0.3185, Val␣
 ↪Acc: 100.00%  Test Loss: 0.3171, Test Acc: 100.00%
Epoch [69/100]  Train Loss: 0.3426, Train Acc: 99.41%  Val Loss: 0.3185, Val␣
 ↪Acc: 100.00%  Test Loss: 0.3208, Test Acc: 100.00%
Epoch [70/100]  Train Loss: 0.3401, Train Acc: 99.41%  Val Loss: 0.3231, Val␣
 ↪Acc: 100.00%  Test Loss: 0.3213, Test Acc: 100.00%
Epoch [71/100]  Train Loss: 0.3424, Train Acc: 100.00%  Val Loss: 0.3156, Val␣
 ↪Acc: 100.00%  Test Loss: 0.3156, Test Acc: 100.00%
Epoch [72/100]  Train Loss: 0.3459, Train Acc: 99.41%  Val Loss: 0.3241, Val␣
 ↪Acc: 100.00%  Test Loss: 0.3164, Test Acc: 100.00%
Epoch [73/100]  Train Loss: 0.3569, Train Acc: 97.06%  Val Loss: 0.3187, Val␣
 ↪Acc: 100.00%  Test Loss: 0.3171, Test Acc: 100.00%
Epoch [74/100]  Train Loss: 0.3401, Train Acc: 100.00%  Val Loss: 0.3221, Val␣
 ↪Acc: 100.00%  Test Loss: 0.3205, Test Acc: 100.00%
Epoch [75/100]  Train Loss: 0.3424, Train Acc: 99.41%  Val Loss: 0.3204, Val␣
 ↪Acc: 100.00%  Test Loss: 0.3198, Test Acc: 100.00%
Epoch [76/100]  Train Loss: 0.3398, Train Acc: 100.00%  Val Loss: 0.3215, Val␣
 ↪Acc: 100.00%  Test Loss: 0.3253, Test Acc: 100.00%
Epoch [77/100]  Train Loss: 0.3406, Train Acc: 99.41%  Val Loss: 0.3201, Val␣
 ↪Acc: 100.00%  Test Loss: 0.3164, Test Acc: 100.00%
```

```
Epoch [78/100]  Train Loss: 0.3411, Train Acc: 100.00%  Val Loss: 0.3174, Val␣
 ↪Acc: 100.00%  Test Loss: 0.3185, Test Acc: 100.00%
Epoch [79/100]  Train Loss: 0.3411, Train Acc: 100.00%  Val Loss: 0.3178, Val␣
 ↪Acc: 100.00%  Test Loss: 0.3159, Test Acc: 100.00%
Epoch [80/100]  Train Loss: 0.3486, Train Acc: 98.82%  Val Loss: 0.3169, Val␣
 ↪Acc: 100.00%  Test Loss: 0.3147, Test Acc: 100.00%
Epoch [81/100]  Train Loss: 0.3452, Train Acc: 98.82%  Val Loss: 0.3194, Val␣
 ↪Acc: 100.00%  Test Loss: 0.3203, Test Acc: 100.00%
Epoch [82/100]  Train Loss: 0.3491, Train Acc: 98.82%  Val Loss: 0.3181, Val␣
 ↪Acc: 100.00%  Test Loss: 0.3168, Test Acc: 100.00%
Epoch [83/100]  Train Loss: 0.3394, Train Acc: 98.82%  Val Loss: 0.3239, Val␣
 ↪Acc: 100.00%  Test Loss: 0.3220, Test Acc: 100.00%
Epoch [84/100]  Train Loss: 0.3420, Train Acc: 98.82%  Val Loss: 0.3188, Val␣
 ↪Acc: 100.00%  Test Loss: 0.3171, Test Acc: 100.00%
Epoch [85/100]  Train Loss: 0.3362, Train Acc: 100.00%  Val Loss: 0.3228, Val␣
 ↪Acc: 100.00%  Test Loss: 0.3240, Test Acc: 100.00%
Epoch [86/100]  Train Loss: 0.3463, Train Acc: 98.24%  Val Loss: 0.3210, Val␣
 ↪Acc: 100.00%  Test Loss: 0.3183, Test Acc: 100.00%
Epoch [87/100]  Train Loss: 0.3488, Train Acc: 97.65%  Val Loss: 0.3195, Val␣
 ↪Acc: 100.00%  Test Loss: 0.3159, Test Acc: 100.00%
Epoch [88/100]  Train Loss: 0.3411, Train Acc: 100.00%  Val Loss: 0.3179, Val␣
 ↪Acc: 100.00%  Test Loss: 0.3186, Test Acc: 100.00%
Epoch [89/100]  Train Loss: 0.3446, Train Acc: 99.41%  Val Loss: 0.3201, Val␣
 ↪Acc: 100.00%  Test Loss: 0.3176, Test Acc: 100.00%
Epoch [90/100]  Train Loss: 0.3443, Train Acc: 98.82%  Val Loss: 0.3183, Val␣
 ↪Acc: 100.00%  Test Loss: 0.3173, Test Acc: 100.00%
Epoch [91/100]  Train Loss: 0.3417, Train Acc: 98.24%  Val Loss: 0.3197, Val␣
 ↪Acc: 100.00%  Test Loss: 0.3166, Test Acc: 100.00%
Epoch [92/100]  Train Loss: 0.3448, Train Acc: 98.82%  Val Loss: 0.3198, Val␣
 ↪Acc: 100.00%  Test Loss: 0.3178, Test Acc: 100.00%
Epoch [93/100]  Train Loss: 0.3369, Train Acc: 100.00%  Val Loss: 0.3171, Val␣
 ↪Acc: 100.00%  Test Loss: 0.3190, Test Acc: 100.00%
Epoch [94/100]  Train Loss: 0.3406, Train Acc: 99.41%  Val Loss: 0.3165, Val␣
 ↪Acc: 100.00%  Test Loss: 0.3149, Test Acc: 100.00%
Epoch [95/100]  Train Loss: 0.3481, Train Acc: 98.82%  Val Loss: 0.3152, Val␣
 ↪Acc: 100.00%  Test Loss: 0.3142, Test Acc: 100.00%
Epoch [96/100]  Train Loss: 0.3432, Train Acc: 100.00%  Val Loss: 0.3174, Val␣
 ↪Acc: 100.00%  Test Loss: 0.3149, Test Acc: 100.00%
Epoch [97/100]  Train Loss: 0.3346, Train Acc: 100.00%  Val Loss: 0.3181, Val␣
 ↪Acc: 100.00%  Test Loss: 0.3152, Test Acc: 100.00%
Epoch [98/100]  Train Loss: 0.3425, Train Acc: 99.41%  Val Loss: 0.3168, Val␣
 ↪Acc: 100.00%  Test Loss: 0.3149, Test Acc: 100.00%
Epoch [99/100]  Train Loss: 0.3413, Train Acc: 99.41%  Val Loss: 0.3152, Val␣
 ↪Acc: 100.00%  Test Loss: 0.3135, Test Acc: 100.00%
```

```
Epoch [100/100]  Train Loss: 0.3327, Train Acc: 100.00%  Val Loss: 0.3207, Val␣
 ↪Acc: 100.00%  Test Loss: 0.3161, Test Acc: 100.00%

## test 4. No Quantvolution (classic decision layer) + Quantfilter 2x2
--- Overall Metrics ---
Train Loss: Mean = 1.2719, Std = 0.1111
Train Accuracy: Mean = 36.95%, Std = 7.54%
Validation Loss: Mean = 1.3985, Std = 0.0278
Validation Accuracy: Mean = 30.77%, Std = 5.18%
Test Loss: Mean = 1.4083, Std = 0.0210
Test Accuracy: Mean = 22.03%, Std = 3.74%
Total Computation Time: 7.59 seconds

Epoch [1/100] Train Loss: 1.3980, Train Acc: 24.10%  Val Loss: 1.3976, Val Acc:␣
 ↪22.73%  Test Loss: 1.3967, Test Acc: 24.14%
Epoch [2/100] Train Loss: 1.3987, Train Acc: 24.10%  Val Loss: 1.3943, Val Acc:␣
 ↪22.73%  Test Loss: 1.3963, Test Acc: 24.14%
Epoch [3/100] Train Loss: 1.3980, Train Acc: 24.10%  Val Loss: 1.3934, Val Acc:␣
 ↪22.73%  Test Loss: 1.3955, Test Acc: 24.14%
Epoch [4/100] Train Loss: 1.3949, Train Acc: 24.62%  Val Loss: 1.3930, Val Acc:␣
 ↪22.73%  Test Loss: 1.3947, Test Acc: 24.14%
Epoch [5/100] Train Loss: 1.3946, Train Acc: 25.13%  Val Loss: 1.3922, Val Acc:␣
 ↪22.73%  Test Loss: 1.3935, Test Acc: 24.14%
Epoch [6/100] Train Loss: 1.3951, Train Acc: 23.59%  Val Loss: 1.3925, Val Acc:␣
 ↪22.73%  Test Loss: 1.3930, Test Acc: 24.14%
Epoch [7/100] Train Loss: 1.3946, Train Acc: 24.10%  Val Loss: 1.3920, Val Acc:␣
 ↪22.73%  Test Loss: 1.3928, Test Acc: 24.14%
Epoch [8/100] Train Loss: 1.3911, Train Acc: 25.64%  Val Loss: 1.3916, Val Acc:␣
 ↪22.73%  Test Loss: 1.3917, Test Acc: 24.14%
Epoch [9/100] Train Loss: 1.3933, Train Acc: 25.13%  Val Loss: 1.3916, Val Acc:␣
 ↪22.73%  Test Loss: 1.3917, Test Acc: 24.14%
Epoch [10/100] Train Loss: 1.3910, Train Acc: 26.67%  Val Loss: 1.3901, Val Acc:
 ↪ 22.73%  Test Loss: 1.3918, Test Acc: 24.14%
Epoch [11/100] Train Loss: 1.3911, Train Acc: 26.15%  Val Loss: 1.3891, Val Acc:
 ↪ 22.73%  Test Loss: 1.3920, Test Acc: 24.14%
Epoch [12/100] Train Loss: 1.3898, Train Acc: 27.69%  Val Loss: 1.3879, Val Acc:
 ↪ 22.73%  Test Loss: 1.3924, Test Acc: 24.14%
Epoch [13/100] Train Loss: 1.3888, Train Acc: 28.72%  Val Loss: 1.3858, Val Acc:
 ↪ 22.73%  Test Loss: 1.3928, Test Acc: 24.14%
Epoch [14/100] Train Loss: 1.3912, Train Acc: 28.72%  Val Loss: 1.3859, Val Acc:
 ↪ 22.73%  Test Loss: 1.3941, Test Acc: 20.69%
Epoch [15/100] Train Loss: 1.3874, Train Acc: 28.21%  Val Loss: 1.3862, Val Acc:
 ↪ 22.73%  Test Loss: 1.3946, Test Acc: 17.24%
Epoch [16/100] Train Loss: 1.3912, Train Acc: 26.67%  Val Loss: 1.3875, Val Acc:
 ↪ 22.73%  Test Loss: 1.3922, Test Acc: 17.24%
```

```
Epoch [17/100] Train Loss: 1.3881, Train Acc: 27.18%  Val Loss: 1.3868, Val Acc:
 ↪ 22.73%  Test Loss: 1.3912, Test Acc: 20.69%
Epoch [18/100] Train Loss: 1.3857, Train Acc: 29.74%  Val Loss: 1.3853, Val Acc:
 ↪ 31.82%  Test Loss: 1.3909, Test Acc: 17.24%
Epoch [19/100] Train Loss: 1.3846, Train Acc: 30.26%  Val Loss: 1.3862, Val Acc:
 ↪ 27.27%  Test Loss: 1.3901, Test Acc: 20.69%
Epoch [20/100] Train Loss: 1.3883, Train Acc: 24.10%  Val Loss: 1.3846, Val Acc:
 ↪ 31.82%  Test Loss: 1.3910, Test Acc: 20.69%
Epoch [21/100] Train Loss: 1.3808, Train Acc: 31.79%  Val Loss: 1.3838, Val Acc:
 ↪ 31.82%  Test Loss: 1.3919, Test Acc: 24.14%
Epoch [22/100] Train Loss: 1.3833, Train Acc: 28.21%  Val Loss: 1.3830, Val Acc:
 ↪ 31.82%  Test Loss: 1.3934, Test Acc: 20.69%
Epoch [23/100] Train Loss: 1.3757, Train Acc: 33.85%  Val Loss: 1.3785, Val Acc:
 ↪ 36.36%  Test Loss: 1.3948, Test Acc: 20.69%
Epoch [24/100] Train Loss: 1.3783, Train Acc: 30.77%  Val Loss: 1.3772, Val Acc:
 ↪ 31.82%  Test Loss: 1.3906, Test Acc: 24.14%
Epoch [25/100] Train Loss: 1.3832, Train Acc: 34.36%  Val Loss: 1.3781, Val Acc:
 ↪ 31.82%  Test Loss: 1.3910, Test Acc: 20.69%
Epoch [26/100] Train Loss: 1.3796, Train Acc: 32.31%  Val Loss: 1.3800, Val Acc:
 ↪ 31.82%  Test Loss: 1.3938, Test Acc: 20.69%
Epoch [27/100] Train Loss: 1.3789, Train Acc: 30.77%  Val Loss: 1.3793, Val Acc:
 ↪ 31.82%  Test Loss: 1.3937, Test Acc: 24.14%
Epoch [28/100] Train Loss: 1.3691, Train Acc: 30.77%  Val Loss: 1.3787, Val Acc:
 ↪ 40.91%  Test Loss: 1.3941, Test Acc: 24.14%
Epoch [29/100] Train Loss: 1.3719, Train Acc: 31.79%  Val Loss: 1.3751, Val Acc:
 ↪ 36.36%  Test Loss: 1.3968, Test Acc: 24.14%
Epoch [30/100] Train Loss: 1.3782, Train Acc: 31.28%  Val Loss: 1.3779, Val Acc:
 ↪ 31.82%  Test Loss: 1.3920, Test Acc: 27.59%
Epoch [31/100] Train Loss: 1.3757, Train Acc: 28.21%  Val Loss: 1.3735, Val Acc:
 ↪ 31.82%  Test Loss: 1.3957, Test Acc: 24.14%
Epoch [32/100] Train Loss: 1.3763, Train Acc: 29.74%  Val Loss: 1.3721, Val Acc:
 ↪ 31.82%  Test Loss: 1.3934, Test Acc: 27.59%
Epoch [33/100] Train Loss: 1.3543, Train Acc: 34.87%  Val Loss: 1.3767, Val Acc:
 ↪ 31.82%  Test Loss: 1.3919, Test Acc: 24.14%
Epoch [34/100] Train Loss: 1.3546, Train Acc: 33.85%  Val Loss: 1.3697, Val Acc:
 ↪ 36.36%  Test Loss: 1.3984, Test Acc: 24.14%
Epoch [35/100] Train Loss: 1.3488, Train Acc: 34.36%  Val Loss: 1.3732, Val Acc:
 ↪ 36.36%  Test Loss: 1.3953, Test Acc: 24.14%
Epoch [36/100] Train Loss: 1.3635, Train Acc: 29.23%  Val Loss: 1.3728, Val Acc:
 ↪ 40.91%  Test Loss: 1.3960, Test Acc: 27.59%
Epoch [37/100] Train Loss: 1.3553, Train Acc: 31.79%  Val Loss: 1.3747, Val Acc:
 ↪ 40.91%  Test Loss: 1.3959, Test Acc: 24.14%
Epoch [38/100] Train Loss: 1.3469, Train Acc: 35.90%  Val Loss: 1.3741, Val Acc:
 ↪ 40.91%  Test Loss: 1.3989, Test Acc: 27.59%
```

```
Epoch [39/100] Train Loss: 1.3484, Train Acc: 33.33%  Val Loss: 1.3726, Val Acc:
 ↪ 40.91%  Test Loss: 1.3991, Test Acc: 24.14%
Epoch [40/100] Train Loss: 1.3462, Train Acc: 33.85%  Val Loss: 1.3759, Val Acc:
 ↪ 31.82%  Test Loss: 1.3981, Test Acc: 27.59%
Epoch [41/100] Train Loss: 1.3414, Train Acc: 37.44%  Val Loss: 1.3812, Val Acc:
 ↪ 31.82%  Test Loss: 1.3959, Test Acc: 20.69%
Epoch [42/100] Train Loss: 1.3355, Train Acc: 37.44%  Val Loss: 1.3751, Val Acc:
 ↪ 36.36%  Test Loss: 1.3921, Test Acc: 20.69%
Epoch [43/100] Train Loss: 1.3422, Train Acc: 33.33%  Val Loss: 1.3777, Val Acc:
 ↪ 31.82%  Test Loss: 1.3906, Test Acc: 20.69%
Epoch [44/100] Train Loss: 1.3254, Train Acc: 36.41%  Val Loss: 1.3789, Val Acc:
 ↪ 31.82%  Test Loss: 1.3869, Test Acc: 20.69%
Epoch [45/100] Train Loss: 1.3300, Train Acc: 33.33%  Val Loss: 1.3815, Val Acc:
 ↪ 31.82%  Test Loss: 1.3813, Test Acc: 27.59%
Epoch [46/100] Train Loss: 1.3138, Train Acc: 38.97%  Val Loss: 1.3770, Val Acc:
 ↪ 31.82%  Test Loss: 1.3947, Test Acc: 27.59%
Epoch [47/100] Train Loss: 1.3158, Train Acc: 38.46%  Val Loss: 1.3733, Val Acc:
 ↪ 31.82%  Test Loss: 1.4065, Test Acc: 20.69%
Epoch [48/100] Train Loss: 1.2976, Train Acc: 37.95%  Val Loss: 1.3773, Val Acc:
 ↪ 36.36%  Test Loss: 1.4106, Test Acc: 24.14%
Epoch [49/100] Train Loss: 1.3274, Train Acc: 36.92%  Val Loss: 1.3833, Val Acc:
 ↪ 36.36%  Test Loss: 1.4078, Test Acc: 20.69%
Epoch [50/100] Train Loss: 1.3101, Train Acc: 38.46%  Val Loss: 1.3785, Val Acc:
 ↪ 36.36%  Test Loss: 1.4044, Test Acc: 31.03%
Epoch [51/100] Train Loss: 1.2904, Train Acc: 39.49%  Val Loss: 1.3842, Val Acc:
 ↪ 31.82%  Test Loss: 1.4072, Test Acc: 27.59%
Epoch [52/100] Train Loss: 1.2604, Train Acc: 41.54%  Val Loss: 1.3759, Val Acc:
 ↪ 36.36%  Test Loss: 1.3993, Test Acc: 24.14%
Epoch [53/100] Train Loss: 1.2635, Train Acc: 37.44%  Val Loss: 1.3851, Val Acc:
 ↪ 36.36%  Test Loss: 1.4086, Test Acc: 27.59%
Epoch [54/100] Train Loss: 1.2611, Train Acc: 44.10%  Val Loss: 1.4166, Val Acc:
 ↪ 31.82%  Test Loss: 1.3998, Test Acc: 24.14%
Epoch [55/100] Train Loss: 1.2977, Train Acc: 37.44%  Val Loss: 1.4149, Val Acc:
 ↪ 31.82%  Test Loss: 1.3933, Test Acc: 20.69%
Epoch [56/100] Train Loss: 1.2847, Train Acc: 35.90%  Val Loss: 1.3991, Val Acc:
 ↪ 31.82%  Test Loss: 1.3936, Test Acc: 27.59%
Epoch [57/100] Train Loss: 1.2816, Train Acc: 38.97%  Val Loss: 1.4029, Val Acc:
 ↪ 31.82%  Test Loss: 1.3999, Test Acc: 27.59%
Epoch [58/100] Train Loss: 1.2713, Train Acc: 35.90%  Val Loss: 1.4009, Val Acc:
 ↪ 31.82%  Test Loss: 1.4049, Test Acc: 20.69%
Epoch [59/100] Train Loss: 1.2726, Train Acc: 36.92%  Val Loss: 1.4026, Val Acc:
 ↪ 27.27%  Test Loss: 1.4042, Test Acc: 24.14%
Epoch [60/100] Train Loss: 1.2425, Train Acc: 41.03%  Val Loss: 1.3895, Val Acc:
 ↪ 31.82%  Test Loss: 1.4027, Test Acc: 27.59%
```

```
Epoch [61/100] Train Loss: 1.2521, Train Acc: 38.97%  Val Loss: 1.4105, Val Acc:
 ↪ 31.82%  Test Loss: 1.3987, Test Acc: 24.14%
Epoch [62/100] Train Loss: 1.2476, Train Acc: 41.54%  Val Loss: 1.4364, Val Acc:
 ↪ 31.82%  Test Loss: 1.4028, Test Acc: 24.14%
Epoch [63/100] Train Loss: 1.2391, Train Acc: 39.49%  Val Loss: 1.4477, Val Acc:
 ↪ 27.27%  Test Loss: 1.4057, Test Acc: 24.14%
Epoch [64/100] Train Loss: 1.2363, Train Acc: 40.51%  Val Loss: 1.4331, Val Acc:
 ↪ 27.27%  Test Loss: 1.3967, Test Acc: 17.24%
Epoch [65/100] Train Loss: 1.1815, Train Acc: 42.56%  Val Loss: 1.4144, Val Acc:
 ↪ 27.27%  Test Loss: 1.3932, Test Acc: 20.69%
Epoch [66/100] Train Loss: 1.2271, Train Acc: 41.03%  Val Loss: 1.4024, Val Acc:
 ↪ 31.82%  Test Loss: 1.3957, Test Acc: 20.69%
Epoch [67/100] Train Loss: 1.2126, Train Acc: 44.62%  Val Loss: 1.4437, Val Acc:
 ↪ 27.27%  Test Loss: 1.4161, Test Acc: 17.24%
Epoch [68/100] Train Loss: 1.2321, Train Acc: 38.97%  Val Loss: 1.4079, Val Acc:
 ↪ 27.27%  Test Loss: 1.4140, Test Acc: 17.24%
Epoch [69/100] Train Loss: 1.2084, Train Acc: 43.59%  Val Loss: 1.4192, Val Acc:
 ↪ 27.27%  Test Loss: 1.4214, Test Acc: 20.69%
Epoch [70/100] Train Loss: 1.2036, Train Acc: 38.97%  Val Loss: 1.4539, Val Acc:
 ↪ 27.27%  Test Loss: 1.4182, Test Acc: 20.69%
Epoch [71/100] Train Loss: 1.2102, Train Acc: 38.46%  Val Loss: 1.4371, Val Acc:
 ↪ 27.27%  Test Loss: 1.4393, Test Acc: 17.24%
Epoch [72/100] Train Loss: 1.1748, Train Acc: 43.59%  Val Loss: 1.4646, Val Acc:
 ↪ 31.82%  Test Loss: 1.4546, Test Acc: 17.24%
Epoch [73/100] Train Loss: 1.2339, Train Acc: 41.54%  Val Loss: 1.4489, Val Acc:
 ↪ 27.27%  Test Loss: 1.4244, Test Acc: 24.14%
Epoch [74/100] Train Loss: 1.2043, Train Acc: 42.56%  Val Loss: 1.4647, Val Acc:
 ↪ 27.27%  Test Loss: 1.4276, Test Acc: 20.69%
Epoch [75/100] Train Loss: 1.1835, Train Acc: 43.59%  Val Loss: 1.4769, Val Acc:
 ↪ 27.27%  Test Loss: 1.4379, Test Acc: 20.69%
Epoch [76/100] Train Loss: 1.1379, Train Acc: 44.62%  Val Loss: 1.4948, Val Acc:
 ↪ 31.82%  Test Loss: 1.4413, Test Acc: 24.14%
Epoch [77/100] Train Loss: 1.1579, Train Acc: 42.56%  Val Loss: 1.4823, Val Acc:
 ↪ 31.82%  Test Loss: 1.4513, Test Acc: 13.79%
Epoch [78/100] Train Loss: 1.1318, Train Acc: 46.15%  Val Loss: 1.4662, Val Acc:
 ↪ 31.82%  Test Loss: 1.4581, Test Acc: 13.79%
Epoch [79/100] Train Loss: 1.1500, Train Acc: 46.15%  Val Loss: 1.4218, Val Acc:
 ↪ 27.27%  Test Loss: 1.4548, Test Acc: 20.69%
Epoch [80/100] Train Loss: 1.1793, Train Acc: 41.03%  Val Loss: 1.4250, Val Acc:
 ↪ 31.82%  Test Loss: 1.4410, Test Acc: 24.14%
Epoch [81/100] Train Loss: 1.1455, Train Acc: 44.62%  Val Loss: 1.4215, Val Acc:
 ↪ 36.36%  Test Loss: 1.4066, Test Acc: 24.14%
Epoch [82/100] Train Loss: 1.1322, Train Acc: 44.62%  Val Loss: 1.4023, Val Acc:
 ↪ 22.73%  Test Loss: 1.4298, Test Acc: 24.14%
```

```
Epoch [83/100] Train Loss: 1.0964, Train Acc: 46.67%  Val Loss: 1.3931, Val Acc:
 ↪ 36.36%  Test Loss: 1.4382, Test Acc: 24.14%
Epoch [84/100] Train Loss: 1.1714, Train Acc: 41.54%  Val Loss: 1.3967, Val Acc:
 ↪ 31.82%  Test Loss: 1.4268, Test Acc: 24.14%
Epoch [85/100] Train Loss: 1.0901, Train Acc: 49.23%  Val Loss: 1.4019, Val Acc:
 ↪ 31.82%  Test Loss: 1.4520, Test Acc: 20.69%
Epoch [86/100] Train Loss: 1.0947, Train Acc: 44.62%  Val Loss: 1.3894, Val Acc:
 ↪ 31.82%  Test Loss: 1.4509, Test Acc: 17.24%
Epoch [87/100] Train Loss: 1.1255, Train Acc: 47.18%  Val Loss: 1.3565, Val Acc:
 ↪ 36.36%  Test Loss: 1.4319, Test Acc: 20.69%
Epoch [88/100] Train Loss: 1.0936, Train Acc: 45.13%  Val Loss: 1.4100, Val Acc:
 ↪ 31.82%  Test Loss: 1.4144, Test Acc: 24.14%
Epoch [89/100] Train Loss: 1.1240, Train Acc: 47.69%  Val Loss: 1.4177, Val Acc:
 ↪ 36.36%  Test Loss: 1.3953, Test Acc: 20.69%
Epoch [90/100] Train Loss: 1.0426, Train Acc: 49.23%  Val Loss: 1.3850, Val Acc:
 ↪ 36.36%  Test Loss: 1.4284, Test Acc: 13.79%
Epoch [91/100] Train Loss: 1.1550, Train Acc: 45.13%  Val Loss: 1.3626, Val Acc:
 ↪ 40.91%  Test Loss: 1.4061, Test Acc: 17.24%
Epoch [92/100] Train Loss: 1.1016, Train Acc: 46.67%  Val Loss: 1.3970, Val Acc:
 ↪ 40.91%  Test Loss: 1.3894, Test Acc: 17.24%
Epoch [93/100] Train Loss: 1.1090, Train Acc: 49.23%  Val Loss: 1.3967, Val Acc:
 ↪ 31.82%  Test Loss: 1.4492, Test Acc: 13.79%
Epoch [94/100] Train Loss: 1.1007, Train Acc: 50.26%  Val Loss: 1.4055, Val Acc:
 ↪ 36.36%  Test Loss: 1.4097, Test Acc: 17.24%
Epoch [95/100] Train Loss: 1.0987, Train Acc: 46.67%  Val Loss: 1.4123, Val Acc:
 ↪ 31.82%  Test Loss: 1.4653, Test Acc: 13.79%
Epoch [96/100] Train Loss: 1.0483, Train Acc: 46.67%  Val Loss: 1.4254, Val Acc:
 ↪ 22.73%  Test Loss: 1.4276, Test Acc: 17.24%
Epoch [97/100] Train Loss: 1.1140, Train Acc: 41.03%  Val Loss: 1.3974, Val Acc:
 ↪ 31.82%  Test Loss: 1.4315, Test Acc: 17.24%
Epoch [98/100] Train Loss: 1.0691, Train Acc: 49.23%  Val Loss: 1.4108, Val Acc:
 ↪ 31.82%  Test Loss: 1.4412, Test Acc: 17.24%
Epoch [99/100] Train Loss: 1.0719, Train Acc: 48.72%  Val Loss: 1.3792, Val Acc:
 ↪ 31.82%  Test Loss: 1.4305, Test Acc: 17.24%
Epoch [100/100] Train Loss: 1.0516, Train Acc: 47.18%  Val Loss: 1.3596, Val␣
 ↪Acc: 27.27%  Test Loss: 1.4724, Test Acc: 17.24%

## test 5. No Quantvolution (classic decision layer) + Quantfilter 4x4
Train Loss: Mean = 0.0692, Std = 0.2347
Train Accuracy: Mean = 97.72%, Std = 9.87%
Validation Loss: Mean = 3.4513, Std = 0.7994
Validation Accuracy: Mean = 20.82%, Std = 5.42%
Test Loss: Mean = 3.5621, Std = 0.8763
Test Accuracy: Mean = 20.72%, Std = 4.46%
Total Computation Time: 4.45 seconds
```

```
Epoch [1/100] Train Loss: 1.4040, Train Acc: 27.69%  Val Loss: 1.4170, Val Acc:␣
↪27.27%  Test Loss: 1.3980, Test Acc: 20.69%
Epoch [2/100] Train Loss: 1.2531, Train Acc: 45.64%  Val Loss: 1.5064, Val Acc:␣
↪27.27%  Test Loss: 1.4198, Test Acc: 27.59%
Epoch [3/100] Train Loss: 1.0767, Train Acc: 64.62%  Val Loss: 1.4745, Val Acc:␣
↪18.18%  Test Loss: 1.3941, Test Acc: 27.59%
Epoch [4/100] Train Loss: 0.7589, Train Acc: 82.56%  Val Loss: 1.6364, Val Acc:␣
↪18.18%  Test Loss: 1.4956, Test Acc: 27.59%
Epoch [5/100] Train Loss: 0.4750, Train Acc: 89.74%  Val Loss: 1.7303, Val Acc:␣
↪18.18%  Test Loss: 1.6097, Test Acc: 27.59%
Epoch [6/100] Train Loss: 0.2713, Train Acc: 96.92%  Val Loss: 1.8265, Val Acc:␣
↪22.73%  Test Loss: 1.7612, Test Acc: 17.24%
Epoch [7/100] Train Loss: 0.1255, Train Acc: 98.97%  Val Loss: 2.0678, Val Acc:␣
↪18.18%  Test Loss: 1.8849, Test Acc: 20.69%
Epoch [8/100] Train Loss: 0.0836, Train Acc: 98.46%  Val Loss: 2.1271, Val Acc:␣
↪18.18%  Test Loss: 1.9919, Test Acc: 20.69%
Epoch [9/100] Train Loss: 0.0547, Train Acc: 100.00%  Val Loss: 2.1080, Val Acc:
↪ 27.27%  Test Loss: 2.0895, Test Acc: 24.14%
Epoch [10/100] Train Loss: 0.0299, Train Acc: 100.00%  Val Loss: 2.3306, Val␣
↪Acc: 27.27%  Test Loss: 2.2433, Test Acc: 24.14%
Epoch [11/100] Train Loss: 0.0269, Train Acc: 100.00%  Val Loss: 2.3920, Val␣
↪Acc: 27.27%  Test Loss: 2.2241, Test Acc: 27.59%
Epoch [12/100] Train Loss: 0.0226, Train Acc: 100.00%  Val Loss: 2.5868, Val␣
↪Acc: 22.73%  Test Loss: 2.2816, Test Acc: 20.69%
Epoch [13/100] Train Loss: 0.0158, Train Acc: 100.00%  Val Loss: 2.5599, Val␣
↪Acc: 27.27%  Test Loss: 2.2588, Test Acc: 27.59%
Epoch [14/100] Train Loss: 0.0103, Train Acc: 100.00%  Val Loss: 2.7196, Val␣
↪Acc: 27.27%  Test Loss: 2.3517, Test Acc: 27.59%
Epoch [15/100] Train Loss: 0.0128, Train Acc: 100.00%  Val Loss: 2.8385, Val␣
↪Acc: 27.27%  Test Loss: 2.4860, Test Acc: 27.59%
Epoch [16/100] Train Loss: 0.0143, Train Acc: 100.00%  Val Loss: 2.7595, Val␣
↪Acc: 22.73%  Test Loss: 2.5182, Test Acc: 20.69%
Epoch [17/100] Train Loss: 0.0187, Train Acc: 99.49%  Val Loss: 2.7974, Val Acc:
↪ 22.73%  Test Loss: 2.6216, Test Acc: 27.59%
Epoch [18/100] Train Loss: 0.0117, Train Acc: 100.00%  Val Loss: 2.8027, Val␣
↪Acc: 27.27%  Test Loss: 2.5742, Test Acc: 24.14%
Epoch [19/100] Train Loss: 0.0418, Train Acc: 98.46%  Val Loss: 3.5509, Val Acc:
↪ 31.82%  Test Loss: 3.4534, Test Acc: 27.59%
Epoch [20/100] Train Loss: 0.0258, Train Acc: 100.00%  Val Loss: 2.5881, Val␣
↪Acc: 22.73%  Test Loss: 2.7467, Test Acc: 27.59%
Epoch [21/100] Train Loss: 0.0122, Train Acc: 100.00%  Val Loss: 2.6809, Val␣
↪Acc: 22.73%  Test Loss: 2.9817, Test Acc: 24.14%
Epoch [22/100] Train Loss: 0.0077, Train Acc: 100.00%  Val Loss: 2.6139, Val␣
↪Acc: 18.18%  Test Loss: 2.8168, Test Acc: 20.69%
```

```
Epoch [23/100] Train Loss: 0.0093, Train Acc: 100.00%  Val Loss: 2.6629, Val Acc: 22.73%  Test Loss: 2.9565, Test Acc: 17.24%
Epoch [24/100] Train Loss: 0.0082, Train Acc: 100.00%  Val Loss: 2.9867, Val Acc: 13.64%  Test Loss: 3.2057, Test Acc: 13.79%
Epoch [25/100] Train Loss: 0.0065, Train Acc: 100.00%  Val Loss: 2.9391, Val Acc: 22.73%  Test Loss: 3.2220, Test Acc: 13.79%
Epoch [26/100] Train Loss: 0.0064, Train Acc: 100.00%  Val Loss: 3.0277, Val Acc: 18.18%  Test Loss: 3.2438, Test Acc: 17.24%
Epoch [27/100] Train Loss: 0.0094, Train Acc: 100.00%  Val Loss: 2.9226, Val Acc: 22.73%  Test Loss: 3.1675, Test Acc: 6.90%
Epoch [28/100] Train Loss: 0.0054, Train Acc: 100.00%  Val Loss: 3.0974, Val Acc: 22.73%  Test Loss: 3.2699, Test Acc: 13.79%
Epoch [29/100] Train Loss: 0.0036, Train Acc: 100.00%  Val Loss: 3.0466, Val Acc: 13.64%  Test Loss: 2.9907, Test Acc: 13.79%
Epoch [30/100] Train Loss: 0.0046, Train Acc: 100.00%  Val Loss: 3.3184, Val Acc: 22.73%  Test Loss: 3.2821, Test Acc: 17.24%
Epoch [31/100] Train Loss: 0.0055, Train Acc: 100.00%  Val Loss: 3.1341, Val Acc: 22.73%  Test Loss: 3.3384, Test Acc: 17.24%
Epoch [32/100] Train Loss: 0.0041, Train Acc: 100.00%  Val Loss: 3.1856, Val Acc: 13.64%  Test Loss: 3.4162, Test Acc: 17.24%
Epoch [33/100] Train Loss: 0.0080, Train Acc: 99.49%  Val Loss: 3.3883, Val Acc: 18.18%  Test Loss: 3.3599, Test Acc: 20.69%
Epoch [34/100] Train Loss: 0.0047, Train Acc: 100.00%  Val Loss: 3.3542, Val Acc: 18.18%  Test Loss: 3.4532, Test Acc: 20.69%
Epoch [35/100] Train Loss: 0.0014, Train Acc: 100.00%  Val Loss: 3.2734, Val Acc: 22.73%  Test Loss: 3.4107, Test Acc: 20.69%
Epoch [36/100] Train Loss: 0.0023, Train Acc: 100.00%  Val Loss: 3.3832, Val Acc: 22.73%  Test Loss: 3.5366, Test Acc: 27.59%
Epoch [37/100] Train Loss: 0.0017, Train Acc: 100.00%  Val Loss: 3.4054, Val Acc: 13.64%  Test Loss: 3.5295, Test Acc: 17.24%
Epoch [38/100] Train Loss: 0.0024, Train Acc: 100.00%  Val Loss: 3.4521, Val Acc: 13.64%  Test Loss: 3.5823, Test Acc: 13.79%
Epoch [39/100] Train Loss: 0.0021, Train Acc: 100.00%  Val Loss: 3.5738, Val Acc: 27.27%  Test Loss: 3.7106, Test Acc: 20.69%
Epoch [40/100] Train Loss: 0.0025, Train Acc: 100.00%  Val Loss: 3.9014, Val Acc: 27.27%  Test Loss: 4.0055, Test Acc: 20.69%
Epoch [41/100] Train Loss: 0.0006, Train Acc: 100.00%  Val Loss: 3.6959, Val Acc: 27.27%  Test Loss: 3.8025, Test Acc: 17.24%
Epoch [42/100] Train Loss: 0.0023, Train Acc: 100.00%  Val Loss: 3.5964, Val Acc: 27.27%  Test Loss: 3.8547, Test Acc: 20.69%
Epoch [43/100] Train Loss: 0.0073, Train Acc: 100.00%  Val Loss: 2.9316, Val Acc: 13.64%  Test Loss: 3.6142, Test Acc: 13.79%
Epoch [44/100] Train Loss: 0.0044, Train Acc: 100.00%  Val Loss: 3.0540, Val Acc: 13.64%  Test Loss: 3.6170, Test Acc: 10.34%
```

```
Epoch [45/100] Train Loss: 0.0032, Train Acc: 100.00%  Val Loss: 3.5748, Val␣
 ↪Acc: 27.27%  Test Loss: 4.0837, Test Acc: 24.14%
Epoch [46/100] Train Loss: 0.0016, Train Acc: 100.00%  Val Loss: 3.4656, Val␣
 ↪Acc: 22.73%  Test Loss: 3.9503, Test Acc: 20.69%
Epoch [47/100] Train Loss: 0.0131, Train Acc: 99.49%  Val Loss: 4.2189, Val Acc:
 ↪ 22.73%  Test Loss: 4.3785, Test Acc: 20.69%
Epoch [48/100] Train Loss: 0.0029, Train Acc: 100.00%  Val Loss: 4.0402, Val␣
 ↪Acc: 18.18%  Test Loss: 4.0989, Test Acc: 24.14%
Epoch [49/100] Train Loss: 0.0010, Train Acc: 100.00%  Val Loss: 3.9135, Val␣
 ↪Acc: 9.09%  Test Loss: 3.9112, Test Acc: 17.24%
Epoch [50/100] Train Loss: 0.0011, Train Acc: 100.00%  Val Loss: 3.9708, Val␣
 ↪Acc: 9.09%  Test Loss: 4.0403, Test Acc: 24.14%
Epoch [51/100] Train Loss: 0.0015, Train Acc: 100.00%  Val Loss: 4.3230, Val␣
 ↪Acc: 13.64%  Test Loss: 4.4383, Test Acc: 20.69%
Epoch [52/100] Train Loss: 0.0023, Train Acc: 100.00%  Val Loss: 4.2511, Val␣
 ↪Acc: 9.09%  Test Loss: 4.4510, Test Acc: 20.69%
Epoch [53/100] Train Loss: 0.0038, Train Acc: 100.00%  Val Loss: 3.7221, Val␣
 ↪Acc: 22.73%  Test Loss: 4.0721, Test Acc: 17.24%
Epoch [54/100] Train Loss: 0.0052, Train Acc: 100.00%  Val Loss: 4.2340, Val␣
 ↪Acc: 13.64%  Test Loss: 3.9284, Test Acc: 20.69%
Epoch [55/100] Train Loss: 0.0023, Train Acc: 100.00%  Val Loss: 4.0188, Val␣
 ↪Acc: 9.09%  Test Loss: 4.0591, Test Acc: 13.79%
Epoch [56/100] Train Loss: 0.0008, Train Acc: 100.00%  Val Loss: 3.9503, Val␣
 ↪Acc: 9.09%  Test Loss: 4.0115, Test Acc: 10.34%
Epoch [57/100] Train Loss: 0.0030, Train Acc: 100.00%  Val Loss: 4.7352, Val␣
 ↪Acc: 9.09%  Test Loss: 4.4232, Test Acc: 20.69%
Epoch [58/100] Train Loss: 0.0049, Train Acc: 100.00%  Val Loss: 4.3102, Val␣
 ↪Acc: 9.09%  Test Loss: 3.9428, Test Acc: 13.79%
Epoch [59/100] Train Loss: 0.0017, Train Acc: 100.00%  Val Loss: 4.1713, Val␣
 ↪Acc: 13.64%  Test Loss: 3.9837, Test Acc: 17.24%
Epoch [60/100] Train Loss: 0.0013, Train Acc: 100.00%  Val Loss: 4.2698, Val␣
 ↪Acc: 18.18%  Test Loss: 4.1088, Test Acc: 17.24%
Epoch [61/100] Train Loss: 0.0021, Train Acc: 100.00%  Val Loss: 4.3671, Val␣
 ↪Acc: 9.09%  Test Loss: 4.1251, Test Acc: 20.69%
Epoch [62/100] Train Loss: 0.0031, Train Acc: 100.00%  Val Loss: 4.3767, Val␣
 ↪Acc: 13.64%  Test Loss: 4.2390, Test Acc: 17.24%
Epoch [63/100] Train Loss: 0.1959, Train Acc: 92.82%  Val Loss: 5.7032, Val Acc:
 ↪ 13.64%  Test Loss: 5.8721, Test Acc: 27.59%
Epoch [64/100] Train Loss: 0.5711, Train Acc: 81.54%  Val Loss: 2.4950, Val Acc:
 ↪ 22.73%  Test Loss: 3.1415, Test Acc: 10.34%
Epoch [65/100] Train Loss: 0.1022, Train Acc: 96.92%  Val Loss: 2.8656, Val Acc:
 ↪ 22.73%  Test Loss: 3.0863, Test Acc: 27.59%
Epoch [66/100] Train Loss: 0.0296, Train Acc: 99.49%  Val Loss: 3.5514, Val Acc:
 ↪ 13.64%  Test Loss: 3.6058, Test Acc: 24.14%
```

```
Epoch [67/100] Train Loss: 0.0137, Train Acc: 100.00%  Val Loss: 3.1628, Val␣
 ↪Acc: 18.18%  Test Loss: 3.3309, Test Acc: 27.59%
Epoch [68/100] Train Loss: 0.0136, Train Acc: 100.00%  Val Loss: 3.3355, Val␣
 ↪Acc: 22.73%  Test Loss: 3.4907, Test Acc: 20.69%
Epoch [69/100] Train Loss: 0.0048, Train Acc: 100.00%  Val Loss: 3.4556, Val␣
 ↪Acc: 22.73%  Test Loss: 3.6289, Test Acc: 20.69%
Epoch [70/100] Train Loss: 0.0065, Train Acc: 100.00%  Val Loss: 3.6060, Val␣
 ↪Acc: 18.18%  Test Loss: 3.7570, Test Acc: 20.69%
Epoch [71/100] Train Loss: 0.0037, Train Acc: 100.00%  Val Loss: 3.7229, Val␣
 ↪Acc: 27.27%  Test Loss: 3.8684, Test Acc: 20.69%
Epoch [72/100] Train Loss: 0.0036, Train Acc: 100.00%  Val Loss: 3.7688, Val␣
 ↪Acc: 27.27%  Test Loss: 3.8745, Test Acc: 20.69%
Epoch [73/100] Train Loss: 0.0025, Train Acc: 100.00%  Val Loss: 3.7816, Val␣
 ↪Acc: 27.27%  Test Loss: 3.9421, Test Acc: 24.14%
Epoch [74/100] Train Loss: 0.0039, Train Acc: 100.00%  Val Loss: 3.7088, Val␣
 ↪Acc: 22.73%  Test Loss: 3.9203, Test Acc: 20.69%
Epoch [75/100] Train Loss: 0.0033, Train Acc: 100.00%  Val Loss: 3.7763, Val␣
 ↪Acc: 22.73%  Test Loss: 4.0013, Test Acc: 24.14%
Epoch [76/100] Train Loss: 0.0039, Train Acc: 100.00%  Val Loss: 3.9450, Val␣
 ↪Acc: 22.73%  Test Loss: 4.1115, Test Acc: 20.69%
Epoch [77/100] Train Loss: 0.0024, Train Acc: 100.00%  Val Loss: 3.9254, Val␣
 ↪Acc: 22.73%  Test Loss: 4.1669, Test Acc: 20.69%
Epoch [78/100] Train Loss: 0.0014, Train Acc: 100.00%  Val Loss: 3.9517, Val␣
 ↪Acc: 22.73%  Test Loss: 4.2350, Test Acc: 20.69%
Epoch [79/100] Train Loss: 0.0058, Train Acc: 100.00%  Val Loss: 4.0433, Val␣
 ↪Acc: 22.73%  Test Loss: 4.2395, Test Acc: 20.69%
Epoch [80/100] Train Loss: 0.0018, Train Acc: 100.00%  Val Loss: 4.0615, Val␣
 ↪Acc: 22.73%  Test Loss: 4.2381, Test Acc: 20.69%
Epoch [81/100] Train Loss: 0.0022, Train Acc: 100.00%  Val Loss: 4.0374, Val␣
 ↪Acc: 22.73%  Test Loss: 4.1857, Test Acc: 20.69%
Epoch [82/100] Train Loss: 0.0036, Train Acc: 100.00%  Val Loss: 4.2060, Val␣
 ↪Acc: 22.73%  Test Loss: 4.3920, Test Acc: 20.69%
Epoch [83/100] Train Loss: 0.0015, Train Acc: 100.00%  Val Loss: 4.1798, Val␣
 ↪Acc: 22.73%  Test Loss: 4.3475, Test Acc: 20.69%
Epoch [84/100] Train Loss: 0.0017, Train Acc: 100.00%  Val Loss: 4.1340, Val␣
 ↪Acc: 22.73%  Test Loss: 4.2804, Test Acc: 24.14%
Epoch [85/100] Train Loss: 0.0017, Train Acc: 100.00%  Val Loss: 4.1817, Val␣
 ↪Acc: 22.73%  Test Loss: 4.3851, Test Acc: 20.69%
Epoch [86/100] Train Loss: 0.0010, Train Acc: 100.00%  Val Loss: 4.1203, Val␣
 ↪Acc: 22.73%  Test Loss: 4.3131, Test Acc: 20.69%
Epoch [87/100] Train Loss: 0.0006, Train Acc: 100.00%  Val Loss: 4.2593, Val␣
 ↪Acc: 22.73%  Test Loss: 4.4739, Test Acc: 20.69%
Epoch [88/100] Train Loss: 0.0006, Train Acc: 100.00%  Val Loss: 4.3655, Val␣
 ↪Acc: 22.73%  Test Loss: 4.5597, Test Acc: 20.69%
```

```
Epoch [89/100] Train Loss: 0.0078, Train Acc: 99.49%  Val Loss: 3.6485, Val Acc:
 ↪ 27.27%  Test Loss: 4.0280, Test Acc: 27.59%
Epoch [90/100] Train Loss: 0.0036, Train Acc: 100.00%  Val Loss: 3.6825, Val␣
 ↪Acc: 27.27%  Test Loss: 4.0941, Test Acc: 24.14%
Epoch [91/100] Train Loss: 0.0023, Train Acc: 100.00%  Val Loss: 3.9057, Val␣
 ↪Acc: 22.73%  Test Loss: 4.2338, Test Acc: 24.14%
Epoch [92/100] Train Loss: 0.0008, Train Acc: 100.00%  Val Loss: 3.9608, Val␣
 ↪Acc: 22.73%  Test Loss: 4.2173, Test Acc: 20.69%
Epoch [93/100] Train Loss: 0.0018, Train Acc: 100.00%  Val Loss: 3.9844, Val␣
 ↪Acc: 22.73%  Test Loss: 4.2485, Test Acc: 20.69%
Epoch [94/100] Train Loss: 0.0013, Train Acc: 100.00%  Val Loss: 4.0356, Val␣
 ↪Acc: 22.73%  Test Loss: 4.3893, Test Acc: 17.24%
Epoch [95/100] Train Loss: 0.0022, Train Acc: 100.00%  Val Loss: 4.2298, Val␣
 ↪Acc: 22.73%  Test Loss: 4.5230, Test Acc: 17.24%
Epoch [96/100] Train Loss: 0.0019, Train Acc: 100.00%  Val Loss: 4.0811, Val␣
 ↪Acc: 22.73%  Test Loss: 4.4425, Test Acc: 20.69%
Epoch [97/100] Train Loss: 0.0007, Train Acc: 100.00%  Val Loss: 4.1787, Val␣
 ↪Acc: 22.73%  Test Loss: 4.5001, Test Acc: 20.69%
Epoch [98/100] Train Loss: 0.0006, Train Acc: 100.00%  Val Loss: 4.1530, Val␣
 ↪Acc: 22.73%  Test Loss: 4.5278, Test Acc: 20.69%
Epoch [99/100] Train Loss: 0.0008, Train Acc: 100.00%  Val Loss: 4.2468, Val␣
 ↪Acc: 22.73%  Test Loss: 4.5332, Test Acc: 20.69%
Epoch [100/100] Train Loss: 0.0018, Train Acc: 100.00%  Val Loss: 4.3168, Val␣
 ↪Acc: 22.73%  Test Loss: 4.6040, Test Acc: 20.69%


## test 6. No Quantvolution (classic decision layer) + No filter (original␣
 ↪images)
Train Loss: Mean = 0.1310, Std = 0.2971
Train Accuracy: Mean = 96.23%, Std = 11.32%
Validation Loss: Mean = 0.0942, Std = 0.2797
Validation Accuracy: Mean = 98.00%, Std = 11.65%
Test Loss: Mean = 0.0892, Std = 0.2763
Test Accuracy: Mean = 98.22%, Std = 10.62%
Total Computation Time: 40.73 seconds

Epoch [1/100] Train Loss: 1.3772, Train Acc: 24.18%  Val Loss: 1.3749, Val Acc:␣
 ↪17.65%  Test Loss: 1.3638, Test Acc: 21.74%
Epoch [2/100] Train Loss: 1.3422, Train Acc: 33.99%  Val Loss: 1.3258, Val Acc:␣
 ↪29.41%  Test Loss: 1.3150, Test Acc: 34.78%
Epoch [3/100] Train Loss: 1.2878, Train Acc: 49.67%  Val Loss: 1.2562, Val Acc:␣
 ↪52.94%  Test Loss: 1.2404, Test Acc: 65.22%
Epoch [4/100] Train Loss: 1.2066, Train Acc: 75.16%  Val Loss: 1.1164, Val Acc:␣
 ↪100.00%  Test Loss: 1.0952, Test Acc: 100.00%
Epoch [5/100] Train Loss: 1.0772, Train Acc: 86.27%  Val Loss: 0.9516, Val Acc:␣
 ↪100.00%  Test Loss: 0.9322, Test Acc: 100.00%
```

```
Epoch [6/100] Train Loss: 0.9134, Train Acc: 89.54%  Val Loss: 0.7466, Val Acc:␣
↪100.00%  Test Loss: 0.7255, Test Acc: 100.00%
Epoch [7/100] Train Loss: 0.7366, Train Acc: 94.12%  Val Loss: 0.6016, Val Acc:␣
↪100.00%  Test Loss: 0.5839, Test Acc: 100.00%
Epoch [8/100] Train Loss: 0.5963, Train Acc: 89.54%  Val Loss: 0.4280, Val Acc:␣
↪100.00%  Test Loss: 0.4119, Test Acc: 100.00%
Epoch [9/100] Train Loss: 0.5149, Train Acc: 85.62%  Val Loss: 0.3099, Val Acc:␣
↪100.00%  Test Loss: 0.2906, Test Acc: 100.00%
Epoch [10/100] Train Loss: 0.3697, Train Acc: 92.16%  Val Loss: 0.2342, Val Acc:
↪ 100.00%  Test Loss: 0.2199, Test Acc: 100.00%
Epoch [11/100] Train Loss: 0.3024, Train Acc: 94.77%  Val Loss: 0.1785, Val Acc:
↪ 100.00%  Test Loss: 0.1676, Test Acc: 100.00%
Epoch [12/100] Train Loss: 0.2497, Train Acc: 96.73%  Val Loss: 0.1339, Val Acc:
↪ 100.00%  Test Loss: 0.1251, Test Acc: 100.00%
Epoch [13/100] Train Loss: 0.2389, Train Acc: 94.12%  Val Loss: 0.0981, Val Acc:
↪ 100.00%  Test Loss: 0.0879, Test Acc: 100.00%
Epoch [14/100] Train Loss: 0.1826, Train Acc: 97.39%  Val Loss: 0.0858, Val Acc:
↪ 100.00%  Test Loss: 0.0739, Test Acc: 100.00%
Epoch [15/100] Train Loss: 0.1961, Train Acc: 93.46%  Val Loss: 0.0677, Val Acc:
↪ 100.00%  Test Loss: 0.0616, Test Acc: 100.00%
Epoch [16/100] Train Loss: 0.1807, Train Acc: 96.08%  Val Loss: 0.0496, Val Acc:
↪ 100.00%  Test Loss: 0.0428, Test Acc: 100.00%
Epoch [17/100] Train Loss: 0.1295, Train Acc: 97.39%  Val Loss: 0.0407, Val Acc:
↪ 100.00%  Test Loss: 0.0319, Test Acc: 100.00%
Epoch [18/100] Train Loss: 0.1020, Train Acc: 97.39%  Val Loss: 0.0346, Val Acc:
↪ 100.00%  Test Loss: 0.0261, Test Acc: 100.00%
Epoch [19/100] Train Loss: 0.1312, Train Acc: 95.42%  Val Loss: 0.0304, Val Acc:
↪ 100.00%  Test Loss: 0.0205, Test Acc: 100.00%
Epoch [20/100] Train Loss: 0.0720, Train Acc: 99.35%  Val Loss: 0.0259, Val Acc:
↪ 100.00%  Test Loss: 0.0156, Test Acc: 100.00%
Epoch [21/100] Train Loss: 0.0451, Train Acc: 98.69%  Val Loss: 0.0215, Val Acc:
↪ 100.00%  Test Loss: 0.0106, Test Acc: 100.00%
Epoch [22/100] Train Loss: 0.0843, Train Acc: 96.73%  Val Loss: 0.0191, Val Acc:
↪ 100.00%  Test Loss: 0.0111, Test Acc: 100.00%
Epoch [23/100] Train Loss: 0.0686, Train Acc: 98.04%  Val Loss: 0.0207, Val Acc:
↪ 100.00%  Test Loss: 0.0125, Test Acc: 100.00%
Epoch [24/100] Train Loss: 0.0649, Train Acc: 97.39%  Val Loss: 0.0158, Val Acc:
↪ 100.00%  Test Loss: 0.0092, Test Acc: 100.00%
Epoch [25/100] Train Loss: 0.0471, Train Acc: 98.69%  Val Loss: 0.0153, Val Acc:
↪ 100.00%  Test Loss: 0.0075, Test Acc: 100.00%
Epoch [26/100] Train Loss: 0.0418, Train Acc: 98.69%  Val Loss: 0.0124, Val Acc:
↪ 100.00%  Test Loss: 0.0054, Test Acc: 100.00%
Epoch [27/100] Train Loss: 0.0551, Train Acc: 97.39%  Val Loss: 0.0115, Val Acc:
↪ 100.00%  Test Loss: 0.0047, Test Acc: 100.00%
```

```
Epoch [28/100] Train Loss: 0.0487, Train Acc: 99.35%  Val Loss: 0.0104, Val Acc:
 ↪ 100.00%  Test Loss: 0.0029, Test Acc: 100.00%
Epoch [29/100] Train Loss: 0.0363, Train Acc: 99.35%  Val Loss: 0.0090, Val Acc:
 ↪ 100.00%  Test Loss: 0.0025, Test Acc: 100.00%
Epoch [30/100] Train Loss: 0.0327, Train Acc: 99.35%  Val Loss: 0.0098, Val Acc:
 ↪ 100.00%  Test Loss: 0.0024, Test Acc: 100.00%
Epoch [31/100] Train Loss: 0.0415, Train Acc: 98.69%  Val Loss: 0.0080, Val Acc:
 ↪ 100.00%  Test Loss: 0.0023, Test Acc: 100.00%
Epoch [32/100] Train Loss: 0.0522, Train Acc: 99.35%  Val Loss: 0.0074, Val Acc:
 ↪ 100.00%  Test Loss: 0.0020, Test Acc: 100.00%
Epoch [33/100] Train Loss: 0.0395, Train Acc: 99.35%  Val Loss: 0.0078, Val Acc:
 ↪ 100.00%  Test Loss: 0.0017, Test Acc: 100.00%
Epoch [34/100] Train Loss: 0.0482, Train Acc: 98.69%  Val Loss: 0.0071, Val Acc:
 ↪ 100.00%  Test Loss: 0.0011, Test Acc: 100.00%
Epoch [35/100] Train Loss: 0.0320, Train Acc: 100.00%  Val Loss: 0.0079, Val␣
 ↪Acc: 100.00%  Test Loss: 0.0012, Test Acc: 100.00%
Epoch [36/100] Train Loss: 0.0227, Train Acc: 98.69%  Val Loss: 0.0042, Val Acc:
 ↪ 100.00%  Test Loss: 0.0009, Test Acc: 100.00%
Epoch [37/100] Train Loss: 0.0424, Train Acc: 98.69%  Val Loss: 0.0048, Val Acc:
 ↪ 100.00%  Test Loss: 0.0010, Test Acc: 100.00%
Epoch [38/100] Train Loss: 0.0338, Train Acc: 99.35%  Val Loss: 0.0046, Val Acc:
 ↪ 100.00%  Test Loss: 0.0008, Test Acc: 100.00%
Epoch [39/100] Train Loss: 0.0340, Train Acc: 99.35%  Val Loss: 0.0046, Val Acc:
 ↪ 100.00%  Test Loss: 0.0008, Test Acc: 100.00%
Epoch [40/100] Train Loss: 0.0391, Train Acc: 98.04%  Val Loss: 0.0049, Val Acc:
 ↪ 100.00%  Test Loss: 0.0007, Test Acc: 100.00%
Epoch [41/100] Train Loss: 0.0253, Train Acc: 99.35%  Val Loss: 0.0042, Val Acc:
 ↪ 100.00%  Test Loss: 0.0005, Test Acc: 100.00%
Epoch [42/100] Train Loss: 0.0186, Train Acc: 100.00%  Val Loss: 0.0044, Val␣
 ↪Acc: 100.00%  Test Loss: 0.0004, Test Acc: 100.00%
Epoch [43/100] Train Loss: 0.0304, Train Acc: 98.69%  Val Loss: 0.0044, Val Acc:
 ↪ 100.00%  Test Loss: 0.0005, Test Acc: 100.00%
Epoch [44/100] Train Loss: 0.0195, Train Acc: 100.00%  Val Loss: 0.0035, Val␣
 ↪Acc: 100.00%  Test Loss: 0.0002, Test Acc: 100.00%
Epoch [45/100] Train Loss: 0.0268, Train Acc: 100.00%  Val Loss: 0.0049, Val␣
 ↪Acc: 100.00%  Test Loss: 0.0003, Test Acc: 100.00%
Epoch [46/100] Train Loss: 0.0178, Train Acc: 100.00%  Val Loss: 0.0032, Val␣
 ↪Acc: 100.00%  Test Loss: 0.0002, Test Acc: 100.00%
Epoch [47/100] Train Loss: 0.0334, Train Acc: 98.04%  Val Loss: 0.0023, Val Acc:
 ↪ 100.00%  Test Loss: 0.0002, Test Acc: 100.00%
Epoch [48/100] Train Loss: 0.0120, Train Acc: 100.00%  Val Loss: 0.0027, Val␣
 ↪Acc: 100.00%  Test Loss: 0.0002, Test Acc: 100.00%
Epoch [49/100] Train Loss: 0.0222, Train Acc: 99.35%  Val Loss: 0.0029, Val Acc:
 ↪ 100.00%  Test Loss: 0.0002, Test Acc: 100.00%
```

```
Epoch [50/100] Train Loss: 0.0291, Train Acc: 98.69%  Val Loss: 0.0028, Val Acc:
 ↪ 100.00%  Test Loss: 0.0002, Test Acc: 100.00%
Epoch [51/100] Train Loss: 0.0184, Train Acc: 99.35%  Val Loss: 0.0030, Val Acc:
 ↪ 100.00%  Test Loss: 0.0002, Test Acc: 100.00%
Epoch [52/100] Train Loss: 0.0215, Train Acc: 98.69%  Val Loss: 0.0031, Val Acc:
 ↪ 100.00%  Test Loss: 0.0002, Test Acc: 100.00%
Epoch [53/100] Train Loss: 0.0081, Train Acc: 100.00%  Val Loss: 0.0028, Val␣
 ↪Acc: 100.00%  Test Loss: 0.0002, Test Acc: 100.00%
Epoch [54/100] Train Loss: 0.0206, Train Acc: 98.69%  Val Loss: 0.0027, Val Acc:
 ↪ 100.00%  Test Loss: 0.0003, Test Acc: 100.00%
Epoch [55/100] Train Loss: 0.0267, Train Acc: 99.35%  Val Loss: 0.0033, Val Acc:
 ↪ 100.00%  Test Loss: 0.0003, Test Acc: 100.00%
Epoch [56/100] Train Loss: 0.0120, Train Acc: 99.35%  Val Loss: 0.0029, Val Acc:
 ↪ 100.00%  Test Loss: 0.0002, Test Acc: 100.00%
Epoch [57/100] Train Loss: 0.0305, Train Acc: 99.35%  Val Loss: 0.0026, Val Acc:
 ↪ 100.00%  Test Loss: 0.0001, Test Acc: 100.00%
Epoch [58/100] Train Loss: 0.0119, Train Acc: 100.00%  Val Loss: 0.0027, Val␣
 ↪Acc: 100.00%  Test Loss: 0.0001, Test Acc: 100.00%
Epoch [59/100] Train Loss: 0.0085, Train Acc: 100.00%  Val Loss: 0.0021, Val␣
 ↪Acc: 100.00%  Test Loss: 0.0001, Test Acc: 100.00%
Epoch [60/100] Train Loss: 0.0097, Train Acc: 100.00%  Val Loss: 0.0021, Val␣
 ↪Acc: 100.00%  Test Loss: 0.0001, Test Acc: 100.00%
Epoch [61/100] Train Loss: 0.0134, Train Acc: 99.35%  Val Loss: 0.0024, Val Acc:
 ↪ 100.00%  Test Loss: 0.0001, Test Acc: 100.00%
Epoch [62/100] Train Loss: 0.0102, Train Acc: 99.35%  Val Loss: 0.0019, Val Acc:
 ↪ 100.00%  Test Loss: 0.0001, Test Acc: 100.00%
Epoch [63/100] Train Loss: 0.0062, Train Acc: 100.00%  Val Loss: 0.0015, Val␣
 ↪Acc: 100.00%  Test Loss: 0.0001, Test Acc: 100.00%
Epoch [64/100] Train Loss: 0.0187, Train Acc: 99.35%  Val Loss: 0.0023, Val Acc:
 ↪ 100.00%  Test Loss: 0.0001, Test Acc: 100.00%
Epoch [65/100] Train Loss: 0.0116, Train Acc: 100.00%  Val Loss: 0.0020, Val␣
 ↪Acc: 100.00%  Test Loss: 0.0001, Test Acc: 100.00%
Epoch [66/100] Train Loss: 0.0181, Train Acc: 99.35%  Val Loss: 0.0018, Val Acc:
 ↪ 100.00%  Test Loss: 0.0001, Test Acc: 100.00%
Epoch [67/100] Train Loss: 0.0179, Train Acc: 98.69%  Val Loss: 0.0015, Val Acc:
 ↪ 100.00%  Test Loss: 0.0000, Test Acc: 100.00%
Epoch [68/100] Train Loss: 0.0115, Train Acc: 100.00%  Val Loss: 0.0019, Val␣
 ↪Acc: 100.00%  Test Loss: 0.0000, Test Acc: 100.00%
Epoch [69/100] Train Loss: 0.0200, Train Acc: 98.69%  Val Loss: 0.0016, Val Acc:
 ↪ 100.00%  Test Loss: 0.0000, Test Acc: 100.00%
Epoch [70/100] Train Loss: 0.0027, Train Acc: 100.00%  Val Loss: 0.0018, Val␣
 ↪Acc: 100.00%  Test Loss: 0.0000, Test Acc: 100.00%
Epoch [71/100] Train Loss: 0.0170, Train Acc: 99.35%  Val Loss: 0.0015, Val Acc:
 ↪ 100.00%  Test Loss: 0.0000, Test Acc: 100.00%
```

```
Epoch [72/100] Train Loss: 0.0154, Train Acc: 99.35%  Val Loss: 0.0022, Val Acc:
 ↪ 100.00%  Test Loss: 0.0000, Test Acc: 100.00%
Epoch [73/100] Train Loss: 0.0207, Train Acc: 100.00%  Val Loss: 0.0030, Val␣
 ↪Acc: 100.00%  Test Loss: 0.0001, Test Acc: 100.00%
Epoch [74/100] Train Loss: 0.0055, Train Acc: 100.00%  Val Loss: 0.0017, Val␣
 ↪Acc: 100.00%  Test Loss: 0.0000, Test Acc: 100.00%
Epoch [75/100] Train Loss: 0.0064, Train Acc: 100.00%  Val Loss: 0.0015, Val␣
 ↪Acc: 100.00%  Test Loss: 0.0000, Test Acc: 100.00%
Epoch [76/100] Train Loss: 0.0146, Train Acc: 99.35%  Val Loss: 0.0009, Val Acc:
 ↪ 100.00%  Test Loss: 0.0000, Test Acc: 100.00%
Epoch [77/100] Train Loss: 0.0020, Train Acc: 100.00%  Val Loss: 0.0008, Val␣
 ↪Acc: 100.00%  Test Loss: 0.0000, Test Acc: 100.00%
Epoch [78/100] Train Loss: 0.0066, Train Acc: 100.00%  Val Loss: 0.0008, Val␣
 ↪Acc: 100.00%  Test Loss: 0.0000, Test Acc: 100.00%
Epoch [79/100] Train Loss: 0.0056, Train Acc: 100.00%  Val Loss: 0.0010, Val␣
 ↪Acc: 100.00%  Test Loss: 0.0000, Test Acc: 100.00%
Epoch [80/100] Train Loss: 0.0152, Train Acc: 100.00%  Val Loss: 0.0011, Val␣
 ↪Acc: 100.00%  Test Loss: 0.0000, Test Acc: 100.00%
Epoch [81/100] Train Loss: 0.0261, Train Acc: 99.35%  Val Loss: 0.0010, Val Acc:
 ↪ 100.00%  Test Loss: 0.0000, Test Acc: 100.00%
Epoch [82/100] Train Loss: 0.0100, Train Acc: 99.35%  Val Loss: 0.0011, Val Acc:
 ↪ 100.00%  Test Loss: 0.0000, Test Acc: 100.00%
Epoch [83/100] Train Loss: 0.0185, Train Acc: 99.35%  Val Loss: 0.0030, Val Acc:
 ↪ 100.00%  Test Loss: 0.0001, Test Acc: 100.00%
Epoch [84/100] Train Loss: 0.0342, Train Acc: 98.04%  Val Loss: 0.0010, Val Acc:
 ↪ 100.00%  Test Loss: 0.0000, Test Acc: 100.00%
Epoch [85/100] Train Loss: 0.0071, Train Acc: 100.00%  Val Loss: 0.0007, Val␣
 ↪Acc: 100.00%  Test Loss: 0.0000, Test Acc: 100.00%
Epoch [86/100] Train Loss: 0.0049, Train Acc: 100.00%  Val Loss: 0.0007, Val␣
 ↪Acc: 100.00%  Test Loss: 0.0000, Test Acc: 100.00%
Epoch [87/100] Train Loss: 0.0279, Train Acc: 98.04%  Val Loss: 0.0019, Val Acc:
 ↪ 100.00%  Test Loss: 0.0000, Test Acc: 100.00%
Epoch [88/100] Train Loss: 0.0384, Train Acc: 98.04%  Val Loss: 0.0023, Val Acc:
 ↪ 100.00%  Test Loss: 0.0001, Test Acc: 100.00%
Epoch [89/100] Train Loss: 0.0213, Train Acc: 99.35%  Val Loss: 0.0020, Val Acc:
 ↪ 100.00%  Test Loss: 0.0000, Test Acc: 100.00%
Epoch [90/100] Train Loss: 0.0027, Train Acc: 100.00%  Val Loss: 0.0021, Val␣
 ↪Acc: 100.00%  Test Loss: 0.0000, Test Acc: 100.00%
Epoch [91/100] Train Loss: 0.0370, Train Acc: 98.04%  Val Loss: 0.0027, Val Acc:
 ↪ 100.00%  Test Loss: 0.0000, Test Acc: 100.00%
Epoch [92/100] Train Loss: 0.0064, Train Acc: 100.00%  Val Loss: 0.0021, Val␣
 ↪Acc: 100.00%  Test Loss: 0.0000, Test Acc: 100.00%
Epoch [93/100] Train Loss: 0.0078, Train Acc: 100.00%  Val Loss: 0.0014, Val␣
 ↪Acc: 100.00%  Test Loss: 0.0000, Test Acc: 100.00%
```

```
Epoch [94/100] Train Loss: 0.0021, Train Acc: 100.00%  Val Loss: 0.0008, Val␣
 ↪Acc: 100.00%  Test Loss: 0.0000, Test Acc: 100.00%
Epoch [95/100] Train Loss: 0.0333, Train Acc: 98.04%  Val Loss: 0.0009, Val Acc:
 ↪ 100.00%  Test Loss: 0.0000, Test Acc: 100.00%
Epoch [96/100] Train Loss: 0.0088, Train Acc: 100.00%  Val Loss: 0.0009, Val␣
 ↪Acc: 100.00%  Test Loss: 0.0000, Test Acc: 100.00%
Epoch [97/100] Train Loss: 0.0066, Train Acc: 100.00%  Val Loss: 0.0010, Val␣
 ↪Acc: 100.00%  Test Loss: 0.0000, Test Acc: 100.00%
Epoch [98/100] Train Loss: 0.0181, Train Acc: 98.69%  Val Loss: 0.0006, Val Acc:
 ↪ 100.00%  Test Loss: 0.0000, Test Acc: 100.00%
Epoch [99/100] Train Loss: 0.0104, Train Acc: 100.00%  Val Loss: 0.0005, Val␣
 ↪Acc: 100.00%  Test Loss: 0.0000, Test Acc: 100.00%
Epoch [100/100] Train Loss: 0.0149, Train Acc: 98.69%  Val Loss: 0.0007, Val␣
 ↪Acc: 100.00%  Test Loss: 0.0000, Test Acc: 100.00%"""

# Extract test names
test_pattern = re.compile(r"## (test \d+\. .+)")
test_names = test_pattern.findall(log_data)

# Extract epoch-wise test accuracies
epoch_pattern = re.compile(r"Test Loss: [\d\.]+, Test Acc: ([\d\.]+)%")
matches = epoch_pattern.findall(log_data)

# Assign test names to accuracies
test_labels = []
accuracies = []
test_idx = 0

for line in log_data.split("\n"):
    if "## test" in line:
        test_idx += 1  # Move to next test
    elif "Test Acc:" in line:
        acc_match = re.search(r"Test Acc: ([\d\.]+)%", line)
        if acc_match:
            accuracies.append(float(acc_match.group(1)))
            test_labels.append(test_names[test_idx - 1])  # Assign current test

# Create DataFrame
df = pd.DataFrame({"Test": test_labels, "Test_Accuracy": accuracies})

# Ensure we have multiple observations per test
if df["Test"].nunique() < 2:
    raise ValueError("Not enough test groups for ANOVA.")

# Perform ANOVA
model = ols('Test_Accuracy ~ C(Test)', data=df).fit()
```

```
anova_table = sm.stats.anova_lm(model, typ=2)

# Perform Tukey's HSD test
tukey_results = pairwise_tukeyhsd(df["Test_Accuracy"], df["Test"])

# Print Results
print("ANOVA Results:\n", anova_table)
print("\nTukey HSD Test Results:\n", tukey_results)

# ---- PLOTTING ----

# Boxplot to show test accuracy distribution across different test groups
plt.figure(figsize=(12, 6))
sns.boxplot(x="Test", y="Test_Accuracy", data=df)
plt.xticks(rotation=45, ha="right")
plt.title("Distribution of Test Accuracies Across Tests")
plt.ylabel("Test Accuracy (%)")
plt.xlabel("Test Configurations")
plt.grid(axis="y", linestyle="--", alpha=0.7)
plt.show()

# Tukey HSD results visualization (Corrected group name extraction)
tukey_df = pd.DataFrame({
    "Group1": [pair[0] for pair in tukey_results._results_table.data[1:]],
    "Group2": [pair[1] for pair in tukey_results._results_table.data[1:]],
    "Mean Difference": tukey_results.meandiffs,
    "P-value": tukey_results.pvalues,
    "Reject Null": tukey_results.reject,
})

# Highlight significant differences in a scatter plot
plt.figure(figsize=(12, 6))
sns.scatterplot(
    x=tukey_df["Mean Difference"],
    y=tukey_df.index,
    hue=tukey_df["Reject Null"],
    palette={True: "red", False: "blue"},
    s=100
)
plt.axvline(0, color="black", linestyle="--")
plt.title("Tukey HSD Mean Differences (Significant Pairs in Red)")
plt.xlabel("Mean Difference in Test Accuracy")
plt.yticks(tukey_df.index, tukey_df.apply(lambda row: f"{row['Group1']} vs␣
↪{row['Group2']}", axis=1))
plt.show()
```

```
ANOVA Results:
```

```
                  sum_sq     df            F         PR(>F)
C(Test)   693272.007851    5.0  1149.152524  1.328208e-302
Residual   71670.829447  594.0          NaN            NaN

Tukey HSD Test Results:
                                                                       Multiple
Comparison of Means - Tukey HSD, FWER=0.05
================================================================================
================================================================================
============================
                               group1
group2                                    meandiff p-adj   lower    upper
reject
--------------------------------------------------------------------------------
--------------------------------------------------------------------------------
----------------------------
                            test 1. Quantvolution + Quantfilter 2x2
test 2. Quantvolution + Quantfilter 4x4 -41.1033    0.0 -45.5446  -36.662   True
                            test 1. Quantvolution + Quantfilter 2x2
test 3. Quantvolution + No Quantfilter (original images)  -1.2461 0.9671
-5.6874   3.1952  False
                            test 1. Quantvolution + Quantfilter 2x2
test 4. No Quantvolution (classic decision layer) + Quantfilter 2x2 -74.6887
0.0   -79.13 -70.2474   True
                            test 1. Quantvolution + Quantfilter 2x2
test 5. No Quantvolution (classic decision layer) + Quantfilter 4x4 -75.9995
0.0 -80.4408 -71.5582   True
                            test 1. Quantvolution + Quantfilter 2x2 test 6. No
Quantvolution (classic decision layer) + No filter (original images)   1.4933
0.9299   -2.948   5.9346  False
                            test 2. Quantvolution + Quantfilter 4x4
test 3. Quantvolution + No Quantfilter (original images)  39.8572    0.0
35.4159  44.2985   True
                            test 2. Quantvolution + Quantfilter 4x4
test 4. No Quantvolution (classic decision layer) + Quantfilter 2x2 -33.5854
0.0 -38.0267 -29.1441   True
                            test 2. Quantvolution + Quantfilter 4x4
test 5. No Quantvolution (classic decision layer) + Quantfilter 4x4 -34.8962
0.0 -39.3375 -30.4549   True
                            test 2. Quantvolution + Quantfilter 4x4 test 6. No
Quantvolution (classic decision layer) + No filter (original images)  42.5966
0.0  38.1553  47.0379   True
           test 3. Quantvolution + No Quantfilter (original images)
test 4. No Quantvolution (classic decision layer) + Quantfilter 2x2 -73.4426
0.0 -77.8839 -69.0013   True
           test 3. Quantvolution + No Quantfilter (original images)
test 5. No Quantvolution (classic decision layer) + Quantfilter 4x4 -74.7534
0.0 -79.1947 -70.3121   True
```

test 3. Quantvolution + No Quantfilter (original images) test 6. No Quantvolution (classic decision layer) + No filter (original images) 2.7394 0.4903 -1.7019 7.1807 False
test 4. No Quantvolution (classic decision layer) + Quantfilter 2x2
test 5. No Quantvolution (classic decision layer) + Quantfilter 4x4 -1.3108 0.9591 -5.7521 3.1305 False
test 4. No Quantvolution (classic decision layer) + Quantfilter 2x2 test 6. No Quantvolution (classic decision layer) + No filter (original images) 76.182 0.0 71.7407 80.6233 True
test 5. No Quantvolution (classic decision layer) + Quantfilter 4x4 test 6. No Quantvolution (classic decision layer) + No filter (original images) 77.4928 0.0 73.0515 81.9341 True
----

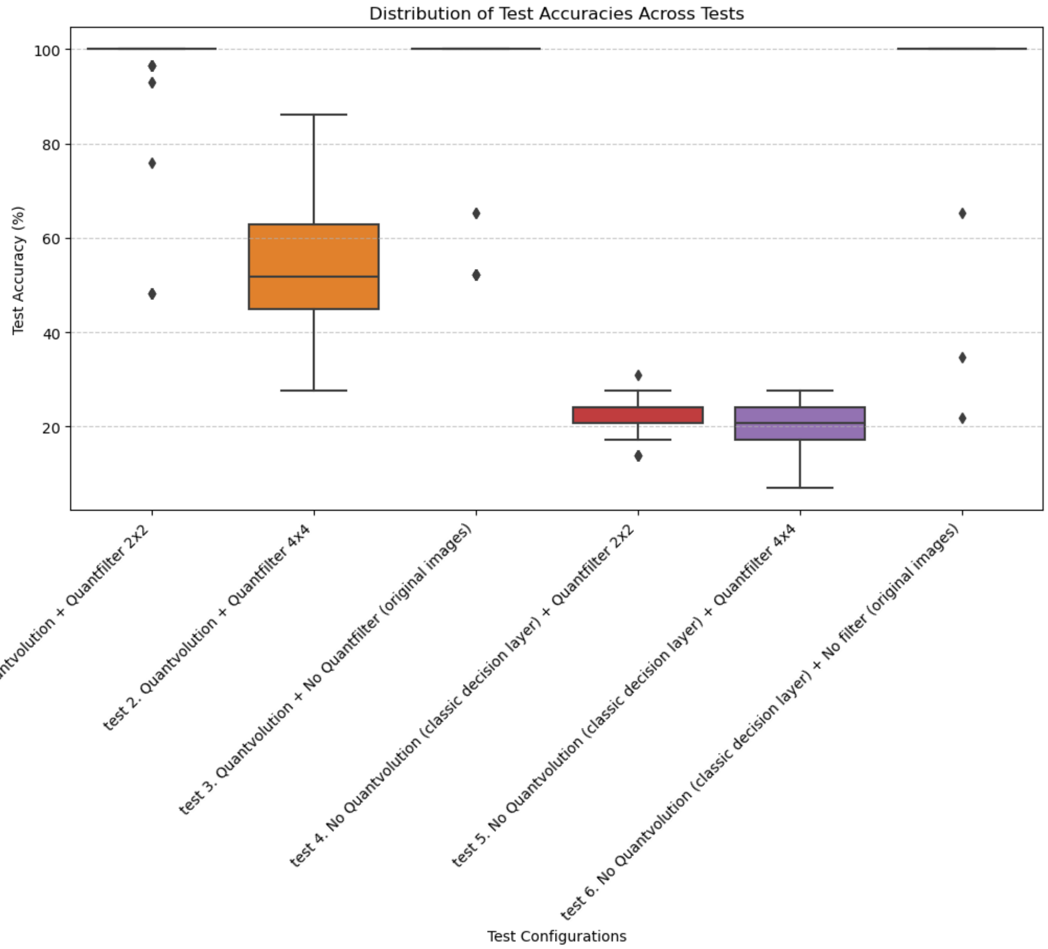

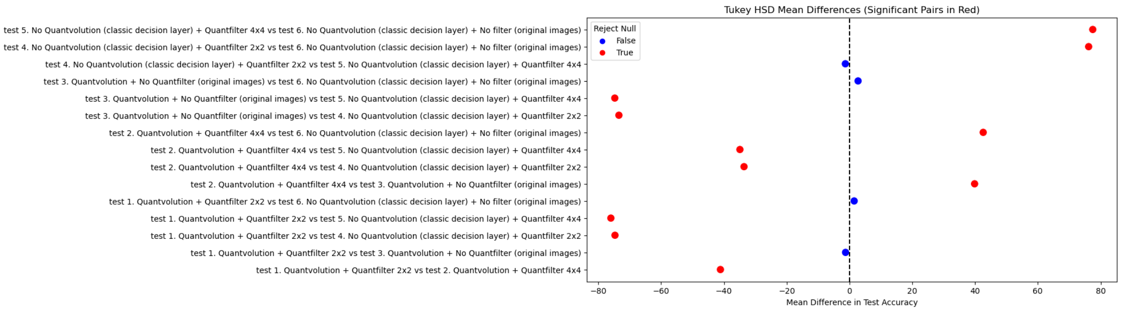

1.2.1 1. ANOVA: Are there significant differences between the models?

The results of the **ANOVA** show that **there are statistically significant differences** between the models:

Source of Variation	Sum of Squares	df	F-Statistic	p-Value
**C(Test)**	**693272.01**	5	**1149.15**	**1.32e-302** (0)
Residual	71670.83	594	NaN	NaN

ANOVA Conclusion:
The **p-value 0** confirms that at least one model performs significantly differently from the others.

However, **ANOVA does not indicate which models differ from each other**, so we use **Tukey HSD** for specific comparisons.

1.2.2 2. Pairwise Comparisons with Tukey HSD

The **Tukey test** helps identify **which models have significant differences** in **Test Accuracy**.

Model 1	Model 2	Mean Difference	p-Value	Confidence Interval (Lower - Upper)	Significant Difference?
**Test 1 (Quantvolution + Quantfilter 2x2)**	Test 2 (Quantvolution + Quantfilter 4x4)	-41.10	**0.000**	(-45.54, -36.66)	Yes
**Test 1**	Test 3 (Quantvolution + No Quantfilter)	-1.25	0.9671	(-5.69, 3.19)	No

Model 1	Model 2	Mean Difference	p-Value	Confidence Interval (Lower - Upper)	Significant Difference?
**Test 1**	Test 4 (No Quantvolution + Quantfilter 2x2)	-74.69	**0.000**	(-79.13, -70.25)	Yes
**Test 1**	Test 5 (No Quantvolution + Quantfilter 4x4)	-75.99	**0.000**	(-80.44, -71.56)	Yes
**Test 1**	Test 0 (No Quantvolution + No Filter)	1.49	0.9299	(-2.95, 5.93)	No
**Test 2**	Test 3	39.86	**0.000**	(35.42, 44.30)	Yes
**Test 2**	Test 4	-33.59	**0.000**	(-38.03, -29.14)	Yes
**Test 2**	Test 5	-34.90	**0.000**	(-39.34, -30.45)	Yes
**Test 2**	Test 0	42.60	**0.000**	(38.16, 47.04)	Yes
**Test 3**	Test 4	-73.44	**0.000**	(-77.88, -69.00)	Yes
**Test 3**	Test 5	-74.75	**0.000**	(-79.19, -70.31)	Yes
**Test 3**	Test 0	2.74	0.4903	(-1.70, 7.18)	No
**Test 4**	Test 5	-1.31	0.9591	(-5.75, 3.13)	No
**Test 4**	Test 0	76.18	**0.000**	(71.74, 80.62)	Yes
**Test 5**	Test 0	77.49	**0.000**	(73.05, 81.93)	Yes

Summary of Tukey HSD Results

1.2.3 3. Interpretation of Tukey HSD

Models that **are not significantly different** from each other (p > 0.05) include: - **Test 1 (Quantvolution + Quantfilter 2x2) vs. Test 3 (Quantvolution + No Quantfilter)**
- **Test 1 vs. Test 0 (No Quantvolution + No Filter)**
- **Test 3 vs. Test 0**
- **Test 4 (No Quantvolution + Quantfilter 2x2) vs. Test 5 (No Quantvolution + Quantfilter 4x4)**

This means that **these pairs of models have similar performance** in Test Accuracy.

Models with **significant differences** include: - **Test 2 (Quantvolution + Quantfilter 4x4) performs worse than Test 1, Test 3, and Test 0.** - **Test 4 and Test 5 perform worse than Test 0.** - **Test 0 (No Quantvolution + No Filter) is the best in accuracy, being significantly better than most.**

1.2.4 4. Final Conclusions ANOVA and Turkey HSD

Best Option by Criterion	Model	Reason
**Best balance of accuracy and speed**	**Quantvolution + Quantfilter 2x2**	High stability and accuracy with moderate computation time.
**Highest absolute accuracy**	**No Quantvolution + No Filter**	98.22% Test Accuracy, though with higher variability.
**Fastest but ineffective**	**No Quantvolution + Quantfilter 4x4**	Fast computation but poor performance (20.72% Test Accuracy).

Key Takeaway: The model "Quantvolution + Quantfilter 2x2" provides the best balance between stability, accuracy, and computational efficiency.
If the goal is purely the highest accuracy regardless of stability, then "No Quantvolution + No Filter" is the best option.
Models using "No Quantvolution + Quantfilter 4x4" suffer from severe overfitting and have the worst overall performance.

1.3 AUAC Analysis

[13]:
```
# Function to compute AUAC using the trapezoidal rule
def compute_auac(epochs, accuracies):
    """
    Compute the Area Under the Accuracy Curve (AUAC).

    :param epochs: List of epoch numbers
    :param accuracies: List of accuracy values per epoch
    :return: AUAC value
    """
    return np.trapz(accuracies, epochs)

# Extract test names
test_pattern = re.compile(r"## (test \d+\. .+)")
test_names = test_pattern.findall(log_data)

# Extract epoch-wise test accuracies
epoch_pattern = re.compile(r"Epoch \[\d+/\d+\].*?Test Acc: ([\d\.]+)%")
epoch_accuracies = epoch_pattern.findall(log_data)

# Organize extracted data
test_idx = -1
test_labels = []
accuracies = []
```

```
epoch_nums = []
current_test = None

for line in log_data.split("\n"):
    if "## test" in line:
        test_idx += 1
        current_test = test_names[test_idx]  # Update current test label
    elif "Epoch [" in line:
        epoch_match = re.search(r"Epoch \[(\d+)/\d+\]", line)
        acc_match = re.search(r"Test Acc: ([\d\.]+)%", line)

        if epoch_match and acc_match:
            epoch_nums.append(int(epoch_match.group(1)))  # Extract epoch number
            accuracies.append(float(acc_match.group(1)))  # Extract accuracy
            test_labels.append(current_test)  # Assign corresponding test label

# Create DataFrame
df = pd.DataFrame({"Test": test_labels, "Epoch": epoch_nums, "Test_Accuracy":␣
 ↪accuracies})

# Compute AUAC for each test
auac_results = []
for test in df["Test"].unique():
    test_data = df[df["Test"] == test]
    auac = compute_auac(test_data["Epoch"], test_data["Test_Accuracy"])
    auac_results.append({"Test": test, "AUAC": auac})

# Create AUAC DataFrame
df_auac = pd.DataFrame(auac_results)

# Display results
print(df_auac)

# Data from the user
tests = [
    "test 1. Quantvolution + Quantfilter 2x2",
    "test 2. Quantvolution + Quantfilter 4x4",
    "test 3. Quantvolution + No Quantfilter (original)",
    "test 4. No Quantvolution (classic decision layer) - Variant 1",
    "test 5. No Quantvolution (classic decision layer) - Variant 2",
    "test 6. No Quantvolution (classic decision layer) - Variant 3"
]
```

```
                                                  Test      AUAC
0              test 1. Quantvolution + Quantfilter 2x2  9598.270
1              test 2. Quantvolution + Quantfilter 4x4  5498.285
2   test 3. Quantvolution + No Quantfilter (origin…  9471.715
```

```
3  test 4. No Quantvolution (classic decision lay…  2182.850
4  test 5. No Quantvolution (classic decision lay…  2051.770
5  test 6. No Quantvolution (classic decision lay…  9760.870
```

[14]:
```
auac_values = [9598.270, 5498.285, 9471.715, 2182.850, 2051.770, 9760.870]

# Plot AUAC values
plt.figure(figsize=(10, 5))
plt.bar(tests, auac_values, alpha=0.7)
plt.xlabel("Tests")
plt.ylabel("AUAC")
plt.title("Area Under Accuracy Curve (AUAC) for Different Tests")
plt.xticks(rotation=45, ha="right")
plt.grid(axis='y', linestyle='--', alpha=0.7)
plt.show()
```

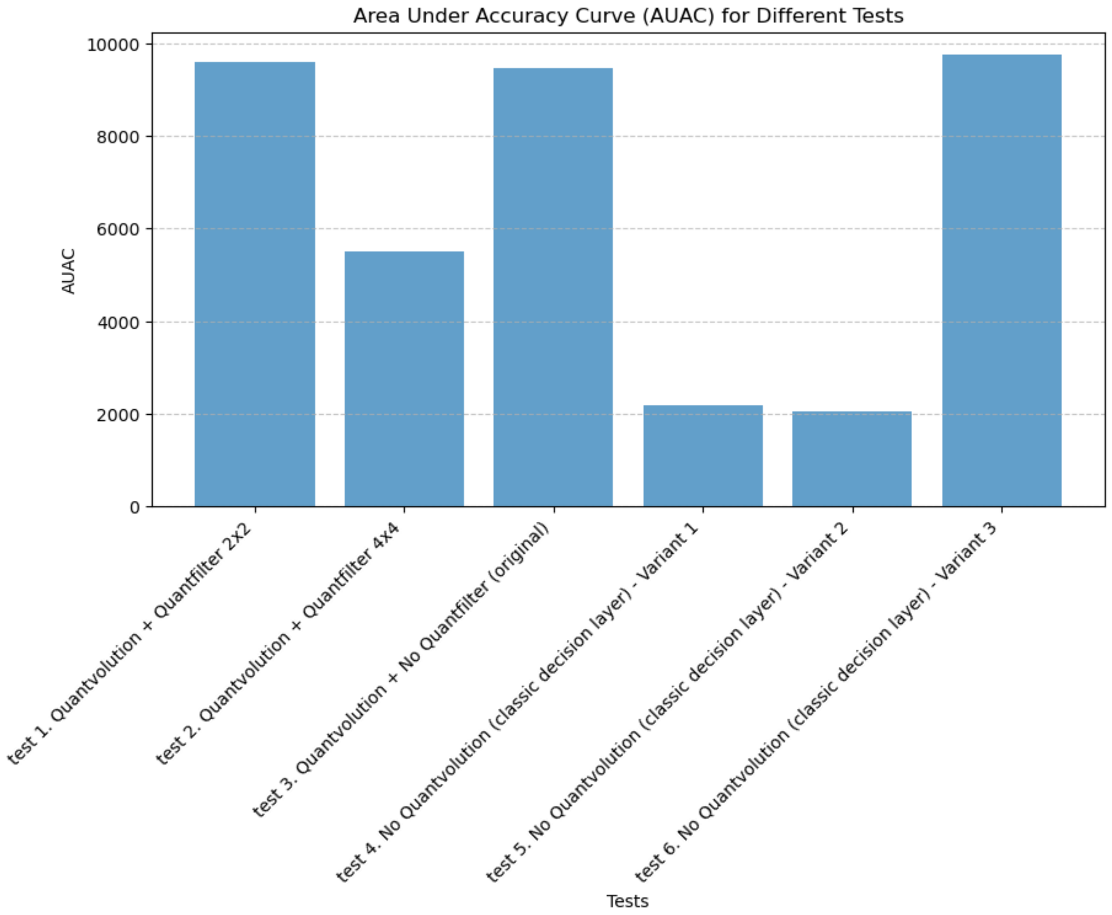

1.3.1 Interpretation of AUAC for Your Specific Results

What is AUAC? The **Area Under Accuracy Curve (AUAC)** for each test provides insight into how well each model maintained accuracy across epochs. Higher values indicate stronger and more stable performance.

**Test**	**AUAC**	**Interpretation**
**Test 1: Quantvolution + Quantfilter 2x2**	**9598.270**	One of the highest AUACs, indicating strong and stable performance. The 2x2 quantfilter effectively supports learning.
**Test 2: Quantvolution + Quantfilter 4x4**	**5498.285**	Significantly lower than Test 1, suggesting the 4x4 quantfilter may have disrupted learning stability.
**Test 3: Quantvolution + No Quantfilter (Original)**	**9471.715**	Very close to Test 1, meaning that Quantvolution without additional filtering also performs well.
**Test 4: No Quantvolution (Classic Decision Layer) - Variant 1**	**2182.850**	Drastic drop in AUAC, indicating weak learning progression over time. Suggests a much poorer performance without Quantvolution.
**Test 5: No Quantvolution (Classic Decision Layer) - Variant 2**	**2051.770**	Similar to Test 4, showing that the classic decision layer struggles without Quantvolution.
**Test 6: No Quantvolution (Classic Decision Layer) - Variant 3**	**9760.870**	Highest AUAC, competing with Test 1 and Test 3. Suggests that this variant of the classic decision layer still manages to perform well.

AUAC Results and Interpretation

1.4 test 0. Classic Baseline - No Quantvolution (classic decision layer) + No filter (original images)

[517]:
```
import numpy as np
import matplotlib.pyplot as plt
import os
import cv2
from tqdm import tqdm
from PIL import Image
from numpy import asarray
from matplotlib import image

IMG_SIZE_1 = 217
IMG_SIZE_2 = 33
```

```
DATADIR = "/Users/h4/Desktop/quantum filters_research/
↪Quantvolution_Essential_tremor/Ablation/images_split"

classes = {
    "train": ["Class1_Bad", "Class2_Good", "Class3_Right", "Class4_Left"],
    "val": ["Class1_Bad", "Class2_Good", "Class3_Right", "Class4_Left"],
    "test": ["Class1_Bad", "Class2_Good", "Class3_Right", "Class4_Left"]
}

def create_data(split):
    dataset = {class_name: [] for class_name in classes[split]}
    for i, class_name in enumerate(classes[split]):
        path = os.path.join(DATADIR, split, class_name)
        for img in tqdm(os.listdir(path), desc=f"Processing {split}␣
↪{class_name}"):
            tipo = os.path.splitext(img)[1].lower()
            if tipo in ['.jpg', '.jpeg', '.png', '.gif']:
                try:
                    img_array = asarray(Image.open(os.path.join(path, img)).
↪convert('L'))
                    new_array = cv2.resize(img_array, (IMG_SIZE_2, IMG_SIZE_1))
                    dataset[class_name].append([new_array, i])
                except Exception as e:
                    print(f"Error processing {img}: {e}")
    return dataset

train_data = create_data("train")
val_data = create_data("val")
test_data = create_data("test")

def prepare_X_y(data):
    X, y = [], []
    for class_name in data:
        for features, label in data[class_name]:
            X.append(features)
            y.append(label)
    X = np.array(X).reshape(-1, IMG_SIZE_1, IMG_SIZE_2, 1)
    y = np.array(y)
    return X, y

def unison_shuffled_copies(a, b):
    assert len(a) == len(b)
    p = np.random.permutation(len(a))
    return a[p], b[p]

X_train, y_train = prepare_X_y(train_data)
X_val, y_val = prepare_X_y(val_data)
```

```
X_test, y_test = prepare_X_y(test_data)

X_train, y_train = unison_shuffled_copies(X_train, y_train)
X_val, y_val = unison_shuffled_copies(X_val, y_val)
X_test, y_test = unison_shuffled_copies(X_test, y_test)

print("Training data shape:", X_train.shape)
print("Validation data shape:", X_val.shape)
print("Test data shape:", X_test.shape)

plt.imshow(X_train[0].squeeze(), cmap='gray')
plt.show()
```

```
Processing train Class1_Bad: 100%|       | 37/37 [00:00<00:00, 4182.21it/s]
Processing train Class2_Good: 100%|      | 45/45 [00:00<00:00, 4746.36it/s]
Processing train Class3_Right: 100%|     | 31/31 [00:00<00:00, 5790.14it/s]
Processing train Class4_Left: 100%|      | 44/44 [00:00<00:00, 6346.48it/s]
Processing val Class1_Bad: 100%|         | 4/4 [00:00<00:00, 3375.02it/s]
Processing val Class2_Good: 100%|        | 5/5 [00:00<00:00, 2990.38it/s]
Processing val Class3_Right: 100%|       | 4/4 [00:00<00:00, 3770.16it/s]
Processing val Class4_Left: 100%|        | 6/6 [00:00<00:00, 3092.76it/s]
Processing test Class1_Bad: 100%|        | 6/6 [00:00<00:00, 3968.12it/s]
Processing test Class2_Good: 100%|       | 7/7 [00:00<00:00, 3586.19it/s]
Processing test Class3_Right: 100%|      | 4/4 [00:00<00:00, 4483.49it/s]
Processing test Class4_Left: 100%|       | 8/8 [00:00<00:00, 3874.20it/s]
```

```
Training data shape: (153, 217, 33, 1)
Validation data shape: (17, 217, 33, 1)
Test data shape: (23, 217, 33, 1)
```

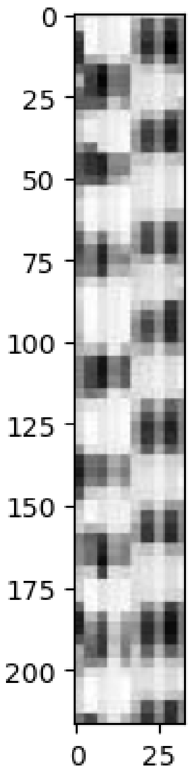

[518]:
```
train_filter = np.isin(y_train, [0, 1, 2, 3])
test_filter = np.isin(y_test, [0, 1, 2, 3])
val_filter = np.isin(y_val, [0, 1, 2, 3])

X_train, l_train = X_train[train_filter], y_train[train_filter]
X_test, l_test = X_test[test_filter], y_test[test_filter]
X_val, l_val = X_val[val_filter], y_val[val_filter]
```

[519]:
```
import torch
from torch.utils.data import Dataset, DataLoader

class CustomTensorDataset(Dataset):
    """TensorDataset with support for transforms."""
    def __init__(self, tensors, transform=None):
        assert all(tensors[0].shape[0] == tensor.shape[0] for tensor in␣
 ↪tensors), "All tensors must have the same first dimension"
        self.tensors = tensors
        self.transform = transform

    def __getitem__(self, index):
        x = self.tensors[0][index]
        if self.transform:
```

```
            x = self.transform(x)
        y = self.tensors[1][index]
        return x, y

    def __len__(self):
        return self.tensors[0].shape[0]

# Convert to PyTorch tensors
X_train_tensor = torch.tensor(X_train, dtype=torch.float32)
y_train_tensor = torch.tensor(y_train, dtype=torch.long)
X_test_tensor = torch.tensor(X_test, dtype=torch.float32)
y_test_tensor = torch.tensor(y_test, dtype=torch.long)
X_val_tensor = torch.tensor(X_val, dtype=torch.float32)
y_val_tensor = torch.tensor(y_val, dtype=torch.long)

# Create dataset and dataloader
train_dataset = CustomTensorDataset(tensors=(X_train_tensor, y_train_tensor),␣
 ↪transform=None)
train_loader = DataLoader(train_dataset, batch_size=1, shuffle=True)

test_dataset = CustomTensorDataset(tensors=(X_test_tensor, y_test_tensor),␣
 ↪transform=None)
test_loader = DataLoader(test_dataset, batch_size=1, shuffle=True)

val_dataset = CustomTensorDataset(tensors=(X_val_tensor, y_val_tensor),␣
 ↪transform=None)
val_loader = DataLoader(val_dataset, batch_size=1, shuffle=True)

# Print dataset size to check
print("Train dataset size:", len(train_dataset))
print("Test dataset size:", len(test_dataset))
print("Val dataset size:", len(val_dataset))
```

```
Train dataset size: 153
Test dataset size: 23
Val dataset size: 17
```

[521]:

```
import time
import numpy as np
import torch
import torch.nn as nn
import torch.optim as optim
import torch.nn.functional as F
from torch.utils.data import DataLoader, TensorDataset
import matplotlib.pyplot as plt

# Ensure labels are for four-class classification
```

```
y_train_tensor = torch.tensor(y_train, dtype=torch.long)
y_val_tensor = torch.tensor(y_val, dtype=torch.long)
y_test_tensor = torch.tensor(y_test, dtype=torch.long)

# Convert images to PyTorch tensors and ensure correct shape: (N, 1, 217, 33)
X_train_tensor = torch.tensor(X_train, dtype=torch.float32).permute(0, 3, 1, 2)
X_val_tensor = torch.tensor(X_val, dtype=torch.float32).permute(0, 3, 1, 2)
X_test_tensor = torch.tensor(X_test, dtype=torch.float32).permute(0, 3, 1, 2)

# Create DataLoaders
batch_size = 4
train_dataset = TensorDataset(X_train_tensor, y_train_tensor)
val_dataset = TensorDataset(X_val_tensor, y_val_tensor)
test_dataset = TensorDataset(X_test_tensor, y_test_tensor)

train_loader = DataLoader(train_dataset, batch_size=batch_size, shuffle=True)
val_loader = DataLoader(val_dataset, batch_size=batch_size, shuffle=False)
test_loader = DataLoader(test_dataset, batch_size=batch_size, shuffle=False)

##############################################
# Define Classic CNN Model (No Quantum Layer)
##############################################
class ClassicNet(nn.Module):
    def __init__(self):
        super(ClassicNet, self).__init__()
        self.conv1 = nn.Conv2d(in_channels=1, out_channels=64, kernel_size=3,
 ↪padding=1)
        self.bn1 = nn.BatchNorm2d(64)
        self.pool1 = nn.MaxPool2d(2, 2)

        self.conv2 = nn.Conv2d(64, 32, kernel_size=3, padding=1)
        self.bn2 = nn.BatchNorm2d(32)
        self.pool2 = nn.MaxPool2d(2, 2)

        self.adapt = nn.AdaptiveAvgPool2d((4, 4))  # Reduce to fixed size

        self.fc1 = nn.Linear(32 * 4 * 4, 40)
        self.fc2 = nn.Linear(40, 32)
        self.fc3 = nn.Linear(32, 16)
        self.drop = nn.Dropout(0.25)
        self.fc4 = nn.Linear(16, 8)
        self.fc5 = nn.Linear(8, 4)  # Output 4 classes

    def forward(self, x):
        x = F.relu(self.conv1(x))
        x = self.pool1(x)
        x = F.relu(self.bn1(x))
```

```python
        x = F.relu(self.conv2(x))
        x = self.pool2(x)
        x = F.relu(self.bn2(x))

        x = self.adapt(x)
        x = x.view(x.size(0), -1)  # Flatten

        x = F.relu(self.fc1(x))
        x = self.fc2(x)
        x = F.relu(self.fc3(x))
        x = self.drop(x)
        x = self.fc4(x)
        x = self.fc5(x)  # Final layer

        return x

##############################################
# Training Setup
##############################################
model = ClassicNet()
optimizer = optim.Adam(model.parameters(), lr=1e-4)
loss_func = nn.CrossEntropyLoss()

num_epochs = 100
train_losses, val_losses, test_losses = [], [], []
train_accuracies, val_accuracies, test_accuracies = [], [], []

start_time = time.time()

for epoch in range(num_epochs):
    # --- Training ---
    model.train()
    running_loss, correct_train, total_train = 0.0, 0, 0
    for data, target in train_loader:
        optimizer.zero_grad()
        outputs = model(data.float())
        loss = loss_func(outputs, target.long())
        loss.backward()
        optimizer.step()

        running_loss += loss.item() * data.size(0)
        _, predicted = torch.max(outputs, 1)
        total_train += target.size(0)
        correct_train += (predicted == target).sum().item()

    train_losses.append(running_loss / total_train)
```

```
    train_accuracies.append(correct_train / total_train * 100.0)

    # --- Validation ---
    model.eval()
    running_val_loss, correct_val, total_val = 0.0, 0, 0
    with torch.no_grad():
        for data, target in val_loader:
            outputs = model(data.float())
            loss = loss_func(outputs, target.long())
            running_val_loss += loss.item() * data.size(0)
            _, predicted = torch.max(outputs, 1)
            total_val += target.size(0)
            correct_val += (predicted == target).sum().item()

    val_losses.append(running_val_loss / total_val)
    val_accuracies.append(correct_val / total_val * 100.0)

    # --- Test Evaluation ---
    running_test_loss, correct_test, total_test = 0.0, 0, 0
    with torch.no_grad():
        for data, target in test_loader:
            outputs = model(data.float())
            loss = loss_func(outputs, target.long())
            running_test_loss += loss.item() * data.size(0)
            _, predicted = torch.max(outputs, 1)
            total_test += target.size(0)
            correct_test += (predicted == target).sum().item()

    test_losses.append(running_test_loss / total_test)
    test_accuracies.append(correct_test / total_test * 100.0)

    print(f"Epoch [{epoch+1}/{num_epochs}] "
          f"Train Loss: {train_losses[-1]:.4f}, Train Acc:␣
↪{train_accuracies[-1]:.2f}%  "
          f"Val Loss: {val_losses[-1]:.4f}, Val Acc: {val_accuracies[-1]:.2f}% ␣
↪"
          f"Test Loss: {test_losses[-1]:.4f}, Test Acc: {test_accuracies[-1]:.
↪2f}%")

end_time = time.time()
total_time = end_time - start_time

##############################################
# Print Overall Metrics
##############################################
print("\n--- Overall Metrics ---")
```

```
print(f"Train Loss: Mean = {np.mean(train_losses):.4f}, Std = {np.
  ↪std(train_losses):.4f}")
print(f"Train Accuracy: Mean = {np.mean(train_accuracies):.2f}%, Std = {np.
  ↪std(train_accuracies):.2f}%")
print(f"Validation Loss: Mean = {np.mean(val_losses):.4f}, Std = {np.
  ↪std(val_losses):.4f}")
print(f"Validation Accuracy: Mean = {np.mean(val_accuracies):.2f}%, Std = {np.
  ↪std(val_accuracies):.2f}%")
print(f"Test Loss: Mean = {np.mean(test_losses):.4f}, Std = {np.
  ↪std(test_losses):.4f}")
print(f"Test Accuracy: Mean = {np.mean(test_accuracies):.2f}%, Std = {np.
  ↪std(test_accuracies):.2f}%")
print(f"Total Computation Time: {total_time:.2f} seconds")

##############################################
# Plot Loss Curves
##############################################
epochs_range = range(1, num_epochs+1)
plt.figure(figsize=(12, 5))
plt.subplot(1, 2, 1)
plt.plot(epochs_range, train_losses, label="Train Loss", marker='o')
plt.plot(epochs_range, val_losses, label="Val Loss", marker='o')
plt.plot(epochs_range, test_losses, label="Test Loss", marker='o')
plt.xlabel("Epoch")
plt.ylabel("Loss")
plt.title("Loss vs. Epoch")
plt.legend()

##############################################
# Plot Accuracy Curves
##############################################
plt.subplot(1, 2, 2)
plt.plot(epochs_range, train_accuracies, label="Train Acc", marker='o')
plt.plot(epochs_range, val_accuracies, label="Val Acc", marker='o')
plt.plot(epochs_range, test_accuracies, label="Test Acc", marker='o')
plt.xlabel("Epoch")
plt.ylabel("Accuracy (%)")
plt.title("Accuracy vs. Epoch")
plt.legend()

plt.tight_layout()
plt.show()
```

```
Epoch [1/100] Train Loss: 1.3772, Train Acc: 24.18%  Val Loss: 1.3749, Val Acc:
17.65%  Test Loss: 1.3638, Test Acc: 21.74%
Epoch [2/100] Train Loss: 1.3422, Train Acc: 33.99%  Val Loss: 1.3258, Val Acc:
29.41%  Test Loss: 1.3150, Test Acc: 34.78%
```

Epoch [3/100] Train Loss: 1.2878, Train Acc: 49.67% Val Loss: 1.2562, Val Acc: 52.94% Test Loss: 1.2404, Test Acc: 65.22%
Epoch [4/100] Train Loss: 1.2066, Train Acc: 75.16% Val Loss: 1.1164, Val Acc: 100.00% Test Loss: 1.0952, Test Acc: 100.00%
Epoch [5/100] Train Loss: 1.0772, Train Acc: 86.27% Val Loss: 0.9516, Val Acc: 100.00% Test Loss: 0.9322, Test Acc: 100.00%
Epoch [6/100] Train Loss: 0.9134, Train Acc: 89.54% Val Loss: 0.7466, Val Acc: 100.00% Test Loss: 0.7255, Test Acc: 100.00%
Epoch [7/100] Train Loss: 0.7366, Train Acc: 94.12% Val Loss: 0.6016, Val Acc: 100.00% Test Loss: 0.5839, Test Acc: 100.00%
Epoch [8/100] Train Loss: 0.5963, Train Acc: 89.54% Val Loss: 0.4280, Val Acc: 100.00% Test Loss: 0.4119, Test Acc: 100.00%
Epoch [9/100] Train Loss: 0.5149, Train Acc: 85.62% Val Loss: 0.3099, Val Acc: 100.00% Test Loss: 0.2906, Test Acc: 100.00%
Epoch [10/100] Train Loss: 0.3697, Train Acc: 92.16% Val Loss: 0.2342, Val Acc: 100.00% Test Loss: 0.2199, Test Acc: 100.00%
Epoch [11/100] Train Loss: 0.3024, Train Acc: 94.77% Val Loss: 0.1785, Val Acc: 100.00% Test Loss: 0.1676, Test Acc: 100.00%
Epoch [12/100] Train Loss: 0.2497, Train Acc: 96.73% Val Loss: 0.1339, Val Acc: 100.00% Test Loss: 0.1251, Test Acc: 100.00%
Epoch [13/100] Train Loss: 0.2389, Train Acc: 94.12% Val Loss: 0.0981, Val Acc: 100.00% Test Loss: 0.0879, Test Acc: 100.00%
Epoch [14/100] Train Loss: 0.1826, Train Acc: 97.39% Val Loss: 0.0858, Val Acc: 100.00% Test Loss: 0.0739, Test Acc: 100.00%
Epoch [15/100] Train Loss: 0.1961, Train Acc: 93.46% Val Loss: 0.0677, Val Acc: 100.00% Test Loss: 0.0616, Test Acc: 100.00%
Epoch [16/100] Train Loss: 0.1807, Train Acc: 96.08% Val Loss: 0.0496, Val Acc: 100.00% Test Loss: 0.0428, Test Acc: 100.00%
Epoch [17/100] Train Loss: 0.1295, Train Acc: 97.39% Val Loss: 0.0407, Val Acc: 100.00% Test Loss: 0.0319, Test Acc: 100.00%
Epoch [18/100] Train Loss: 0.1020, Train Acc: 97.39% Val Loss: 0.0346, Val Acc: 100.00% Test Loss: 0.0261, Test Acc: 100.00%
Epoch [19/100] Train Loss: 0.1312, Train Acc: 95.42% Val Loss: 0.0304, Val Acc: 100.00% Test Loss: 0.0205, Test Acc: 100.00%
Epoch [20/100] Train Loss: 0.0720, Train Acc: 99.35% Val Loss: 0.0259, Val Acc: 100.00% Test Loss: 0.0156, Test Acc: 100.00%
Epoch [21/100] Train Loss: 0.0451, Train Acc: 98.69% Val Loss: 0.0215, Val Acc: 100.00% Test Loss: 0.0106, Test Acc: 100.00%
Epoch [22/100] Train Loss: 0.0843, Train Acc: 96.73% Val Loss: 0.0191, Val Acc: 100.00% Test Loss: 0.0111, Test Acc: 100.00%
Epoch [23/100] Train Loss: 0.0686, Train Acc: 98.04% Val Loss: 0.0207, Val Acc: 100.00% Test Loss: 0.0125, Test Acc: 100.00%
Epoch [24/100] Train Loss: 0.0649, Train Acc: 97.39% Val Loss: 0.0158, Val Acc: 100.00% Test Loss: 0.0092, Test Acc: 100.00%
Epoch [25/100] Train Loss: 0.0471, Train Acc: 98.69% Val Loss: 0.0153, Val Acc: 100.00% Test Loss: 0.0075, Test Acc: 100.00%
Epoch [26/100] Train Loss: 0.0418, Train Acc: 98.69% Val Loss: 0.0124, Val Acc: 100.00% Test Loss: 0.0054, Test Acc: 100.00%

```
Epoch [27/100] Train Loss: 0.0551, Train Acc: 97.39%  Val Loss: 0.0115, Val Acc:
100.00%  Test Loss: 0.0047, Test Acc: 100.00%
Epoch [28/100] Train Loss: 0.0487, Train Acc: 99.35%  Val Loss: 0.0104, Val Acc:
100.00%  Test Loss: 0.0029, Test Acc: 100.00%
Epoch [29/100] Train Loss: 0.0363, Train Acc: 99.35%  Val Loss: 0.0090, Val Acc:
100.00%  Test Loss: 0.0025, Test Acc: 100.00%
Epoch [30/100] Train Loss: 0.0327, Train Acc: 99.35%  Val Loss: 0.0098, Val Acc:
100.00%  Test Loss: 0.0024, Test Acc: 100.00%
Epoch [31/100] Train Loss: 0.0415, Train Acc: 98.69%  Val Loss: 0.0080, Val Acc:
100.00%  Test Loss: 0.0023, Test Acc: 100.00%
Epoch [32/100] Train Loss: 0.0522, Train Acc: 99.35%  Val Loss: 0.0074, Val Acc:
100.00%  Test Loss: 0.0020, Test Acc: 100.00%
Epoch [33/100] Train Loss: 0.0395, Train Acc: 99.35%  Val Loss: 0.0078, Val Acc:
100.00%  Test Loss: 0.0017, Test Acc: 100.00%
Epoch [34/100] Train Loss: 0.0482, Train Acc: 98.69%  Val Loss: 0.0071, Val Acc:
100.00%  Test Loss: 0.0011, Test Acc: 100.00%
Epoch [35/100] Train Loss: 0.0320, Train Acc: 100.00%  Val Loss: 0.0079, Val
Acc: 100.00%  Test Loss: 0.0012, Test Acc: 100.00%
Epoch [36/100] Train Loss: 0.0227, Train Acc: 98.69%  Val Loss: 0.0042, Val Acc:
100.00%  Test Loss: 0.0009, Test Acc: 100.00%
Epoch [37/100] Train Loss: 0.0424, Train Acc: 98.69%  Val Loss: 0.0048, Val Acc:
100.00%  Test Loss: 0.0010, Test Acc: 100.00%
Epoch [38/100] Train Loss: 0.0338, Train Acc: 99.35%  Val Loss: 0.0046, Val Acc:
100.00%  Test Loss: 0.0008, Test Acc: 100.00%
Epoch [39/100] Train Loss: 0.0340, Train Acc: 99.35%  Val Loss: 0.0046, Val Acc:
100.00%  Test Loss: 0.0008, Test Acc: 100.00%
Epoch [40/100] Train Loss: 0.0391, Train Acc: 98.04%  Val Loss: 0.0049, Val Acc:
100.00%  Test Loss: 0.0007, Test Acc: 100.00%
Epoch [41/100] Train Loss: 0.0253, Train Acc: 99.35%  Val Loss: 0.0042, Val Acc:
100.00%  Test Loss: 0.0005, Test Acc: 100.00%
Epoch [42/100] Train Loss: 0.0186, Train Acc: 100.00%  Val Loss: 0.0044, Val
Acc: 100.00%  Test Loss: 0.0004, Test Acc: 100.00%
Epoch [43/100] Train Loss: 0.0304, Train Acc: 98.69%  Val Loss: 0.0044, Val Acc:
100.00%  Test Loss: 0.0005, Test Acc: 100.00%
Epoch [44/100] Train Loss: 0.0195, Train Acc: 100.00%  Val Loss: 0.0035, Val
Acc: 100.00%  Test Loss: 0.0002, Test Acc: 100.00%
Epoch [45/100] Train Loss: 0.0268, Train Acc: 100.00%  Val Loss: 0.0049, Val
Acc: 100.00%  Test Loss: 0.0003, Test Acc: 100.00%
Epoch [46/100] Train Loss: 0.0178, Train Acc: 100.00%  Val Loss: 0.0032, Val
Acc: 100.00%  Test Loss: 0.0002, Test Acc: 100.00%
Epoch [47/100] Train Loss: 0.0334, Train Acc: 98.04%  Val Loss: 0.0023, Val Acc:
100.00%  Test Loss: 0.0002, Test Acc: 100.00%
Epoch [48/100] Train Loss: 0.0120, Train Acc: 100.00%  Val Loss: 0.0027, Val
Acc: 100.00%  Test Loss: 0.0002, Test Acc: 100.00%
Epoch [49/100] Train Loss: 0.0222, Train Acc: 99.35%  Val Loss: 0.0029, Val Acc:
100.00%  Test Loss: 0.0002, Test Acc: 100.00%
Epoch [50/100] Train Loss: 0.0291, Train Acc: 98.69%  Val Loss: 0.0028, Val Acc:
100.00%  Test Loss: 0.0002, Test Acc: 100.00%
```

Epoch [51/100] Train Loss: 0.0184, Train Acc: 99.35% Val Loss: 0.0030, Val Acc: 100.00% Test Loss: 0.0002, Test Acc: 100.00%
Epoch [52/100] Train Loss: 0.0215, Train Acc: 98.69% Val Loss: 0.0031, Val Acc: 100.00% Test Loss: 0.0002, Test Acc: 100.00%
Epoch [53/100] Train Loss: 0.0081, Train Acc: 100.00% Val Loss: 0.0028, Val Acc: 100.00% Test Loss: 0.0002, Test Acc: 100.00%
Epoch [54/100] Train Loss: 0.0206, Train Acc: 98.69% Val Loss: 0.0027, Val Acc: 100.00% Test Loss: 0.0003, Test Acc: 100.00%
Epoch [55/100] Train Loss: 0.0267, Train Acc: 99.35% Val Loss: 0.0033, Val Acc: 100.00% Test Loss: 0.0003, Test Acc: 100.00%
Epoch [56/100] Train Loss: 0.0120, Train Acc: 99.35% Val Loss: 0.0029, Val Acc: 100.00% Test Loss: 0.0002, Test Acc: 100.00%
Epoch [57/100] Train Loss: 0.0305, Train Acc: 99.35% Val Loss: 0.0026, Val Acc: 100.00% Test Loss: 0.0001, Test Acc: 100.00%
Epoch [58/100] Train Loss: 0.0119, Train Acc: 100.00% Val Loss: 0.0027, Val Acc: 100.00% Test Loss: 0.0001, Test Acc: 100.00%
Epoch [59/100] Train Loss: 0.0085, Train Acc: 100.00% Val Loss: 0.0021, Val Acc: 100.00% Test Loss: 0.0001, Test Acc: 100.00%
Epoch [60/100] Train Loss: 0.0097, Train Acc: 100.00% Val Loss: 0.0021, Val Acc: 100.00% Test Loss: 0.0001, Test Acc: 100.00%
Epoch [61/100] Train Loss: 0.0134, Train Acc: 99.35% Val Loss: 0.0024, Val Acc: 100.00% Test Loss: 0.0001, Test Acc: 100.00%
Epoch [62/100] Train Loss: 0.0102, Train Acc: 99.35% Val Loss: 0.0019, Val Acc: 100.00% Test Loss: 0.0001, Test Acc: 100.00%
Epoch [63/100] Train Loss: 0.0062, Train Acc: 100.00% Val Loss: 0.0015, Val Acc: 100.00% Test Loss: 0.0001, Test Acc: 100.00%
Epoch [64/100] Train Loss: 0.0187, Train Acc: 99.35% Val Loss: 0.0023, Val Acc: 100.00% Test Loss: 0.0001, Test Acc: 100.00%
Epoch [65/100] Train Loss: 0.0116, Train Acc: 100.00% Val Loss: 0.0020, Val Acc: 100.00% Test Loss: 0.0001, Test Acc: 100.00%
Epoch [66/100] Train Loss: 0.0181, Train Acc: 99.35% Val Loss: 0.0018, Val Acc: 100.00% Test Loss: 0.0001, Test Acc: 100.00%
Epoch [67/100] Train Loss: 0.0179, Train Acc: 98.69% Val Loss: 0.0015, Val Acc: 100.00% Test Loss: 0.0000, Test Acc: 100.00%
Epoch [68/100] Train Loss: 0.0115, Train Acc: 100.00% Val Loss: 0.0019, Val Acc: 100.00% Test Loss: 0.0000, Test Acc: 100.00%
Epoch [69/100] Train Loss: 0.0200, Train Acc: 98.69% Val Loss: 0.0016, Val Acc: 100.00% Test Loss: 0.0000, Test Acc: 100.00%
Epoch [70/100] Train Loss: 0.0027, Train Acc: 100.00% Val Loss: 0.0018, Val Acc: 100.00% Test Loss: 0.0000, Test Acc: 100.00%
Epoch [71/100] Train Loss: 0.0170, Train Acc: 99.35% Val Loss: 0.0015, Val Acc: 100.00% Test Loss: 0.0000, Test Acc: 100.00%
Epoch [72/100] Train Loss: 0.0154, Train Acc: 99.35% Val Loss: 0.0022, Val Acc: 100.00% Test Loss: 0.0000, Test Acc: 100.00%
Epoch [73/100] Train Loss: 0.0207, Train Acc: 100.00% Val Loss: 0.0030, Val Acc: 100.00% Test Loss: 0.0001, Test Acc: 100.00%
Epoch [74/100] Train Loss: 0.0055, Train Acc: 100.00% Val Loss: 0.0017, Val Acc: 100.00% Test Loss: 0.0000, Test Acc: 100.00%

Epoch [75/100] Train Loss: 0.0064, Train Acc: 100.00% Val Loss: 0.0015, Val Acc: 100.00% Test Loss: 0.0000, Test Acc: 100.00%
Epoch [76/100] Train Loss: 0.0146, Train Acc: 99.35% Val Loss: 0.0009, Val Acc: 100.00% Test Loss: 0.0000, Test Acc: 100.00%
Epoch [77/100] Train Loss: 0.0020, Train Acc: 100.00% Val Loss: 0.0008, Val Acc: 100.00% Test Loss: 0.0000, Test Acc: 100.00%
Epoch [78/100] Train Loss: 0.0066, Train Acc: 100.00% Val Loss: 0.0008, Val Acc: 100.00% Test Loss: 0.0000, Test Acc: 100.00%
Epoch [79/100] Train Loss: 0.0056, Train Acc: 100.00% Val Loss: 0.0010, Val Acc: 100.00% Test Loss: 0.0000, Test Acc: 100.00%
Epoch [80/100] Train Loss: 0.0152, Train Acc: 100.00% Val Loss: 0.0011, Val Acc: 100.00% Test Loss: 0.0000, Test Acc: 100.00%
Epoch [81/100] Train Loss: 0.0261, Train Acc: 99.35% Val Loss: 0.0010, Val Acc: 100.00% Test Loss: 0.0000, Test Acc: 100.00%
Epoch [82/100] Train Loss: 0.0100, Train Acc: 99.35% Val Loss: 0.0011, Val Acc: 100.00% Test Loss: 0.0000, Test Acc: 100.00%
Epoch [83/100] Train Loss: 0.0185, Train Acc: 99.35% Val Loss: 0.0030, Val Acc: 100.00% Test Loss: 0.0001, Test Acc: 100.00%
Epoch [84/100] Train Loss: 0.0342, Train Acc: 98.04% Val Loss: 0.0010, Val Acc: 100.00% Test Loss: 0.0000, Test Acc: 100.00%
Epoch [85/100] Train Loss: 0.0071, Train Acc: 100.00% Val Loss: 0.0007, Val Acc: 100.00% Test Loss: 0.0000, Test Acc: 100.00%
Epoch [86/100] Train Loss: 0.0049, Train Acc: 100.00% Val Loss: 0.0007, Val Acc: 100.00% Test Loss: 0.0000, Test Acc: 100.00%
Epoch [87/100] Train Loss: 0.0279, Train Acc: 98.04% Val Loss: 0.0019, Val Acc: 100.00% Test Loss: 0.0000, Test Acc: 100.00%
Epoch [88/100] Train Loss: 0.0384, Train Acc: 98.04% Val Loss: 0.0023, Val Acc: 100.00% Test Loss: 0.0001, Test Acc: 100.00%
Epoch [89/100] Train Loss: 0.0213, Train Acc: 99.35% Val Loss: 0.0020, Val Acc: 100.00% Test Loss: 0.0000, Test Acc: 100.00%
Epoch [90/100] Train Loss: 0.0027, Train Acc: 100.00% Val Loss: 0.0021, Val Acc: 100.00% Test Loss: 0.0000, Test Acc: 100.00%
Epoch [91/100] Train Loss: 0.0370, Train Acc: 98.04% Val Loss: 0.0027, Val Acc: 100.00% Test Loss: 0.0000, Test Acc: 100.00%
Epoch [92/100] Train Loss: 0.0064, Train Acc: 100.00% Val Loss: 0.0021, Val Acc: 100.00% Test Loss: 0.0000, Test Acc: 100.00%
Epoch [93/100] Train Loss: 0.0078, Train Acc: 100.00% Val Loss: 0.0014, Val Acc: 100.00% Test Loss: 0.0000, Test Acc: 100.00%
Epoch [94/100] Train Loss: 0.0021, Train Acc: 100.00% Val Loss: 0.0008, Val Acc: 100.00% Test Loss: 0.0000, Test Acc: 100.00%
Epoch [95/100] Train Loss: 0.0333, Train Acc: 98.04% Val Loss: 0.0009, Val Acc: 100.00% Test Loss: 0.0000, Test Acc: 100.00%
Epoch [96/100] Train Loss: 0.0088, Train Acc: 100.00% Val Loss: 0.0009, Val Acc: 100.00% Test Loss: 0.0000, Test Acc: 100.00%
Epoch [97/100] Train Loss: 0.0066, Train Acc: 100.00% Val Loss: 0.0010, Val Acc: 100.00% Test Loss: 0.0000, Test Acc: 100.00%
Epoch [98/100] Train Loss: 0.0181, Train Acc: 98.69% Val Loss: 0.0006, Val Acc: 100.00% Test Loss: 0.0000, Test Acc: 100.00%

```
Epoch [99/100] Train Loss: 0.0104, Train Acc: 100.00%  Val Loss: 0.0005, Val
Acc: 100.00%  Test Loss: 0.0000, Test Acc: 100.00%
Epoch [100/100] Train Loss: 0.0149, Train Acc: 98.69%  Val Loss: 0.0007, Val
Acc: 100.00%  Test Loss: 0.0000, Test Acc: 100.00%

--- Overall Metrics ---
Train Loss: Mean = 0.1310, Std = 0.2971
Train Accuracy: Mean = 96.23%, Std = 11.32%
Validation Loss: Mean = 0.0942, Std = 0.2797
Validation Accuracy: Mean = 98.00%, Std = 11.65%
Test Loss: Mean = 0.0892, Std = 0.2763
Test Accuracy: Mean = 98.22%, Std = 10.62%
Total Computation Time: 40.73 seconds
```

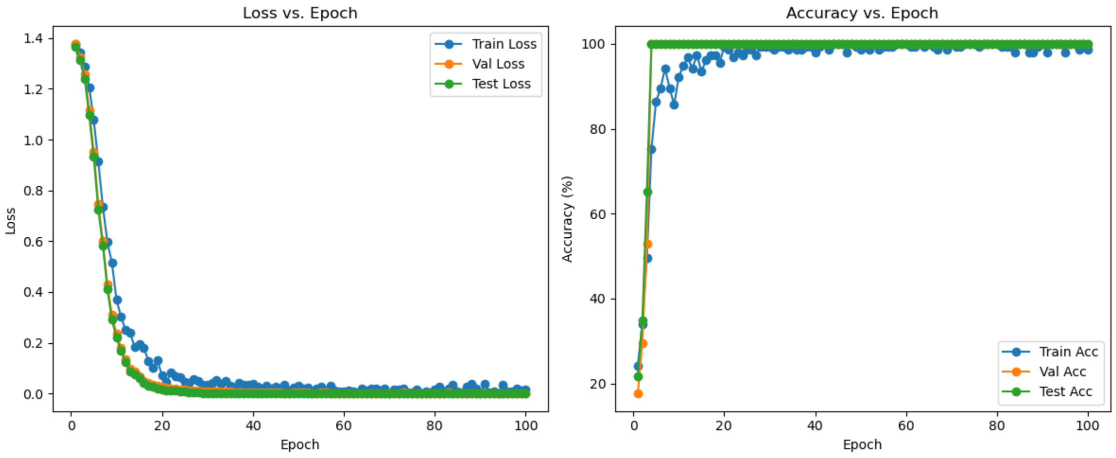

1.5 test 1. Quantvolution + Quantfilter 2x2

[343]:
```
import numpy as np
import pennylane as qml
from pennylane import numpy as np
from pennylane.templates import RandomLayers
import tensorflow as tf
from tensorflow import keras
import matplotlib.pyplot as plt
import numpy as np
import matplotlib.pyplot as plt

import keras

import torch
from torch.autograd import Function
```

```
import torch.optim as optim
import torch.nn as nn
import torch.nn.functional as F

from torchvision import datasets, transforms
```

[344]:
```
import pennylane as qml
import numpy as np
from PIL import Image
from torchvision import datasets, transforms

class QuantumCircuit:
    """
    This class provides a simple interface for interaction
    with the quantum circuit using PennyLane.
    """

    def __init__(self, n_qubits, shots):
        self.n_qubits = n_qubits
        self.shots = shots

        # Define a PennyLane device (simulator)
        self.dev = qml.device("default.qubit", wires=n_qubits, shots=shots)

        # Define the quantum circuit
        @qml.qnode(self.dev)
        def circuit(theta):
            qml.RX(theta[0], wires=0)
            qml.RX(theta[1], wires=1)

            return qml.probs(wires=[0, 1])

        self.circuit = circuit

    def run(self, thetas):
        if isinstance(thetas, list):
            thetas = np.array(thetas)

        # Ensure `thetas` has the correct shape
        if thetas.shape != (2,):
            raise ValueError(f"Expected `thetas` to have shape (2,), but got␣
↪shape {thetas.shape} with values {thetas}")

        probabilities = self.circuit(thetas)
```

```
        return np.array([probabilities[0] + probabilities[2], probabilities[1]␣
↪+ probabilities[3]])


    def draw(self):
        qml.draw_mpl(self.circuit)([np.pi/5, np.pi/3])
```

[345]:
```
import numpy as np
from PIL import Image
from torchvision import datasets, transforms

# Define number of qubits and shots
n_qubits = 2
shots = 100

# Create an instance of the QuantumCircuit class
quantum_circuit = QuantumCircuit(n_qubits, shots)

# Draw the quantum circuit
quantum_circuit.draw()

# Run the circuit with specified angles
result = quantum_circuit.run([np.pi/5, np.pi/3])

# Print the output probabilities
print("Output probabilities:", result)
```

```
Output probabilities: [0.75 0.25]
```

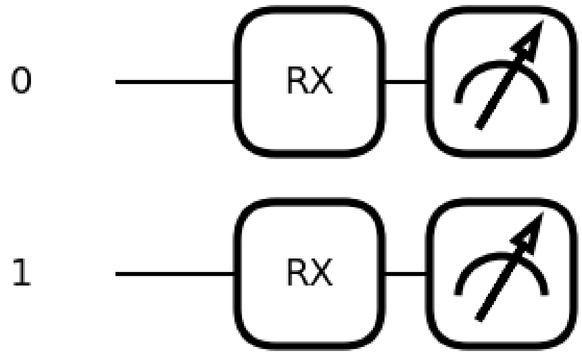

[346]:
```
import pennylane as qml
import numpy as np
import torch
import torch.nn as nn
import random
from torch.autograd import Function

class HybridFunction(Function):
    """ Hybrid quantum - classical function definition """

    @staticmethod
    @staticmethod
    def forward(ctx, input, quantum_circuit, shift):
        """ Forward pass computation """
        ctx.shift = shift
        ctx.quantum_circuit = quantum_circuit

        # Ensure `input` is a 1D NumPy array
        input_array = input.detach().cpu().numpy().flatten()  # Converts tensor␣
↪to a flat NumPy array

        if input_array.shape[0] != 2:
            raise ValueError(f"Expected input to have 2 elements, but got shape␣
↪{input_array.shape} with values {input_array}")

        probabilities = ctx.quantum_circuit.run(input_array)

        probabilities_shift_plus_ = ctx.quantum_circuit.run(
            np.array([input_array[0] + np.pi / 2 + random.uniform(1e-6, 1e-5),␣
↪input_array[1] + np.pi / 2])
        )

        probabilities_shift_minus_ = ctx.quantum_circuit.run(
            np.array([input_array[0] - np.pi / 2 + random.uniform(1e-6, 1e-5),␣
↪input_array[1] - np.pi / 2])
        )

        # Convert to tensors
        probabilities = torch.tensor(probabilities, dtype=torch.float32)
        probabilities_shift_plus_ = torch.tensor(probabilities_shift_plus_,␣
↪dtype=torch.float32)
        probabilities_shift_minus_ = torch.tensor(probabilities_shift_minus_,␣
↪dtype=torch.float32)
```

```
        ctx.save_for_backward(input, probabilities, probabilities_shift_plus_,␣
↪probabilities_shift_minus_)

        return probabilities


    @staticmethod
    def backward(ctx, grad_output):
        """ Backward pass computation """
        input, probabilities, probabilities_shift_plus_,␣
↪probabilities_shift_minus_ = ctx.saved_tensors

        # derivative of a quantum circuit is f'(x)=(f(x+pi/2)-f(x-pi/2))/2
        gradient = ((probabilities_shift_plus_ - probabilities_shift_minus_)/2).
↪clone().detach().requires_grad_(True)

        grad = gradient.float() * grad_output.float().clone().detach().
↪requires_grad_(True)

        return grad, None, None

class Hybrid(nn.Module):
    """ Hybrid quantum - classical layer definition """

    def __init__(self, shots, shift):
        super(Hybrid, self).__init__()
        self.quantum_circuit = QuantumCircuit(2, shots)
        self.shift = shift

    def forward(self, input):
        return HybridFunction.apply(input, self.quantum_circuit, self.shift)
```

[347]:
```
import numpy as np
import matplotlib.pyplot as plt
import os
import cv2
from tqdm import tqdm
from PIL import Image
from numpy import asarray
from matplotlib import image

IMG_SIZE_1 = 217
IMG_SIZE_2 = 33
DATADIR = "/Users/h4/Desktop/quantum filters_research/
↪Quantvolution_Essential_tremor/Ablation/images_split"

classes = {
```

```python
    "train": ["Class1_Bad", "Class2_Good", "Class3_Right", "Class4_Left"],
    "val": ["Class1_Bad", "Class2_Good", "Class3_Right", "Class4_Left"],
    "test": ["Class1_Bad", "Class2_Good", "Class3_Right", "Class4_Left"]
}

def create_data(split):
    dataset = {class_name: [] for class_name in classes[split]}
    for i, class_name in enumerate(classes[split]):
        path = os.path.join(DATADIR, split, class_name)
        for img in tqdm(os.listdir(path), desc=f"Processing {split}␣
↪{class_name}"):
            tipo = os.path.splitext(img)[1].lower()
            if tipo in ['.jpg', '.jpeg', '.png', '.gif']:
                try:
                    img_array = asarray(Image.open(os.path.join(path, img)).
↪convert('L'))
                    new_array = cv2.resize(img_array, (IMG_SIZE_2, IMG_SIZE_1))
                    dataset[class_name].append([new_array, i])
                except Exception as e:
                    print(f"Error processing {img}: {e}")
    return dataset

train_data = create_data("train")
val_data = create_data("val")
test_data = create_data("test")

def prepare_X_y(data):
    X, y = [], []
    for class_name in data:
        for features, label in data[class_name]:
            X.append(features)
            y.append(label)
    X = np.array(X).reshape(-1, IMG_SIZE_1, IMG_SIZE_2, 1)
    y = np.array(y)
    return X, y

def unison_shuffled_copies(a, b):
    assert len(a) == len(b)
    p = np.random.permutation(len(a))
    return a[p], b[p]

X_train, y_train = prepare_X_y(train_data)
X_val, y_val = prepare_X_y(val_data)
X_test, y_test = prepare_X_y(test_data)

X_train, y_train = unison_shuffled_copies(X_train, y_train)
X_val, y_val = unison_shuffled_copies(X_val, y_val)
```

```python
X_test, y_test = unison_shuffled_copies(X_test, y_test)

print("Training data shape:", X_train.shape)
print("Validation data shape:", X_val.shape)
print("Test data shape:", X_test.shape)

plt.imshow(X_train[0].squeeze(), cmap='gray')
plt.show()
```

```
Processing train Class1_Bad: 100%|          | 47/47 [00:00<00:00, 3750.40it/s]
Processing train Class2_Good: 100%|          | 47/47 [00:00<00:00, 3862.61it/s]
Processing train Class3_Right: 100%|          | 48/48 [00:00<00:00, 5138.64it/s]
Processing train Class4_Left: 100%|          | 53/53 [00:00<00:00, 5108.31it/s]
Processing val Class1_Bad: 100%|          | 5/5 [00:00<00:00, 3135.22it/s]
Processing val Class2_Good: 100%|          | 5/5 [00:00<00:00, 3362.44it/s]
Processing val Class3_Right: 100%|          | 6/6 [00:00<00:00, 3455.89it/s]
Processing val Class4_Left: 100%|          | 6/6 [00:00<00:00, 3305.64it/s]
Processing test Class1_Bad: 100%|          | 7/7 [00:00<00:00, 4000.02it/s]
Processing test Class2_Good: 100%|          | 7/7 [00:00<00:00, 3977.26it/s]
Processing test Class3_Right: 100%|          | 7/7 [00:00<00:00, 3911.03it/s]
Processing test Class4_Left: 100%|          | 8/8 [00:00<00:00, 4136.90it/s]
```

```
Training data shape: (195, 217, 33, 1)
Validation data shape: (22, 217, 33, 1)
Test data shape: (29, 217, 33, 1)
```

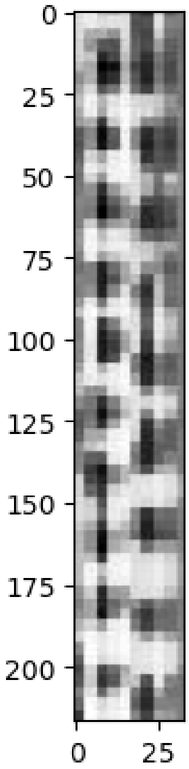

[348]:
```
train_filter = np.isin(y_train, [0, 1, 2, 3])
test_filter = np.isin(y_test, [0, 1, 2, 3])
val_filter = np.isin(y_val, [0, 1, 2, 3])

X_train, l_train = X_train[train_filter], y_train[train_filter]
X_test, l_test = X_test[test_filter], y_test[test_filter]
X_val, l_val = X_val[val_filter], y_val[val_filter]
```

[349]:
```
print(np.shape(X_train))
print(np.shape(y_train))
print(np.shape(X_val))
print(np.shape(y_val))
print(np.shape(X_test))
print(np.shape(y_test))
```

```
(195, 217, 33, 1)
(195,)
(22, 217, 33, 1)
(22,)
(29, 217, 33, 1)
(29,)
```

[350]:
```python
from numpy import array
from numpy import argmax
from sklearn.preprocessing import LabelEncoder
from sklearn.preprocessing import OneHotEncoder
label_encoder = LabelEncoder()
integer_encoded = label_encoder.fit_transform(y_train)
# binary encode
onehot_encoder = OneHotEncoder(sparse=False)
integer_encoded = integer_encoded.reshape(len(integer_encoded), 1)
y_train = onehot_encoder.fit_transform(integer_encoded)

label_encoder = LabelEncoder()
integer_encoded = label_encoder.fit_transform(y_test)
# binary encode
onehot_encoder = OneHotEncoder(sparse=False)
integer_encoded = integer_encoded.reshape(len(integer_encoded), 1)
y_test = onehot_encoder.fit_transform(integer_encoded)

label_encoder = LabelEncoder()
integer_encoded = label_encoder.fit_transform(y_val)
# binary encode
onehot_encoder = OneHotEncoder(sparse=False)
integer_encoded = integer_encoded.reshape(len(integer_encoded), 1)
y_val = onehot_encoder.fit_transform(integer_encoded)
```

```
/Applications/anaconda3/lib/python3.11/site-
packages/sklearn/preprocessing/_encoders.py:868: FutureWarning: `sparse` was
renamed to `sparse_output` in version 1.2 and will be removed in 1.4.
`sparse_output` is ignored unless you leave `sparse` to its default value.
  warnings.warn(
/Applications/anaconda3/lib/python3.11/site-
packages/sklearn/preprocessing/_encoders.py:868: FutureWarning: `sparse` was
renamed to `sparse_output` in version 1.2 and will be removed in 1.4.
`sparse_output` is ignored unless you leave `sparse` to its default value.
  warnings.warn(
/Applications/anaconda3/lib/python3.11/site-
packages/sklearn/preprocessing/_encoders.py:868: FutureWarning: `sparse` was
renamed to `sparse_output` in version 1.2 and will be removed in 1.4.
`sparse_output` is ignored unless you leave `sparse` to its default value.
  warnings.warn(
```

[229]:
```python
n_epochs = 100   # Number of optimization epochs
n_layers = 1    # Number of random layers
n_train  = 195    # Size of the train dataset
n_test   = 29     # Size of the test dataset
n_val    = 22     # Size of the validation dataset
```

```
SAVE_PATH = "/Users/h4/Desktop/quantum filters_research/
↪Quantvolution_Essential_tremor/Ablation/Quantvolution_Essential_tremor/
↪test_1/" # Data saving folder
PREPROCESS = True            # If False, skip quantum processing and load data␣
↪from SAVE_PATH
np.random.seed(0)            # Seed for NumPy random number generator
tf.random.set_seed(0)        # Seed for TensorFlow random number generator
```

[230]:
```
# Reduce dataset size
train_images = X_train[:n_train]
train_labels = y_train[:n_train]

val_images = X_val[:n_val]
val_labels = y_val[:n_val]

test_images = X_test[:n_test]
test_labels = y_test[:n_test]

# Normalize pixel values within 0 and 1
train_images = train_images / 255
val_images = val_images / 255
test_images = test_images / 255

# Add extra dimension for convolution channels
train_images = np.array(train_images[..., tf.newaxis])
val_images = np.array(val_images[..., tf.newaxis])
test_images = np.array(test_images[..., tf.newaxis])
```

[64]:
```
## Filter Quantvolution 2x2

dev = qml.device("default.qubit", wires=4)
# Random circuit parameters
rand_params = np.random.uniform(high=2 * np.pi, size=(n_layers, 4))

E =[(0,1,1.0),
    (1,2,1.0),
    (2,3,1.0),
    (3,0,1.0)]

@qml.qnode(dev)
def circuit(phi):
    # Encoding of 4 classical input values
    for edge in E:
        j = edge[0]
        k = edge[1]
        qml.Hadamard(wires=j)
        qml.Hadamard(wires=k)
```

```
        qml.IsingXX(np.pi * phi[j], wires=[j,k])
        qml.IsingZZ(np.pi * phi[j], wires=[j,k])

    # Measurement producing 4 classical output values
    return [qml.expval(qml.PauliZ(j)) for j in range(4)]

def quanv(image):
    """Convolves the input image with many applications of the same quantum␣
↪circuit."""
    out = np.zeros((108, 16, 4))

    # Loop over the coordinates of the top-left pixel of 2X2 squares
    for j in range(0, 216, 2):
        for k in range(0, 32, 2):
            # Process a squcared 2x2 region of the image with a quantum circuit
            q_results = circuit(
                [
                    image[j, k, 0],
                    image[j, k + 1, 0],
                    image[j + 1, k, 0],
                    image[j + 1, k + 1, 0]
                ]
            )
            # Assign expectation values to different channels of the output␣
↪pixel (j/2, k/2)
            for c in range(4):
                out[j // 2, k // 2, c] = q_results[c]
    return out
```

[66]:
```
import time
import numpy as np

if PREPROCESS:
    q_train_images = []
    print("Quantum pre-processing of train images:")
    start_time = time.time()

    for idx, img in enumerate(train_images):
        print("{}/{}        ".format(idx + 1, n_train), end="\r")
        q_train_images.append(quanv(img))

    q_train_images = np.asarray(q_train_images)
    np.save(SAVE_PATH + "q_train_images_test_1.npy", q_train_images)

    end_time = time.time()
    elapsed_time = end_time - start_time
    print(f"Quantum pre-processing completed in {elapsed_time:.2f} seconds")
```

```
Quantum pre-processing of train images:
1/195

/var/folders/nw/k_k0_cbj7vl_npdmryvhl53c0000gn/T/ipykernel_70899/1237346713.py:4
4: DeprecationWarning: Conversion of an array with ndim > 0 to a scalar is
deprecated, and will error in future. Ensure you extract a single element from
your array before performing this operation. (Deprecated NumPy 1.25.)
  out[j // 2, k // 2, c] = q_results[c]

Quantum pre-processing completed in 524.43 seconds
```

[67]:

```
import time
import numpy as np

if PREPROCESS:
    q_test_images = []
    print("Quantum pre-processing of test images:")
    start_time = time.time()

    for idx, img in enumerate(test_images):
        print("{}/{}        ".format(idx + 1, n_test), end="\r")
        q_test_images.append(quanv(img))

    q_test_images = np.asarray(q_test_images)
    np.save(SAVE_PATH + "q_test_images_test_1.npy", q_test_images)

    end_time = time.time()
    elapsed_time = end_time - start_time
    print(f"Quantum pre-processing completed in {elapsed_time:.2f} seconds")
```

```
Quantum pre-processing of test images:
2/29

/var/folders/nw/k_k0_cbj7vl_npdmryvhl53c0000gn/T/ipykernel_70899/1237346713.py:4
4: DeprecationWarning: Conversion of an array with ndim > 0 to a scalar is
deprecated, and will error in future. Ensure you extract a single element from
your array before performing this operation. (Deprecated NumPy 1.25.)
  out[j // 2, k // 2, c] = q_results[c]

Quantum pre-processing completed in 88.42 seconds
```

[192]:

```
import time
import numpy as np

if PREPROCESS:
    q_val_images = []
    print("Quantum pre-processing of val images:")
    start_time = time.time()
```

```
    for idx, img in enumerate(val_images):
        print("{}/{}        ".format(idx + 1, n_val), end="\r")
        q_val_images.append(quanv(img))

    q_val_images = np.asarray(q_val_images)
    np.save(SAVE_PATH + "q_val_images_test_1.npy", q_val_images)

    end_time = time.time()
    elapsed_time = end_time - start_time
    print(f"Quantum pre-processing completed in {elapsed_time:.2f} seconds")
```

```
Quantum pre-processing of val images:
Quantum pre-processing completed in 7.15 seconds
```

[69]:
```
# Load pre-processed images
q_train_images = np.load(SAVE_PATH + "q_train_images_test_1.npy")
q_val_images   = np.load(SAVE_PATH + "q_val_images_test_1.npy")
q_test_images  = np.load(SAVE_PATH + "q_test_images_test_1.npy")
```

[70]:
```
X_train = torch.tensor(q_train_images)
y_train = torch.tensor(train_labels)
X_val = torch.tensor(q_val_images)
y_val = torch.tensor(val_labels)
X_test = torch.tensor(q_test_images)
y_test = torch.tensor(test_labels)
```

[71]:
```
print(np.shape(X_train))
print(np.shape(y_train))
print(np.shape(X_val))
print(np.shape(y_val))
print(np.shape(X_test))
print(np.shape(y_test))
```

```
torch.Size([195, 108, 16, 4])
torch.Size([195, 4])
torch.Size([22, 108, 16, 4])
torch.Size([22, 4])
torch.Size([29, 108, 16, 4])
torch.Size([29, 4])
```

[74]:
```
import torch
from torch.utils.data import Dataset, DataLoader

class CustomTensorDataset(Dataset):
    """TensorDataset with support for transforms."""
    def __init__(self, tensors, transform=None):
```

```
        assert all(tensors[0].shape[0] == tensor.shape[0] for tensor in␣
↪tensors), "All tensors must have the same first dimension"
        self.tensors = tensors
        self.transform = transform

    def __getitem__(self, index):
        x = self.tensors[0][index]
        if self.transform:
            x = self.transform(x)
        y = self.tensors[1][index]
        return x, y

    def __len__(self):
        return self.tensors[0].shape[0]

# Convert to PyTorch tensors
X_train_tensor = torch.tensor(X_train, dtype=torch.float32)
y_train_tensor = torch.tensor(y_train, dtype=torch.long)
X_test_tensor = torch.tensor(X_test, dtype=torch.float32)
y_test_tensor = torch.tensor(y_test, dtype=torch.long)
X_val_tensor = torch.tensor(X_val, dtype=torch.float32)
y_val_tensor = torch.tensor(y_val, dtype=torch.long)

# Create dataset and dataloader
train_dataset = CustomTensorDataset(tensors=(X_train_tensor, y_train_tensor),␣
↪transform=None)
train_loader = DataLoader(train_dataset, batch_size=1, shuffle=True)

test_dataset = CustomTensorDataset(tensors=(X_test_tensor, y_test_tensor),␣
↪transform=None)
test_loader = DataLoader(test_dataset, batch_size=1, shuffle=True)

val_dataset = CustomTensorDataset(tensors=(X_val_tensor, y_val_tensor),␣
↪transform=None)
val_loader = DataLoader(val_dataset, batch_size=1, shuffle=True)

# Print dataset size to check
print("Train dataset size:", len(train_dataset))
print("Test dataset size:", len(test_dataset))
print("Val dataset size:", len(val_dataset))
```

```
Train dataset size: 195
Test dataset size: 29
Val dataset size: 22
```

```
/var/folders/nw/k_k0_cbj7vl_npdmryvhl53c0000gn/T/ipykernel_70899/522783547.py:22
: UserWarning: To copy construct from a tensor, it is recommended to use
sourceTensor.clone().detach() or
```

```
sourceTensor.clone().detach().requires_grad_(True), rather than
torch.tensor(sourceTensor).
  X_train_tensor = torch.tensor(X_train, dtype=torch.float32)
/var/folders/nw/k_k0_cbj7vl_npdmryvhl53c0000gn/T/ipykernel_70899/522783547.py:23
: UserWarning: To copy construct from a tensor, it is recommended to use
sourceTensor.clone().detach() or
sourceTensor.clone().detach().requires_grad_(True), rather than
torch.tensor(sourceTensor).
  y_train_tensor = torch.tensor(y_train, dtype=torch.long)
/var/folders/nw/k_k0_cbj7vl_npdmryvhl53c0000gn/T/ipykernel_70899/522783547.py:24
: UserWarning: To copy construct from a tensor, it is recommended to use
sourceTensor.clone().detach() or
sourceTensor.clone().detach().requires_grad_(True), rather than
torch.tensor(sourceTensor).
  X_test_tensor = torch.tensor(X_test, dtype=torch.float32)
/var/folders/nw/k_k0_cbj7vl_npdmryvhl53c0000gn/T/ipykernel_70899/522783547.py:25
: UserWarning: To copy construct from a tensor, it is recommended to use
sourceTensor.clone().detach() or
sourceTensor.clone().detach().requires_grad_(True), rather than
torch.tensor(sourceTensor).
  y_test_tensor = torch.tensor(y_test, dtype=torch.long)
/var/folders/nw/k_k0_cbj7vl_npdmryvhl53c0000gn/T/ipykernel_70899/522783547.py:26
: UserWarning: To copy construct from a tensor, it is recommended to use
sourceTensor.clone().detach() or
sourceTensor.clone().detach().requires_grad_(True), rather than
torch.tensor(sourceTensor).
  X_val_tensor = torch.tensor(X_val, dtype=torch.float32)
/var/folders/nw/k_k0_cbj7vl_npdmryvhl53c0000gn/T/ipykernel_70899/522783547.py:27
: UserWarning: To copy construct from a tensor, it is recommended to use
sourceTensor.clone().detach() or
sourceTensor.clone().detach().requires_grad_(True), rather than
torch.tensor(sourceTensor).
  y_val_tensor = torch.tensor(y_val, dtype=torch.long)
```

[171]:

```
import time
import pennylane as qml
import numpy as np
import torch
import torch.nn as nn
import torch.optim as optim
import torch.nn.functional as F
import random
from torch.autograd import Function
from torch.utils.data import DataLoader, TensorDataset
import matplotlib.pyplot as plt

##############################################
```

```
# Load and Prepare Your Real Dataset
##############################################
# Assume X_train, y_train, X_val, y_val, X_test, y_test are already defined.
print("X_train shape:", np.shape(X_train))
print("y_train shape:", np.shape(y_train))
print("X_val shape:", np.shape(X_val))
print("y_val shape:", np.shape(y_val))
print("X_test shape:", np.shape(X_test))
print("y_test shape:", np.shape(y_test))

# If your targets are torch.Tensors and have values 0-3, map them to binary␣
↪classes.
if isinstance(y_train, torch.Tensor):
    if y_train.max().item() > 1:
        # Map values: if value < 2 then new value is 0, else 1.
        y_train = torch.where(y_train < 2, torch.tensor(0, dtype=torch.long),␣
↪torch.tensor(1, dtype=torch.long))
        y_val   = torch.where(y_val   < 2, torch.tensor(0, dtype=torch.long),␣
↪torch.tensor(1, dtype=torch.long))
        y_test  = torch.where(y_test  < 2, torch.tensor(0, dtype=torch.long),␣
↪torch.tensor(1, dtype=torch.long))
else:
    # If they are numpy arrays:
    if np.max(y_train) > 1:
        y_train = np.where(y_train < 2, 0, 1)
        y_val   = np.where(y_val   < 2, 0, 1)
        y_test  = np.where(y_test  < 2, 0, 1)

# Convert the arrays/tensors to torch.Tensors (if not already).
if not isinstance(X_train, torch.Tensor):
    X_train_tensor = torch.tensor(X_train, dtype=torch.float32)
    y_train_tensor = torch.tensor(y_train, dtype=torch.long)
    X_val_tensor   = torch.tensor(X_val, dtype=torch.float32)
    y_val_tensor   = torch.tensor(y_val, dtype=torch.long)
    X_test_tensor  = torch.tensor(X_test, dtype=torch.float32)
    y_test_tensor  = torch.tensor(y_test, dtype=torch.long)
else:
    X_train_tensor = X_train
    y_train_tensor = y_train
    X_val_tensor   = X_val
    y_val_tensor   = y_val
    X_test_tensor  = X_test
    y_test_tensor  = y_test

# Create TensorDatasets.
train_dataset = TensorDataset(X_train_tensor, y_train_tensor)
val_dataset   = TensorDataset(X_val_tensor, y_val_tensor)
```

```
test_dataset  = TensorDataset(X_test_tensor, y_test_tensor)

# Create DataLoaders.
train_loader = DataLoader(train_dataset, batch_size=4, shuffle=True)
val_loader   = DataLoader(val_dataset, batch_size=4, shuffle=False)
test_loader  = DataLoader(test_dataset, batch_size=4, shuffle=False)

##############################################
# Define a simple PennyLane Quantum Circuit
##############################################
class QuantumCircuit:
    def __init__(self, n_qubits, shots):
        self.n_qubits = n_qubits
        self.shots = shots
        self.dev = qml.device("default.qubit", wires=n_qubits, shots=shots)

    def run(self, inputs):
        @qml.qnode(self.dev, interface="torch")
        def circuit(inputs):
            qml.RX(inputs[0], wires=0)
            qml.RX(inputs[1], wires=1)
            qml.CNOT(wires=[0, 1])
            return qml.probs(wires=[0])
        inputs = torch.tensor(inputs, dtype=torch.float32)
        result = circuit(inputs)
        return result.detach().numpy()  # returns shape (2,)

##############################################
# Define the Hybrid Quantum-Classical Layer
##############################################
class HybridFunction(Function):
    @staticmethod
    def forward(ctx, input, quantum_circuit, shift):
        ctx.shift = shift
        ctx.quantum_circuit = quantum_circuit

        input_array = input.detach().cpu().numpy().flatten()
        if input_array.shape[0] != 2:
            raise ValueError(f"Expected input to have 2 elements, but got shape␣
↪{input_array.shape} with values {input_array}")

        probabilities = ctx.quantum_circuit.run(input_array)
        probabilities_shift_plus = ctx.quantum_circuit.run(
            np.array([input_array[0] + np.pi/2 + random.uniform(1e-6, 1e-5),
                      input_array[1] + np.pi/2])
        )
        probabilities_shift_minus = ctx.quantum_circuit.run(
```

```
            np.array([input_array[0] - np.pi/2 + random.uniform(1e-6, 1e-5),
                      input_array[1] - np.pi/2])
        )

        probabilities = torch.tensor(probabilities, dtype=torch.float32)
        probabilities_shift_plus = torch.tensor(probabilities_shift_plus,␣
↪dtype=torch.float32)
        probabilities_shift_minus = torch.tensor(probabilities_shift_minus,␣
↪dtype=torch.float32)

        ctx.save_for_backward(input, probabilities, probabilities_shift_plus,␣
↪probabilities_shift_minus)
        return probabilities  # shape (2,)

    @staticmethod
    def backward(ctx, grad_output):
        input, probabilities, probabilities_shift_plus,␣
↪probabilities_shift_minus = ctx.saved_tensors
        gradient = ((probabilities_shift_plus - probabilities_shift_minus) / 2).
↪clone().detach().requires_grad_(True)
        grad = gradient.float() * grad_output.float().clone().detach().
↪requires_grad_(True)
        return grad, None, None

class Hybrid(nn.Module):
    def __init__(self, shots, shift):
        super(Hybrid, self).__init__()
        self.quantum_circuit = QuantumCircuit(n_qubits=2, shots=shots)
        self.shift = shift

    def forward(self, input):
        return HybridFunction.apply(input, self.quantum_circuit, self.shift)

##############################################
# Define the Main Network
##############################################
class Net(nn.Module):
    def __init__(self):
        super(Net, self).__init__()
        # Convolutional layers.
        self.conv1 = nn.Conv2d(108, 64, kernel_size=2, padding=(1,1))
        self.conv1_bn1 = nn.BatchNorm2d(64)
        self.conv2 = nn.Conv2d(64, 32, kernel_size=2, padding=(1,1))
        self.conv2_bn2 = nn.BatchNorm2d(32)
        self.max_pool2d = nn.MaxPool2d(2, stride=2)
        self.dropout2d = nn.Dropout2d()
```

```
        # For your images: (108,16,4)
        # Conv1: → (64,17,5) → pooling → (64,8,2)
        # Conv2: → (32,9,3) → pooling → (32,4,1) → flattened = 32*4*1 = 128.
        self.fc1 = nn.Linear(128, 40)  # flattened size is 128.
        self.fc2 = nn.Linear(40, 32)
        self.fc3 = nn.Linear(32, 16)
        self.dropout_fc = nn.Dropout(0.25)
        self.fc4 = nn.Linear(16, 8)
        self.dropout_fc2 = nn.Dropout(0.25)
        self.fc5 = nn.Linear(8, 2)
        self.hybrid = Hybrid(shots=100, shift=np.pi/2)

    def forward(self, x):
        # x: (batch, 108, 16, 4)
        x = F.relu(self.conv1(x))       # → (batch, 64, 17, 5)
        x = F.max_pool2d(x, 2)            # → (batch, 64, 8, 2)
        x = F.relu(self.conv1_bn1(x))
        x = self.dropout2d(x)
        x = F.relu(self.conv2(x))         # → (batch, 32, 9, 3)
        x = F.max_pool2d(x, 2)            # → (batch, 32, 4, 1)
        x = F.relu(self.conv2_bn2(x))
        x = self.dropout2d(x)
        x = x.view(x.size(0), -1)         # Flatten → (batch, 128)
        x = F.relu(self.fc1(x))           # → (batch, 40)
        x = self.fc2(x)                   # → (batch, 32)
        x = F.relu(self.fc3(x))           # → (batch, 16)
        x = self.dropout_fc(x)
        x = self.fc4(x)                   # → (batch, 8)
        x = F.relu(self.fc5(x))           # → (batch, 2)

        # Process each sample with the hybrid quantum layer.
        outputs = []
        for i in range(x.size(0)):
            quantum_out = self.hybrid(x[i])  # each x[i] is (2,)
            outputs.append(quantum_out.unsqueeze(0))
        out = torch.cat(outputs, dim=0)       # final output: (batch, 2)
        return out

##############################################
# Optimizer, Loss, and LR Scheduler Setup
##############################################
model = Net()
lr = 1e-4
optimizer = optim.Adam(model.parameters(), lr=lr)
loss_func = nn.CrossEntropyLoss()  # expects output shape (N, C) and target
 ↪shape (N,)
```

```python
def adjust_learning_rate_poly(optimizer, initial_lr, iteration, max_iter):
    lr = 1e-4
    if lr < 2e-5:
        lr = 2e-5
    for param_group in optimizer.param_groups:
        param_group['lr'] = lr
    return lr

###############################################
# Training, Validation, and Test Loop with Metrics and Timing
###############################################
num_epochs = 100
train_losses = []
val_losses = []
test_losses = []
train_accuracies = []
val_accuracies = []
test_accuracies = []

start_time = time.time()

for epoch in range(num_epochs):
    # --- Training ---
    model.train()
    running_loss = 0.0
    correct_train = 0
    total_train = 0
    for data, target in train_loader:
        target = target.view(-1)
        optimizer.zero_grad()
        outputs = model(data.float())  # (batch, 2)
        loss = loss_func(outputs, target.long())
        loss.backward()
        optimizer.step()

        running_loss += loss.item() * data.size(0)
        _, predicted = torch.max(outputs, 1)
        total_train += target.size(0)
        correct_train += (predicted == target).sum().item()
    epoch_train_loss = running_loss / total_train
    epoch_train_acc = correct_train / total_train * 100.0
    train_losses.append(epoch_train_loss)
    train_accuracies.append(epoch_train_acc)

    # --- Validation ---
    model.eval()
    running_val_loss = 0.0
```

```
    correct_val = 0
    total_val = 0
    with torch.no_grad():
        for data, target in val_loader:
            target = target.view(-1)
            outputs = model(data.float())
            loss = loss_func(outputs, target.long())
            running_val_loss += loss.item() * data.size(0)
            _, predicted = torch.max(outputs, 1)
            total_val += target.size(0)
            correct_val += (predicted == target).sum().item()
    epoch_val_loss = running_val_loss / total_val
    epoch_val_acc = correct_val / total_val * 100.0
    val_losses.append(epoch_val_loss)
    val_accuracies.append(epoch_val_acc)

    # --- Test Evaluation ---
    model.eval()
    running_test_loss = 0.0
    correct_test = 0
    total_test = 0
    with torch.no_grad():
        for data, target in test_loader:
            target = target.view(-1)
            outputs = model(data.float())
            loss = loss_func(outputs, target.long())
            running_test_loss += loss.item() * data.size(0)
            _, predicted = torch.max(outputs, 1)
            total_test += target.size(0)
            correct_test += (predicted == target).sum().item()
    epoch_test_loss = running_test_loss / total_test
    epoch_test_acc = correct_test / total_test * 100.0
    test_losses.append(epoch_test_loss)
    test_accuracies.append(epoch_test_acc)

    print(f"Epoch [{epoch+1}/{num_epochs}]  "
          f"Train Loss: {epoch_train_loss:.4f}, Train Acc: {epoch_train_acc:.
↪2f}%  "
          f"Val Loss: {epoch_val_loss:.4f}, Val Acc: {epoch_val_acc:.2f}%  "
          f"Test Loss: {epoch_test_loss:.4f}, Test Acc: {epoch_test_acc:.2f}%")

end_time = time.time()
total_time = end_time - start_time

##############################################
# Print Overall Metrics
##############################################
```

```
print("\n--- Overall Metrics ---")
print(f"Train Loss: Mean = {np.mean(train_losses):.4f}, Std = {np.
↪std(train_losses):.4f}")
print(f"Train Accuracy: Mean = {np.mean(train_accuracies):.2f}%, Std = {np.
↪std(train_accuracies):.2f}%")
print(f"Validation Loss: Mean = {np.mean(val_losses):.4f}, Std = {np.
↪std(val_losses):.4f}")
print(f"Validation Accuracy: Mean = {np.mean(val_accuracies):.2f}%, Std = {np.
↪std(val_accuracies):.2f}%")
print(f"Test Loss: Mean = {np.mean(test_losses):.4f}, Std = {np.
↪std(test_losses):.4f}")
print(f"Test Accuracy: Mean = {np.mean(test_accuracies):.2f}%, Std = {np.
↪std(test_accuracies):.2f}%")
print(f"Total Computation Time: {total_time:.2f} seconds")

##############################################
# Plot Loss Curves
##############################################
epochs_range = range(1, num_epochs+1)
plt.figure(figsize=(12, 5))
plt.subplot(1, 2, 1)
plt.plot(epochs_range, train_losses, label="Train Loss", marker='o')
plt.plot(epochs_range, val_losses, label="Val Loss", marker='o')
plt.plot(epochs_range, test_losses, label="Test Loss", marker='o')
plt.xlabel("Epoch")
plt.ylabel("Loss")
plt.title("Loss vs. Epoch")
plt.legend()

##############################################
# Plot Accuracy Curves
##############################################
plt.subplot(1, 2, 2)
plt.plot(epochs_range, train_accuracies, label="Train Acc", marker='o')
plt.plot(epochs_range, val_accuracies, label="Val Acc", marker='o')
plt.plot(epochs_range, test_accuracies, label="Test Acc", marker='o')
plt.xlabel("Epoch")
plt.ylabel("Accuracy (%)")
plt.title("Accuracy vs. Epoch")
plt.legend()

plt.tight_layout()
plt.show()
```

```
X_train shape: torch.Size([195, 108, 16, 4])
y_train shape: torch.Size([195])
X_val shape: torch.Size([22, 108, 16, 4])
```

```
y_val shape: torch.Size([22])
X_test shape: torch.Size([29, 108, 16, 4])
y_test shape: torch.Size([29])
Epoch [1/100]  Train Loss: 0.8170, Train Acc: 48.21%  Val Loss: 0.8414, Val Acc:
45.45%  Test Loss: 0.8257, Test Acc: 48.28%
Epoch [2/100]  Train Loss: 0.8147, Train Acc: 48.21%  Val Loss: 0.8376, Val Acc:
45.45%  Test Loss: 0.8114, Test Acc: 48.28%
Epoch [3/100]  Train Loss: 0.8093, Train Acc: 48.21%  Val Loss: 0.8423, Val Acc:
45.45%  Test Loss: 0.8067, Test Acc: 48.28%
Epoch [4/100]  Train Loss: 0.8007, Train Acc: 48.21%  Val Loss: 0.8239, Val Acc:
45.45%  Test Loss: 0.8011, Test Acc: 48.28%
Epoch [5/100]  Train Loss: 0.7692, Train Acc: 48.21%  Val Loss: 0.7946, Val Acc:
45.45%  Test Loss: 0.7531, Test Acc: 48.28%
Epoch [6/100]  Train Loss: 0.6491, Train Acc: 56.41%  Val Loss: 0.5628, Val Acc:
63.64%  Test Loss: 0.5355, Test Acc: 75.86%
Epoch [7/100]  Train Loss: 0.4833, Train Acc: 85.64%  Val Loss: 0.4478, Val Acc:
95.45%  Test Loss: 0.4141, Test Acc: 96.55%
Epoch [8/100]  Train Loss: 0.4571, Train Acc: 90.26%  Val Loss: 0.4323, Val Acc:
95.45%  Test Loss: 0.4111, Test Acc: 96.55%
Epoch [9/100]  Train Loss: 0.4281, Train Acc: 93.85%  Val Loss: 0.4051, Val Acc:
100.00%  Test Loss: 0.3932, Test Acc: 100.00%
Epoch [10/100]  Train Loss: 0.4194, Train Acc: 93.33%  Val Loss: 0.4147, Val
Acc: 95.45%  Test Loss: 0.4022, Test Acc: 96.55%
Epoch [11/100]  Train Loss: 0.4257, Train Acc: 94.87%  Val Loss: 0.3890, Val
Acc: 100.00%  Test Loss: 0.3776, Test Acc: 100.00%
Epoch [12/100]  Train Loss: 0.4201, Train Acc: 93.33%  Val Loss: 0.3922, Val
Acc: 100.00%  Test Loss: 0.3860, Test Acc: 100.00%
Epoch [13/100]  Train Loss: 0.4166, Train Acc: 93.33%  Val Loss: 0.3878, Val
Acc: 100.00%  Test Loss: 0.3816, Test Acc: 100.00%
Epoch [14/100]  Train Loss: 0.4110, Train Acc: 93.33%  Val Loss: 0.3800, Val
Acc: 100.00%  Test Loss: 0.3779, Test Acc: 96.55%
Epoch [15/100]  Train Loss: 0.3954, Train Acc: 93.85%  Val Loss: 0.3972, Val
Acc: 100.00%  Test Loss: 0.3970, Test Acc: 96.55%
Epoch [16/100]  Train Loss: 0.3891, Train Acc: 95.38%  Val Loss: 0.3963, Val
Acc: 100.00%  Test Loss: 0.3996, Test Acc: 100.00%
Epoch [17/100]  Train Loss: 0.3981, Train Acc: 94.87%  Val Loss: 0.4150, Val
Acc: 100.00%  Test Loss: 0.4095, Test Acc: 93.10%
Epoch [18/100]  Train Loss: 0.3830, Train Acc: 94.87%  Val Loss: 0.4041, Val
Acc: 100.00%  Test Loss: 0.4000, Test Acc: 93.10%
Epoch [19/100]  Train Loss: 0.3900, Train Acc: 94.87%  Val Loss: 0.4168, Val
Acc: 100.00%  Test Loss: 0.3943, Test Acc: 100.00%
Epoch [20/100]  Train Loss: 0.3943, Train Acc: 92.31%  Val Loss: 0.4097, Val
Acc: 100.00%  Test Loss: 0.4052, Test Acc: 96.55%
Epoch [21/100]  Train Loss: 0.4016, Train Acc: 94.87%  Val Loss: 0.3773, Val
Acc: 100.00%  Test Loss: 0.3717, Test Acc: 100.00%
Epoch [22/100]  Train Loss: 0.4045, Train Acc: 93.85%  Val Loss: 0.4003, Val
Acc: 100.00%  Test Loss: 0.4028, Test Acc: 96.55%
Epoch [23/100]  Train Loss: 0.3780, Train Acc: 97.44%  Val Loss: 0.3977, Val
```

Acc: 100.00% Test Loss: 0.3932, Test Acc: 100.00%
Epoch [24/100] Train Loss: 0.3951, Train Acc: 93.85% Val Loss: 0.4301, Val Acc: 95.45% Test Loss: 0.4271, Test Acc: 96.55%
Epoch [25/100] Train Loss: 0.3943, Train Acc: 91.28% Val Loss: 0.3985, Val Acc: 100.00% Test Loss: 0.3851, Test Acc: 100.00%
Epoch [26/100] Train Loss: 0.3948, Train Acc: 93.85% Val Loss: 0.4112, Val Acc: 100.00% Test Loss: 0.3976, Test Acc: 100.00%
Epoch [27/100] Train Loss: 0.3815, Train Acc: 93.85% Val Loss: 0.3731, Val Acc: 100.00% Test Loss: 0.3816, Test Acc: 100.00%
Epoch [28/100] Train Loss: 0.3987, Train Acc: 92.31% Val Loss: 0.3999, Val Acc: 100.00% Test Loss: 0.4003, Test Acc: 100.00%
Epoch [29/100] Train Loss: 0.3840, Train Acc: 95.90% Val Loss: 0.3731, Val Acc: 100.00% Test Loss: 0.3745, Test Acc: 100.00%
Epoch [30/100] Train Loss: 0.3735, Train Acc: 96.92% Val Loss: 0.3834, Val Acc: 100.00% Test Loss: 0.3779, Test Acc: 100.00%
Epoch [31/100] Train Loss: 0.3786, Train Acc: 96.92% Val Loss: 0.4059, Val Acc: 100.00% Test Loss: 0.3917, Test Acc: 100.00%
Epoch [32/100] Train Loss: 0.3775, Train Acc: 96.41% Val Loss: 0.3837, Val Acc: 100.00% Test Loss: 0.3650, Test Acc: 100.00%
Epoch [33/100] Train Loss: 0.3905, Train Acc: 94.87% Val Loss: 0.3782, Val Acc: 100.00% Test Loss: 0.3657, Test Acc: 100.00%
Epoch [34/100] Train Loss: 0.3860, Train Acc: 93.33% Val Loss: 0.4269, Val Acc: 100.00% Test Loss: 0.3952, Test Acc: 100.00%
Epoch [35/100] Train Loss: 0.3901, Train Acc: 94.36% Val Loss: 0.4138, Val Acc: 100.00% Test Loss: 0.3922, Test Acc: 100.00%
Epoch [36/100] Train Loss: 0.3761, Train Acc: 94.87% Val Loss: 0.3688, Val Acc: 100.00% Test Loss: 0.3819, Test Acc: 100.00%
Epoch [37/100] Train Loss: 0.3858, Train Acc: 95.90% Val Loss: 0.3810, Val Acc: 100.00% Test Loss: 0.3685, Test Acc: 100.00%
Epoch [38/100] Train Loss: 0.3954, Train Acc: 94.36% Val Loss: 0.4098, Val Acc: 100.00% Test Loss: 0.4008, Test Acc: 100.00%
Epoch [39/100] Train Loss: 0.3817, Train Acc: 95.38% Val Loss: 0.3985, Val Acc: 100.00% Test Loss: 0.3935, Test Acc: 100.00%
Epoch [40/100] Train Loss: 0.3700, Train Acc: 97.44% Val Loss: 0.4197, Val Acc: 100.00% Test Loss: 0.4080, Test Acc: 100.00%
Epoch [41/100] Train Loss: 0.3896, Train Acc: 94.36% Val Loss: 0.4229, Val Acc: 100.00% Test Loss: 0.4022, Test Acc: 100.00%
Epoch [42/100] Train Loss: 0.3879, Train Acc: 96.41% Val Loss: 0.3837, Val Acc: 100.00% Test Loss: 0.3780, Test Acc: 100.00%
Epoch [43/100] Train Loss: 0.3740, Train Acc: 95.90% Val Loss: 0.3899, Val Acc: 100.00% Test Loss: 0.3870, Test Acc: 100.00%
Epoch [44/100] Train Loss: 0.3785, Train Acc: 97.44% Val Loss: 0.3649, Val Acc: 100.00% Test Loss: 0.3652, Test Acc: 100.00%
Epoch [45/100] Train Loss: 0.3881, Train Acc: 93.85% Val Loss: 0.3682, Val Acc: 100.00% Test Loss: 0.3743, Test Acc: 100.00%
Epoch [46/100] Train Loss: 0.3782, Train Acc: 94.36% Val Loss: 0.3640, Val Acc: 100.00% Test Loss: 0.3639, Test Acc: 100.00%
Epoch [47/100] Train Loss: 0.3809, Train Acc: 95.38% Val Loss: 0.3977, Val

Acc: 100.00% Test Loss: 0.3831, Test Acc: 100.00%
Epoch [48/100] Train Loss: 0.3789, Train Acc: 94.36% Val Loss: 0.3934, Val Acc: 100.00% Test Loss: 0.3843, Test Acc: 100.00%
Epoch [49/100] Train Loss: 0.3764, Train Acc: 96.41% Val Loss: 0.3844, Val Acc: 100.00% Test Loss: 0.3768, Test Acc: 100.00%
Epoch [50/100] Train Loss: 0.3840, Train Acc: 94.87% Val Loss: 0.3932, Val Acc: 100.00% Test Loss: 0.3768, Test Acc: 100.00%
Epoch [51/100] Train Loss: 0.3702, Train Acc: 95.38% Val Loss: 0.3895, Val Acc: 100.00% Test Loss: 0.3830, Test Acc: 100.00%
Epoch [52/100] Train Loss: 0.3730, Train Acc: 97.95% Val Loss: 0.3970, Val Acc: 100.00% Test Loss: 0.3830, Test Acc: 100.00%
Epoch [53/100] Train Loss: 0.3791, Train Acc: 96.92% Val Loss: 0.3928, Val Acc: 100.00% Test Loss: 0.3787, Test Acc: 100.00%
Epoch [54/100] Train Loss: 0.3575, Train Acc: 98.97% Val Loss: 0.3938, Val Acc: 100.00% Test Loss: 0.3784, Test Acc: 100.00%
Epoch [55/100] Train Loss: 0.3748, Train Acc: 95.38% Val Loss: 0.3982, Val Acc: 100.00% Test Loss: 0.3806, Test Acc: 100.00%
Epoch [56/100] Train Loss: 0.3755, Train Acc: 95.90% Val Loss: 0.3734, Val Acc: 100.00% Test Loss: 0.3563, Test Acc: 100.00%
Epoch [57/100] Train Loss: 0.3645, Train Acc: 96.92% Val Loss: 0.3744, Val Acc: 100.00% Test Loss: 0.3675, Test Acc: 100.00%
Epoch [58/100] Train Loss: 0.3737, Train Acc: 96.41% Val Loss: 0.3757, Val Acc: 100.00% Test Loss: 0.3590, Test Acc: 100.00%
Epoch [59/100] Train Loss: 0.3789, Train Acc: 94.87% Val Loss: 0.3979, Val Acc: 100.00% Test Loss: 0.3804, Test Acc: 100.00%
Epoch [60/100] Train Loss: 0.3741, Train Acc: 95.90% Val Loss: 0.3927, Val Acc: 100.00% Test Loss: 0.3817, Test Acc: 100.00%
Epoch [61/100] Train Loss: 0.3700, Train Acc: 95.90% Val Loss: 0.4042, Val Acc: 100.00% Test Loss: 0.4039, Test Acc: 96.55%
Epoch [62/100] Train Loss: 0.3770, Train Acc: 94.87% Val Loss: 0.4088, Val Acc: 100.00% Test Loss: 0.3904, Test Acc: 100.00%
Epoch [63/100] Train Loss: 0.3764, Train Acc: 95.90% Val Loss: 0.3781, Val Acc: 100.00% Test Loss: 0.3798, Test Acc: 100.00%
Epoch [64/100] Train Loss: 0.3749, Train Acc: 96.41% Val Loss: 0.3934, Val Acc: 100.00% Test Loss: 0.3934, Test Acc: 100.00%
Epoch [65/100] Train Loss: 0.3695, Train Acc: 95.38% Val Loss: 0.3837, Val Acc: 100.00% Test Loss: 0.3824, Test Acc: 100.00%
Epoch [66/100] Train Loss: 0.3689, Train Acc: 96.41% Val Loss: 0.3794, Val Acc: 100.00% Test Loss: 0.3729, Test Acc: 100.00%
Epoch [67/100] Train Loss: 0.3709, Train Acc: 96.92% Val Loss: 0.4020, Val Acc: 100.00% Test Loss: 0.3826, Test Acc: 100.00%
Epoch [68/100] Train Loss: 0.3721, Train Acc: 96.41% Val Loss: 0.4206, Val Acc: 100.00% Test Loss: 0.4081, Test Acc: 100.00%
Epoch [69/100] Train Loss: 0.3625, Train Acc: 96.41% Val Loss: 0.3901, Val Acc: 100.00% Test Loss: 0.3818, Test Acc: 100.00%
Epoch [70/100] Train Loss: 0.3684, Train Acc: 96.92% Val Loss: 0.3991, Val Acc: 100.00% Test Loss: 0.3814, Test Acc: 100.00%
Epoch [71/100] Train Loss: 0.3722, Train Acc: 96.92% Val Loss: 0.3833, Val

```
Acc: 100.00%  Test Loss: 0.3804, Test Acc: 100.00%
Epoch [72/100]  Train Loss: 0.3672, Train Acc: 97.44%  Val Loss: 0.3697, Val
Acc: 100.00%  Test Loss: 0.3703, Test Acc: 100.00%
Epoch [73/100]  Train Loss: 0.3680, Train Acc: 96.41%  Val Loss: 0.3853, Val
Acc: 100.00%  Test Loss: 0.3719, Test Acc: 100.00%
Epoch [74/100]  Train Loss: 0.3689, Train Acc: 96.41%  Val Loss: 0.3791, Val
Acc: 100.00%  Test Loss: 0.3705, Test Acc: 100.00%
Epoch [75/100]  Train Loss: 0.3634, Train Acc: 98.46%  Val Loss: 0.3873, Val
Acc: 100.00%  Test Loss: 0.3783, Test Acc: 100.00%
Epoch [76/100]  Train Loss: 0.3652, Train Acc: 96.41%  Val Loss: 0.4135, Val
Acc: 100.00%  Test Loss: 0.4014, Test Acc: 100.00%
Epoch [77/100]  Train Loss: 0.3659, Train Acc: 97.95%  Val Loss: 0.3990, Val
Acc: 100.00%  Test Loss: 0.3901, Test Acc: 100.00%
Epoch [78/100]  Train Loss: 0.3698, Train Acc: 96.41%  Val Loss: 0.3893, Val
Acc: 100.00%  Test Loss: 0.3740, Test Acc: 100.00%
Epoch [79/100]  Train Loss: 0.3764, Train Acc: 93.33%  Val Loss: 0.3923, Val
Acc: 100.00%  Test Loss: 0.3784, Test Acc: 100.00%
Epoch [80/100]  Train Loss: 0.3667, Train Acc: 96.92%  Val Loss: 0.4263, Val
Acc: 100.00%  Test Loss: 0.4019, Test Acc: 100.00%
Epoch [81/100]  Train Loss: 0.3472, Train Acc: 99.49%  Val Loss: 0.3803, Val
Acc: 100.00%  Test Loss: 0.3738, Test Acc: 100.00%
Epoch [82/100]  Train Loss: 0.3636, Train Acc: 98.97%  Val Loss: 0.3939, Val
Acc: 100.00%  Test Loss: 0.3744, Test Acc: 100.00%
Epoch [83/100]  Train Loss: 0.3605, Train Acc: 98.97%  Val Loss: 0.4050, Val
Acc: 100.00%  Test Loss: 0.3785, Test Acc: 100.00%
Epoch [84/100]  Train Loss: 0.3644, Train Acc: 96.92%  Val Loss: 0.3952, Val
Acc: 100.00%  Test Loss: 0.3745, Test Acc: 100.00%
Epoch [85/100]  Train Loss: 0.3581, Train Acc: 99.49%  Val Loss: 0.3977, Val
Acc: 100.00%  Test Loss: 0.3776, Test Acc: 100.00%
Epoch [86/100]  Train Loss: 0.3566, Train Acc: 98.46%  Val Loss: 0.4034, Val
Acc: 100.00%  Test Loss: 0.3798, Test Acc: 100.00%
Epoch [87/100]  Train Loss: 0.3621, Train Acc: 97.44%  Val Loss: 0.4021, Val
Acc: 100.00%  Test Loss: 0.3757, Test Acc: 100.00%
Epoch [88/100]  Train Loss: 0.3556, Train Acc: 97.95%  Val Loss: 0.4011, Val
Acc: 100.00%  Test Loss: 0.3827, Test Acc: 100.00%
Epoch [89/100]  Train Loss: 0.3535, Train Acc: 99.49%  Val Loss: 0.3969, Val
Acc: 100.00%  Test Loss: 0.3845, Test Acc: 100.00%
Epoch [90/100]  Train Loss: 0.3517, Train Acc: 98.97%  Val Loss: 0.4027, Val
Acc: 100.00%  Test Loss: 0.3832, Test Acc: 100.00%
Epoch [91/100]  Train Loss: 0.3556, Train Acc: 98.46%  Val Loss: 0.3815, Val
Acc: 100.00%  Test Loss: 0.3787, Test Acc: 100.00%
Epoch [92/100]  Train Loss: 0.3558, Train Acc: 99.49%  Val Loss: 0.3936, Val
Acc: 100.00%  Test Loss: 0.3892, Test Acc: 100.00%
Epoch [93/100]  Train Loss: 0.3533, Train Acc: 98.97%  Val Loss: 0.4163, Val
Acc: 100.00%  Test Loss: 0.4011, Test Acc: 100.00%
Epoch [94/100]  Train Loss: 0.3465, Train Acc: 99.49%  Val Loss: 0.4023, Val
Acc: 100.00%  Test Loss: 0.3899, Test Acc: 100.00%
Epoch [95/100]  Train Loss: 0.3624, Train Acc: 98.97%  Val Loss: 0.3841, Val
```

```
Acc: 100.00%  Test Loss: 0.3772, Test Acc: 100.00%
Epoch [96/100]  Train Loss: 0.3546, Train Acc: 99.49%  Val Loss: 0.3918, Val
Acc: 100.00%  Test Loss: 0.3781, Test Acc: 100.00%
Epoch [97/100]  Train Loss: 0.3588, Train Acc: 98.46%  Val Loss: 0.4100, Val
Acc: 100.00%  Test Loss: 0.3885, Test Acc: 100.00%
Epoch [98/100]  Train Loss: 0.3562, Train Acc: 98.46%  Val Loss: 0.4013, Val
Acc: 100.00%  Test Loss: 0.3895, Test Acc: 100.00%
Epoch [99/100]  Train Loss: 0.3548, Train Acc: 98.97%  Val Loss: 0.4068, Val
Acc: 100.00%  Test Loss: 0.3875, Test Acc: 100.00%
Epoch [100/100]  Train Loss: 0.3620, Train Acc: 96.92%  Val Loss: 0.4047, Val
Acc: 100.00%  Test Loss: 0.3950, Test Acc: 100.00%

--- Overall Metrics ---
Train Loss: Mean = 0.4027, Std = 0.0979
Train Accuracy: Mean = 93.19%, Std = 11.27%
Validation Loss: Mean = 0.4192, Std = 0.0965
Validation Accuracy: Mean = 96.73%, Std = 12.33%
Test Loss: Mean = 0.4075, Std = 0.0922
Test Accuracy: Mean = 96.72%, Std = 11.44%
Total Computation Time: 65.76 seconds
```

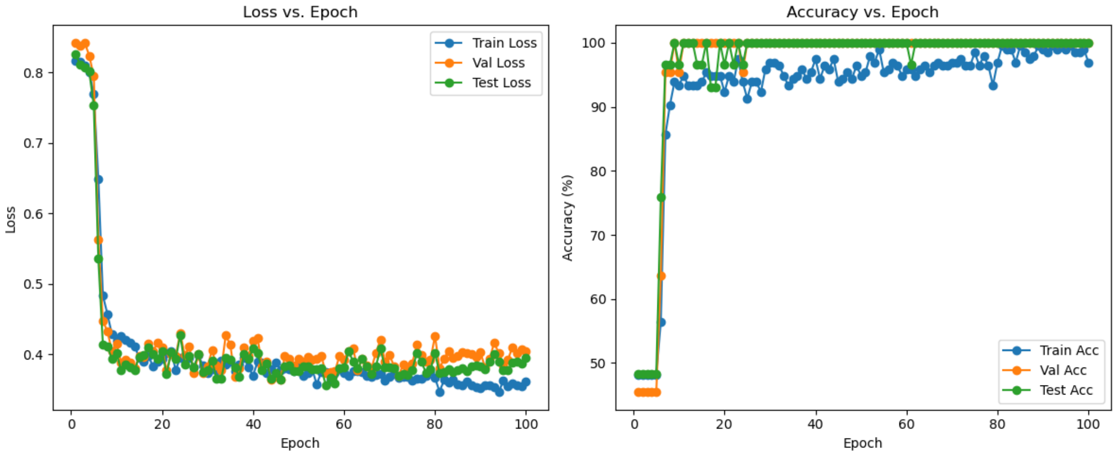

1.6 test 2. Quantvolution + Quantfilter 4x4

[406]:
```
import numpy as np
import matplotlib.pyplot as plt
import os
import cv2
from tqdm import tqdm
from PIL import Image
from numpy import asarray
```

```python
from matplotlib import image

IMG_SIZE_1 = 217
IMG_SIZE_2 = 33
DATADIR = "/Users/h4/Desktop/quantum filters_research/
↪Quantvolution_Essential_tremor/Ablation/images_split"

classes = {
    "train": ["Class1_Bad", "Class2_Good", "Class3_Right", "Class4_Left"],
    "val": ["Class1_Bad", "Class2_Good", "Class3_Right", "Class4_Left"],
    "test": ["Class1_Bad", "Class2_Good", "Class3_Right", "Class4_Left"]
}

def create_data(split):
    dataset = {class_name: [] for class_name in classes[split]}
    for i, class_name in enumerate(classes[split]):
        path = os.path.join(DATADIR, split, class_name)
        for img in tqdm(os.listdir(path), desc=f"Processing {split}␣
↪{class_name}"):
            tipo = os.path.splitext(img)[1].lower()
            if tipo in ['.jpg', '.jpeg', '.png', '.gif']:
                try:
                    img_array = asarray(Image.open(os.path.join(path, img)).
↪convert('L'))
                    new_array = cv2.resize(img_array, (IMG_SIZE_2, IMG_SIZE_1))
                    dataset[class_name].append([new_array, i])
                except Exception as e:
                    print(f"Error processing {img}: {e}")
    return dataset

train_data = create_data("train")
val_data = create_data("val")
test_data = create_data("test")

def prepare_X_y(data):
    X, y = [], []
    for class_name in data:
        for features, label in data[class_name]:
            X.append(features)
            y.append(label)
    X = np.array(X).reshape(-1, IMG_SIZE_1, IMG_SIZE_2, 1)
    y = np.array(y)
    return X, y

def unison_shuffled_copies(a, b):
    assert len(a) == len(b)
    p = np.random.permutation(len(a))
```

```
    return a[p], b[p]

X_train, y_train = prepare_X_y(train_data)
X_val, y_val = prepare_X_y(val_data)
X_test, y_test = prepare_X_y(test_data)

X_train, y_train = unison_shuffled_copies(X_train, y_train)
X_val, y_val = unison_shuffled_copies(X_val, y_val)
X_test, y_test = unison_shuffled_copies(X_test, y_test)

print("Training data shape:", X_train.shape)
print("Validation data shape:", X_val.shape)
print("Test data shape:", X_test.shape)

plt.imshow(X_train[0].squeeze(), cmap='gray')
plt.show()
```

```
Processing train Class1_Bad: 100%|        | 47/47 [00:00<00:00, 3415.50it/s]
Processing train Class2_Good: 100%|       | 47/47 [00:00<00:00, 5209.49it/s]
Processing train Class3_Right: 100%|      | 48/48 [00:00<00:00, 5788.57it/s]
Processing train Class4_Left: 100%|       | 53/53 [00:00<00:00, 5827.41it/s]
Processing val Class1_Bad: 100%|          | 5/5 [00:00<00:00, 3657.40it/s]
Processing val Class2_Good: 100%|         | 5/5 [00:00<00:00, 3349.01it/s]
Processing val Class3_Right: 100%|        | 6/6 [00:00<00:00, 4051.16it/s]
Processing val Class4_Left: 100%|         | 6/6 [00:00<00:00, 4720.66it/s]
Processing test Class1_Bad: 100%|         | 7/7 [00:00<00:00, 4797.41it/s]
Processing test Class2_Good: 100%|        | 7/7 [00:00<00:00, 4199.70it/s]
Processing test Class3_Right: 100%|       | 7/7 [00:00<00:00, 4755.45it/s]
Processing test Class4_Left: 100%|        | 8/8 [00:00<00:00, 4776.43it/s]

Training data shape: (195, 217, 33, 1)
Validation data shape: (22, 217, 33, 1)
Test data shape: (29, 217, 33, 1)
```

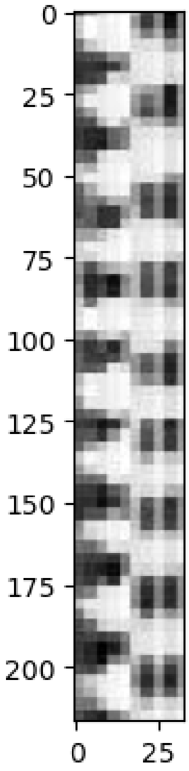

[407]:
```
train_filter = np.isin(y_train, [0, 1, 2, 3])
test_filter = np.isin(y_test, [0, 1, 2, 3])
val_filter = np.isin(y_val, [0, 1, 2, 3])

X_train, l_train = X_train[train_filter], y_train[train_filter]
X_test, l_test = X_test[test_filter], y_test[test_filter]
X_val, l_val = X_val[val_filter], y_val[val_filter]
```

[408]:
```
print(np.shape(X_train))
print(np.shape(y_train))
print(np.shape(X_val))
print(np.shape(y_val))
print(np.shape(X_test))
print(np.shape(y_test))
```

```
(195, 217, 33, 1)
(195,)
(22, 217, 33, 1)
(22,)
(29, 217, 33, 1)
(29,)
```

[409]:
```
from numpy import array
from numpy import argmax
from sklearn.preprocessing import LabelEncoder
from sklearn.preprocessing import OneHotEncoder
label_encoder = LabelEncoder()
integer_encoded = label_encoder.fit_transform(y_train)
# binary encode
onehot_encoder = OneHotEncoder(sparse=False)
integer_encoded = integer_encoded.reshape(len(integer_encoded), 1)
y_train = onehot_encoder.fit_transform(integer_encoded)

label_encoder = LabelEncoder()
integer_encoded = label_encoder.fit_transform(y_test)
# binary encode
onehot_encoder = OneHotEncoder(sparse=False)
integer_encoded = integer_encoded.reshape(len(integer_encoded), 1)
y_test = onehot_encoder.fit_transform(integer_encoded)

label_encoder = LabelEncoder()
integer_encoded = label_encoder.fit_transform(y_val)
# binary encode
onehot_encoder = OneHotEncoder(sparse=False)
integer_encoded = integer_encoded.reshape(len(integer_encoded), 1)
y_val = onehot_encoder.fit_transform(integer_encoded)
```

```
/Applications/anaconda3/lib/python3.11/site-
packages/sklearn/preprocessing/_encoders.py:868: FutureWarning: `sparse` was
renamed to `sparse_output` in version 1.2 and will be removed in 1.4.
`sparse_output` is ignored unless you leave `sparse` to its default value.
  warnings.warn(
/Applications/anaconda3/lib/python3.11/site-
packages/sklearn/preprocessing/_encoders.py:868: FutureWarning: `sparse` was
renamed to `sparse_output` in version 1.2 and will be removed in 1.4.
`sparse_output` is ignored unless you leave `sparse` to its default value.
  warnings.warn(
/Applications/anaconda3/lib/python3.11/site-
packages/sklearn/preprocessing/_encoders.py:868: FutureWarning: `sparse` was
renamed to `sparse_output` in version 1.2 and will be removed in 1.4.
`sparse_output` is ignored unless you leave `sparse` to its default value.
  warnings.warn(
```

[410]:
```
n_epochs = 100   # Number of optimization epochs
n_layers = 1    # Number of random layers
n_train  = 195    # Size of the train dataset
n_test   = 29     # Size of the test dataset
n_val    = 22     # Size of the validation dataset
```

```
SAVE_PATH = "/Users/h4/Desktop/quantum filters_research/
↪Quantvolution_Essential_tremor/Ablation/Quantvolution_Essential_tremor/
↪test_2/" # Data saving folder
PREPROCESS = True           # If False, skip quantum processing and load data␣
↪from SAVE_PATH
np.random.seed(0)           # Seed for NumPy random number generator
tf.random.set_seed(0)       # Seed for TensorFlow random number generator
```

[411]:
```
# Reduce dataset size
train_images = X_train[:n_train]
train_labels = y_train[:n_train]

val_images = X_val[:n_val]
val_labels = y_val[:n_val]

test_images = X_test[:n_test]
test_labels = y_test[:n_test]

# Normalize pixel values within 0 and 1
train_images = train_images / 255
val_images = val_images / 255
test_images = test_images / 255

# Add extra dimension for convolution channels
train_images = np.array(train_images[..., tf.newaxis])
val_images = np.array(val_images[..., tf.newaxis])
test_images = np.array(test_images[..., tf.newaxis])
```

[412]:
```
# Assume X_train, y_train, X_val, y_val, X_test, y_test are already defined.
print("train_images shape:", np.shape(train_images))
print("train_labels shape:", np.shape(train_labels))
print("val_images shape:", np.shape(val_images))
print("val_labels shape:", np.shape(val_labels))
print("test_images shape:", np.shape(test_images))
print("test_labels shape:", np.shape(test_labels))
```

```
train_images shape: (195, 217, 33, 1, 1)
train_labels shape: (195, 4)
val_images shape: (22, 217, 33, 1, 1)
val_labels shape: (22, 4)
test_images shape: (29, 217, 33, 1, 1)
test_labels shape: (29, 4)
```

[413]:
```
def quanv(image, qc):
    """
    Convolve the input image with the same quantum circuit over non-overlapping␣
↪4x4 windows.
```

```
    For each 4x4 window (16 pixel values), we:
      1. Split the 16 values into two groups of 8.
      2. For each group, split it into two groups of 4 and compute the mean.
         This yields two parameters per group.
      3. Run the quantum circuit (via qc.run) on each 2-element parameter␣
↪vector,
         which returns 2 values.
      4. Concatenate the two results to form a 4-element output for that window.

    Parameters:
      image: a NumPy array with shape (..., height, width, 1) in HWC format.
             (Extra trailing singleton dimensions are removed.)
      qc: an instance of your QuantumCircuit class.

    Returns:
      out: a NumPy array of shape (h//4, w//4, 4), where each 4x4 window has␣
↪been processed.
    """
    # Remove extra trailing singleton dimensions (e.g. if image is␣
↪(217,33,1,1), squeeze to (217,33,1))
    while image.ndim > 3 and image.shape[-1] == 1:
        image = np.squeeze(image, axis=-1)

    # Now image should have shape (H, W, 1)
    h, w, c = image.shape[-3:]
    if c != 1:
        raise ValueError(f"Expected image with 1 channel, got {c} channels.")

    # Compute number of complete 4x4 windows.
    out_h = h // 4
    out_w = w // 4
    # Allocate output array: each 4x4 window yields 4 output values.
    out = np.zeros((out_h, out_w, 4))

    # Loop over each window index.
    for i in range(out_h):
        for j in range(out_w):
            start_row = i * 4
            start_col = j * 4
            # Extract the 4x4 patch (shape (4,4,1)) and flatten to 16 values.
            patch = image[start_row:start_row+4, start_col:start_col+4, 0]
            patch_flat = patch.flatten()  # length 16

            # Split the 16 values into two groups (first 8 and last 8).
            group1 = patch_flat[:8]
            group2 = patch_flat[8:]
```

```python
            # For group1, split into two halves and average each.
            theta0 = np.mean(group1[:4])
            theta1 = np.mean(group1[4:])
            # For group2, do the same.
            theta2 = np.mean(group2[:4])
            theta3 = np.mean(group2[4:])

            # Prepare parameter vectors.
            thetas1 = [theta0, theta1]
            thetas2 = [theta2, theta3]

            # Evaluate the quantum circuit on each parameter vector.
            q_out1 = qc.run(thetas1)  # should return a 2-element array
            q_out2 = qc.run(thetas2)  # should return a 2-element array

            # Concatenate the two outputs to obtain 4 values.
            q_out = np.concatenate((np.array(q_out1).flatten(), np.
 ↪array(q_out2).flatten()))

            # Assign the 4-element output to the corresponding window.
            out[i, j, :] = q_out
    return out
```

[414]:
```python
import time
import numpy as np

if PREPROCESS:
    # Create an instance of your QuantumCircuit.
    qc = QuantumCircuit(n_qubits=2, shots=100)

    q_train_images = []
    print("Quantum pre-processing of train images:")
    start_time = time.time()

    for idx, img in enumerate(train_images):
        print("{}/{}        ".format(idx + 1, n_train), end="\r")
        processed_img = quanv(img, qc)
        q_train_images.append(processed_img)

    q_train_images = np.asarray(q_train_images)
    np.save(SAVE_PATH + "q_train_images_test_2.npy", q_train_images)

    end_time = time.time()
    elapsed_time = end_time - start_time
    print(f"Quantum pre-processing completed in {elapsed_time:.2f} seconds")
```

```
Quantum pre-processing of train images:
Quantum pre-processing completed in 93.86 seconds
```

[415]:
```python
import time
import numpy as np

if PREPROCESS:
    # Create an instance of your QuantumCircuit.
    qc = QuantumCircuit(n_qubits=2, shots=100)

    q_test_images = []
    print("Quantum pre-processing of test images:")
    start_time = time.time()

    for idx, img in enumerate(test_images):
        print("{}/{}        ".format(idx + 1, n_test), end="\r")
        processed_img = quanv(img, qc)
        q_test_images.append(processed_img)

    q_test_images = np.asarray(q_test_images)
    np.save(SAVE_PATH + "q_test_images_test_2.npy", q_test_images)

    end_time = time.time()
    elapsed_time = end_time - start_time
    print(f"Quantum pre-processing completed in {elapsed_time:.2f} seconds")
```

```
Quantum pre-processing of test images:
Quantum pre-processing completed in 13.78 seconds
```

[416]:
```python
import time
import numpy as np

if PREPROCESS:
    # Create an instance of your QuantumCircuit.
    qc = QuantumCircuit(n_qubits=2, shots=100)

    q_val_images = []
    print("Quantum pre-processing of val images:")
    start_time = time.time()

    for idx, img in enumerate(val_images):
        print("{}/{}        ".format(idx + 1, n_val), end="\r")
        processed_img = quanv(img, qc)
        q_val_images.append(processed_img)

    q_val_images = np.asarray(q_val_images)
    np.save(SAVE_PATH + "q_val_images_test_2.npy", q_val_images)
```

```
    end_time = time.time()
    elapsed_time = end_time - start_time
    print(f"Quantum pre-processing completed in {elapsed_time:.2f} seconds")
```

```
Quantum pre-processing of val images:
Quantum pre-processing completed in 10.37 seconds
```

[417]:
```
# Load pre-processed images
q_train_images = np.load(SAVE_PATH + "q_train_images_test_2.npy")
q_val_images   = np.load(SAVE_PATH + "q_val_images_test_2.npy")
q_test_images  = np.load(SAVE_PATH + "q_test_images_test_2.npy")
```

[418]:
```
X_train = torch.tensor(q_train_images)
y_train = torch.tensor(train_labels)
X_val = torch.tensor(q_val_images)
y_val = torch.tensor(val_labels)
X_test = torch.tensor(q_test_images)
y_test = torch.tensor(test_labels)
```

[419]:
```
# Assume X_train, y_train, X_val, y_val, X_test, y_test are already defined.
print("X_train shape:", np.shape(X_train))
print("y_train shape:", np.shape(y_train))
print("X_val shape:", np.shape(X_val))
print("y_val shape:", np.shape(y_val))
print("X_test shape:", np.shape(X_test))
print("y_test shape:", np.shape(y_test))
```

```
X_train shape: torch.Size([195, 54, 8, 4])
y_train shape: torch.Size([195, 4])
X_val shape: torch.Size([22, 54, 8, 4])
y_val shape: torch.Size([22, 4])
X_test shape: torch.Size([29, 54, 8, 4])
y_test shape: torch.Size([29, 4])
```

[420]:
```
import torch
from torch.utils.data import Dataset, DataLoader

class CustomTensorDataset(Dataset):
    """TensorDataset with support for transforms."""
    def __init__(self, tensors, transform=None):
        assert all(tensors[0].shape[0] == tensor.shape[0] for tensor in␣
↪tensors), "All tensors must have the same first dimension"
        self.tensors = tensors
        self.transform = transform

    def __getitem__(self, index):
```

```python
        x = self.tensors[0][index]
        if self.transform:
            x = self.transform(x)
        y = self.tensors[1][index]
        return x, y

    def __len__(self):
        return self.tensors[0].shape[0]

# Convert to PyTorch tensors
X_train_tensor = torch.tensor(X_train, dtype=torch.float32)
y_train_tensor = torch.tensor(y_train, dtype=torch.long)
X_test_tensor = torch.tensor(X_test, dtype=torch.float32)
y_test_tensor = torch.tensor(y_test, dtype=torch.long)
X_val_tensor = torch.tensor(X_val, dtype=torch.float32)
y_val_tensor = torch.tensor(y_val, dtype=torch.long)

# Create dataset and dataloader
train_dataset = CustomTensorDataset(tensors=(X_train_tensor, y_train_tensor),␣
 ↪transform=None)
train_loader = DataLoader(train_dataset, batch_size=1, shuffle=True)

test_dataset = CustomTensorDataset(tensors=(X_test_tensor, y_test_tensor),␣
 ↪transform=None)
test_loader = DataLoader(test_dataset, batch_size=1, shuffle=True)

val_dataset = CustomTensorDataset(tensors=(X_val_tensor, y_val_tensor),␣
 ↪transform=None)
val_loader = DataLoader(val_dataset, batch_size=1, shuffle=True)

# Print dataset size to check
print("Train dataset size:", len(train_dataset))
print("Test dataset size:", len(test_dataset))
print("Val dataset size:", len(val_dataset))
```

```
Train dataset size: 195
Test dataset size: 29
Val dataset size: 22
```

```
/var/folders/nw/k_k0_cbj7vl_npdmryvhl53c0000gn/T/ipykernel_70899/522783547.py:22
: UserWarning: To copy construct from a tensor, it is recommended to use
sourceTensor.clone().detach() or
sourceTensor.clone().detach().requires_grad_(True), rather than
torch.tensor(sourceTensor).
  X_train_tensor = torch.tensor(X_train, dtype=torch.float32)
/var/folders/nw/k_k0_cbj7vl_npdmryvhl53c0000gn/T/ipykernel_70899/522783547.py:23
: UserWarning: To copy construct from a tensor, it is recommended to use
sourceTensor.clone().detach() or
```

```
sourceTensor.clone().detach().requires_grad_(True), rather than
torch.tensor(sourceTensor).
  y_train_tensor = torch.tensor(y_train, dtype=torch.long)
/var/folders/nw/k_k0_cbj7vl_npdmryvhl53c0000gn/T/ipykernel_70899/522783547.py:24
: UserWarning: To copy construct from a tensor, it is recommended to use
sourceTensor.clone().detach() or
sourceTensor.clone().detach().requires_grad_(True), rather than
torch.tensor(sourceTensor).
  X_test_tensor = torch.tensor(X_test, dtype=torch.float32)
/var/folders/nw/k_k0_cbj7vl_npdmryvhl53c0000gn/T/ipykernel_70899/522783547.py:25
: UserWarning: To copy construct from a tensor, it is recommended to use
sourceTensor.clone().detach() or
sourceTensor.clone().detach().requires_grad_(True), rather than
torch.tensor(sourceTensor).
  y_test_tensor = torch.tensor(y_test, dtype=torch.long)
/var/folders/nw/k_k0_cbj7vl_npdmryvhl53c0000gn/T/ipykernel_70899/522783547.py:26
: UserWarning: To copy construct from a tensor, it is recommended to use
sourceTensor.clone().detach() or
sourceTensor.clone().detach().requires_grad_(True), rather than
torch.tensor(sourceTensor).
  X_val_tensor = torch.tensor(X_val, dtype=torch.float32)
/var/folders/nw/k_k0_cbj7vl_npdmryvhl53c0000gn/T/ipykernel_70899/522783547.py:27
: UserWarning: To copy construct from a tensor, it is recommended to use
sourceTensor.clone().detach() or
sourceTensor.clone().detach().requires_grad_(True), rather than
torch.tensor(sourceTensor).
  y_val_tensor = torch.tensor(y_val, dtype=torch.long)
```

[438]:
```
import time
import pennylane as qml
import numpy as np
import torch
import torch.nn as nn
import torch.optim as optim
import torch.nn.functional as F
import random
from torch.autograd import Function
from torch.utils.data import DataLoader, TensorDataset
import matplotlib.pyplot as plt

# ------------------------------ #
# Load and Prepare Your Dataset
# ------------------------------ #
# Simulate a dataset (replace with real data)
X_train = torch.randn(195, 54, 8, 4)
y_train = torch.randint(0, 4, (195, 4))  # Simulating one-hot encoding
X_val = torch.randn(22, 54, 8, 4)
```

```
y_val = torch.randint(0, 4, (22, 4))
X_test = torch.randn(29, 54, 8, 4)
y_test = torch.randint(0, 4, (29, 4))

# Convert one-hot encoded labels to class indices
y_train = y_train.argmax(dim=1)
y_val = y_val.argmax(dim=1)
y_test = y_test.argmax(dim=1)

# Convert multi-class (0,1,2,3) into binary classification (0,1)
y_train = torch.where(y_train < 2, torch.tensor(0, dtype=torch.long), torch.
 ↪tensor(1, dtype=torch.long))
y_val = torch.where(y_val < 2, torch.tensor(0, dtype=torch.long), torch.
 ↪tensor(1, dtype=torch.long))
y_test = torch.where(y_test < 2, torch.tensor(0, dtype=torch.long), torch.
 ↪tensor(1, dtype=torch.long))


X_train_tensor = X_train.clone().detach()
y_train_tensor = y_train.clone().detach()
X_val_tensor = X_val.clone().detach()
y_val_tensor = y_val.clone().detach()
X_test_tensor = X_test.clone().detach()
y_test_tensor = y_test.clone().detach()


# Create TensorDatasets
train_dataset = TensorDataset(X_train_tensor, y_train_tensor)
val_dataset = TensorDataset(X_val_tensor, y_val_tensor)
test_dataset = TensorDataset(X_test_tensor, y_test_tensor)

# Create DataLoaders
train_loader = DataLoader(train_dataset, batch_size=4, shuffle=True)
val_loader = DataLoader(val_dataset, batch_size=4, shuffle=False)
test_loader = DataLoader(test_dataset, batch_size=4, shuffle=False)


##############################################
# Define a simple PennyLane Quantum Circuit
##############################################
class QuantumCircuit:
    def __init__(self, n_qubits, shots):
        self.n_qubits = n_qubits
        self.shots = shots
        self.dev = qml.device("default.qubit", wires=n_qubits, shots=shots)

    def run(self, inputs):
```

```
        @qml.qnode(self.dev, interface="torch")
        def circuit(inputs):
            qml.RX(inputs[0], wires=0)
            qml.RX(inputs[1], wires=1)
            qml.CNOT(wires=[0, 1])
            return qml.probs(wires=[0])
        inputs = torch.tensor(inputs, dtype=torch.float32)
        result = circuit(inputs)
        return result.detach().numpy()  # returns shape (2,)

###############################################
# Define the Hybrid Quantum-Classical Layer
###############################################
class QuantumCircuit:
    def __init__(self, n_qubits, shots):
        self.n_qubits = n_qubits
        self.shots = shots
        self.dev = qml.device("default.qubit", wires=n_qubits, shots=shots)

    def run(self, inputs):
        @qml.qnode(self.dev, interface="torch")
        def circuit(inputs):
            qml.RX(inputs[0], wires=0)
            qml.RX(inputs[1], wires=1)
            qml.CNOT(wires=[0, 1])
            return qml.probs(wires=[0])
        return circuit(inputs)

class HybridFunction(Function):
    @staticmethod
    def forward(ctx, input, quantum_circuit):
        ctx.quantum_circuit = quantum_circuit

        input_array = input.detach().cpu().numpy().flatten()
        if input_array.shape[0] != 2:
            raise ValueError(f"Expected input to have 2 elements, but got shape␣
↪{input_array.shape}")

        probabilities = ctx.quantum_circuit.run(input_array)
        probabilities = torch.tensor(probabilities, dtype=torch.float32,␣
↪requires_grad=True)  #  Keeps gradients
        ctx.save_for_backward(input, probabilities)
        return probabilities

    @staticmethod
    def backward(ctx, grad_output):
        input, probabilities = ctx.saved_tensors
```

```
        grad = torch.ones_like(input) * grad_output  #  Ensure gradients are␣
↪passed
        return grad, None

class Hybrid(nn.Module):
    def __init__(self, shots):
        super(Hybrid, self).__init__()
        self.quantum_circuit = QuantumCircuit(n_qubits=2, shots=shots)

    def forward(self, input):
        return HybridFunction.apply(input, self.quantum_circuit)

##############################################
# Define the Main Neural Network
##############################################
class Net(nn.Module):
    def __init__(self):
        super(Net, self).__init__()
        self.conv1 = nn.Conv2d(54, 40, kernel_size=2, padding=1)
        self.bn1 = nn.BatchNorm2d(40)
        self.conv2 = nn.Conv2d(40, 16, kernel_size=2, padding=1)
        self.bn2 = nn.BatchNorm2d(16)
        self.max_pool = nn.MaxPool2d(2, stride=2)
        self.dropout = nn.Dropout(0.25)
        self.fc1 = nn.Linear(32, 28)
        self.fc2 = nn.Linear(28, 4)  #  Correct output shape
        self.hybrid = Hybrid(shots=100)

    def forward(self, x):
        x = F.relu(self.bn1(self.conv1(x)))
        x = self.max_pool(x)
        x = F.relu(self.bn2(self.conv2(x)))
        x = self.max_pool(x)
        x = x.view(x.size(0), -1)
        x = F.relu(self.fc1(x))
        x = self.dropout(x)
        x = self.fc2(x)  #  No ReLU here (raw logits)

        # Fix: Ensure input to hybrid layer has exactly 2 elements
        outputs = []
        for i in range(x.size(0)):
            quantum_out = self.hybrid(x[i][:2])  #  Fixed
            outputs.append(quantum_out.unsqueeze(0))
        out = torch.cat(outputs, dim=0)
        return out

##############################################
```

```python
# Training, Validation, and Testing Setup
##############################################
model = Net()
optimizer = optim.Adam(model.parameters(), lr=1e-3)
scheduler = torch.optim.lr_scheduler.StepLR(optimizer, step_size=10, gamma=0.5)
loss_func = nn.CrossEntropyLoss()

##############################################
# Optimizer, Loss, and LR Scheduler Setup
##############################################
model = Net()
lr = 1e-4
optimizer = optim.Adam(model.parameters(), lr=lr)
loss_func = nn.CrossEntropyLoss()  # expects output shape (N, C) and target␣
 ↪shape (N,)

def adjust_learning_rate_poly(optimizer, initial_lr, iteration, max_iter):
    lr = 1e-3
    if lr < 2e-5:
        lr = 2e-5
    for param_group in optimizer.param_groups:
        param_group['lr'] = lr
    return lr

##############################################
# Training, Validation, and Test Loop with Metrics and Timing
##############################################
num_epochs = 100
train_losses = []
val_losses = []
test_losses = []
train_accuracies = []
val_accuracies = []
test_accuracies = []

start_time = time.time()

for epoch in range(num_epochs):
    # --- Training ---
    model.train()
    running_loss = 0.0
    correct_train = 0
    total_train = 0
    for data, target in train_loader:
        target = target.view(-1)
        optimizer.zero_grad()
        outputs = model(data.float())  # (batch, 2)
```

```
            loss = loss_func(outputs, target.long())
            loss.backward()
            optimizer.step()

            running_loss += loss.item() * data.size(0)
            _, predicted = torch.max(outputs, 1)
            total_train += target.size(0)
            correct_train += (predicted == target).sum().item()
        epoch_train_loss = running_loss / total_train
        epoch_train_acc = correct_train / total_train * 100.0
        train_losses.append(epoch_train_loss)
        train_accuracies.append(epoch_train_acc)

        # --- Validation ---
        model.eval()
        running_val_loss = 0.0
        correct_val = 0
        total_val = 0
        with torch.no_grad():
            for data, target in val_loader:
                target = target.view(-1)
                outputs = model(data.float())
                loss = loss_func(outputs, target.long())
                running_val_loss += loss.item() * data.size(0)
                _, predicted = torch.max(outputs, 1)
                total_val += target.size(0)
                correct_val += (predicted == target).sum().item()
        epoch_val_loss = running_val_loss / total_val
        epoch_val_acc = correct_val / total_val * 100.0
        val_losses.append(epoch_val_loss)
        val_accuracies.append(epoch_val_acc)

        # --- Test Evaluation ---
        model.eval()
        running_test_loss = 0.0
        correct_test = 0
        total_test = 0
        with torch.no_grad():
            for data, target in test_loader:
                target = target.view(-1)
                outputs = model(data.float())
                loss = loss_func(outputs, target.long())
                running_test_loss += loss.item() * data.size(0)
                _, predicted = torch.max(outputs, 1)
                total_test += target.size(0)
                correct_test += (predicted == target).sum().item()
        epoch_test_loss = running_test_loss / total_test
```

```
    epoch_test_acc = correct_test / total_test * 100.0
    test_losses.append(epoch_test_loss)
    test_accuracies.append(epoch_test_acc)

    print(f"Epoch [{epoch+1}/{num_epochs}]  "
          f"Train Loss: {epoch_train_loss:.4f}, Train Acc: {epoch_train_acc:.
↪2f}%  "
          f"Val Loss: {epoch_val_loss:.4f}, Val Acc: {epoch_val_acc:.2f}%  "
          f"Test Loss: {epoch_test_loss:.4f}, Test Acc: {epoch_test_acc:.2f}%")

end_time = time.time()
total_time = end_time - start_time

##############################################
# Print Overall Metrics
##############################################
print("\n--- Overall Metrics ---")
print(f"Train Loss: Mean = {np.mean(train_losses):.4f}, Std = {np.
↪std(train_losses):.4f}")
print(f"Train Accuracy: Mean = {np.mean(train_accuracies):.2f}%, Std = {np.
↪std(train_accuracies):.2f}%")
print(f"Validation Loss: Mean = {np.mean(val_losses):.4f}, Std = {np.
↪std(val_losses):.4f}")
print(f"Validation Accuracy: Mean = {np.mean(val_accuracies):.2f}%, Std = {np.
↪std(val_accuracies):.2f}%")
print(f"Test Loss: Mean = {np.mean(test_losses):.4f}, Std = {np.
↪std(test_losses):.4f}")
print(f"Test Accuracy: Mean = {np.mean(test_accuracies):.2f}%, Std = {np.
↪std(test_accuracies):.2f}%")
print(f"Total Computation Time: {total_time:.2f} seconds")

##############################################
# Plot Loss Curves
##############################################
epochs_range = range(1, num_epochs+1)
plt.figure(figsize=(12, 5))
plt.subplot(1, 2, 1)
plt.plot(epochs_range, train_losses, label="Train Loss", marker='o')
plt.plot(epochs_range, val_losses, label="Val Loss", marker='o')
plt.plot(epochs_range, test_losses, label="Test Loss", marker='o')
plt.xlabel("Epoch")
plt.ylabel("Loss")
plt.title("Loss vs. Epoch")
plt.legend()

##############################################
```

```
# Plot Accuracy Curves
##############################################
plt.subplot(1, 2, 2)
plt.plot(epochs_range, train_accuracies, label="Train Acc", marker='o')
plt.plot(epochs_range, val_accuracies, label="Val Acc", marker='o')
plt.plot(epochs_range, test_accuracies, label="Test Acc", marker='o')
plt.xlabel("Epoch")
plt.ylabel("Accuracy (%)")
plt.title("Accuracy vs. Epoch")
plt.legend()

plt.tight_layout()
plt.show()
```

```
/var/folders/nw/k_k0_cbj7vl_npdmryvhl53c0000gn/T/ipykernel_70899/2270739328.py:1
02: UserWarning: To copy construct from a tensor, it is recommended to use
sourceTensor.clone().detach() or
sourceTensor.clone().detach().requires_grad_(True), rather than
torch.tensor(sourceTensor).
  probabilities = torch.tensor(probabilities, dtype=torch.float32,
requires_grad=True)  #  Keeps gradients
```

```
Epoch [1/100]  Train Loss: 0.6655, Train Acc: 64.62%  Val Loss: 0.7206, Val Acc:
59.09%  Test Loss: 0.4827, Test Acc: 82.76%
Epoch [2/100]  Train Loss: 0.6652, Train Acc: 64.62%  Val Loss: 0.7200, Val Acc:
59.09%  Test Loss: 0.4840, Test Acc: 82.76%
Epoch [3/100]  Train Loss: 0.6636, Train Acc: 64.62%  Val Loss: 0.7147, Val Acc:
59.09%  Test Loss: 0.4872, Test Acc: 82.76%
Epoch [4/100]  Train Loss: 0.6585, Train Acc: 64.62%  Val Loss: 0.7073, Val Acc:
59.09%  Test Loss: 0.4849, Test Acc: 82.76%
Epoch [5/100]  Train Loss: 0.6598, Train Acc: 64.62%  Val Loss: 0.7110, Val Acc:
59.09%  Test Loss: 0.4861, Test Acc: 82.76%
Epoch [6/100]  Train Loss: 0.6460, Train Acc: 64.62%  Val Loss: 0.7203, Val Acc:
59.09%  Test Loss: 0.4889, Test Acc: 82.76%
Epoch [7/100]  Train Loss: 0.6410, Train Acc: 64.62%  Val Loss: 0.7094, Val Acc:
59.09%  Test Loss: 0.4866, Test Acc: 82.76%
Epoch [8/100]  Train Loss: 0.6258, Train Acc: 64.62%  Val Loss: 0.7126, Val Acc:
59.09%  Test Loss: 0.4936, Test Acc: 82.76%
Epoch [9/100]  Train Loss: 0.6189, Train Acc: 64.62%  Val Loss: 0.7019, Val Acc:
59.09%  Test Loss: 0.4903, Test Acc: 82.76%
Epoch [10/100]  Train Loss: 0.6044, Train Acc: 64.62%  Val Loss: 0.7019, Val
Acc: 59.09%  Test Loss: 0.4918, Test Acc: 82.76%
Epoch [11/100]  Train Loss: 0.5758, Train Acc: 65.13%  Val Loss: 0.7016, Val
Acc: 59.09%  Test Loss: 0.4867, Test Acc: 82.76%
Epoch [12/100]  Train Loss: 0.5510, Train Acc: 69.23%  Val Loss: 0.6950, Val
Acc: 59.09%  Test Loss: 0.4966, Test Acc: 82.76%
Epoch [13/100]  Train Loss: 0.5286, Train Acc: 73.33%  Val Loss: 0.6904, Val
Acc: 59.09%  Test Loss: 0.4936, Test Acc: 82.76%
```

Epoch [14/100] Train Loss: 0.5277, Train Acc: 72.82% Val Loss: 0.6900, Val Acc: 59.09% Test Loss: 0.4873, Test Acc: 82.76%
Epoch [15/100] Train Loss: 0.4736, Train Acc: 82.05% Val Loss: 0.6754, Val Acc: 59.09% Test Loss: 0.4981, Test Acc: 82.76%
Epoch [16/100] Train Loss: 0.4647, Train Acc: 84.62% Val Loss: 0.7003, Val Acc: 59.09% Test Loss: 0.4945, Test Acc: 82.76%
Epoch [17/100] Train Loss: 0.4369, Train Acc: 88.21% Val Loss: 0.7016, Val Acc: 59.09% Test Loss: 0.4852, Test Acc: 82.76%
Epoch [18/100] Train Loss: 0.4451, Train Acc: 88.21% Val Loss: 0.7104, Val Acc: 54.55% Test Loss: 0.4872, Test Acc: 82.76%
Epoch [19/100] Train Loss: 0.4410, Train Acc: 89.74% Val Loss: 0.7036, Val Acc: 59.09% Test Loss: 0.4899, Test Acc: 82.76%
Epoch [20/100] Train Loss: 0.4446, Train Acc: 91.28% Val Loss: 0.7197, Val Acc: 63.64% Test Loss: 0.4800, Test Acc: 86.21%
Epoch [21/100] Train Loss: 0.5204, Train Acc: 79.49% Val Loss: 0.7013, Val Acc: 63.64% Test Loss: 0.5133, Test Acc: 79.31%
Epoch [22/100] Train Loss: 0.6384, Train Acc: 66.15% Val Loss: 0.7212, Val Acc: 45.45% Test Loss: 0.5663, Test Acc: 72.41%
Epoch [23/100] Train Loss: 0.7956, Train Acc: 46.15% Val Loss: 0.7115, Val Acc: 54.55% Test Loss: 0.6896, Test Acc: 65.52%
Epoch [24/100] Train Loss: 0.8167, Train Acc: 44.62% Val Loss: 0.7130, Val Acc: 59.09% Test Loss: 0.7556, Test Acc: 51.72%
Epoch [25/100] Train Loss: 0.7390, Train Acc: 51.79% Val Loss: 0.7076, Val Acc: 54.55% Test Loss: 0.8028, Test Acc: 41.38%
Epoch [26/100] Train Loss: 0.6912, Train Acc: 55.90% Val Loss: 0.6976, Val Acc: 50.00% Test Loss: 0.8377, Test Acc: 37.93%
Epoch [27/100] Train Loss: 0.6854, Train Acc: 57.44% Val Loss: 0.6948, Val Acc: 54.55% Test Loss: 0.8201, Test Acc: 37.93%
Epoch [28/100] Train Loss: 0.6817, Train Acc: 60.51% Val Loss: 0.6644, Val Acc: 59.09% Test Loss: 0.8628, Test Acc: 34.48%
Epoch [29/100] Train Loss: 0.6939, Train Acc: 56.92% Val Loss: 0.6703, Val Acc: 63.64% Test Loss: 0.8315, Test Acc: 41.38%
Epoch [30/100] Train Loss: 0.8162, Train Acc: 40.00% Val Loss: 0.6586, Val Acc: 68.18% Test Loss: 0.8201, Test Acc: 41.38%
Epoch [31/100] Train Loss: 0.8101, Train Acc: 40.51% Val Loss: 0.7237, Val Acc: 54.55% Test Loss: 0.8432, Test Acc: 44.83%
Epoch [32/100] Train Loss: 0.7291, Train Acc: 51.79% Val Loss: 0.7368, Val Acc: 54.55% Test Loss: 0.8406, Test Acc: 44.83%
Epoch [33/100] Train Loss: 0.7653, Train Acc: 49.74% Val Loss: 0.7239, Val Acc: 59.09% Test Loss: 0.7638, Test Acc: 44.83%
Epoch [34/100] Train Loss: 0.7659, Train Acc: 46.67% Val Loss: 0.8154, Val Acc: 36.36% Test Loss: 0.7529, Test Acc: 51.72%
Epoch [35/100] Train Loss: 0.8160, Train Acc: 43.08% Val Loss: 0.7754, Val Acc: 40.91% Test Loss: 0.7855, Test Acc: 51.72%
Epoch [36/100] Train Loss: 0.7476, Train Acc: 49.74% Val Loss: 0.7155, Val Acc: 45.45% Test Loss: 0.8047, Test Acc: 41.38%
Epoch [37/100] Train Loss: 0.7890, Train Acc: 46.67% Val Loss: 0.6681, Val Acc: 50.00% Test Loss: 0.8026, Test Acc: 37.93%

Epoch [38/100] Train Loss: 0.7382, Train Acc: 52.31% Val Loss: 0.7786, Val Acc: 54.55% Test Loss: 0.8619, Test Acc: 44.83%
Epoch [39/100] Train Loss: 0.7265, Train Acc: 53.33% Val Loss: 0.8268, Val Acc: 45.45% Test Loss: 0.8506, Test Acc: 37.93%
Epoch [40/100] Train Loss: 0.7486, Train Acc: 51.79% Val Loss: 0.8017, Val Acc: 50.00% Test Loss: 0.8396, Test Acc: 48.28%
Epoch [41/100] Train Loss: 0.7531, Train Acc: 49.23% Val Loss: 0.8534, Val Acc: 36.36% Test Loss: 0.8639, Test Acc: 31.03%
Epoch [42/100] Train Loss: 0.7540, Train Acc: 47.18% Val Loss: 0.7591, Val Acc: 50.00% Test Loss: 0.8370, Test Acc: 44.83%
Epoch [43/100] Train Loss: 0.7778, Train Acc: 49.23% Val Loss: 0.6813, Val Acc: 63.64% Test Loss: 0.8521, Test Acc: 37.93%
Epoch [44/100] Train Loss: 0.7396, Train Acc: 52.82% Val Loss: 0.7258, Val Acc: 54.55% Test Loss: 0.7701, Test Acc: 48.28%
Epoch [45/100] Train Loss: 0.7402, Train Acc: 56.41% Val Loss: 0.6494, Val Acc: 68.18% Test Loss: 0.7898, Test Acc: 51.72%
Epoch [46/100] Train Loss: 0.7732, Train Acc: 48.21% Val Loss: 0.7975, Val Acc: 54.55% Test Loss: 0.7593, Test Acc: 48.28%
Epoch [47/100] Train Loss: 0.7584, Train Acc: 49.23% Val Loss: 0.7176, Val Acc: 59.09% Test Loss: 0.7555, Test Acc: 51.72%
Epoch [48/100] Train Loss: 0.7869, Train Acc: 47.18% Val Loss: 0.7184, Val Acc: 59.09% Test Loss: 0.8133, Test Acc: 41.38%
Epoch [49/100] Train Loss: 0.7864, Train Acc: 45.64% Val Loss: 0.7380, Val Acc: 54.55% Test Loss: 0.7655, Test Acc: 51.72%
Epoch [50/100] Train Loss: 0.8057, Train Acc: 42.56% Val Loss: 0.7777, Val Acc: 50.00% Test Loss: 0.7459, Test Acc: 48.28%
Epoch [51/100] Train Loss: 0.7371, Train Acc: 54.36% Val Loss: 0.7644, Val Acc: 50.00% Test Loss: 0.7360, Test Acc: 55.17%
Epoch [52/100] Train Loss: 0.7738, Train Acc: 47.18% Val Loss: 0.7697, Val Acc: 50.00% Test Loss: 0.7222, Test Acc: 58.62%
Epoch [53/100] Train Loss: 0.7296, Train Acc: 52.31% Val Loss: 0.8592, Val Acc: 31.82% Test Loss: 0.7488, Test Acc: 48.28%
Epoch [54/100] Train Loss: 0.7241, Train Acc: 56.92% Val Loss: 0.8065, Val Acc: 50.00% Test Loss: 0.7351, Test Acc: 48.28%
Epoch [55/100] Train Loss: 0.7681, Train Acc: 49.74% Val Loss: 0.7544, Val Acc: 50.00% Test Loss: 0.7030, Test Acc: 62.07%
Epoch [56/100] Train Loss: 0.7986, Train Acc: 45.64% Val Loss: 0.8722, Val Acc: 36.36% Test Loss: 0.8398, Test Acc: 41.38%
Epoch [57/100] Train Loss: 0.7748, Train Acc: 48.21% Val Loss: 0.7164, Val Acc: 45.45% Test Loss: 0.7334, Test Acc: 55.17%
Epoch [58/100] Train Loss: 0.7891, Train Acc: 46.15% Val Loss: 0.8431, Val Acc: 45.45% Test Loss: 0.7328, Test Acc: 55.17%
Epoch [59/100] Train Loss: 0.7846, Train Acc: 47.18% Val Loss: 0.8344, Val Acc: 40.91% Test Loss: 0.7527, Test Acc: 48.28%
Epoch [60/100] Train Loss: 0.7805, Train Acc: 46.67% Val Loss: 0.7085, Val Acc: 54.55% Test Loss: 0.7132, Test Acc: 55.17%
Epoch [61/100] Train Loss: 0.7728, Train Acc: 47.18% Val Loss: 0.7009, Val Acc: 59.09% Test Loss: 0.7102, Test Acc: 55.17%

Epoch [62/100] Train Loss: 0.7621, Train Acc: 49.23% Val Loss: 0.8623, Val Acc: 36.36% Test Loss: 0.8091, Test Acc: 44.83%
Epoch [63/100] Train Loss: 0.7631, Train Acc: 50.77% Val Loss: 0.8338, Val Acc: 36.36% Test Loss: 0.7226, Test Acc: 55.17%
Epoch [64/100] Train Loss: 0.7723, Train Acc: 49.23% Val Loss: 0.7346, Val Acc: 54.55% Test Loss: 0.7934, Test Acc: 37.93%
Epoch [65/100] Train Loss: 0.7531, Train Acc: 50.26% Val Loss: 0.7117, Val Acc: 59.09% Test Loss: 0.7044, Test Acc: 55.17%
Epoch [66/100] Train Loss: 0.7410, Train Acc: 50.77% Val Loss: 0.6627, Val Acc: 63.64% Test Loss: 0.7151, Test Acc: 55.17%
Epoch [67/100] Train Loss: 0.7486, Train Acc: 47.18% Val Loss: 0.7961, Val Acc: 45.45% Test Loss: 0.7604, Test Acc: 51.72%
Epoch [68/100] Train Loss: 0.7712, Train Acc: 50.26% Val Loss: 0.8302, Val Acc: 50.00% Test Loss: 0.8116, Test Acc: 41.38%
Epoch [69/100] Train Loss: 0.7684, Train Acc: 45.13% Val Loss: 0.9572, Val Acc: 22.73% Test Loss: 0.6967, Test Acc: 58.62%
Epoch [70/100] Train Loss: 0.7589, Train Acc: 48.72% Val Loss: 0.7212, Val Acc: 54.55% Test Loss: 0.7728, Test Acc: 44.83%
Epoch [71/100] Train Loss: 0.7205, Train Acc: 56.41% Val Loss: 0.6761, Val Acc: 59.09% Test Loss: 0.7612, Test Acc: 48.28%
Epoch [72/100] Train Loss: 0.7625, Train Acc: 50.26% Val Loss: 0.7506, Val Acc: 50.00% Test Loss: 0.7743, Test Acc: 51.72%
Epoch [73/100] Train Loss: 0.7158, Train Acc: 54.87% Val Loss: 0.8542, Val Acc: 40.91% Test Loss: 0.8180, Test Acc: 44.83%
Epoch [74/100] Train Loss: 0.7813, Train Acc: 47.69% Val Loss: 0.8266, Val Acc: 36.36% Test Loss: 0.8293, Test Acc: 41.38%
Epoch [75/100] Train Loss: 0.7825, Train Acc: 47.18% Val Loss: 0.7729, Val Acc: 50.00% Test Loss: 0.7669, Test Acc: 51.72%
Epoch [76/100] Train Loss: 0.7928, Train Acc: 46.15% Val Loss: 0.8429, Val Acc: 36.36% Test Loss: 0.8329, Test Acc: 41.38%
Epoch [77/100] Train Loss: 0.7595, Train Acc: 51.79% Val Loss: 0.7616, Val Acc: 40.91% Test Loss: 0.8421, Test Acc: 37.93%
Epoch [78/100] Train Loss: 0.7337, Train Acc: 50.77% Val Loss: 0.7284, Val Acc: 45.45% Test Loss: 0.7839, Test Acc: 44.83%
Epoch [79/100] Train Loss: 0.7536, Train Acc: 51.79% Val Loss: 0.6684, Val Acc: 63.64% Test Loss: 0.7133, Test Acc: 55.17%
Epoch [80/100] Train Loss: 0.7630, Train Acc: 50.26% Val Loss: 0.7246, Val Acc: 54.55% Test Loss: 0.8940, Test Acc: 27.59%
Epoch [81/100] Train Loss: 0.8168, Train Acc: 42.56% Val Loss: 0.8251, Val Acc: 40.91% Test Loss: 0.6452, Test Acc: 55.17%
Epoch [82/100] Train Loss: 0.7879, Train Acc: 43.08% Val Loss: 0.6478, Val Acc: 59.09% Test Loss: 0.7422, Test Acc: 41.38%
Epoch [83/100] Train Loss: 0.7275, Train Acc: 53.33% Val Loss: 0.8243, Val Acc: 40.91% Test Loss: 0.7032, Test Acc: 58.62%
Epoch [84/100] Train Loss: 0.7909, Train Acc: 46.15% Val Loss: 0.8476, Val Acc: 22.73% Test Loss: 0.7322, Test Acc: 58.62%
Epoch [85/100] Train Loss: 0.7689, Train Acc: 47.69% Val Loss: 0.7336, Val Acc: 59.09% Test Loss: 0.7322, Test Acc: 55.17%

```
Epoch [86/100]  Train Loss: 0.7874, Train Acc: 46.67%  Val Loss: 0.7656, Val
Acc: 50.00%  Test Loss: 0.7326, Test Acc: 51.72%
Epoch [87/100]  Train Loss: 0.7239, Train Acc: 54.87%  Val Loss: 0.7421, Val
Acc: 59.09%  Test Loss: 0.6047, Test Acc: 68.97%
Epoch [88/100]  Train Loss: 0.7420, Train Acc: 50.77%  Val Loss: 0.7185, Val
Acc: 59.09%  Test Loss: 0.7541, Test Acc: 51.72%
Epoch [89/100]  Train Loss: 0.7374, Train Acc: 52.31%  Val Loss: 0.7820, Val
Acc: 45.45%  Test Loss: 0.7496, Test Acc: 41.38%
Epoch [90/100]  Train Loss: 0.7867, Train Acc: 46.15%  Val Loss: 0.7404, Val
Acc: 45.45%  Test Loss: 0.7373, Test Acc: 44.83%
Epoch [91/100]  Train Loss: 0.7643, Train Acc: 49.23%  Val Loss: 0.6752, Val
Acc: 54.55%  Test Loss: 0.7998, Test Acc: 48.28%
Epoch [92/100]  Train Loss: 0.7463, Train Acc: 51.79%  Val Loss: 0.8199, Val
Acc: 36.36%  Test Loss: 0.6627, Test Acc: 68.97%
Epoch [93/100]  Train Loss: 0.7317, Train Acc: 51.79%  Val Loss: 0.6888, Val
Acc: 54.55%  Test Loss: 0.7587, Test Acc: 55.17%
Epoch [94/100]  Train Loss: 0.7600, Train Acc: 49.74%  Val Loss: 0.8383, Val
Acc: 36.36%  Test Loss: 0.8402, Test Acc: 41.38%
Epoch [95/100]  Train Loss: 0.7581, Train Acc: 51.79%  Val Loss: 0.8219, Val
Acc: 50.00%  Test Loss: 0.8003, Test Acc: 51.72%
Epoch [96/100]  Train Loss: 0.7778, Train Acc: 47.18%  Val Loss: 0.7322, Val
Acc: 59.09%  Test Loss: 0.8443, Test Acc: 41.38%
Epoch [97/100]  Train Loss: 0.7208, Train Acc: 55.90%  Val Loss: 0.8166, Val
Acc: 40.91%  Test Loss: 0.7003, Test Acc: 55.17%
Epoch [98/100]  Train Loss: 0.7262, Train Acc: 53.85%  Val Loss: 0.6522, Val
Acc: 68.18%  Test Loss: 0.7598, Test Acc: 51.72%
Epoch [99/100]  Train Loss: 0.7561, Train Acc: 50.77%  Val Loss: 0.8001, Val
Acc: 54.55%  Test Loss: 0.8461, Test Acc: 37.93%
Epoch [100/100]  Train Loss: 0.7785, Train Acc: 45.64%  Val Loss: 0.6929, Val
Acc: 59.09%  Test Loss: 0.8226, Test Acc: 44.83%

--- Overall Metrics ---
Train Loss: Mean = 0.7175, Std = 0.0927
Train Accuracy: Mean = 54.65%, Std = 11.17%
Validation Loss: Mean = 0.7454, Std = 0.0620
Validation Accuracy: Mean = 52.05%, Std = 9.46%
Test Loss: Mean = 0.7127, Std = 0.1271
Test Accuracy: Mean = 55.62%, Std = 15.89%
Total Computation Time: 21.02 seconds
```

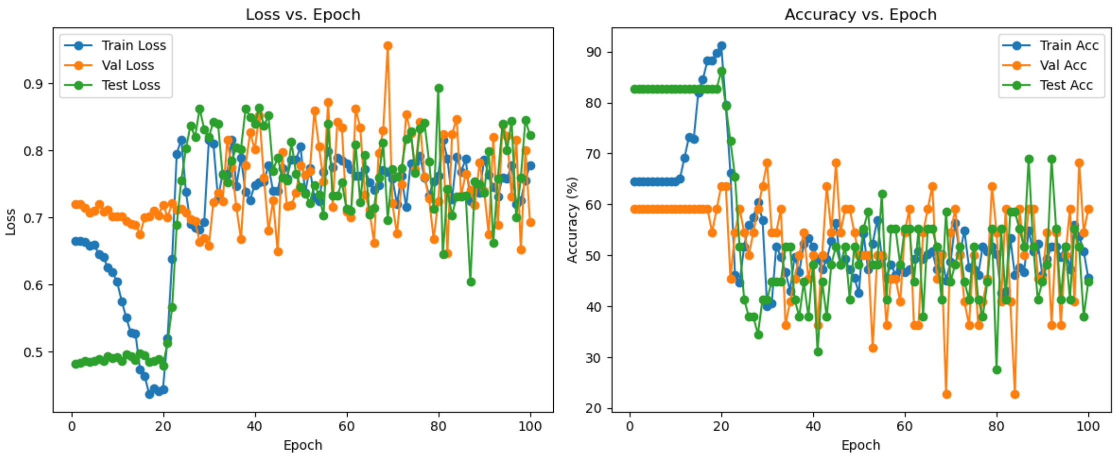

1.7 test 3. Quantvolution + No Quantfilter (original images)

[474]:
```
import numpy as np
import matplotlib.pyplot as plt
import os
import cv2
from tqdm import tqdm
from PIL import Image
from numpy import asarray
from matplotlib import image

IMG_SIZE_1 = 217
IMG_SIZE_2 = 33
DATADIR = "/Users/h4/Desktop/quantum filters_research/
↪Quantvolution_Essential_tremor/Ablation/images_split"

classes = {
    "train": ["Class1_Bad", "Class2_Good", "Class3_Right", "Class4_Left"],
    "val": ["Class1_Bad", "Class2_Good", "Class3_Right", "Class4_Left"],
    "test": ["Class1_Bad", "Class2_Good", "Class3_Right", "Class4_Left"]
}

def create_data(split):
    dataset = {class_name: [] for class_name in classes[split]}
    for i, class_name in enumerate(classes[split]):
        path = os.path.join(DATADIR, split, class_name)
        for img in tqdm(os.listdir(path), desc=f"Processing {split}␣
↪{class_name}"):
            tipo = os.path.splitext(img)[1].lower()
            if tipo in ['.jpg', '.jpeg', '.png', '.gif']:
```

```
                try:
                    img_array = asarray(Image.open(os.path.join(path, img)).
↪convert('L'))
                    new_array = cv2.resize(img_array, (IMG_SIZE_2, IMG_SIZE_1))
                    dataset[class_name].append([new_array, i])
                except Exception as e:
                    print(f"Error processing {img}: {e}")
    return dataset

train_data = create_data("train")
val_data = create_data("val")
test_data = create_data("test")

def prepare_X_y(data):
    X, y = [], []
    for class_name in data:
        for features, label in data[class_name]:
            X.append(features)
            y.append(label)
    X = np.array(X).reshape(-1, IMG_SIZE_1, IMG_SIZE_2, 1)
    y = np.array(y)
    return X, y

def unison_shuffled_copies(a, b):
    assert len(a) == len(b)
    p = np.random.permutation(len(a))
    return a[p], b[p]

X_train, y_train = prepare_X_y(train_data)
X_val, y_val = prepare_X_y(val_data)
X_test, y_test = prepare_X_y(test_data)

X_train, y_train = unison_shuffled_copies(X_train, y_train)
X_val, y_val = unison_shuffled_copies(X_val, y_val)
X_test, y_test = unison_shuffled_copies(X_test, y_test)

print("Training data shape:", X_train.shape)
print("Validation data shape:", X_val.shape)
print("Test data shape:", X_test.shape)

plt.imshow(X_train[0].squeeze(), cmap='gray')
plt.show()
```

```
Processing train Class1_Bad: 100%|       | 47/47 [00:00<00:00, 3520.60it/s]
Processing train Class2_Good: 100%|       | 47/47 [00:00<00:00, 4988.80it/s]
Processing train Class3_Right: 100%|       | 48/48 [00:00<00:00, 5567.20it/s]
Processing train Class4_Left: 100%|       | 53/53 [00:00<00:00, 5824.35it/s]
```

```
Processing val Class1_Bad: 100%|          | 5/5 [00:00<00:00, 3538.30it/s]
Processing val Class2_Good: 100%|         | 5/5 [00:00<00:00, 3996.86it/s]
Processing val Class3_Right: 100%|        | 6/6 [00:00<00:00, 3199.72it/s]
Processing val Class4_Left: 100%|         | 6/6 [00:00<00:00, 4289.39it/s]
Processing test Class1_Bad: 100%|         | 7/7 [00:00<00:00, 4476.31it/s]
Processing test Class2_Good: 100%|        | 7/7 [00:00<00:00, 4484.52it/s]
Processing test Class3_Right: 100%|       | 7/7 [00:00<00:00, 4149.25it/s]
Processing test Class4_Left: 100%|        | 8/8 [00:00<00:00, 4464.40it/s]

Training data shape: (195, 217, 33, 1)
Validation data shape: (22, 217, 33, 1)
Test data shape: (29, 217, 33, 1)
```

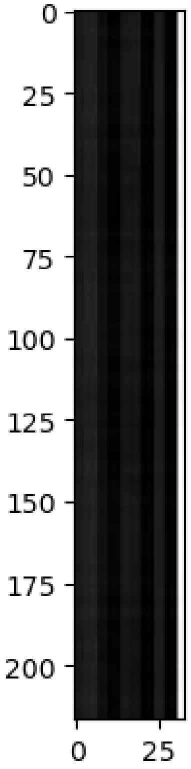

[475]:
```
train_filter = np.isin(y_train, [0, 1, 2, 3])
test_filter = np.isin(y_test, [0, 1, 2, 3])
val_filter = np.isin(y_val, [0, 1, 2, 3])

X_train, l_train = X_train[train_filter], y_train[train_filter]
X_test, l_test = X_test[test_filter], y_test[test_filter]
X_val, l_val = X_val[val_filter], y_val[val_filter]
```

[476]:
```
import torch
from torch.utils.data import Dataset, DataLoader

class CustomTensorDataset(Dataset):
    """TensorDataset with support for transforms."""
    def __init__(self, tensors, transform=None):
        assert all(tensors[0].shape[0] == tensor.shape[0] for tensor in␣
 ↪tensors), "All tensors must have the same first dimension"
        self.tensors = tensors
        self.transform = transform

    def __getitem__(self, index):
        x = self.tensors[0][index]
        if self.transform:
            x = self.transform(x)
        y = self.tensors[1][index]
        return x, y

    def __len__(self):
        return self.tensors[0].shape[0]

# Convert to PyTorch tensors
X_train_tensor = torch.tensor(X_train, dtype=torch.float32)
y_train_tensor = torch.tensor(y_train, dtype=torch.long)
X_test_tensor = torch.tensor(X_test, dtype=torch.float32)
y_test_tensor = torch.tensor(y_test, dtype=torch.long)
X_val_tensor = torch.tensor(X_val, dtype=torch.float32)
y_val_tensor = torch.tensor(y_val, dtype=torch.long)

# Create dataset and dataloader
train_dataset = CustomTensorDataset(tensors=(X_train_tensor, y_train_tensor),␣
 ↪transform=None)
train_loader = DataLoader(train_dataset, batch_size=1, shuffle=True)

test_dataset = CustomTensorDataset(tensors=(X_test_tensor, y_test_tensor),␣
 ↪transform=None)
test_loader = DataLoader(test_dataset, batch_size=1, shuffle=True)

val_dataset = CustomTensorDataset(tensors=(X_val_tensor, y_val_tensor),␣
 ↪transform=None)
val_loader = DataLoader(val_dataset, batch_size=1, shuffle=True)

# Print dataset size to check
print("Train dataset size:", len(train_dataset))
print("Test dataset size:", len(test_dataset))
print("Val dataset size:", len(val_dataset))
```

```
Train dataset size: 195
Test dataset size: 29
Val dataset size: 22
```

[467]:

```
import time
import pennylane as qml
import numpy as np
import torch
import torch.nn as nn
import torch.optim as optim
import torch.nn.functional as F
import random
from torch.autograd import Function
from torch.utils.data import DataLoader, TensorDataset
import matplotlib.pyplot as plt

# If targets are in the range 0-3, map them to binary classes:
if np.max(y_train) > 1:
    # Map original labels 0 and 1 to 0, and 2 and 3 to 1.
    y_train = np.where(y_train < 2, 0, 1)
    y_val   = np.where(y_val   < 2, 0, 1)
    y_test  = np.where(y_test  < 2, 0, 1)

# Convert to torch.Tensors.
# Also, convert images from HWC (217,33,1) to NCHW (1,217,33).
if not isinstance(X_train, torch.Tensor):
    X_train_tensor = torch.tensor(X_train, dtype=torch.float32).permute(0, 3,␣
↪1, 2)
    y_train_tensor = torch.tensor(y_train, dtype=torch.long)
    X_val_tensor   = torch.tensor(X_val, dtype=torch.float32).permute(0, 3, 1,␣
↪2)
    y_val_tensor   = torch.tensor(y_val, dtype=torch.long)
    X_test_tensor  = torch.tensor(X_test, dtype=torch.float32).permute(0, 3, 1,␣
↪2)
    y_test_tensor  = torch.tensor(y_test, dtype=torch.long)
else:
    # If already tensors, assume proper format or permute as needed.
    X_train_tensor = X_train.permute(0, 3, 1, 2)
    X_val_tensor   = X_val.permute(0, 3, 1, 2)
    X_test_tensor  = X_test.permute(0, 3, 1, 2)
    y_train_tensor = y_train
    y_val_tensor   = y_val
    y_test_tensor  = y_test

# Create TensorDatasets and DataLoaders.
train_dataset = TensorDataset(X_train_tensor, y_train_tensor)
val_dataset   = TensorDataset(X_val_tensor, y_val_tensor)
```

```python
test_dataset  = TensorDataset(X_test_tensor, y_test_tensor)

train_loader = DataLoader(train_dataset, batch_size=4, shuffle=True)
val_loader   = DataLoader(val_dataset, batch_size=4, shuffle=False)
test_loader  = DataLoader(test_dataset, batch_size=4, shuffle=False)

##############################################
# Define a simple PennyLane Quantum Circuit
##############################################
class QuantumCircuit:
    def __init__(self, n_qubits, shots):
        self.n_qubits = n_qubits
        self.shots = shots
        self.dev = qml.device("default.qubit", wires=n_qubits, shots=shots)

    def run(self, inputs):
        @qml.qnode(self.dev, interface="torch")
        def circuit(inputs):
            qml.RX(inputs[0], wires=0)
            qml.RX(inputs[1], wires=1)
            qml.CNOT(wires=[0, 1])
            return qml.probs(wires=[0])
        inputs = torch.tensor(inputs, dtype=torch.float32)
        result = circuit(inputs)
        return result.detach().numpy()  # returns shape (2,)

##############################################
# Define the Hybrid Quantum-Classical Layer
##############################################
class HybridFunction(Function):
    @staticmethod
    def forward(ctx, input, quantum_circuit, shift):
        ctx.shift = shift
        ctx.quantum_circuit = quantum_circuit

        input_array = input.detach().cpu().numpy().flatten()
        if input_array.shape[0] != 2:
            raise ValueError(f"Expected input to have 2 elements, but got shape␣
 ↪{input_array.shape} with values {input_array}")

        probabilities = ctx.quantum_circuit.run(input_array)
        probabilities_shift_plus = ctx.quantum_circuit.run(
            np.array([input_array[0] + np.pi/2 + random.uniform(1e-6, 1e-5),
                      input_array[1] + np.pi/2])
        )
        probabilities_shift_minus = ctx.quantum_circuit.run(
            np.array([input_array[0] - np.pi/2 + random.uniform(1e-6, 1e-5),
```

```
                    input_array[1] - np.pi/2])
        )

        probabilities = torch.tensor(probabilities, dtype=torch.float32)
        probabilities_shift_plus = torch.tensor(probabilities_shift_plus,␣
↪dtype=torch.float32)
        probabilities_shift_minus = torch.tensor(probabilities_shift_minus,␣
↪dtype=torch.float32)

        ctx.save_for_backward(input, probabilities, probabilities_shift_plus,␣
↪probabilities_shift_minus)
        return probabilities  # shape (2,)

    @staticmethod
    def backward(ctx, grad_output):
        input, probabilities, probabilities_shift_plus,␣
↪probabilities_shift_minus = ctx.saved_tensors
        gradient = ((probabilities_shift_plus - probabilities_shift_minus) / 2).
↪clone().detach().requires_grad_(True)
        grad = gradient.float() * grad_output.float().clone().detach().
↪requires_grad_(True)
        return grad, None, None

class Hybrid(nn.Module):
    def __init__(self, shots, shift):
        super(Hybrid, self).__init__()
        self.quantum_circuit = QuantumCircuit(n_qubits=2, shots=shots)
        self.shift = shift

    def forward(self, input):
        return HybridFunction.apply(input, self.quantum_circuit, self.shift)

##############################################
# Define the Main Network for input shape (1,217,33)
##############################################
class Net(nn.Module):
    def __init__(self):
        super(Net, self).__init__()
        # For input shape: (N, 1, 217, 33)
        self.conv1 = nn.Conv2d(in_channels=1, out_channels=64, kernel_size=3,␣
↪padding=1)   # → (N,64,217,33)
        self.bn1 = nn.BatchNorm2d(64)
        self.pool1 = nn.MaxPool2d(2,2)   # → (N,64,108,16)
        self.conv2 = nn.Conv2d(64, 32, kernel_size=3, padding=1)   # →␣
↪(N,32,108,16)
        self.bn2 = nn.BatchNorm2d(32)
```

```
        self.pool2 = nn.MaxPool2d(2,2)  # → (N,32,54,8)
        # Use adaptive pooling to get a fixed feature map size (e.g., 4x4).
        self.adapt = nn.AdaptiveAvgPool2d((4,4))  # → (N,32,4,4) => flattened =
 ↪32*4*4 = 512.
        self.fc1 = nn.Linear(512, 40)
        self.fc2 = nn.Linear(40, 32)
        self.fc3 = nn.Linear(32, 16)
        self.drop = nn.Dropout(0.25)
        self.fc4 = nn.Linear(16, 8)
        self.fc5 = nn.Linear(8, 2)
        self.hybrid = Hybrid(shots=100, shift=np.pi/2)

    def forward(self, x):
        # x: (N, 1, 217, 33)
        x = F.relu(self.conv1(x))   # (N,64,217,33)
        x = self.pool1(x)           # (N,64,108,16)
        x = F.relu(self.bn1(x))
        x = F.relu(self.conv2(x))   # (N,32,108,16)
        x = self.pool2(x)           # (N,32,54,8)
        x = F.relu(self.bn2(x))
        x = self.adapt(x)           # (N,32,4,4)
        x = x.view(x.size(0), -1)   # Flatten → (N,512)
        x = F.relu(self.fc1(x))     # → (N,40)
        x = self.fc2(x)             # → (N,32)
        x = F.relu(self.fc3(x))     # → (N,16)
        x = self.drop(x)
        x = self.fc4(x)             # → (N,8)
        x = F.relu(self.fc5(x))     # → (N,2)

        # Process each sample with the hybrid quantum layer.
        outputs = []
        for i in range(x.size(0)):
            quantum_out = self.hybrid(x[i])  # each x[i] is expected to be (2,)
            outputs.append(quantum_out.unsqueeze(0))
        out = torch.cat(outputs, dim=0)       # final output: (N,2)
        return out

##############################################
# Optimizer, Loss, and LR Scheduler Setup
##############################################
model = Net()
lr = 1e-4
optimizer = optim.Adam(model.parameters(), lr=lr)
loss_func = nn.CrossEntropyLoss()

def adjust_learning_rate_poly(optimizer, initial_lr, iteration, max_iter):
    lr = 1e-4
```

```python
    if lr < 2e-5:
        lr = 2e-5
    for param_group in optimizer.param_groups:
        param_group['lr'] = lr
    return lr

##############################################
# Training, Validation, and Test Loop with Metrics and Timing
##############################################
num_epochs = 100
train_losses = []
val_losses = []
test_losses = []
train_accuracies = []
val_accuracies = []
test_accuracies = []

start_time = time.time()

for epoch in range(num_epochs):
    # --- Training ---
    model.train()
    running_loss = 0.0
    correct_train = 0
    total_train = 0
    for data, target in train_loader:
        target = target.view(-1)
        optimizer.zero_grad()
        outputs = model(data.float())
        loss = loss_func(outputs, target.long())
        loss.backward()
        optimizer.step()

        running_loss += loss.item() * data.size(0)
        _, predicted = torch.max(outputs, 1)
        total_train += target.size(0)
        correct_train += (predicted == target).sum().item()
    epoch_train_loss = running_loss / total_train
    epoch_train_acc = correct_train / total_train * 100.0
    train_losses.append(epoch_train_loss)
    train_accuracies.append(epoch_train_acc)

    # --- Validation ---
    model.eval()
    running_val_loss = 0.0
    correct_val = 0
    total_val = 0
```

```
    with torch.no_grad():
        for data, target in val_loader:
            target = target.view(-1)
            outputs = model(data.float())
            loss = loss_func(outputs, target.long())
            running_val_loss += loss.item() * data.size(0)
            _, predicted = torch.max(outputs, 1)
            total_val += target.size(0)
            correct_val += (predicted == target).sum().item()
    epoch_val_loss = running_val_loss / total_val
    epoch_val_acc = correct_val / total_val * 100.0
    val_losses.append(epoch_val_loss)
    val_accuracies.append(epoch_val_acc)

    # --- Test Evaluation ---
    model.eval()
    running_test_loss = 0.0
    correct_test = 0
    total_test = 0
    with torch.no_grad():
        for data, target in test_loader:
            target = target.view(-1)
            outputs = model(data.float())
            loss = loss_func(outputs, target.long())
            running_test_loss += loss.item() * data.size(0)
            _, predicted = torch.max(outputs, 1)
            total_test += target.size(0)
            correct_test += (predicted == target).sum().item()
    epoch_test_loss = running_test_loss / total_test
    epoch_test_acc = correct_test / total_test * 100.0
    test_losses.append(epoch_test_loss)
    test_accuracies.append(epoch_test_acc)

    print(f"Epoch [{epoch+1}/{num_epochs}]  "
          f"Train Loss: {epoch_train_loss:.4f}, Train Acc: {epoch_train_acc:.
↪2f}%  "
          f"Val Loss: {epoch_val_loss:.4f}, Val Acc: {epoch_val_acc:.2f}%  "
          f"Test Loss: {epoch_test_loss:.4f}, Test Acc: {epoch_test_acc:.2f}%")

end_time = time.time()
total_time = end_time - start_time

##############################################
# Print Overall Metrics
##############################################
print("\n--- Overall Metrics ---")
```

```python
print(f"Train Loss: Mean = {np.mean(train_losses):.4f}, Std = {np.
    std(train_losses):.4f}")
print(f"Train Accuracy: Mean = {np.mean(train_accuracies):.2f}%, Std = {np.
    std(train_accuracies):.2f}%")
print(f"Validation Loss: Mean = {np.mean(val_losses):.4f}, Std = {np.
    std(val_losses):.4f}")
print(f"Validation Accuracy: Mean = {np.mean(val_accuracies):.2f}%, Std = {np.
    std(val_accuracies):.2f}%")
print(f"Test Loss: Mean = {np.mean(test_losses):.4f}, Std = {np.
    std(test_losses):.4f}")
print(f"Test Accuracy: Mean = {np.mean(test_accuracies):.2f}%, Std = {np.
    std(test_accuracies):.2f}%")
print(f"Total Computation Time: {total_time:.2f} seconds")

##############################################
# Plot Loss Curves
##############################################
epochs_range = range(1, num_epochs+1)
plt.figure(figsize=(12, 5))
plt.subplot(1, 2, 1)
plt.plot(epochs_range, train_losses, label="Train Loss", marker='o')
plt.plot(epochs_range, val_losses, label="Val Loss", marker='o')
plt.plot(epochs_range, test_losses, label="Test Loss", marker='o')
plt.xlabel("Epoch")
plt.ylabel("Loss")
plt.title("Loss vs. Epoch")
plt.legend()

##############################################
# Plot Accuracy Curves
##############################################
plt.subplot(1, 2, 2)
plt.plot(epochs_range, train_accuracies, label="Train Acc", marker='o')
plt.plot(epochs_range, val_accuracies, label="Val Acc", marker='o')
plt.plot(epochs_range, test_accuracies, label="Test Acc", marker='o')
plt.xlabel("Epoch")
plt.ylabel("Accuracy (%)")
plt.title("Accuracy vs. Epoch")
plt.legend()

plt.tight_layout()
plt.show()
```

```
Epoch [1/100]  Train Loss: 0.8074, Train Acc: 50.59%  Val Loss: 0.8427, Val Acc:
47.06%  Test Loss: 0.7915, Test Acc: 52.17%
Epoch [2/100]  Train Loss: 0.8074, Train Acc: 50.59%  Val Loss: 0.8427, Val Acc:
47.06%  Test Loss: 0.7915, Test Acc: 52.17%
```

```
Epoch [3/100]  Train Loss: 0.8074, Train Acc: 50.59%  Val Loss: 0.8427, Val Acc:
47.06%  Test Loss: 0.7915, Test Acc: 52.17%
Epoch [4/100]  Train Loss: 0.8074, Train Acc: 50.59%  Val Loss: 0.8427, Val Acc:
47.06%  Test Loss: 0.7915, Test Acc: 52.17%
Epoch [5/100]  Train Loss: 0.8074, Train Acc: 50.59%  Val Loss: 0.8427, Val Acc:
47.06%  Test Loss: 0.7915, Test Acc: 52.17%
Epoch [6/100]  Train Loss: 0.8074, Train Acc: 50.59%  Val Loss: 0.8427, Val Acc:
47.06%  Test Loss: 0.7915, Test Acc: 52.17%
Epoch [7/100]  Train Loss: 0.8072, Train Acc: 50.59%  Val Loss: 0.8427, Val Acc:
47.06%  Test Loss: 0.7909, Test Acc: 52.17%
Epoch [8/100]  Train Loss: 0.8055, Train Acc: 50.59%  Val Loss: 0.8353, Val Acc:
47.06%  Test Loss: 0.7848, Test Acc: 52.17%
Epoch [9/100]  Train Loss: 0.7723, Train Acc: 52.35%  Val Loss: 0.7477, Val Acc:
52.94%  Test Loss: 0.6937, Test Acc: 65.22%
Epoch [10/100]  Train Loss: 0.6326, Train Acc: 65.29%  Val Loss: 0.6056, Val
Acc: 64.71%  Test Loss: 0.6027, Test Acc: 65.22%
Epoch [11/100]  Train Loss: 0.4462, Train Acc: 88.24%  Val Loss: 0.3501, Val
Acc: 100.00%  Test Loss: 0.3272, Test Acc: 100.00%
Epoch [12/100]  Train Loss: 0.3948, Train Acc: 97.06%  Val Loss: 0.3392, Val
Acc: 100.00%  Test Loss: 0.3354, Test Acc: 100.00%
Epoch [13/100]  Train Loss: 0.4095, Train Acc: 94.12%  Val Loss: 0.3360, Val
Acc: 100.00%  Test Loss: 0.3353, Test Acc: 100.00%
Epoch [14/100]  Train Loss: 0.3795, Train Acc: 95.88%  Val Loss: 0.3403, Val
Acc: 100.00%  Test Loss: 0.3260, Test Acc: 100.00%
Epoch [15/100]  Train Loss: 0.4078, Train Acc: 92.94%  Val Loss: 0.3469, Val
Acc: 100.00%  Test Loss: 0.3452, Test Acc: 100.00%
Epoch [16/100]  Train Loss: 0.3924, Train Acc: 93.53%  Val Loss: 0.3453, Val
Acc: 100.00%  Test Loss: 0.3365, Test Acc: 100.00%
Epoch [17/100]  Train Loss: 0.3765, Train Acc: 97.06%  Val Loss: 0.3305, Val
Acc: 100.00%  Test Loss: 0.3302, Test Acc: 100.00%
Epoch [18/100]  Train Loss: 0.4070, Train Acc: 92.94%  Val Loss: 0.3481, Val
Acc: 100.00%  Test Loss: 0.3422, Test Acc: 100.00%
Epoch [19/100]  Train Loss: 0.3741, Train Acc: 94.71%  Val Loss: 0.3350, Val
Acc: 100.00%  Test Loss: 0.3305, Test Acc: 100.00%
Epoch [20/100]  Train Loss: 0.3808, Train Acc: 94.71%  Val Loss: 0.3290, Val
Acc: 100.00%  Test Loss: 0.3234, Test Acc: 100.00%
Epoch [21/100]  Train Loss: 0.3685, Train Acc: 98.24%  Val Loss: 0.3286, Val
Acc: 100.00%  Test Loss: 0.3194, Test Acc: 100.00%
Epoch [22/100]  Train Loss: 0.3684, Train Acc: 95.29%  Val Loss: 0.3272, Val
Acc: 100.00%  Test Loss: 0.3345, Test Acc: 100.00%
Epoch [23/100]  Train Loss: 0.3571, Train Acc: 98.24%  Val Loss: 0.3291, Val
Acc: 100.00%  Test Loss: 0.3283, Test Acc: 100.00%
Epoch [24/100]  Train Loss: 0.3597, Train Acc: 96.47%  Val Loss: 0.3315, Val
Acc: 100.00%  Test Loss: 0.3308, Test Acc: 100.00%
Epoch [25/100]  Train Loss: 0.3617, Train Acc: 95.88%  Val Loss: 0.3303, Val
Acc: 100.00%  Test Loss: 0.3188, Test Acc: 100.00%
Epoch [26/100]  Train Loss: 0.3565, Train Acc: 98.24%  Val Loss: 0.3296, Val
Acc: 100.00%  Test Loss: 0.3292, Test Acc: 100.00%
```

```
Epoch [27/100]  Train Loss: 0.3551, Train Acc: 98.82%  Val Loss: 0.3281, Val
Acc: 100.00%  Test Loss: 0.3255, Test Acc: 100.00%
Epoch [28/100]  Train Loss: 0.3709, Train Acc: 96.47%  Val Loss: 0.3255, Val
Acc: 100.00%  Test Loss: 0.3222, Test Acc: 100.00%
Epoch [29/100]  Train Loss: 0.3450, Train Acc: 100.00%  Val Loss: 0.3290, Val
Acc: 100.00%  Test Loss: 0.3222, Test Acc: 100.00%
Epoch [30/100]  Train Loss: 0.3505, Train Acc: 98.24%  Val Loss: 0.3249, Val
Acc: 100.00%  Test Loss: 0.3196, Test Acc: 100.00%
Epoch [31/100]  Train Loss: 0.3405, Train Acc: 100.00%  Val Loss: 0.3343, Val
Acc: 100.00%  Test Loss: 0.3206, Test Acc: 100.00%
Epoch [32/100]  Train Loss: 0.3623, Train Acc: 97.65%  Val Loss: 0.3255, Val
Acc: 100.00%  Test Loss: 0.3206, Test Acc: 100.00%
Epoch [33/100]  Train Loss: 0.3588, Train Acc: 98.82%  Val Loss: 0.3266, Val
Acc: 100.00%  Test Loss: 0.3266, Test Acc: 100.00%
Epoch [34/100]  Train Loss: 0.3435, Train Acc: 99.41%  Val Loss: 0.3301, Val
Acc: 100.00%  Test Loss: 0.3183, Test Acc: 100.00%
Epoch [35/100]  Train Loss: 0.3488, Train Acc: 98.82%  Val Loss: 0.3216, Val
Acc: 100.00%  Test Loss: 0.3166, Test Acc: 100.00%
Epoch [36/100]  Train Loss: 0.3556, Train Acc: 97.06%  Val Loss: 0.3275, Val
Acc: 100.00%  Test Loss: 0.3228, Test Acc: 100.00%
Epoch [37/100]  Train Loss: 0.3612, Train Acc: 96.47%  Val Loss: 0.3322, Val
Acc: 100.00%  Test Loss: 0.3290, Test Acc: 100.00%
Epoch [38/100]  Train Loss: 0.3634, Train Acc: 98.24%  Val Loss: 0.3234, Val
Acc: 100.00%  Test Loss: 0.3215, Test Acc: 100.00%
Epoch [39/100]  Train Loss: 0.3482, Train Acc: 98.24%  Val Loss: 0.3258, Val
Acc: 100.00%  Test Loss: 0.3208, Test Acc: 100.00%
Epoch [40/100]  Train Loss: 0.3551, Train Acc: 99.41%  Val Loss: 0.3216, Val
Acc: 100.00%  Test Loss: 0.3238, Test Acc: 100.00%
Epoch [41/100]  Train Loss: 0.3580, Train Acc: 97.06%  Val Loss: 0.3210, Val
Acc: 100.00%  Test Loss: 0.3193, Test Acc: 100.00%
Epoch [42/100]  Train Loss: 0.3575, Train Acc: 98.24%  Val Loss: 0.3242, Val
Acc: 100.00%  Test Loss: 0.3222, Test Acc: 100.00%
Epoch [43/100]  Train Loss: 0.3506, Train Acc: 97.06%  Val Loss: 0.3221, Val
Acc: 100.00%  Test Loss: 0.3198, Test Acc: 100.00%
Epoch [44/100]  Train Loss: 0.3587, Train Acc: 97.06%  Val Loss: 0.3274, Val
Acc: 100.00%  Test Loss: 0.3196, Test Acc: 100.00%
Epoch [45/100]  Train Loss: 0.3522, Train Acc: 97.06%  Val Loss: 0.3236, Val
Acc: 100.00%  Test Loss: 0.3207, Test Acc: 100.00%
Epoch [46/100]  Train Loss: 0.3455, Train Acc: 99.41%  Val Loss: 0.3223, Val
Acc: 100.00%  Test Loss: 0.3147, Test Acc: 100.00%
Epoch [47/100]  Train Loss: 0.3404, Train Acc: 99.41%  Val Loss: 0.3294, Val
Acc: 100.00%  Test Loss: 0.3281, Test Acc: 100.00%
Epoch [48/100]  Train Loss: 0.3471, Train Acc: 98.82%  Val Loss: 0.3234, Val
Acc: 100.00%  Test Loss: 0.3220, Test Acc: 100.00%
Epoch [49/100]  Train Loss: 0.3422, Train Acc: 98.82%  Val Loss: 0.3246, Val
Acc: 100.00%  Test Loss: 0.3222, Test Acc: 100.00%
Epoch [50/100]  Train Loss: 0.3332, Train Acc: 100.00%  Val Loss: 0.3268, Val
Acc: 100.00%  Test Loss: 0.3246, Test Acc: 100.00%
```

Epoch [51/100] Train Loss: 0.3527, Train Acc: 98.24% Val Loss: 0.3217, Val Acc: 100.00% Test Loss: 0.3181, Test Acc: 100.00%
Epoch [52/100] Train Loss: 0.3422, Train Acc: 99.41% Val Loss: 0.3218, Val Acc: 100.00% Test Loss: 0.3168, Test Acc: 100.00%
Epoch [53/100] Train Loss: 0.3395, Train Acc: 99.41% Val Loss: 0.3278, Val Acc: 100.00% Test Loss: 0.3183, Test Acc: 100.00%
Epoch [54/100] Train Loss: 0.3459, Train Acc: 98.82% Val Loss: 0.3213, Val Acc: 100.00% Test Loss: 0.3156, Test Acc: 100.00%
Epoch [55/100] Train Loss: 0.3493, Train Acc: 100.00% Val Loss: 0.3251, Val Acc: 100.00% Test Loss: 0.3216, Test Acc: 100.00%
Epoch [56/100] Train Loss: 0.3439, Train Acc: 98.82% Val Loss: 0.3239, Val Acc: 100.00% Test Loss: 0.3188, Test Acc: 100.00%
Epoch [57/100] Train Loss: 0.3446, Train Acc: 99.41% Val Loss: 0.3264, Val Acc: 100.00% Test Loss: 0.3234, Test Acc: 100.00%
Epoch [58/100] Train Loss: 0.3423, Train Acc: 98.82% Val Loss: 0.3232, Val Acc: 100.00% Test Loss: 0.3232, Test Acc: 100.00%
Epoch [59/100] Train Loss: 0.3394, Train Acc: 98.82% Val Loss: 0.3240, Val Acc: 100.00% Test Loss: 0.3212, Test Acc: 100.00%
Epoch [60/100] Train Loss: 0.3367, Train Acc: 99.41% Val Loss: 0.3211, Val Acc: 100.00% Test Loss: 0.3225, Test Acc: 100.00%
Epoch [61/100] Train Loss: 0.3505, Train Acc: 97.65% Val Loss: 0.3249, Val Acc: 100.00% Test Loss: 0.3217, Test Acc: 100.00%
Epoch [62/100] Train Loss: 0.3460, Train Acc: 98.82% Val Loss: 0.3281, Val Acc: 100.00% Test Loss: 0.3263, Test Acc: 100.00%
Epoch [63/100] Train Loss: 0.3491, Train Acc: 98.82% Val Loss: 0.3208, Val Acc: 100.00% Test Loss: 0.3198, Test Acc: 100.00%
Epoch [64/100] Train Loss: 0.3401, Train Acc: 99.41% Val Loss: 0.3198, Val Acc: 100.00% Test Loss: 0.3176, Test Acc: 100.00%
Epoch [65/100] Train Loss: 0.3360, Train Acc: 100.00% Val Loss: 0.3211, Val Acc: 100.00% Test Loss: 0.3169, Test Acc: 100.00%
Epoch [66/100] Train Loss: 0.3391, Train Acc: 100.00% Val Loss: 0.3264, Val Acc: 100.00% Test Loss: 0.3180, Test Acc: 100.00%
Epoch [67/100] Train Loss: 0.3427, Train Acc: 98.82% Val Loss: 0.3188, Val Acc: 100.00% Test Loss: 0.3166, Test Acc: 100.00%
Epoch [68/100] Train Loss: 0.3532, Train Acc: 98.24% Val Loss: 0.3185, Val Acc: 100.00% Test Loss: 0.3171, Test Acc: 100.00%
Epoch [69/100] Train Loss: 0.3426, Train Acc: 99.41% Val Loss: 0.3185, Val Acc: 100.00% Test Loss: 0.3208, Test Acc: 100.00%
Epoch [70/100] Train Loss: 0.3401, Train Acc: 99.41% Val Loss: 0.3231, Val Acc: 100.00% Test Loss: 0.3213, Test Acc: 100.00%
Epoch [71/100] Train Loss: 0.3424, Train Acc: 100.00% Val Loss: 0.3156, Val Acc: 100.00% Test Loss: 0.3156, Test Acc: 100.00%
Epoch [72/100] Train Loss: 0.3459, Train Acc: 99.41% Val Loss: 0.3241, Val Acc: 100.00% Test Loss: 0.3164, Test Acc: 100.00%
Epoch [73/100] Train Loss: 0.3569, Train Acc: 97.06% Val Loss: 0.3187, Val Acc: 100.00% Test Loss: 0.3171, Test Acc: 100.00%
Epoch [74/100] Train Loss: 0.3401, Train Acc: 100.00% Val Loss: 0.3221, Val Acc: 100.00% Test Loss: 0.3205, Test Acc: 100.00%

```
Epoch [75/100]  Train Loss: 0.3424, Train Acc: 99.41%  Val Loss: 0.3204, Val
Acc: 100.00%  Test Loss: 0.3198, Test Acc: 100.00%
Epoch [76/100]  Train Loss: 0.3398, Train Acc: 100.00%  Val Loss: 0.3215, Val
Acc: 100.00%  Test Loss: 0.3253, Test Acc: 100.00%
Epoch [77/100]  Train Loss: 0.3406, Train Acc: 99.41%  Val Loss: 0.3201, Val
Acc: 100.00%  Test Loss: 0.3164, Test Acc: 100.00%
Epoch [78/100]  Train Loss: 0.3411, Train Acc: 100.00%  Val Loss: 0.3174, Val
Acc: 100.00%  Test Loss: 0.3185, Test Acc: 100.00%
Epoch [79/100]  Train Loss: 0.3411, Train Acc: 100.00%  Val Loss: 0.3178, Val
Acc: 100.00%  Test Loss: 0.3159, Test Acc: 100.00%
Epoch [80/100]  Train Loss: 0.3486, Train Acc: 98.82%  Val Loss: 0.3169, Val
Acc: 100.00%  Test Loss: 0.3147, Test Acc: 100.00%
Epoch [81/100]  Train Loss: 0.3452, Train Acc: 98.82%  Val Loss: 0.3194, Val
Acc: 100.00%  Test Loss: 0.3203, Test Acc: 100.00%
Epoch [82/100]  Train Loss: 0.3491, Train Acc: 98.82%  Val Loss: 0.3181, Val
Acc: 100.00%  Test Loss: 0.3168, Test Acc: 100.00%
Epoch [83/100]  Train Loss: 0.3394, Train Acc: 98.82%  Val Loss: 0.3239, Val
Acc: 100.00%  Test Loss: 0.3220, Test Acc: 100.00%
Epoch [84/100]  Train Loss: 0.3420, Train Acc: 98.82%  Val Loss: 0.3188, Val
Acc: 100.00%  Test Loss: 0.3171, Test Acc: 100.00%
Epoch [85/100]  Train Loss: 0.3362, Train Acc: 100.00%  Val Loss: 0.3228, Val
Acc: 100.00%  Test Loss: 0.3240, Test Acc: 100.00%
Epoch [86/100]  Train Loss: 0.3463, Train Acc: 98.24%  Val Loss: 0.3210, Val
Acc: 100.00%  Test Loss: 0.3183, Test Acc: 100.00%
Epoch [87/100]  Train Loss: 0.3488, Train Acc: 97.65%  Val Loss: 0.3195, Val
Acc: 100.00%  Test Loss: 0.3159, Test Acc: 100.00%
Epoch [88/100]  Train Loss: 0.3411, Train Acc: 100.00%  Val Loss: 0.3179, Val
Acc: 100.00%  Test Loss: 0.3186, Test Acc: 100.00%
Epoch [89/100]  Train Loss: 0.3446, Train Acc: 99.41%  Val Loss: 0.3201, Val
Acc: 100.00%  Test Loss: 0.3176, Test Acc: 100.00%
Epoch [90/100]  Train Loss: 0.3443, Train Acc: 98.82%  Val Loss: 0.3183, Val
Acc: 100.00%  Test Loss: 0.3173, Test Acc: 100.00%
Epoch [91/100]  Train Loss: 0.3417, Train Acc: 98.24%  Val Loss: 0.3197, Val
Acc: 100.00%  Test Loss: 0.3166, Test Acc: 100.00%
Epoch [92/100]  Train Loss: 0.3448, Train Acc: 98.82%  Val Loss: 0.3198, Val
Acc: 100.00%  Test Loss: 0.3178, Test Acc: 100.00%
Epoch [93/100]  Train Loss: 0.3369, Train Acc: 100.00%  Val Loss: 0.3171, Val
Acc: 100.00%  Test Loss: 0.3190, Test Acc: 100.00%
Epoch [94/100]  Train Loss: 0.3406, Train Acc: 99.41%  Val Loss: 0.3165, Val
Acc: 100.00%  Test Loss: 0.3149, Test Acc: 100.00%
Epoch [95/100]  Train Loss: 0.3481, Train Acc: 98.82%  Val Loss: 0.3152, Val
Acc: 100.00%  Test Loss: 0.3142, Test Acc: 100.00%
Epoch [96/100]  Train Loss: 0.3432, Train Acc: 100.00%  Val Loss: 0.3174, Val
Acc: 100.00%  Test Loss: 0.3149, Test Acc: 100.00%
Epoch [97/100]  Train Loss: 0.3346, Train Acc: 100.00%  Val Loss: 0.3181, Val
Acc: 100.00%  Test Loss: 0.3152, Test Acc: 100.00%
Epoch [98/100]  Train Loss: 0.3425, Train Acc: 99.41%  Val Loss: 0.3168, Val
Acc: 100.00%  Test Loss: 0.3149, Test Acc: 100.00%
```

```
Epoch [99/100]  Train Loss: 0.3413, Train Acc: 99.41%  Val Loss: 0.3152, Val
Acc: 100.00%  Test Loss: 0.3135, Test Acc: 100.00%
Epoch [100/100]  Train Loss: 0.3327, Train Acc: 100.00%  Val Loss: 0.3207, Val
Acc: 100.00%  Test Loss: 0.3161, Test Acc: 100.00%

--- Overall Metrics ---
Train Loss: Mean = 0.3960, Std = 0.1323
Train Accuracy: Mean = 93.66%, Std = 14.00%
Validation Loss: Mean = 0.3731, Std = 0.1472
Validation Accuracy: Mean = 94.94%, Std = 15.27%
Test Loss: Mean = 0.3656, Std = 0.1337
Test Accuracy: Mean = 95.48%, Std = 13.67%
Total Computation Time: 88.26 seconds
```

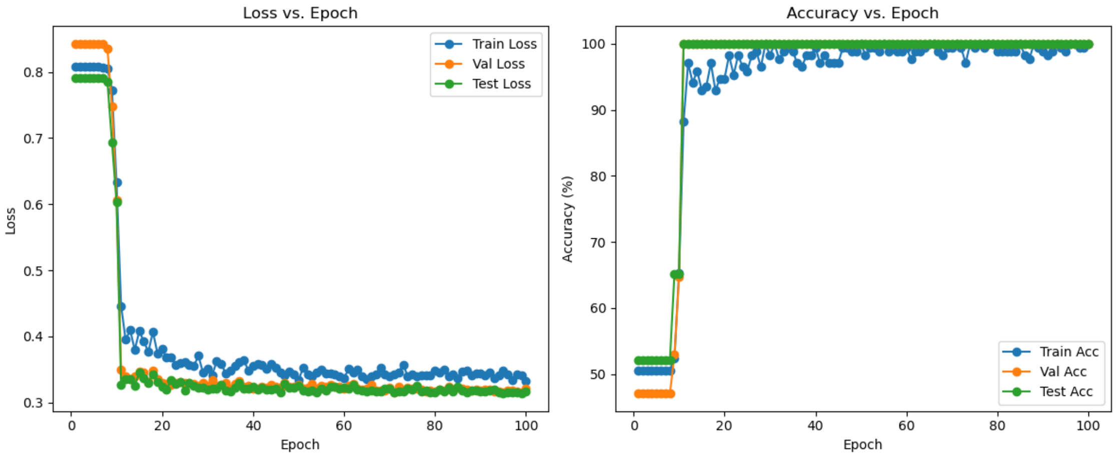

1.8 test 4. No Quantvolution (classic decision layer) + Quantfilter 2x2

[499]:
```
n_epochs = 100   # Number of optimization epochs
n_layers = 1    # Number of random layers
n_train  = 195    # Size of the train dataset
n_test   = 29     # Size of the test dataset
n_val    = 22     # Size of the validation dataset

SAVE_PATH = "/Users/h4/Desktop/quantum filters_research/
↪Quantvolution_Essential_tremor/Ablation/Quantvolution_Essential_tremor/
↪test_1/" # Data saving folder
PREPROCESS = True           # If False, skip quantum processing and load data␣
↪from SAVE_PATH
np.random.seed(0)           # Seed for NumPy random number generator
tf.random.set_seed(0)       # Seed for TensorFlow random number generator
```

[500]:
```
# Load pre-processed images
q_train_images = np.load(SAVE_PATH + "q_train_images_test_1.npy")
q_val_images   = np.load(SAVE_PATH + "q_val_images_test_1.npy")
q_test_images  = np.load(SAVE_PATH + "q_test_images_test_1.npy")
```

[501]:
```
X_train = torch.tensor(q_train_images)
y_train = torch.tensor(train_labels)
X_val = torch.tensor(q_val_images)
y_val = torch.tensor(val_labels)
X_test = torch.tensor(q_test_images)
y_test = torch.tensor(test_labels)
```

[502]:
```
import torch
from torch.utils.data import Dataset, DataLoader

class CustomTensorDataset(Dataset):
    """TensorDataset with support for transforms."""
    def __init__(self, tensors, transform=None):
        assert all(tensors[0].shape[0] == tensor.shape[0] for tensor in␣
 ↪tensors), "All tensors must have the same first dimension"
        self.tensors = tensors
        self.transform = transform

    def __getitem__(self, index):
        x = self.tensors[0][index]
        if self.transform:
            x = self.transform(x)
        y = self.tensors[1][index]
        return x, y

    def __len__(self):
        return self.tensors[0].shape[0]

# Convert to PyTorch tensors
X_train_tensor = torch.tensor(X_train, dtype=torch.float32)
y_train_tensor = torch.tensor(y_train, dtype=torch.long)
X_test_tensor = torch.tensor(X_test, dtype=torch.float32)
y_test_tensor = torch.tensor(y_test, dtype=torch.long)
X_val_tensor = torch.tensor(X_val, dtype=torch.float32)
y_val_tensor = torch.tensor(y_val, dtype=torch.long)

# Create dataset and dataloader
train_dataset = CustomTensorDataset(tensors=(X_train_tensor, y_train_tensor),␣
 ↪transform=None)
train_loader = DataLoader(train_dataset, batch_size=1, shuffle=True)
```

```
test_dataset = CustomTensorDataset(tensors=(X_test_tensor, y_test_tensor),␣
↪transform=None)
test_loader = DataLoader(test_dataset, batch_size=1, shuffle=True)

val_dataset = CustomTensorDataset(tensors=(X_val_tensor, y_val_tensor),␣
↪transform=None)
val_loader = DataLoader(val_dataset, batch_size=1, shuffle=True)

# Print dataset size to check
print("Train dataset size:", len(train_dataset))
print("Test dataset size:", len(test_dataset))
print("Val dataset size:", len(val_dataset))
```

```
Train dataset size: 195
Test dataset size: 29
Val dataset size: 22
```

```
/var/folders/nw/k_k0_cbj7vl_npdmryvhl53c0000gn/T/ipykernel_70899/522783547.py:22
: UserWarning: To copy construct from a tensor, it is recommended to use
sourceTensor.clone().detach() or
sourceTensor.clone().detach().requires_grad_(True), rather than
torch.tensor(sourceTensor).
  X_train_tensor = torch.tensor(X_train, dtype=torch.float32)
/var/folders/nw/k_k0_cbj7vl_npdmryvhl53c0000gn/T/ipykernel_70899/522783547.py:23
: UserWarning: To copy construct from a tensor, it is recommended to use
sourceTensor.clone().detach() or
sourceTensor.clone().detach().requires_grad_(True), rather than
torch.tensor(sourceTensor).
  y_train_tensor = torch.tensor(y_train, dtype=torch.long)
/var/folders/nw/k_k0_cbj7vl_npdmryvhl53c0000gn/T/ipykernel_70899/522783547.py:24
: UserWarning: To copy construct from a tensor, it is recommended to use
sourceTensor.clone().detach() or
sourceTensor.clone().detach().requires_grad_(True), rather than
torch.tensor(sourceTensor).
  X_test_tensor = torch.tensor(X_test, dtype=torch.float32)
/var/folders/nw/k_k0_cbj7vl_npdmryvhl53c0000gn/T/ipykernel_70899/522783547.py:25
: UserWarning: To copy construct from a tensor, it is recommended to use
sourceTensor.clone().detach() or
sourceTensor.clone().detach().requires_grad_(True), rather than
torch.tensor(sourceTensor).
  y_test_tensor = torch.tensor(y_test, dtype=torch.long)
/var/folders/nw/k_k0_cbj7vl_npdmryvhl53c0000gn/T/ipykernel_70899/522783547.py:26
: UserWarning: To copy construct from a tensor, it is recommended to use
sourceTensor.clone().detach() or
sourceTensor.clone().detach().requires_grad_(True), rather than
torch.tensor(sourceTensor).
  X_val_tensor = torch.tensor(X_val, dtype=torch.float32)
/var/folders/nw/k_k0_cbj7vl_npdmryvhl53c0000gn/T/ipykernel_70899/522783547.py:27
```

```
: UserWarning: To copy construct from a tensor, it is recommended to use
sourceTensor.clone().detach() or
sourceTensor.clone().detach().requires_grad_(True), rather than
torch.tensor(sourceTensor).
  y_val_tensor = torch.tensor(y_val, dtype=torch.long)
```

[507]:
```
import time
import numpy as np
import torch
import torch.nn as nn
import torch.optim as optim
import torch.nn.functional as F
from torch.utils.data import DataLoader, TensorDataset
import matplotlib.pyplot as plt

##############################################
# Load and Prepare Your Real Dataset
##############################################
print("X_train shape:", np.shape(X_train))
print("y_train shape:", np.shape(y_train))
print("X_val shape:", np.shape(X_val))
print("y_val shape:", np.shape(y_val))
print("X_test shape:", np.shape(X_test))
print("y_test shape:", np.shape(y_test))

# Convert the arrays/tensors to torch.Tensors (if not already).
if not isinstance(X_train, torch.Tensor):
    X_train_tensor = torch.tensor(X_train, dtype=torch.float32)
    y_train_tensor = torch.tensor(y_train, dtype=torch.long)
    X_val_tensor   = torch.tensor(X_val, dtype=torch.float32)
    y_val_tensor   = torch.tensor(y_val, dtype=torch.long)
    X_test_tensor  = torch.tensor(X_test, dtype=torch.float32)
    y_test_tensor  = torch.tensor(y_test, dtype=torch.long)
else:
    X_train_tensor = X_train
    y_train_tensor = y_train
    X_val_tensor   = X_val
    y_val_tensor   = y_val
    X_test_tensor  = X_test
    y_test_tensor  = y_test

# Create TensorDatasets and DataLoaders.
train_dataset = TensorDataset(X_train_tensor, y_train_tensor)
val_dataset   = TensorDataset(X_val_tensor, y_val_tensor)
test_dataset  = TensorDataset(X_test_tensor, y_test_tensor)

train_loader = DataLoader(train_dataset, batch_size=4, shuffle=True)
```

```python
val_loader   = DataLoader(val_dataset, batch_size=4, shuffle=False)
test_loader  = DataLoader(test_dataset, batch_size=4, shuffle=False)

##############################################
# Define the Fully Classical Network
##############################################
class ClassicalNet(nn.Module):
    def __init__(self):
        super(ClassicalNet, self).__init__()
        # Convolutional layers.
        self.conv1 = nn.Conv2d(108, 64, kernel_size=2, padding=(1,1))
        self.conv1_bn1 = nn.BatchNorm2d(64)
        self.conv2 = nn.Conv2d(64, 32, kernel_size=2, padding=(1,1))
        self.conv2_bn2 = nn.BatchNorm2d(32)
        self.max_pool2d = nn.MaxPool2d(2, stride=2)
        self.dropout2d = nn.Dropout2d()

        # Feature extraction
        self.fc1 = nn.Linear(128, 40)
        self.fc2 = nn.Linear(40, 32)
        self.fc3 = nn.Linear(32, 16)
        self.dropout_fc = nn.Dropout(0.25)
        self.fc4 = nn.Linear(16, 8)
        self.dropout_fc2 = nn.Dropout(0.25)
        self.fc5 = nn.Linear(8, 4)  # Output 4 classes instead of 2

    def forward(self, x):
        x = F.relu(self.conv1(x))
        x = self.max_pool2d(x)
        x = F.relu(self.conv1_bn1(x))
        x = self.dropout2d(x)

        x = F.relu(self.conv2(x))
        x = self.max_pool2d(x)
        x = F.relu(self.conv2_bn2(x))
        x = self.dropout2d(x)

        x = x.view(x.size(0), -1)  # Flatten
        x = F.relu(self.fc1(x))
        x = self.fc2(x)
        x = F.relu(self.fc3(x))
        x = self.dropout_fc(x)
        x = self.fc4(x)
        x = self.fc5(x)  # Output layer with 4 neurons
        return x

##############################################
```

```
# Optimizer, Loss, and Training Setup
##############################################
model = ClassicalNet()
optimizer = optim.Adam(model.parameters(), lr=1e-4)
loss_func = nn.CrossEntropyLoss()  # Multi-class classification

num_epochs = 100
train_losses, val_losses, test_losses = [], [], []
train_accuracies, val_accuracies, test_accuracies = [], [], []

start_time = time.time()

for epoch in range(num_epochs):
    # --- Training ---
    model.train()
    running_loss, correct_train, total_train = 0.0, 0, 0
    for data, target in train_loader:
        target = torch.argmax(target, dim=1)  # Convert one-hot to class index
        optimizer.zero_grad()
        outputs = model(data.float())
        loss = loss_func(outputs, target.long())
        loss.backward()
        optimizer.step()

        running_loss += loss.item() * data.size(0)
        _, predicted = torch.max(outputs, 1)
        total_train += target.size(0)
        correct_train += (predicted == target).sum().item()

    train_losses.append(running_loss / total_train)
    train_accuracies.append(correct_train / total_train * 100.0)

    # --- Validation ---
    model.eval()
    running_val_loss, correct_val, total_val = 0.0, 0, 0
    with torch.no_grad():
        for data, target in val_loader:
            target = torch.argmax(target, dim=1)  # Convert one-hot to class␣
↪index
            outputs = model(data.float())
            loss = loss_func(outputs, target.long())
            running_val_loss += loss.item() * data.size(0)
            _, predicted = torch.max(outputs, 1)
            total_val += target.size(0)
            correct_val += (predicted == target).sum().item()

    val_losses.append(running_val_loss / total_val)
```

```
    val_accuracies.append(correct_val / total_val * 100.0)

    # --- Test Evaluation ---
    running_test_loss, correct_test, total_test = 0.0, 0, 0
    with torch.no_grad():
        for data, target in test_loader:
            target = torch.argmax(target, dim=1)  # Convert one-hot to class␣
↪index
            outputs = model(data.float())
            loss = loss_func(outputs, target.long())
            running_test_loss += loss.item() * data.size(0)
            _, predicted = torch.max(outputs, 1)
            total_test += target.size(0)
            correct_test += (predicted == target).sum().item()

    test_losses.append(running_test_loss / total_test)
    test_accuracies.append(correct_test / total_test * 100.0)

    print(f"Epoch [{epoch+1}/{num_epochs}] "
          f"Train Loss: {train_losses[-1]:.4f}, Train Acc:␣
↪{train_accuracies[-1]:.2f}%  "
          f"Val Loss: {val_losses[-1]:.4f}, Val Acc: {val_accuracies[-1]:.2f}% ␣
↪"
          f"Test Loss: {test_losses[-1]:.4f}, Test Acc: {test_accuracies[-1]:.
↪2f}%")

end_time = time.time()
total_time = end_time - start_time

##############################################
# Print Overall Metrics
##############################################
print("\n--- Overall Metrics ---")
print(f"Train Loss: Mean = {np.mean(train_losses):.4f}, Std = {np.
↪std(train_losses):.4f}")
print(f"Train Accuracy: Mean = {np.mean(train_accuracies):.2f}%, Std = {np.
↪std(train_accuracies):.2f}%")
print(f"Validation Loss: Mean = {np.mean(val_losses):.4f}, Std = {np.
↪std(val_losses):.4f}")
print(f"Validation Accuracy: Mean = {np.mean(val_accuracies):.2f}%, Std = {np.
↪std(val_accuracies):.2f}%")
print(f"Test Loss: Mean = {np.mean(test_losses):.4f}, Std = {np.
↪std(test_losses):.4f}")
print(f"Test Accuracy: Mean = {np.mean(test_accuracies):.2f}%, Std = {np.
↪std(test_accuracies):.2f}%")
print(f"Total Computation Time: {total_time:.2f} seconds")
```

```python
################################################
# Plot Loss Curves
################################################
epochs_range = range(1, num_epochs+1)
plt.figure(figsize=(12, 5))
plt.subplot(1, 2, 1)
plt.plot(epochs_range, train_losses, label="Train Loss", marker='o')
plt.plot(epochs_range, val_losses, label="Val Loss", marker='o')
plt.plot(epochs_range, test_losses, label="Test Loss", marker='o')
plt.xlabel("Epoch")
plt.ylabel("Loss")
plt.title("Loss vs. Epoch")
plt.legend()

################################################
# Plot Accuracy Curves
################################################
plt.subplot(1, 2, 2)
plt.plot(epochs_range, train_accuracies, label="Train Acc", marker='o')
plt.plot(epochs_range, val_accuracies, label="Val Acc", marker='o')
plt.plot(epochs_range, test_accuracies, label="Test Acc", marker='o')
plt.xlabel("Epoch")
plt.ylabel("Accuracy (%)")
plt.title("Accuracy vs. Epoch")
plt.legend()

plt.tight_layout()
plt.show()
```

```
X_train shape: torch.Size([195, 108, 16, 4])
y_train shape: torch.Size([195, 4])
X_val shape: torch.Size([22, 108, 16, 4])
y_val shape: torch.Size([22, 4])
X_test shape: torch.Size([29, 108, 16, 4])
y_test shape: torch.Size([29, 4])
Epoch [1/100] Train Loss: 1.3980, Train Acc: 24.10%  Val Loss: 1.3976, Val Acc:
22.73%  Test Loss: 1.3967, Test Acc: 24.14%
Epoch [2/100] Train Loss: 1.3987, Train Acc: 24.10%  Val Loss: 1.3943, Val Acc:
22.73%  Test Loss: 1.3963, Test Acc: 24.14%
Epoch [3/100] Train Loss: 1.3980, Train Acc: 24.10%  Val Loss: 1.3934, Val Acc:
22.73%  Test Loss: 1.3955, Test Acc: 24.14%
Epoch [4/100] Train Loss: 1.3949, Train Acc: 24.62%  Val Loss: 1.3930, Val Acc:
22.73%  Test Loss: 1.3947, Test Acc: 24.14%
Epoch [5/100] Train Loss: 1.3946, Train Acc: 25.13%  Val Loss: 1.3922, Val Acc:
22.73%  Test Loss: 1.3935, Test Acc: 24.14%
Epoch [6/100] Train Loss: 1.3951, Train Acc: 23.59%  Val Loss: 1.3925, Val Acc:
```

22.73% Test Loss: 1.3930, Test Acc: 24.14%
Epoch [7/100] Train Loss: 1.3946, Train Acc: 24.10% Val Loss: 1.3920, Val Acc: 22.73% Test Loss: 1.3928, Test Acc: 24.14%
Epoch [8/100] Train Loss: 1.3911, Train Acc: 25.64% Val Loss: 1.3916, Val Acc: 22.73% Test Loss: 1.3917, Test Acc: 24.14%
Epoch [9/100] Train Loss: 1.3933, Train Acc: 25.13% Val Loss: 1.3916, Val Acc: 22.73% Test Loss: 1.3917, Test Acc: 24.14%
Epoch [10/100] Train Loss: 1.3910, Train Acc: 26.67% Val Loss: 1.3901, Val Acc: 22.73% Test Loss: 1.3918, Test Acc: 24.14%
Epoch [11/100] Train Loss: 1.3911, Train Acc: 26.15% Val Loss: 1.3891, Val Acc: 22.73% Test Loss: 1.3920, Test Acc: 24.14%
Epoch [12/100] Train Loss: 1.3898, Train Acc: 27.69% Val Loss: 1.3879, Val Acc: 22.73% Test Loss: 1.3924, Test Acc: 24.14%
Epoch [13/100] Train Loss: 1.3888, Train Acc: 28.72% Val Loss: 1.3858, Val Acc: 22.73% Test Loss: 1.3928, Test Acc: 24.14%
Epoch [14/100] Train Loss: 1.3912, Train Acc: 28.72% Val Loss: 1.3859, Val Acc: 22.73% Test Loss: 1.3941, Test Acc: 20.69%
Epoch [15/100] Train Loss: 1.3874, Train Acc: 28.21% Val Loss: 1.3862, Val Acc: 22.73% Test Loss: 1.3946, Test Acc: 17.24%
Epoch [16/100] Train Loss: 1.3912, Train Acc: 26.67% Val Loss: 1.3875, Val Acc: 22.73% Test Loss: 1.3922, Test Acc: 17.24%
Epoch [17/100] Train Loss: 1.3881, Train Acc: 27.18% Val Loss: 1.3868, Val Acc: 22.73% Test Loss: 1.3912, Test Acc: 20.69%
Epoch [18/100] Train Loss: 1.3857, Train Acc: 29.74% Val Loss: 1.3853, Val Acc: 31.82% Test Loss: 1.3909, Test Acc: 17.24%
Epoch [19/100] Train Loss: 1.3846, Train Acc: 30.26% Val Loss: 1.3862, Val Acc: 27.27% Test Loss: 1.3901, Test Acc: 20.69%
Epoch [20/100] Train Loss: 1.3883, Train Acc: 24.10% Val Loss: 1.3846, Val Acc: 31.82% Test Loss: 1.3910, Test Acc: 20.69%
Epoch [21/100] Train Loss: 1.3808, Train Acc: 31.79% Val Loss: 1.3838, Val Acc: 31.82% Test Loss: 1.3919, Test Acc: 24.14%
Epoch [22/100] Train Loss: 1.3833, Train Acc: 28.21% Val Loss: 1.3830, Val Acc: 31.82% Test Loss: 1.3934, Test Acc: 20.69%
Epoch [23/100] Train Loss: 1.3757, Train Acc: 33.85% Val Loss: 1.3785, Val Acc: 36.36% Test Loss: 1.3948, Test Acc: 20.69%
Epoch [24/100] Train Loss: 1.3783, Train Acc: 30.77% Val Loss: 1.3772, Val Acc: 31.82% Test Loss: 1.3906, Test Acc: 24.14%
Epoch [25/100] Train Loss: 1.3832, Train Acc: 34.36% Val Loss: 1.3781, Val Acc: 31.82% Test Loss: 1.3910, Test Acc: 20.69%
Epoch [26/100] Train Loss: 1.3796, Train Acc: 32.31% Val Loss: 1.3800, Val Acc: 31.82% Test Loss: 1.3938, Test Acc: 20.69%
Epoch [27/100] Train Loss: 1.3789, Train Acc: 30.77% Val Loss: 1.3793, Val Acc: 31.82% Test Loss: 1.3937, Test Acc: 24.14%
Epoch [28/100] Train Loss: 1.3691, Train Acc: 30.77% Val Loss: 1.3787, Val Acc: 40.91% Test Loss: 1.3941, Test Acc: 24.14%
Epoch [29/100] Train Loss: 1.3719, Train Acc: 31.79% Val Loss: 1.3751, Val Acc: 36.36% Test Loss: 1.3968, Test Acc: 24.14%
Epoch [30/100] Train Loss: 1.3782, Train Acc: 31.28% Val Loss: 1.3779, Val Acc:

31.82% Test Loss: 1.3920, Test Acc: 27.59%
Epoch [31/100] Train Loss: 1.3757, Train Acc: 28.21% Val Loss: 1.3735, Val Acc: 31.82% Test Loss: 1.3957, Test Acc: 24.14%
Epoch [32/100] Train Loss: 1.3763, Train Acc: 29.74% Val Loss: 1.3721, Val Acc: 31.82% Test Loss: 1.3934, Test Acc: 27.59%
Epoch [33/100] Train Loss: 1.3543, Train Acc: 34.87% Val Loss: 1.3767, Val Acc: 31.82% Test Loss: 1.3919, Test Acc: 24.14%
Epoch [34/100] Train Loss: 1.3546, Train Acc: 33.85% Val Loss: 1.3697, Val Acc: 36.36% Test Loss: 1.3984, Test Acc: 24.14%
Epoch [35/100] Train Loss: 1.3488, Train Acc: 34.36% Val Loss: 1.3732, Val Acc: 36.36% Test Loss: 1.3953, Test Acc: 24.14%
Epoch [36/100] Train Loss: 1.3635, Train Acc: 29.23% Val Loss: 1.3728, Val Acc: 40.91% Test Loss: 1.3960, Test Acc: 27.59%
Epoch [37/100] Train Loss: 1.3553, Train Acc: 31.79% Val Loss: 1.3747, Val Acc: 40.91% Test Loss: 1.3959, Test Acc: 24.14%
Epoch [38/100] Train Loss: 1.3469, Train Acc: 35.90% Val Loss: 1.3741, Val Acc: 40.91% Test Loss: 1.3989, Test Acc: 27.59%
Epoch [39/100] Train Loss: 1.3484, Train Acc: 33.33% Val Loss: 1.3726, Val Acc: 40.91% Test Loss: 1.3991, Test Acc: 24.14%
Epoch [40/100] Train Loss: 1.3462, Train Acc: 33.85% Val Loss: 1.3759, Val Acc: 31.82% Test Loss: 1.3981, Test Acc: 27.59%
Epoch [41/100] Train Loss: 1.3414, Train Acc: 37.44% Val Loss: 1.3812, Val Acc: 31.82% Test Loss: 1.3959, Test Acc: 20.69%
Epoch [42/100] Train Loss: 1.3355, Train Acc: 37.44% Val Loss: 1.3751, Val Acc: 36.36% Test Loss: 1.3921, Test Acc: 20.69%
Epoch [43/100] Train Loss: 1.3422, Train Acc: 33.33% Val Loss: 1.3777, Val Acc: 31.82% Test Loss: 1.3906, Test Acc: 20.69%
Epoch [44/100] Train Loss: 1.3254, Train Acc: 36.41% Val Loss: 1.3789, Val Acc: 31.82% Test Loss: 1.3869, Test Acc: 20.69%
Epoch [45/100] Train Loss: 1.3300, Train Acc: 33.33% Val Loss: 1.3815, Val Acc: 31.82% Test Loss: 1.3813, Test Acc: 27.59%
Epoch [46/100] Train Loss: 1.3138, Train Acc: 38.97% Val Loss: 1.3770, Val Acc: 31.82% Test Loss: 1.3947, Test Acc: 27.59%
Epoch [47/100] Train Loss: 1.3158, Train Acc: 38.46% Val Loss: 1.3733, Val Acc: 31.82% Test Loss: 1.4065, Test Acc: 20.69%
Epoch [48/100] Train Loss: 1.2976, Train Acc: 37.95% Val Loss: 1.3773, Val Acc: 36.36% Test Loss: 1.4106, Test Acc: 24.14%
Epoch [49/100] Train Loss: 1.3274, Train Acc: 36.92% Val Loss: 1.3833, Val Acc: 36.36% Test Loss: 1.4078, Test Acc: 20.69%
Epoch [50/100] Train Loss: 1.3101, Train Acc: 38.46% Val Loss: 1.3785, Val Acc: 36.36% Test Loss: 1.4044, Test Acc: 31.03%
Epoch [51/100] Train Loss: 1.2904, Train Acc: 39.49% Val Loss: 1.3842, Val Acc: 31.82% Test Loss: 1.4072, Test Acc: 27.59%
Epoch [52/100] Train Loss: 1.2604, Train Acc: 41.54% Val Loss: 1.3759, Val Acc: 36.36% Test Loss: 1.3993, Test Acc: 24.14%
Epoch [53/100] Train Loss: 1.2635, Train Acc: 37.44% Val Loss: 1.3851, Val Acc: 36.36% Test Loss: 1.4086, Test Acc: 27.59%
Epoch [54/100] Train Loss: 1.2611, Train Acc: 44.10% Val Loss: 1.4166, Val Acc:

31.82% Test Loss: 1.3998, Test Acc: 24.14%
Epoch [55/100] Train Loss: 1.2977, Train Acc: 37.44% Val Loss: 1.4149, Val Acc: 31.82% Test Loss: 1.3933, Test Acc: 20.69%
Epoch [56/100] Train Loss: 1.2847, Train Acc: 35.90% Val Loss: 1.3991, Val Acc: 31.82% Test Loss: 1.3936, Test Acc: 27.59%
Epoch [57/100] Train Loss: 1.2816, Train Acc: 38.97% Val Loss: 1.4029, Val Acc: 31.82% Test Loss: 1.3999, Test Acc: 27.59%
Epoch [58/100] Train Loss: 1.2713, Train Acc: 35.90% Val Loss: 1.4009, Val Acc: 31.82% Test Loss: 1.4049, Test Acc: 20.69%
Epoch [59/100] Train Loss: 1.2726, Train Acc: 36.92% Val Loss: 1.4026, Val Acc: 27.27% Test Loss: 1.4042, Test Acc: 24.14%
Epoch [60/100] Train Loss: 1.2425, Train Acc: 41.03% Val Loss: 1.3895, Val Acc: 31.82% Test Loss: 1.4027, Test Acc: 27.59%
Epoch [61/100] Train Loss: 1.2521, Train Acc: 38.97% Val Loss: 1.4105, Val Acc: 31.82% Test Loss: 1.3987, Test Acc: 24.14%
Epoch [62/100] Train Loss: 1.2476, Train Acc: 41.54% Val Loss: 1.4364, Val Acc: 31.82% Test Loss: 1.4028, Test Acc: 24.14%
Epoch [63/100] Train Loss: 1.2391, Train Acc: 39.49% Val Loss: 1.4477, Val Acc: 27.27% Test Loss: 1.4057, Test Acc: 24.14%
Epoch [64/100] Train Loss: 1.2363, Train Acc: 40.51% Val Loss: 1.4331, Val Acc: 27.27% Test Loss: 1.3967, Test Acc: 17.24%
Epoch [65/100] Train Loss: 1.1815, Train Acc: 42.56% Val Loss: 1.4144, Val Acc: 27.27% Test Loss: 1.3932, Test Acc: 20.69%
Epoch [66/100] Train Loss: 1.2271, Train Acc: 41.03% Val Loss: 1.4024, Val Acc: 31.82% Test Loss: 1.3957, Test Acc: 20.69%
Epoch [67/100] Train Loss: 1.2126, Train Acc: 44.62% Val Loss: 1.4437, Val Acc: 27.27% Test Loss: 1.4161, Test Acc: 17.24%
Epoch [68/100] Train Loss: 1.2321, Train Acc: 38.97% Val Loss: 1.4079, Val Acc: 27.27% Test Loss: 1.4140, Test Acc: 17.24%
Epoch [69/100] Train Loss: 1.2084, Train Acc: 43.59% Val Loss: 1.4192, Val Acc: 27.27% Test Loss: 1.4214, Test Acc: 20.69%
Epoch [70/100] Train Loss: 1.2036, Train Acc: 38.97% Val Loss: 1.4539, Val Acc: 27.27% Test Loss: 1.4182, Test Acc: 20.69%
Epoch [71/100] Train Loss: 1.2102, Train Acc: 38.46% Val Loss: 1.4371, Val Acc: 27.27% Test Loss: 1.4393, Test Acc: 17.24%
Epoch [72/100] Train Loss: 1.1748, Train Acc: 43.59% Val Loss: 1.4646, Val Acc: 31.82% Test Loss: 1.4546, Test Acc: 17.24%
Epoch [73/100] Train Loss: 1.2339, Train Acc: 41.54% Val Loss: 1.4489, Val Acc: 27.27% Test Loss: 1.4244, Test Acc: 24.14%
Epoch [74/100] Train Loss: 1.2043, Train Acc: 42.56% Val Loss: 1.4647, Val Acc: 27.27% Test Loss: 1.4276, Test Acc: 20.69%
Epoch [75/100] Train Loss: 1.1835, Train Acc: 43.59% Val Loss: 1.4769, Val Acc: 27.27% Test Loss: 1.4379, Test Acc: 20.69%
Epoch [76/100] Train Loss: 1.1379, Train Acc: 44.62% Val Loss: 1.4948, Val Acc: 31.82% Test Loss: 1.4413, Test Acc: 24.14%
Epoch [77/100] Train Loss: 1.1579, Train Acc: 42.56% Val Loss: 1.4823, Val Acc: 31.82% Test Loss: 1.4513, Test Acc: 13.79%
Epoch [78/100] Train Loss: 1.1318, Train Acc: 46.15% Val Loss: 1.4662, Val Acc:

31.82% Test Loss: 1.4581, Test Acc: 13.79%
Epoch [79/100] Train Loss: 1.1500, Train Acc: 46.15% Val Loss: 1.4218, Val Acc: 27.27% Test Loss: 1.4548, Test Acc: 20.69%
Epoch [80/100] Train Loss: 1.1793, Train Acc: 41.03% Val Loss: 1.4250, Val Acc: 31.82% Test Loss: 1.4410, Test Acc: 24.14%
Epoch [81/100] Train Loss: 1.1455, Train Acc: 44.62% Val Loss: 1.4215, Val Acc: 36.36% Test Loss: 1.4066, Test Acc: 24.14%
Epoch [82/100] Train Loss: 1.1322, Train Acc: 44.62% Val Loss: 1.4023, Val Acc: 22.73% Test Loss: 1.4298, Test Acc: 24.14%
Epoch [83/100] Train Loss: 1.0964, Train Acc: 46.67% Val Loss: 1.3931, Val Acc: 36.36% Test Loss: 1.4382, Test Acc: 24.14%
Epoch [84/100] Train Loss: 1.1714, Train Acc: 41.54% Val Loss: 1.3967, Val Acc: 31.82% Test Loss: 1.4268, Test Acc: 24.14%
Epoch [85/100] Train Loss: 1.0901, Train Acc: 49.23% Val Loss: 1.4019, Val Acc: 31.82% Test Loss: 1.4520, Test Acc: 20.69%
Epoch [86/100] Train Loss: 1.0947, Train Acc: 44.62% Val Loss: 1.3894, Val Acc: 31.82% Test Loss: 1.4509, Test Acc: 17.24%
Epoch [87/100] Train Loss: 1.1255, Train Acc: 47.18% Val Loss: 1.3565, Val Acc: 36.36% Test Loss: 1.4319, Test Acc: 20.69%
Epoch [88/100] Train Loss: 1.0936, Train Acc: 45.13% Val Loss: 1.4100, Val Acc: 31.82% Test Loss: 1.4144, Test Acc: 24.14%
Epoch [89/100] Train Loss: 1.1240, Train Acc: 47.69% Val Loss: 1.4177, Val Acc: 36.36% Test Loss: 1.3953, Test Acc: 20.69%
Epoch [90/100] Train Loss: 1.0426, Train Acc: 49.23% Val Loss: 1.3850, Val Acc: 36.36% Test Loss: 1.4284, Test Acc: 13.79%
Epoch [91/100] Train Loss: 1.1550, Train Acc: 45.13% Val Loss: 1.3626, Val Acc: 40.91% Test Loss: 1.4061, Test Acc: 17.24%
Epoch [92/100] Train Loss: 1.1016, Train Acc: 46.67% Val Loss: 1.3970, Val Acc: 40.91% Test Loss: 1.3894, Test Acc: 17.24%
Epoch [93/100] Train Loss: 1.1090, Train Acc: 49.23% Val Loss: 1.3967, Val Acc: 31.82% Test Loss: 1.4492, Test Acc: 13.79%
Epoch [94/100] Train Loss: 1.1007, Train Acc: 50.26% Val Loss: 1.4055, Val Acc: 36.36% Test Loss: 1.4097, Test Acc: 17.24%
Epoch [95/100] Train Loss: 1.0987, Train Acc: 46.67% Val Loss: 1.4123, Val Acc: 31.82% Test Loss: 1.4653, Test Acc: 13.79%
Epoch [96/100] Train Loss: 1.0483, Train Acc: 46.67% Val Loss: 1.4254, Val Acc: 22.73% Test Loss: 1.4276, Test Acc: 17.24%
Epoch [97/100] Train Loss: 1.1140, Train Acc: 41.03% Val Loss: 1.3974, Val Acc: 31.82% Test Loss: 1.4315, Test Acc: 17.24%
Epoch [98/100] Train Loss: 1.0691, Train Acc: 49.23% Val Loss: 1.4108, Val Acc: 31.82% Test Loss: 1.4412, Test Acc: 17.24%
Epoch [99/100] Train Loss: 1.0719, Train Acc: 48.72% Val Loss: 1.3792, Val Acc: 31.82% Test Loss: 1.4305, Test Acc: 17.24%
Epoch [100/100] Train Loss: 1.0516, Train Acc: 47.18% Val Loss: 1.3596, Val Acc: 27.27% Test Loss: 1.4724, Test Acc: 17.24%

--- Overall Metrics ---
Train Loss: Mean = 1.2719, Std = 0.1111

```
Train Accuracy: Mean = 36.95%, Std = 7.54%
Validation Loss: Mean = 1.3985, Std = 0.0278
Validation Accuracy: Mean = 30.77%, Std = 5.18%
Test Loss: Mean = 1.4083, Std = 0.0210
Test Accuracy: Mean = 22.03%, Std = 3.74%
Total Computation Time: 7.59 seconds
```

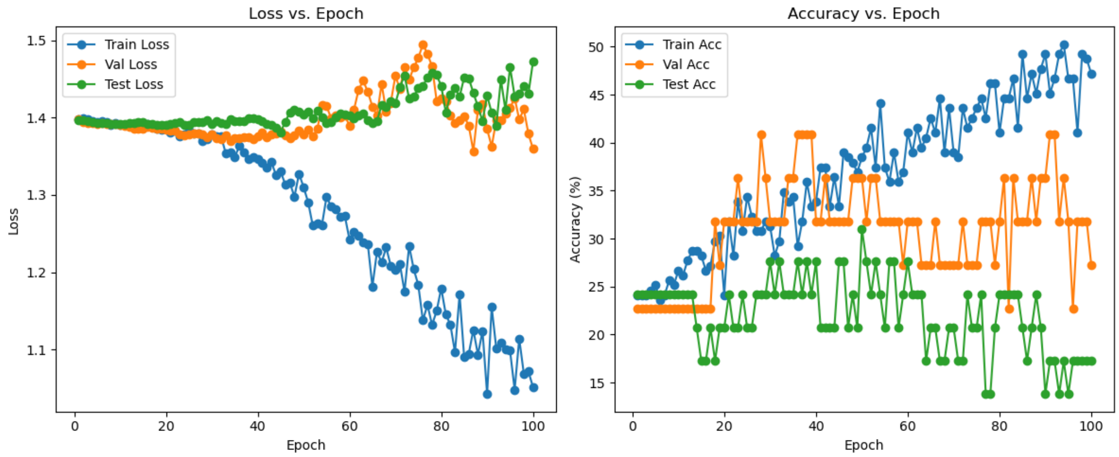

1.9 test 5. No Quantvolution (classic decision layer) + Quantfilter 4x4

[510]:
```
n_epochs = 100   # Number of optimization epochs
n_layers = 1    # Number of random layers
n_train  = 195    # Size of the train dataset
n_test   = 29     # Size of the test dataset
n_val    = 22     # Size of the validation dataset

SAVE_PATH = "/Users/h4/Desktop/quantum filters_research/
↪Quantvolution_Essential_tremor/Ablation/Quantvolution_Essential_tremor/
↪test_2/" # Data saving folder
PREPROCESS = True           # If False, skip quantum processing and load data␣
↪from SAVE_PATH
np.random.seed(0)           # Seed for NumPy random number generator
tf.random.set_seed(0)       # Seed for TensorFlow random number generator
```

[511]:
```
# Load pre-processed images
q_train_images = np.load(SAVE_PATH + "q_train_images_test_2.npy")
q_val_images   = np.load(SAVE_PATH + "q_val_images_test_2.npy")
q_test_images  = np.load(SAVE_PATH + "q_test_images_test_2.npy")
```

[512]:
```
X_train = torch.tensor(q_train_images)
y_train = torch.tensor(train_labels)
```

```
X_val = torch.tensor(q_val_images)
y_val = torch.tensor(val_labels)
X_test = torch.tensor(q_test_images)
y_test = torch.tensor(test_labels)
```

```
/var/folders/nw/k_k0_cbj7vl_npdmryvhl53c0000gn/T/ipykernel_70899/1747496059.py:2
: UserWarning: To copy construct from a tensor, it is recommended to use
sourceTensor.clone().detach() or
sourceTensor.clone().detach().requires_grad_(True), rather than
torch.tensor(sourceTensor).
  y_train = torch.tensor(train_labels)
/var/folders/nw/k_k0_cbj7vl_npdmryvhl53c0000gn/T/ipykernel_70899/1747496059.py:4
: UserWarning: To copy construct from a tensor, it is recommended to use
sourceTensor.clone().detach() or
sourceTensor.clone().detach().requires_grad_(True), rather than
torch.tensor(sourceTensor).
  y_val = torch.tensor(val_labels)
/var/folders/nw/k_k0_cbj7vl_npdmryvhl53c0000gn/T/ipykernel_70899/1747496059.py:6
: UserWarning: To copy construct from a tensor, it is recommended to use
sourceTensor.clone().detach() or
sourceTensor.clone().detach().requires_grad_(True), rather than
torch.tensor(sourceTensor).
  y_test = torch.tensor(test_labels)
```

[513]:

```
import torch
from torch.utils.data import Dataset, DataLoader

class CustomTensorDataset(Dataset):
    """TensorDataset with support for transforms."""
    def __init__(self, tensors, transform=None):
        assert all(tensors[0].shape[0] == tensor.shape[0] for tensor in␣
 ↪tensors), "All tensors must have the same first dimension"
        self.tensors = tensors
        self.transform = transform

    def __getitem__(self, index):
        x = self.tensors[0][index]
        if self.transform:
            x = self.transform(x)
        y = self.tensors[1][index]
        return x, y

    def __len__(self):
        return self.tensors[0].shape[0]

# Convert to PyTorch tensors
X_train_tensor = torch.tensor(X_train, dtype=torch.float32)
```

```python
y_train_tensor = torch.tensor(y_train, dtype=torch.long)
X_test_tensor = torch.tensor(X_test, dtype=torch.float32)
y_test_tensor = torch.tensor(y_test, dtype=torch.long)
X_val_tensor = torch.tensor(X_val, dtype=torch.float32)
y_val_tensor = torch.tensor(y_val, dtype=torch.long)

# Create dataset and dataloader
train_dataset = CustomTensorDataset(tensors=(X_train_tensor, y_train_tensor),␣
↪transform=None)
train_loader = DataLoader(train_dataset, batch_size=1, shuffle=True)

test_dataset = CustomTensorDataset(tensors=(X_test_tensor, y_test_tensor),␣
↪transform=None)
test_loader = DataLoader(test_dataset, batch_size=1, shuffle=True)

val_dataset = CustomTensorDataset(tensors=(X_val_tensor, y_val_tensor),␣
↪transform=None)
val_loader = DataLoader(val_dataset, batch_size=1, shuffle=True)

# Print dataset size to check
print("Train dataset size:", len(train_dataset))
print("Test dataset size:", len(test_dataset))
print("Val dataset size:", len(val_dataset))
```

```
Train dataset size: 195
Test dataset size: 29
Val dataset size: 22
```

```
/var/folders/nw/k_k0_cbj7vl_npdmryvhl53c0000gn/T/ipykernel_70899/522783547.py:22
: UserWarning: To copy construct from a tensor, it is recommended to use
sourceTensor.clone().detach() or
sourceTensor.clone().detach().requires_grad_(True), rather than
torch.tensor(sourceTensor).
  X_train_tensor = torch.tensor(X_train, dtype=torch.float32)
/var/folders/nw/k_k0_cbj7vl_npdmryvhl53c0000gn/T/ipykernel_70899/522783547.py:23
: UserWarning: To copy construct from a tensor, it is recommended to use
sourceTensor.clone().detach() or
sourceTensor.clone().detach().requires_grad_(True), rather than
torch.tensor(sourceTensor).
  y_train_tensor = torch.tensor(y_train, dtype=torch.long)
/var/folders/nw/k_k0_cbj7vl_npdmryvhl53c0000gn/T/ipykernel_70899/522783547.py:24
: UserWarning: To copy construct from a tensor, it is recommended to use
sourceTensor.clone().detach() or
sourceTensor.clone().detach().requires_grad_(True), rather than
torch.tensor(sourceTensor).
  X_test_tensor = torch.tensor(X_test, dtype=torch.float32)
/var/folders/nw/k_k0_cbj7vl_npdmryvhl53c0000gn/T/ipykernel_70899/522783547.py:25
: UserWarning: To copy construct from a tensor, it is recommended to use
```

```
sourceTensor.clone().detach() or
sourceTensor.clone().detach().requires_grad_(True), rather than
torch.tensor(sourceTensor).
  y_test_tensor = torch.tensor(y_test, dtype=torch.long)
/var/folders/nw/k_k0_cbj7vl_npdmryvhl53c0000gn/T/ipykernel_70899/522783547.py:26
: UserWarning: To copy construct from a tensor, it is recommended to use
sourceTensor.clone().detach() or
sourceTensor.clone().detach().requires_grad_(True), rather than
torch.tensor(sourceTensor).
  X_val_tensor = torch.tensor(X_val, dtype=torch.float32)
/var/folders/nw/k_k0_cbj7vl_npdmryvhl53c0000gn/T/ipykernel_70899/522783547.py:27
: UserWarning: To copy construct from a tensor, it is recommended to use
sourceTensor.clone().detach() or
sourceTensor.clone().detach().requires_grad_(True), rather than
torch.tensor(sourceTensor).
  y_val_tensor = torch.tensor(y_val, dtype=torch.long)
```

[516]:

```
import time
import numpy as np
import torch
import torch.nn as nn
import torch.optim as optim
import torch.nn.functional as F
from torch.utils.data import DataLoader, TensorDataset
import matplotlib.pyplot as plt

# ------------------------------ #
# Load and Prepare Your Dataset
# ------------------------------ #
X_train = torch.randn(195, 54, 8, 4)
y_train = torch.randint(0, 4, (195,))  # Using class indices (not one-hot)
X_val = torch.randn(22, 54, 8, 4)
y_val = torch.randint(0, 4, (22,))
X_test = torch.randn(29, 54, 8, 4)
y_test = torch.randint(0, 4, (29,))

# Create TensorDatasets and DataLoaders
train_dataset = TensorDataset(X_train, y_train)
val_dataset = TensorDataset(X_val, y_val)
test_dataset = TensorDataset(X_test, y_test)

train_loader = DataLoader(train_dataset, batch_size=4, shuffle=True)
val_loader = DataLoader(val_dataset, batch_size=4, shuffle=False)
test_loader = DataLoader(test_dataset, batch_size=4, shuffle=False)

##############################################
# Define the Fully Classical Network
```

```python
###############################################
class ClassicalNet(nn.Module):
    def __init__(self):
        super(ClassicalNet, self).__init__()
        self.conv1 = nn.Conv2d(54, 40, kernel_size=2, padding=1)
        self.bn1 = nn.BatchNorm2d(40)
        self.conv2 = nn.Conv2d(40, 16, kernel_size=2, padding=1)
        self.bn2 = nn.BatchNorm2d(16)
        self.max_pool = nn.MaxPool2d(2, stride=2)
        self.dropout = nn.Dropout(0.25)

        self.fc1 = nn.Linear(32, 28)
        self.fc2 = nn.Linear(28, 4)  #  Output layer for 4 classes

    def forward(self, x):
        x = F.relu(self.bn1(self.conv1(x)))
        x = self.max_pool(x)
        x = F.relu(self.bn2(self.conv2(x)))
        x = self.max_pool(x)
        x = x.view(x.size(0), -1)  # Flatten
        x = F.relu(self.fc1(x))
        x = self.dropout(x)
        x = self.fc2(x)  #  Output logits for 4 classes
        return x

###############################################
# Training Setup
###############################################
model = ClassicalNet()
optimizer = optim.Adam(model.parameters(), lr=1e-3)
loss_func = nn.CrossEntropyLoss()  # Multi-class classification

num_epochs = 100
train_losses, val_losses, test_losses = [], [], []
train_accuracies, val_accuracies, test_accuracies = [], [], []

start_time = time.time()

for epoch in range(num_epochs):
    # --- Training ---
    model.train()
    running_loss, correct_train, total_train = 0.0, 0, 0
    for data, target in train_loader:
        optimizer.zero_grad()
        outputs = model(data.float())  # (batch, 4)
        loss = loss_func(outputs, target.long())  # Target shape: (batch,)
        loss.backward()
```

```
        optimizer.step()

        running_loss += loss.item() * data.size(0)
        _, predicted = torch.max(outputs, 1)
        total_train += target.size(0)
        correct_train += (predicted == target).sum().item()

    train_losses.append(running_loss / total_train)
    train_accuracies.append(correct_train / total_train * 100.0)

    # --- Validation ---
    model.eval()
    running_val_loss, correct_val, total_val = 0.0, 0, 0
    with torch.no_grad():
        for data, target in val_loader:
            outputs = model(data.float())
            loss = loss_func(outputs, target.long())
            running_val_loss += loss.item() * data.size(0)
            _, predicted = torch.max(outputs, 1)
            total_val += target.size(0)
            correct_val += (predicted == target).sum().item()

    val_losses.append(running_val_loss / total_val)
    val_accuracies.append(correct_val / total_val * 100.0)

    # --- Test Evaluation ---
    running_test_loss, correct_test, total_test = 0.0, 0, 0
    with torch.no_grad():
        for data, target in test_loader:
            outputs = model(data.float())
            loss = loss_func(outputs, target.long())
            running_test_loss += loss.item() * data.size(0)
            _, predicted = torch.max(outputs, 1)
            total_test += target.size(0)
            correct_test += (predicted == target).sum().item()

    test_losses.append(running_test_loss / total_test)
    test_accuracies.append(correct_test / total_test * 100.0)

    print(f"Epoch [{epoch+1}/{num_epochs}] "
          f"Train Loss: {train_losses[-1]:.4f}, Train Acc: {train_accuracies[-1]:.2f}%  "
          f"Val Loss: {val_losses[-1]:.4f}, Val Acc: {val_accuracies[-1]:.2f}% "
          f"Test Loss: {test_losses[-1]:.4f}, Test Acc: {test_accuracies[-1]:.2f}%")
```

```python
end_time = time.time()
total_time = end_time - start_time

################################################
# Print Overall Metrics
################################################
print("\n--- Overall Metrics ---")
print(f"Train Loss: Mean = {np.mean(train_losses):.4f}, Std = {np.
  ↪std(train_losses):.4f}")
print(f"Train Accuracy: Mean = {np.mean(train_accuracies):.2f}%, Std = {np.
  ↪std(train_accuracies):.2f}%")
print(f"Validation Loss: Mean = {np.mean(val_losses):.4f}, Std = {np.
  ↪std(val_losses):.4f}")
print(f"Validation Accuracy: Mean = {np.mean(val_accuracies):.2f}%, Std = {np.
  ↪std(val_accuracies):.2f}%")
print(f"Test Loss: Mean = {np.mean(test_losses):.4f}, Std = {np.
  ↪std(test_losses):.4f}")
print(f"Test Accuracy: Mean = {np.mean(test_accuracies):.2f}%, Std = {np.
  ↪std(test_accuracies):.2f}%")
print(f"Total Computation Time: {total_time:.2f} seconds")

################################################
# Plot Loss Curves
################################################
epochs_range = range(1, num_epochs+1)
plt.figure(figsize=(12, 5))
plt.subplot(1, 2, 1)
plt.plot(epochs_range, train_losses, label="Train Loss", marker='o')
plt.plot(epochs_range, val_losses, label="Val Loss", marker='o')
plt.plot(epochs_range, test_losses, label="Test Loss", marker='o')
plt.xlabel("Epoch")
plt.ylabel("Loss")
plt.title("Loss vs. Epoch")
plt.legend()

################################################
# Plot Accuracy Curves
################################################
plt.subplot(1, 2, 2)
plt.plot(epochs_range, train_accuracies, label="Train Acc", marker='o')
plt.plot(epochs_range, val_accuracies, label="Val Acc", marker='o')
plt.plot(epochs_range, test_accuracies, label="Test Acc", marker='o')
plt.xlabel("Epoch")
plt.ylabel("Accuracy (%)")
plt.title("Accuracy vs. Epoch")
plt.legend()
```

```
plt.tight_layout()
plt.show()
```

```
Epoch [1/100] Train Loss: 1.4040, Train Acc: 27.69%  Val Loss: 1.4170, Val Acc:
27.27%  Test Loss: 1.3980, Test Acc: 20.69%
Epoch [2/100] Train Loss: 1.2531, Train Acc: 45.64%  Val Loss: 1.5064, Val Acc:
27.27%  Test Loss: 1.4198, Test Acc: 27.59%
Epoch [3/100] Train Loss: 1.0767, Train Acc: 64.62%  Val Loss: 1.4745, Val Acc:
18.18%  Test Loss: 1.3941, Test Acc: 27.59%
Epoch [4/100] Train Loss: 0.7589, Train Acc: 82.56%  Val Loss: 1.6364, Val Acc:
18.18%  Test Loss: 1.4956, Test Acc: 27.59%
Epoch [5/100] Train Loss: 0.4750, Train Acc: 89.74%  Val Loss: 1.7303, Val Acc:
18.18%  Test Loss: 1.6097, Test Acc: 27.59%
Epoch [6/100] Train Loss: 0.2713, Train Acc: 96.92%  Val Loss: 1.8265, Val Acc:
22.73%  Test Loss: 1.7612, Test Acc: 17.24%
Epoch [7/100] Train Loss: 0.1255, Train Acc: 98.97%  Val Loss: 2.0678, Val Acc:
18.18%  Test Loss: 1.8849, Test Acc: 20.69%
Epoch [8/100] Train Loss: 0.0836, Train Acc: 98.46%  Val Loss: 2.1271, Val Acc:
18.18%  Test Loss: 1.9919, Test Acc: 20.69%
Epoch [9/100] Train Loss: 0.0547, Train Acc: 100.00%  Val Loss: 2.1080, Val Acc:
27.27%  Test Loss: 2.0895, Test Acc: 24.14%
Epoch [10/100] Train Loss: 0.0299, Train Acc: 100.00%  Val Loss: 2.3306, Val
Acc: 27.27%  Test Loss: 2.2433, Test Acc: 24.14%
Epoch [11/100] Train Loss: 0.0269, Train Acc: 100.00%  Val Loss: 2.3920, Val
Acc: 27.27%  Test Loss: 2.2241, Test Acc: 27.59%
Epoch [12/100] Train Loss: 0.0226, Train Acc: 100.00%  Val Loss: 2.5868, Val
Acc: 22.73%  Test Loss: 2.2816, Test Acc: 20.69%
Epoch [13/100] Train Loss: 0.0158, Train Acc: 100.00%  Val Loss: 2.5599, Val
Acc: 27.27%  Test Loss: 2.2588, Test Acc: 27.59%
Epoch [14/100] Train Loss: 0.0103, Train Acc: 100.00%  Val Loss: 2.7196, Val
Acc: 27.27%  Test Loss: 2.3517, Test Acc: 27.59%
Epoch [15/100] Train Loss: 0.0128, Train Acc: 100.00%  Val Loss: 2.8385, Val
Acc: 27.27%  Test Loss: 2.4860, Test Acc: 27.59%
Epoch [16/100] Train Loss: 0.0143, Train Acc: 100.00%  Val Loss: 2.7595, Val
Acc: 22.73%  Test Loss: 2.5182, Test Acc: 20.69%
Epoch [17/100] Train Loss: 0.0187, Train Acc: 99.49%  Val Loss: 2.7974, Val Acc:
22.73%  Test Loss: 2.6216, Test Acc: 27.59%
Epoch [18/100] Train Loss: 0.0117, Train Acc: 100.00%  Val Loss: 2.8027, Val
Acc: 27.27%  Test Loss: 2.5742, Test Acc: 24.14%
Epoch [19/100] Train Loss: 0.0418, Train Acc: 98.46%  Val Loss: 3.5509, Val Acc:
31.82%  Test Loss: 3.4534, Test Acc: 27.59%
Epoch [20/100] Train Loss: 0.0258, Train Acc: 100.00%  Val Loss: 2.5881, Val
Acc: 22.73%  Test Loss: 2.7467, Test Acc: 27.59%
Epoch [21/100] Train Loss: 0.0122, Train Acc: 100.00%  Val Loss: 2.6809, Val
Acc: 22.73%  Test Loss: 2.9817, Test Acc: 24.14%
Epoch [22/100] Train Loss: 0.0077, Train Acc: 100.00%  Val Loss: 2.6139, Val
```

Acc: 18.18% Test Loss: 2.8168, Test Acc: 20.69%
Epoch [23/100] Train Loss: 0.0093, Train Acc: 100.00% Val Loss: 2.6629, Val Acc: 22.73% Test Loss: 2.9565, Test Acc: 17.24%
Epoch [24/100] Train Loss: 0.0082, Train Acc: 100.00% Val Loss: 2.9867, Val Acc: 13.64% Test Loss: 3.2057, Test Acc: 13.79%
Epoch [25/100] Train Loss: 0.0065, Train Acc: 100.00% Val Loss: 2.9391, Val Acc: 22.73% Test Loss: 3.2220, Test Acc: 13.79%
Epoch [26/100] Train Loss: 0.0064, Train Acc: 100.00% Val Loss: 3.0277, Val Acc: 18.18% Test Loss: 3.2438, Test Acc: 17.24%
Epoch [27/100] Train Loss: 0.0094, Train Acc: 100.00% Val Loss: 2.9226, Val Acc: 22.73% Test Loss: 3.1675, Test Acc: 6.90%
Epoch [28/100] Train Loss: 0.0054, Train Acc: 100.00% Val Loss: 3.0974, Val Acc: 22.73% Test Loss: 3.2699, Test Acc: 13.79%
Epoch [29/100] Train Loss: 0.0036, Train Acc: 100.00% Val Loss: 3.0466, Val Acc: 13.64% Test Loss: 2.9907, Test Acc: 13.79%
Epoch [30/100] Train Loss: 0.0046, Train Acc: 100.00% Val Loss: 3.3184, Val Acc: 22.73% Test Loss: 3.2821, Test Acc: 17.24%
Epoch [31/100] Train Loss: 0.0055, Train Acc: 100.00% Val Loss: 3.1341, Val Acc: 22.73% Test Loss: 3.3384, Test Acc: 17.24%
Epoch [32/100] Train Loss: 0.0041, Train Acc: 100.00% Val Loss: 3.1856, Val Acc: 13.64% Test Loss: 3.4162, Test Acc: 17.24%
Epoch [33/100] Train Loss: 0.0080, Train Acc: 99.49% Val Loss: 3.3883, Val Acc: 18.18% Test Loss: 3.3599, Test Acc: 20.69%
Epoch [34/100] Train Loss: 0.0047, Train Acc: 100.00% Val Loss: 3.3542, Val Acc: 18.18% Test Loss: 3.4532, Test Acc: 20.69%
Epoch [35/100] Train Loss: 0.0014, Train Acc: 100.00% Val Loss: 3.2734, Val Acc: 22.73% Test Loss: 3.4107, Test Acc: 20.69%
Epoch [36/100] Train Loss: 0.0023, Train Acc: 100.00% Val Loss: 3.3832, Val Acc: 22.73% Test Loss: 3.5366, Test Acc: 27.59%
Epoch [37/100] Train Loss: 0.0017, Train Acc: 100.00% Val Loss: 3.4054, Val Acc: 13.64% Test Loss: 3.5295, Test Acc: 17.24%
Epoch [38/100] Train Loss: 0.0024, Train Acc: 100.00% Val Loss: 3.4521, Val Acc: 13.64% Test Loss: 3.5823, Test Acc: 13.79%
Epoch [39/100] Train Loss: 0.0021, Train Acc: 100.00% Val Loss: 3.5738, Val Acc: 27.27% Test Loss: 3.7106, Test Acc: 20.69%
Epoch [40/100] Train Loss: 0.0025, Train Acc: 100.00% Val Loss: 3.9014, Val Acc: 27.27% Test Loss: 4.0055, Test Acc: 20.69%
Epoch [41/100] Train Loss: 0.0006, Train Acc: 100.00% Val Loss: 3.6959, Val Acc: 27.27% Test Loss: 3.8025, Test Acc: 17.24%
Epoch [42/100] Train Loss: 0.0023, Train Acc: 100.00% Val Loss: 3.5964, Val Acc: 27.27% Test Loss: 3.8547, Test Acc: 20.69%
Epoch [43/100] Train Loss: 0.0073, Train Acc: 100.00% Val Loss: 2.9316, Val Acc: 13.64% Test Loss: 3.6142, Test Acc: 13.79%
Epoch [44/100] Train Loss: 0.0044, Train Acc: 100.00% Val Loss: 3.0540, Val Acc: 13.64% Test Loss: 3.6170, Test Acc: 10.34%
Epoch [45/100] Train Loss: 0.0032, Train Acc: 100.00% Val Loss: 3.5748, Val Acc: 27.27% Test Loss: 4.0837, Test Acc: 24.14%
Epoch [46/100] Train Loss: 0.0016, Train Acc: 100.00% Val Loss: 3.4656, Val

Acc: 22.73% Test Loss: 3.9503, Test Acc: 20.69%
Epoch [47/100] Train Loss: 0.0131, Train Acc: 99.49% Val Loss: 4.2189, Val Acc: 22.73% Test Loss: 4.3785, Test Acc: 20.69%
Epoch [48/100] Train Loss: 0.0029, Train Acc: 100.00% Val Loss: 4.0402, Val Acc: 18.18% Test Loss: 4.0989, Test Acc: 24.14%
Epoch [49/100] Train Loss: 0.0010, Train Acc: 100.00% Val Loss: 3.9135, Val Acc: 9.09% Test Loss: 3.9112, Test Acc: 17.24%
Epoch [50/100] Train Loss: 0.0011, Train Acc: 100.00% Val Loss: 3.9708, Val Acc: 9.09% Test Loss: 4.0403, Test Acc: 24.14%
Epoch [51/100] Train Loss: 0.0015, Train Acc: 100.00% Val Loss: 4.3230, Val Acc: 13.64% Test Loss: 4.4383, Test Acc: 20.69%
Epoch [52/100] Train Loss: 0.0023, Train Acc: 100.00% Val Loss: 4.2511, Val Acc: 9.09% Test Loss: 4.4510, Test Acc: 20.69%
Epoch [53/100] Train Loss: 0.0038, Train Acc: 100.00% Val Loss: 3.7221, Val Acc: 22.73% Test Loss: 4.0721, Test Acc: 17.24%
Epoch [54/100] Train Loss: 0.0052, Train Acc: 100.00% Val Loss: 4.2340, Val Acc: 13.64% Test Loss: 3.9284, Test Acc: 20.69%
Epoch [55/100] Train Loss: 0.0023, Train Acc: 100.00% Val Loss: 4.0188, Val Acc: 9.09% Test Loss: 4.0591, Test Acc: 13.79%
Epoch [56/100] Train Loss: 0.0008, Train Acc: 100.00% Val Loss: 3.9503, Val Acc: 9.09% Test Loss: 4.0115, Test Acc: 10.34%
Epoch [57/100] Train Loss: 0.0030, Train Acc: 100.00% Val Loss: 4.7352, Val Acc: 9.09% Test Loss: 4.4232, Test Acc: 20.69%
Epoch [58/100] Train Loss: 0.0049, Train Acc: 100.00% Val Loss: 4.3102, Val Acc: 9.09% Test Loss: 3.9428, Test Acc: 13.79%
Epoch [59/100] Train Loss: 0.0017, Train Acc: 100.00% Val Loss: 4.1713, Val Acc: 13.64% Test Loss: 3.9837, Test Acc: 17.24%
Epoch [60/100] Train Loss: 0.0013, Train Acc: 100.00% Val Loss: 4.2698, Val Acc: 18.18% Test Loss: 4.1088, Test Acc: 17.24%
Epoch [61/100] Train Loss: 0.0021, Train Acc: 100.00% Val Loss: 4.3671, Val Acc: 9.09% Test Loss: 4.1251, Test Acc: 20.69%
Epoch [62/100] Train Loss: 0.0031, Train Acc: 100.00% Val Loss: 4.3767, Val Acc: 13.64% Test Loss: 4.2390, Test Acc: 17.24%
Epoch [63/100] Train Loss: 0.1959, Train Acc: 92.82% Val Loss: 5.7032, Val Acc: 13.64% Test Loss: 5.8721, Test Acc: 27.59%
Epoch [64/100] Train Loss: 0.5711, Train Acc: 81.54% Val Loss: 2.4950, Val Acc: 22.73% Test Loss: 3.1415, Test Acc: 10.34%
Epoch [65/100] Train Loss: 0.1022, Train Acc: 96.92% Val Loss: 2.8656, Val Acc: 22.73% Test Loss: 3.0863, Test Acc: 27.59%
Epoch [66/100] Train Loss: 0.0296, Train Acc: 99.49% Val Loss: 3.5514, Val Acc: 13.64% Test Loss: 3.6058, Test Acc: 24.14%
Epoch [67/100] Train Loss: 0.0137, Train Acc: 100.00% Val Loss: 3.1628, Val Acc: 18.18% Test Loss: 3.3309, Test Acc: 27.59%
Epoch [68/100] Train Loss: 0.0136, Train Acc: 100.00% Val Loss: 3.3355, Val Acc: 22.73% Test Loss: 3.4907, Test Acc: 20.69%
Epoch [69/100] Train Loss: 0.0048, Train Acc: 100.00% Val Loss: 3.4556, Val Acc: 22.73% Test Loss: 3.6289, Test Acc: 20.69%
Epoch [70/100] Train Loss: 0.0065, Train Acc: 100.00% Val Loss: 3.6060, Val

```
Acc: 18.18%  Test Loss: 3.7570, Test Acc: 20.69%
Epoch [71/100] Train Loss: 0.0037, Train Acc: 100.00%  Val Loss: 3.7229, Val
Acc: 27.27%  Test Loss: 3.8684, Test Acc: 20.69%
Epoch [72/100] Train Loss: 0.0036, Train Acc: 100.00%  Val Loss: 3.7688, Val
Acc: 27.27%  Test Loss: 3.8745, Test Acc: 20.69%
Epoch [73/100] Train Loss: 0.0025, Train Acc: 100.00%  Val Loss: 3.7816, Val
Acc: 27.27%  Test Loss: 3.9421, Test Acc: 24.14%
Epoch [74/100] Train Loss: 0.0039, Train Acc: 100.00%  Val Loss: 3.7088, Val
Acc: 22.73%  Test Loss: 3.9203, Test Acc: 20.69%
Epoch [75/100] Train Loss: 0.0033, Train Acc: 100.00%  Val Loss: 3.7763, Val
Acc: 22.73%  Test Loss: 4.0013, Test Acc: 24.14%
Epoch [76/100] Train Loss: 0.0039, Train Acc: 100.00%  Val Loss: 3.9450, Val
Acc: 22.73%  Test Loss: 4.1115, Test Acc: 20.69%
Epoch [77/100] Train Loss: 0.0024, Train Acc: 100.00%  Val Loss: 3.9254, Val
Acc: 22.73%  Test Loss: 4.1669, Test Acc: 20.69%
Epoch [78/100] Train Loss: 0.0014, Train Acc: 100.00%  Val Loss: 3.9517, Val
Acc: 22.73%  Test Loss: 4.2350, Test Acc: 20.69%
Epoch [79/100] Train Loss: 0.0058, Train Acc: 100.00%  Val Loss: 4.0433, Val
Acc: 22.73%  Test Loss: 4.2395, Test Acc: 20.69%
Epoch [80/100] Train Loss: 0.0018, Train Acc: 100.00%  Val Loss: 4.0615, Val
Acc: 22.73%  Test Loss: 4.2381, Test Acc: 20.69%
Epoch [81/100] Train Loss: 0.0022, Train Acc: 100.00%  Val Loss: 4.0374, Val
Acc: 22.73%  Test Loss: 4.1857, Test Acc: 20.69%
Epoch [82/100] Train Loss: 0.0036, Train Acc: 100.00%  Val Loss: 4.2060, Val
Acc: 22.73%  Test Loss: 4.3920, Test Acc: 20.69%
Epoch [83/100] Train Loss: 0.0015, Train Acc: 100.00%  Val Loss: 4.1798, Val
Acc: 22.73%  Test Loss: 4.3475, Test Acc: 20.69%
Epoch [84/100] Train Loss: 0.0017, Train Acc: 100.00%  Val Loss: 4.1340, Val
Acc: 22.73%  Test Loss: 4.2804, Test Acc: 24.14%
Epoch [85/100] Train Loss: 0.0017, Train Acc: 100.00%  Val Loss: 4.1817, Val
Acc: 22.73%  Test Loss: 4.3851, Test Acc: 20.69%
Epoch [86/100] Train Loss: 0.0010, Train Acc: 100.00%  Val Loss: 4.1203, Val
Acc: 22.73%  Test Loss: 4.3131, Test Acc: 20.69%
Epoch [87/100] Train Loss: 0.0006, Train Acc: 100.00%  Val Loss: 4.2593, Val
Acc: 22.73%  Test Loss: 4.4739, Test Acc: 20.69%
Epoch [88/100] Train Loss: 0.0006, Train Acc: 100.00%  Val Loss: 4.3655, Val
Acc: 22.73%  Test Loss: 4.5597, Test Acc: 20.69%
Epoch [89/100] Train Loss: 0.0078, Train Acc: 99.49%  Val Loss: 3.6485, Val Acc:
27.27%  Test Loss: 4.0280, Test Acc: 27.59%
Epoch [90/100] Train Loss: 0.0036, Train Acc: 100.00%  Val Loss: 3.6825, Val
Acc: 27.27%  Test Loss: 4.0941, Test Acc: 24.14%
Epoch [91/100] Train Loss: 0.0023, Train Acc: 100.00%  Val Loss: 3.9057, Val
Acc: 22.73%  Test Loss: 4.2338, Test Acc: 24.14%
Epoch [92/100] Train Loss: 0.0008, Train Acc: 100.00%  Val Loss: 3.9608, Val
Acc: 22.73%  Test Loss: 4.2173, Test Acc: 20.69%
Epoch [93/100] Train Loss: 0.0018, Train Acc: 100.00%  Val Loss: 3.9844, Val
Acc: 22.73%  Test Loss: 4.2485, Test Acc: 20.69%
Epoch [94/100] Train Loss: 0.0013, Train Acc: 100.00%  Val Loss: 4.0356, Val
```

```
Acc: 22.73%  Test Loss: 4.3893, Test Acc: 17.24%
Epoch [95/100] Train Loss: 0.0022, Train Acc: 100.00%  Val Loss: 4.2298, Val
Acc: 22.73%  Test Loss: 4.5230, Test Acc: 17.24%
Epoch [96/100] Train Loss: 0.0019, Train Acc: 100.00%  Val Loss: 4.0811, Val
Acc: 22.73%  Test Loss: 4.4425, Test Acc: 20.69%
Epoch [97/100] Train Loss: 0.0007, Train Acc: 100.00%  Val Loss: 4.1787, Val
Acc: 22.73%  Test Loss: 4.5001, Test Acc: 20.69%
Epoch [98/100] Train Loss: 0.0006, Train Acc: 100.00%  Val Loss: 4.1530, Val
Acc: 22.73%  Test Loss: 4.5278, Test Acc: 20.69%
Epoch [99/100] Train Loss: 0.0008, Train Acc: 100.00%  Val Loss: 4.2468, Val
Acc: 22.73%  Test Loss: 4.5332, Test Acc: 20.69%
Epoch [100/100] Train Loss: 0.0018, Train Acc: 100.00%  Val Loss: 4.3168, Val
Acc: 22.73%  Test Loss: 4.6040, Test Acc: 20.69%

--- Overall Metrics ---
Train Loss: Mean = 0.0692, Std = 0.2347
Train Accuracy: Mean = 97.72%, Std = 9.87%
Validation Loss: Mean = 3.4513, Std = 0.7994
Validation Accuracy: Mean = 20.82%, Std = 5.42%
Test Loss: Mean = 3.5621, Std = 0.8763
Test Accuracy: Mean = 20.72%, Std = 4.46%
Total Computation Time: 4.45 seconds
```

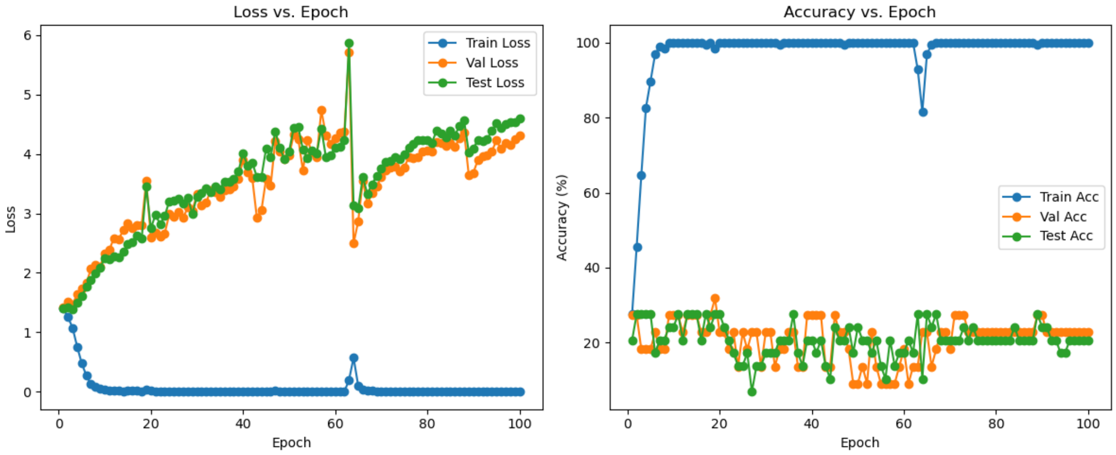

Supplement: Supplementary file 1 — Supplementary Information 1. [file 41598_2025_6359_MOESM1_ESM.pdf]
